# Recent Advances in Single-Electron-Transfer-Mediated Carbonylation

**DOI:** 10.1021/acs.chemrev.5c00664

**Published:** 2025-10-22

**Authors:** Le-Cheng Wang, Hefei Yang, Zhen-Wei Liu, Ren-Guan Miao, Ming Hou, Xiao-Feng Wu

**Affiliations:** Dalian National Laboratory for Clean Energy, Dalian Institute of Chemical Physics, Chinese Academy of Sciences, Dalian 116023, China; 28392 Leibniz-Institut für Katalyse e.V., Rostock 18059, Germany

## Abstract

Carbonylation reactions constitute one of the most powerful and widely utilized strategies for synthesizing carbonyl-containing compounds in organic chemistry. Among the mechanistic pathways explored, two-electron transfer (TET) processes have been extensively developed and industrially applied. However, besides their obvious advantages, their intrinsic limitations, such as reliance on precious metal catalysts and restricted compatibility with alkyl substrates, have prompted increasing interest in single-electron transfer (SET) alternatives. Alternatively, SET-mediated carbonylation bypasses the traditional oxidative addition step, generating highly reactive radical intermediates under milder reaction conditions, thus providing enhanced selectivity and broader substrate compatibility. This review offers a comprehensive overview of SET-mediated carbonylation chemistry from 2000 to July 2025, emphasizing mechanistic insights, catalytic systems, and synthetic applications. The objective is to establish a conceptual foundation for understanding recent advances and inspire further exploration into novel reactivity paradigms based on SET strategies within the realm of carbonylation chemistry.

## Graphical abstract

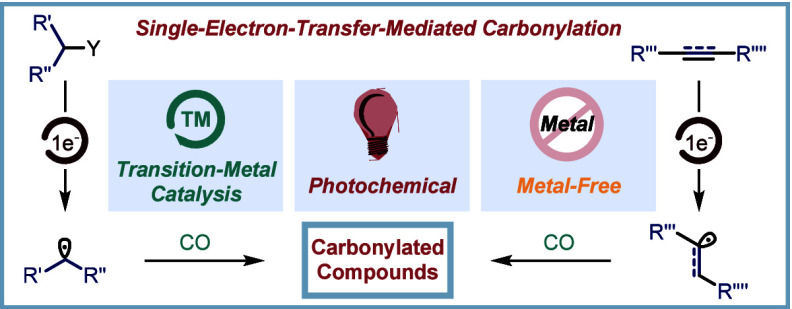

## 1 Introduction

Carbonylation reactions employing carbon monoxide (CO) as a readily available and versatile C1 synthon have emerged as indispensable tools for constructing diverse carbonyl-containing compounds from simple, accessible starting materials, finding extensive applications in both academic and industrial contexts.^1–6^ Over recent decades, the efficient and selective transformation of CO has become a cornerstone of organic synthesis, driven by its capability to enable precise formation of carbonyl functionalities crucial to pharmaceuticals, materials science, and related fields.^7–9^ Numerous innovative carbonylation methodologies have thus been developed, many successfully translated into industrial processes,^10–12^ including the Fischer–Tropsch synthesis,^13^ hydroformylation,^14^ and Monsanto/Cativa processes.^15,16^ These strategies facilitate both carbon–carbon and carbon-heteroatom bond formation and offer platforms for carbon chain elongation,^17–19^ allowing synthesis of valuable compounds such as aldehydes, ketones, esters, amides, carboxylic acids, and alcohols.^20–25^

Carbonylative coupling reactions generally proceed via two distinct mechanistic pathways. (1) Two-electron transfer (TET): Following Heck’s seminal report on palladium-catalyzed carbonylation in 1974, classical transition-metal-catalyzed carbonylation reactions based on classical two-electron oxidative addition and reductive elimination have experienced extensive development due to their well-defined mechanistic clarity and operational reliability.^26,27^ Nevertheless, their intrinsic limitations persist, such as mutual constraints among catalytic cycle steps,^28^ the strong π-acidic nature of CO reducing electron density at metal centers (thus impeding oxidative addition), the necessity for elevated temperatures relative to analogous cross-couplings, and limited substrate scope, especially with less reactive alkyl halides.^29^ Furthermore, these reactions frequently require strong nucleophiles to facilitate reductive elimination.^30^ Organometallic intermediates with open coordination sites are also prone to β-hydride elimination from C­(sp^3^) substrates, causing isomerization and diminished selectivity.^31^ (2) Single-electron transfer (SET): In contrast, SET-mediated carbonylation provides a mechanistically distinct alternative that circumvents traditional oxidative addition, enabling mild activation of otherwise inert bonds and overcoming electronic and steric limitations.^2,32^ SET-mediated carbonylation demonstrates excellent compatibility with primary, secondary, and tertiary alkyl halides and related precursors. Alkyl radicals generated by this pathway typically avoid β-hydride elimination, thus improving reaction selectivity and efficiency, positioning SET-based strategies as powerful complements to traditional metal-catalyzed approaches.

SET-mediated carbonylation has origins dating back to 1952, when Coffman and colleagues disclosed the copolymerization of ethylene and CO.^33^ After decades of dormancy, radical formylation was revisited and achieved in the 1990s.^34^ Subsequently, this breakthrough sparked widespread interest within the scientific community, driving researchers to explore and capitalize on the potential of these single-electron species. This led to a surge of studies aimed at disrupting the transition metal-catalyzed oxidative addition pathway to generate highly reactive single-electron radical species. Another significant advancement occurred with the discovery of atom-transfer carbonylation (ATC) in the 1990s, enabling synthesis of aliphatic esters and amides from alkyl iodides and CO, initially using high-energy radiation or radical initiators.^35,36^ Comprehensive discussions of early advancements were provided by Ryu and colleagues in reviews published in 1996 and 2001.^37,38^ Despite historical perceptions of SET-mediated carbonylation as chaotic and challenging to control, numerous practical advances in recent decades have significantly mitigated these concerns.^39–41^

Since the early 2000s, SET-mediated carbonylations catalyzed by transition metals or photocatalysts have rapidly evolved, particularly in transformations of unsaturated bonds (e.g., alkenes, alkynes) or strong bonds (e.g., C­(sp^3^)-X, C­(sp^3^)-H). Developing novel SET-mediated carbonylation reactions mediated by various transition metals and photocatalysts will require a comprehensive understanding of the possible interaction modes between different radicals and CO under a catalytic system.^42^ Due to single electron intermediates’ intrinsic reactivity, capturing or isolating such species remains challenging,^43,44^ complicating the clear elucidation of interactions between radicals and metal complexes. Typically, SET-mediated carbonylation proceeds via four catalytic modes (Figure 1c): (a) carbonylation of alkyl halides (single-electron reduction of C-X bonds), (b) carbonylation of alkanes (C–H bond activation via HAT (hydrogen atom transfer) process), (c) carbonylation of unsaturated bonds through radical relay mechanisms, and (d) carbonylation of unsaturated bonds initiated by organometallic addition. Single-electron intermediates can interact with CO through two pathways: either radicals add covalently to metal centers, generating stable alkyl–metal intermediates that undergo CO insertion, or radicals directly react with CO, forming acyl radicals. The latter possess electrophilicity derived from their π* orbital and nucleophilicity from their singly occupied molecular orbital (SOMO).^45–47^ In 1999, Chatgilialoglu, Crich, and Ryu’s comprehensive review systematically examined physicochemical properties, reaction kinetics, generation, and synthetic applications of acyl radicals.^48^

**Figure 1:**
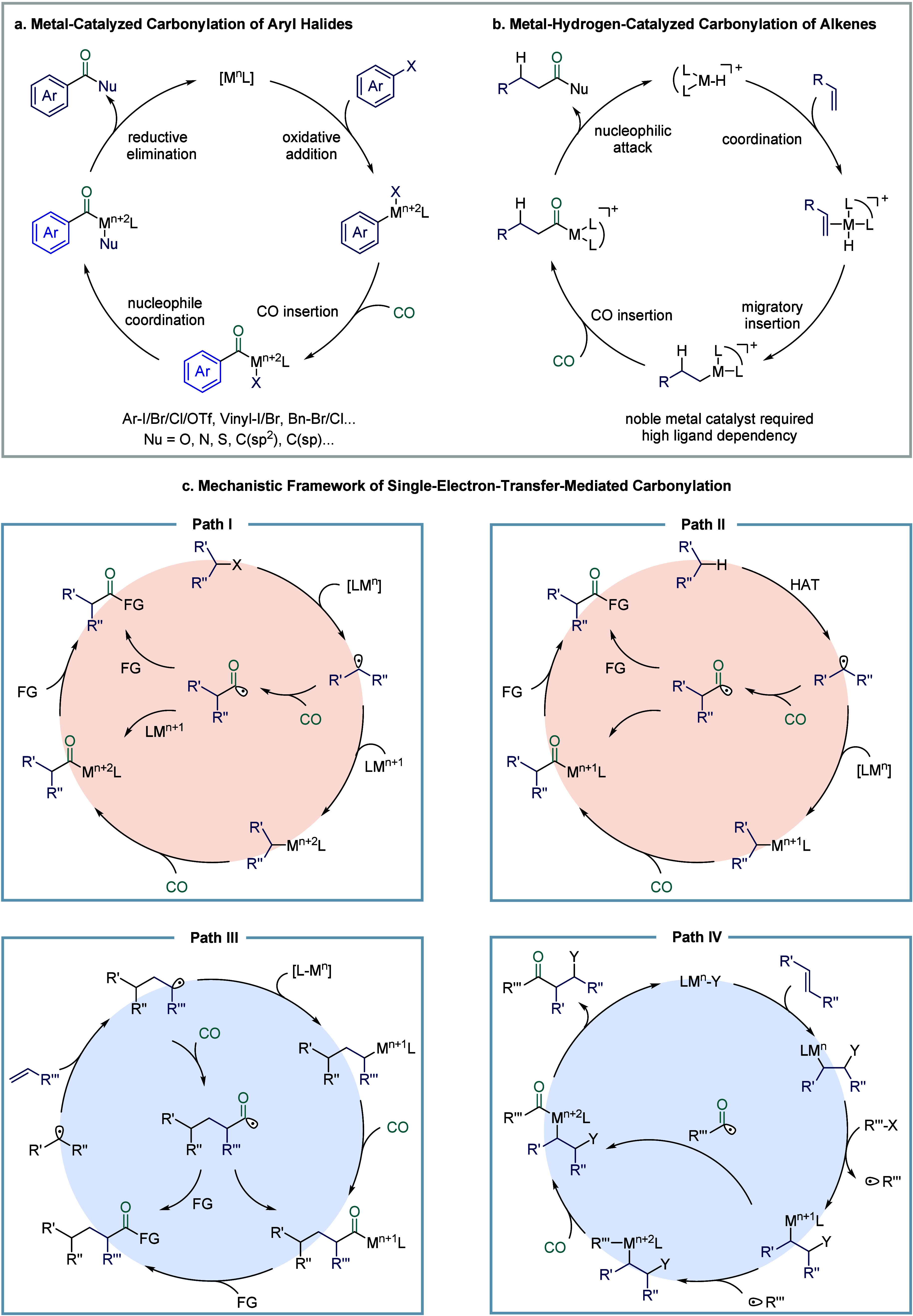
Catalytic cycles for TET-mediated carbonylation and SET-mediated carbonylation. a) Metal-catalyzed carbonylation of aryl halides. b) metal–hydrogen-catalyzed carbonylation of alkenes. c) Mechanistic framework of single-electron-transfer-mediated carbonylation.

While multiple mini reviews addressing specific reaction classes, catalytic systems, or mechanistic motifs have appeared over recent decades, a unified, comprehensive analysis covering the full breadth of this expanding domain remains conspicuously absent. Building on the foundational summaries of radical carbonylation by Ryu and co-workers,^37,38,48^ as well as recent significant advances in transition metal-catalyzed and visible-light-mediated carbonylation reactions, this review aims to fill this gap, offering a systematic, in-depth overview from 2000 to July 2025. It emphasizes key conceptual advances, representative reaction platforms, and emerging mechanistic paradigms. Particular attention is devoted to diverse carbonylation modes via SET under both metal-catalyzed and metal-free conditions, encompassing alkyl, aryl, heteroatom, and π-bond-containing substrates (Figure 2). Herein, we define SET-mediated carbonylation as transformations in which key single-electron radical intermediates generated from reaction substrates can either directly capture CO or form organometallic intermediates with metals that subsequently capture CO, ultimately affording carbonylated products. Through detailed analysis, we highlight how strategic radical generation, relay, and termination have enabled previously inaccessible bond formations and broadened the scope of carbonylative transformations. By situating recent advances within historical SET carbonylation development and providing forward-looking perspectives on emerging opportunities, this review serves as a timely resource for researchers aiming to explore, refine, and innovate within this dynamic field of synthetic chemistry. The contents are classified based on the catalyst used, from transition metal to metal free system, and subcatalogued by the chemical bond property, from saturated bond to unsaturated bond, from direct capture CO to multicomponent reaction.

**Figure 2:**
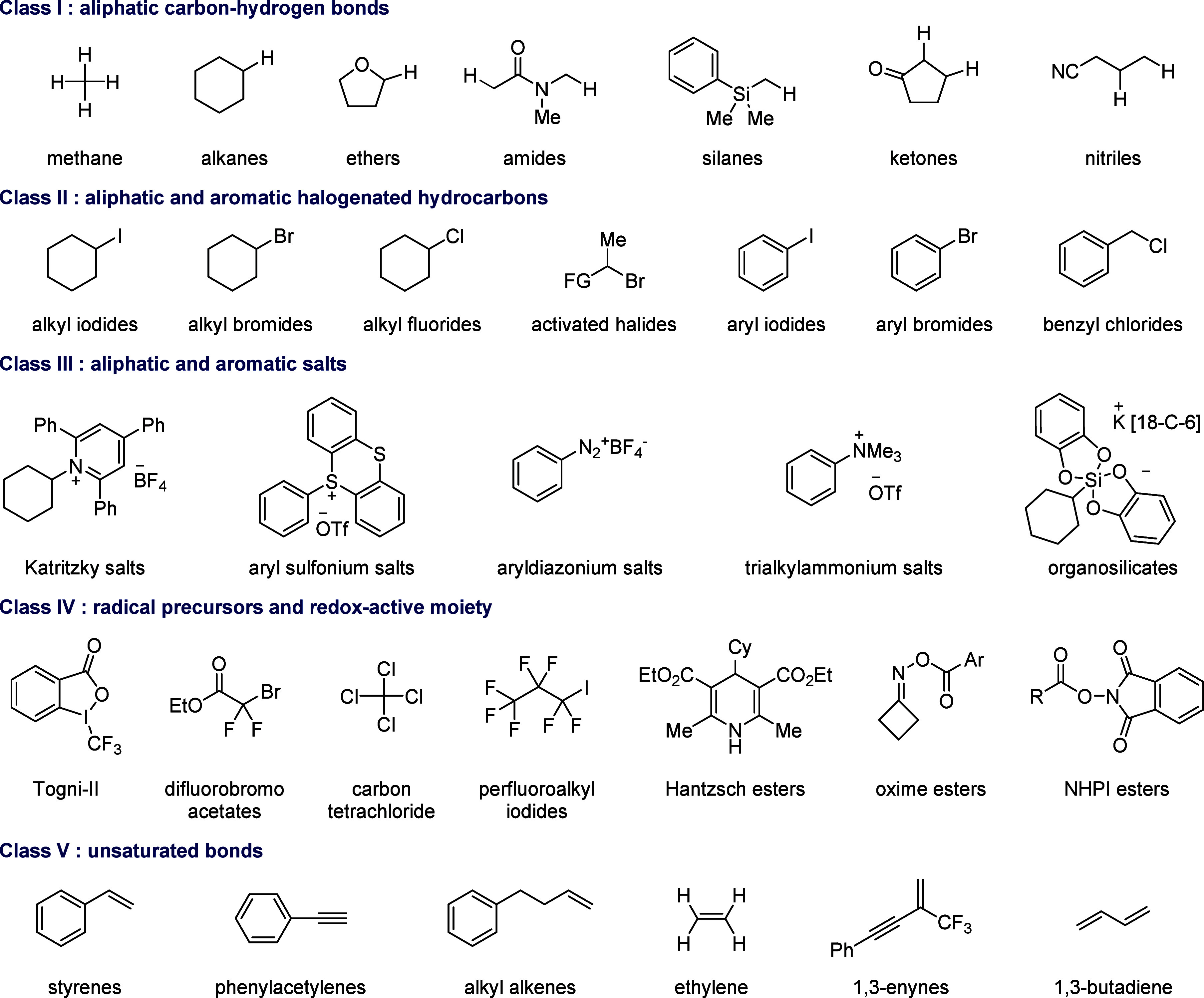
Representative substrates in SET-mediated carbonylation.

## 2 First-Row Transition Metals

3d transition metals, valued for their abundance in the Earth’s crust and environmental benignity, have recently emerged as effective homogeneous catalysts for a wide range of organic transformations.^49–53^ The development of 3d metal complexes has also inspired sustainable approaches for the controlled incorporation of CO into carbonylation reactions. These metals utilize both their 3d and 4s electrons in bonding and exhibit properties such as small atomic radii and short metal–carbon (M-C) bond lengths.^54^ As a result, their catalytic activity, accessible oxidation states, and magnetic properties are strongly influenced by the surrounding chemical environment of the 3d and 4s orbitals. Notably, 3d electrons readily participate in single-electron redox processes, enabling a variety of catalytic functions in reactions involving 1e^–^ species.^55^ Benefited from the advances in transition metal catalysis, the integration of 3d transition metals into carbonylation chemistry has significantly broadened the scope of synthetic methodologies.^56^ These metals not only facilitate the generation of reactive radical intermediates but also allow fine-tuning of their reactivity, thereby enabling efficient carbonylation processes. In particular, 3d transition metals have demonstrated considerable potential in SET-mediated carbonylation reactions due to their inherent aptitude for single-electron pathways.

Scandium, the first element in the 3d series, has an estimated crustal abundance of approximately 5 ppm. Although Sc chemistry is dominated by the +3 oxidation state, lower oxidation states such as +2, + 1, and even 0 have been reported in specialized complexes.^57,58^ Titanium, by contrast, is the second most abundant transition metal in the Earth’s crust and is prized for its low toxicity and favorable environmental profile, making it an appealing candidate for catalytic applications.^59,60^ Both scandium­(III) and titanium­(IV) salts, such as Sc­(OTf)_3_ and TiCl_4_, are well-known Lewis acids and have found broad utility in organic synthesis, particularly in Lewis acid-catalyzed processes.^61^ Nevertheless, despite these promising characteristics, there are currently no reported applications of either scandium or titanium in carbonylation chemistry. Vanadium, which can access oxidation states from −3 to +5, participates in both oxidative and reductive transformations.^62,63^ In 1999, Fujiwara and co-workers reported a seminal methane C­(sp^3^)-H carbonylation reaction catalyzed by vanadium, enabling the formation of acetic acid via a methyl radical intermediate.^64,65^ In addition, tungsten-doped vanadium dioxide has also found applications as a catalyst in various fields, particularly in hydrogenation catalysis.^66^ Chromium, exhibiting oxidation states from −2 to +6, is another versatile metal with relevance in catalysis. It readily forms stable carbonyl complexes with CO, some of which display catalytic activity, though typically via closed-shell, two-electron pathways rather than single-electron routes.^67,68^ Zinc is the final element in 3d transition metals and is generally not classified as a transition element, as its stable compounds in +2 oxidation state feature filled 3d electron shells.^69^ In addition, many organic transformations rely on stoichiometric amounts of zinc reagents, while zinc catalysis is less commonly employed compared to that of other transition metals.^70^ To date, no zinc-catalyzed carbonylation reactions have been reported. Compared to Sc, Ti, V, Cr, and Zn, the metals Mn, Fe, Co, Ni, and Cu exhibit superior reactivity in SET-mediated carbonylation. The following section focuses on mechanistic insights and experimental strategies for SET-mediated carbonylation processes catalyzed by these first-row transition metals under homogeneous conditions.

### 2.1 Titanium-Catalyzed System

Although titanium catalysts are highly efficient, environmentally friendly, and have demonstrated good catalytic activity in certain photocatalytic transformations, titanium-based carbonylation methods remain scarce. Titanium dioxide (TiO_2_), an inexpensive and abundantly available semiconductor, has been extensively utilized as a photocatalyst due to its excellent photostability, low toxicity, and favorable alignment of conduction and valence band energies.^71^ It plays a significant role in the photodecomposition of organic pollutants and in photocatalytic hydrogen generation.^72^ Upon light irradiation, electrons are promoted from the valence band (VB) to the conduction band (CB), resulting in the formation of electron–hole pairs. These charge carriers subsequently participate in surface-mediated redox reactions. In 2025, Chen and Wu collaboratively reported a light-induced perfluoroalkylative carbonylation of unactivated alkenes employing a recyclable photocatalyst (Scheme 1).^73^ Mechanistic studies revealed that TiO_2_ was photoexcited under Xe lamp irradiation, promoting electrons from the valence band to the conduction band and generating electron–hole pairs. The perfluoroalkyl radical **5** was subsequently produced from perfluoroalkyl iodides via single-electron transfer on the valence band. The resulting perfluoroalkyl radical added to the alkene to form radical intermediate **6**, which then captured CO to afford radical intermediate **7**. The Ti-based catalyst exhibited high activity and broad functional group tolerance. When internal alkenes were employed, the desired product **12** was obtained in 25% yield. Notably, this photocatalyst maintained high catalytic performance over multiple cycles. Furthermore, the system reduces the reliance on precious metals, avoids the need for ligands, and can be readily separated from the reaction mixture.

**Scheme 1:**
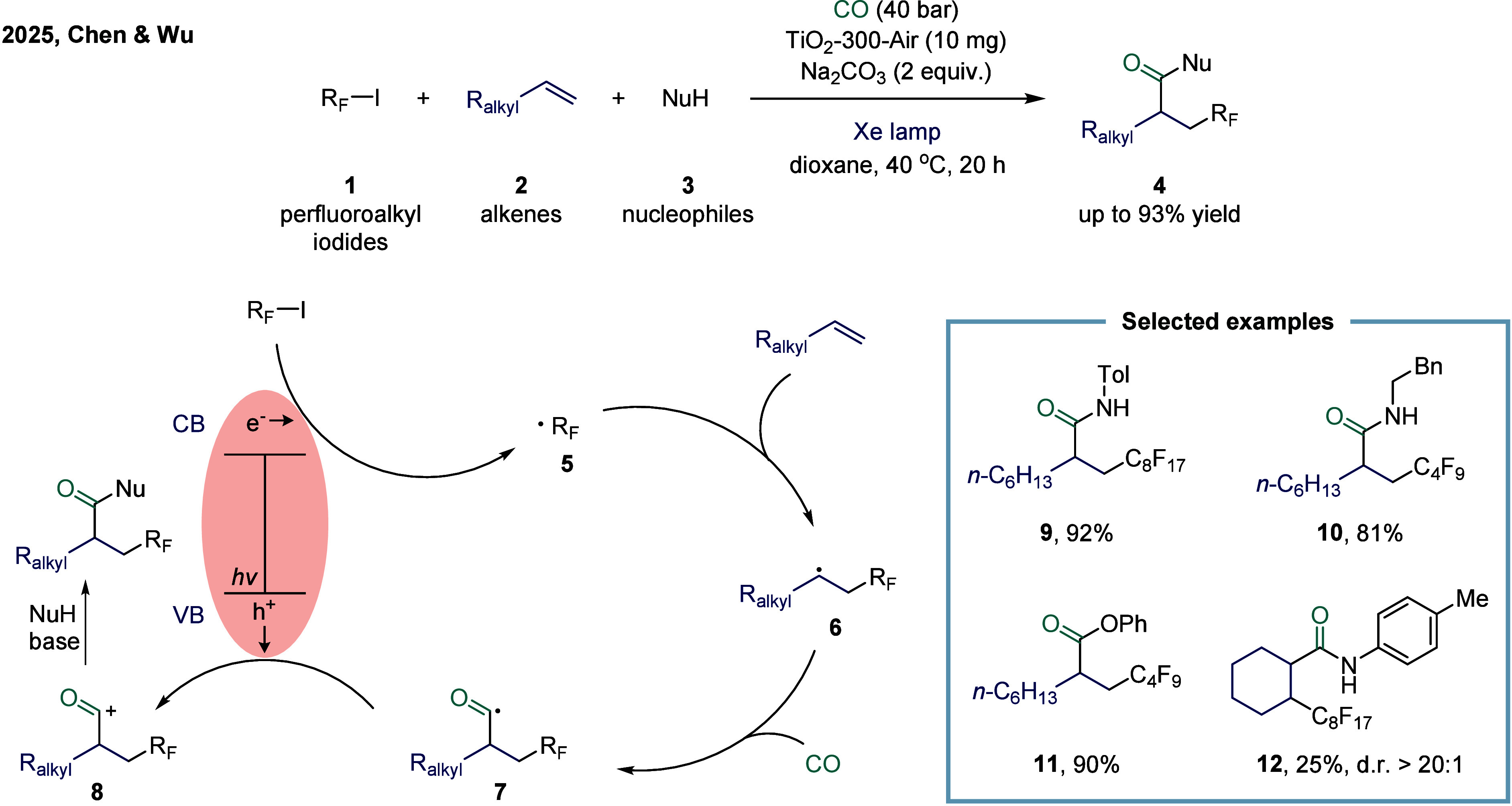
Perfluoroalkyl Carbonylation of Inactive Olefins Catalyzed by Titanium Dioxide

### 2.2 Vanadium-Catalyzed System

Vanadium, an earth-abundant early transition metal, has emerged as a versatile catalyst in a wide range of organic transformations. Its multiple accessible oxidation states (from +2 to +5) enable unique redox properties and the activation of various substrates under mild conditions. The combination of tunable reactivity, relatively low toxicity compared to heavier transition metals, the study of vanadium salt as catalyst is attractive especially from environmental sustainability aspect. In carbonylation reactions, vanadium catalysts are primarily employed for the C–H carbonylation of alkanes to synthesize short-chain carboxylic acids. In 1999, Fujiwara and co-workers reported a vanadium-catalyzed carbonylative transformation of methane (CH_4_) **13** and CO for the synthesis of acetic acid (Scheme 2a).^65^ Although the detailed mechanism has yet to be fully elucidated, it was proposed that a high-valent oxo-vanadium species, V­(V)=O, abstracted a hydrogen radical from methane to generate a methyl radical. This methyl radical then reacted with CO to form an acetyl radical, which was further oxidized by V­(V)=O to an acetyl cation, ultimately leading to the formation of acetic acid. Inspired by the naturally occurring vanadium complex amavadin, Pombeiro and co-workers identified a series of vanadium­(IV)-based catalysts capable of efficiently promoting the carbonylation of methane and ethane (Scheme 2b, 2c).^74,75^

**Scheme 2:**
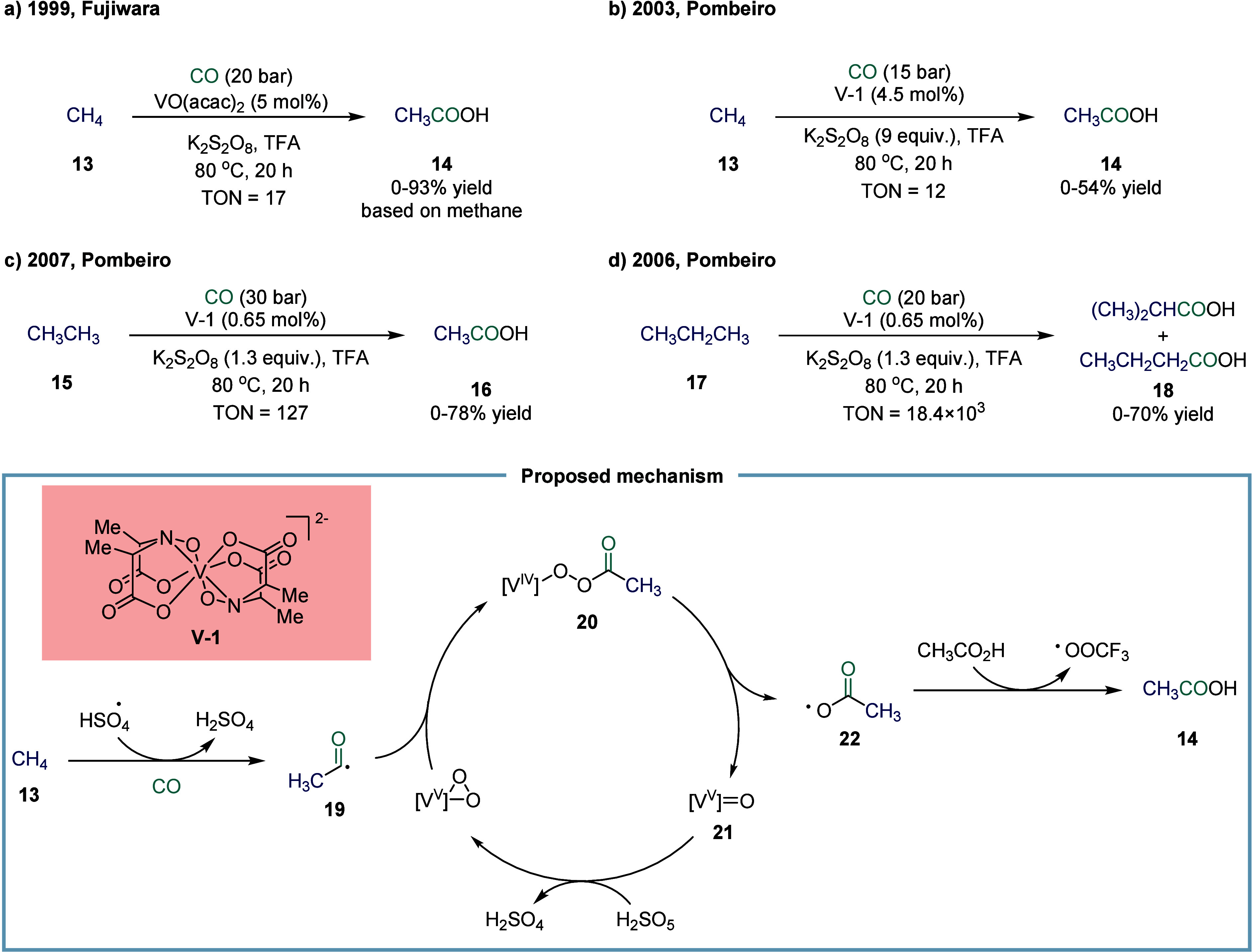
Vanadium-Catalyzed Carbonylation Transformation of C1–C3 Alkanes with CO

In 2006, a vanadium-catalyzed highly efficient direct carbonylation of propane to butyric acids was described by Pombeiro and co-workers (Scheme 2d).^76^ In the presence of potassium persulfate (K_2_S_2_O_8_) and trifluoroacetic acid, propane **17** underwent direct and efficient carboxylation with carbon monoxide, catalyzed by a vanadium-based system, producing predominantly isobutyric acid alongside minor amounts of *n*-butyric acid. The catalytic system comprised [V]-1, which demonstrated superior activity relative to simpler vanadium compounds such as [VO­(acac)_2_] and VOSO_4_. The reaction furnished carboxylic acids in yields of up to 70%, with turnover numbers (TON) as high as 18.4 × 10^3^. Mechanistic studies indicate that the process involves both carbon- and oxygen-centered radical intermediates, with K_2_S_2_O_8_ serving as the oxidant to initiate a radical mechanism. Later in 2007, the same group suggested that these reactions are likely to proceed via the generation of initial acyl radicals from alkanes.^77^

### 2.3 Manganese-Catalyzed System

In recent years, there has been growing interest in the use of manganese as a catalyst in organic synthesis, owing to its earth abundance, low cost, and low toxicity. Since the seminal work by Heck and Breslow in 1963 on alkoxycarbonylation and aminocarbonylation of alkyl iodides catalyzed by [Co­(CO)_4_]^−^ and [Mn­(CO)_5_]^−^ complexes,^78^ the field experienced a prolonged period of inactivity. However, several manganese-catalyzed carbonylative coupling reactions of alkyl iodides have been developed recently, reigniting interest in this area. For example, the formation of esters via the carbonylative coupling of alkyl iodides and alcohols was investigated by Watanabe and co-workers in 1994 using cobalt- and manganese–carbonyl precatalysts.^79^ Subsequently, Coates and co-workers expanded this transformation to the carbonylation of epoxides through a bifunctional catalysis strategy, enabling the efficient synthesis of β-lactones.^80^

#### 2.3.1 Carbon–Halogen Bonds

In SET-mediated carbonylative coupling reactions, manganese catalysts continue to exhibit excellent catalytic activity. In 2017, Mankad and co-workers reported a bimetallic system comprising copper and manganese cocatalysts for the carbonylative coupling of alkyl iodide electrophiles with arylboronic esters, enabling the synthesis of alkyl-aryl ketones (Scheme 3).^81^ Preliminary mechanistic experiments suggested that the process involved codependent catalytic cycles. In one cycle, the Cu-carbene cocatalyst **26** underwent transmetalation with aryl borate esters to generate organocopper nucleophiles **27**. In parallel, the Mn-carbonyl cocatalyst activated alkyl halide electrophiles via SET process, forming alkyl manganese carbonyl species **28**, which subsequently underwent reversible carbonylation to produce acyl manganese electrophiles **29**. Finally, these two catalytic cycles converged in a heterobimetallic C–C bond-forming step that released the ketone product **25**. Among the nucleophilic coupling partners evaluated, pinacol ester **32** demonstrated superior reactivity relative to **33**, whereas the unprotected boronic acid **34** afforded significantly lower reactivity. Moreover, the formation of ketone **25** proceeded with low efficiency when 1-bromooctane **35** or 1-octyltosylate **36** was employed as substrate instead of alkyl iodides. However, the catalytic activity was recovered upon addition of stoichiometric tetrabutylammonium iodide, likely due to the in situ generation of the corresponding alkyl iodides.

**Scheme 3:**
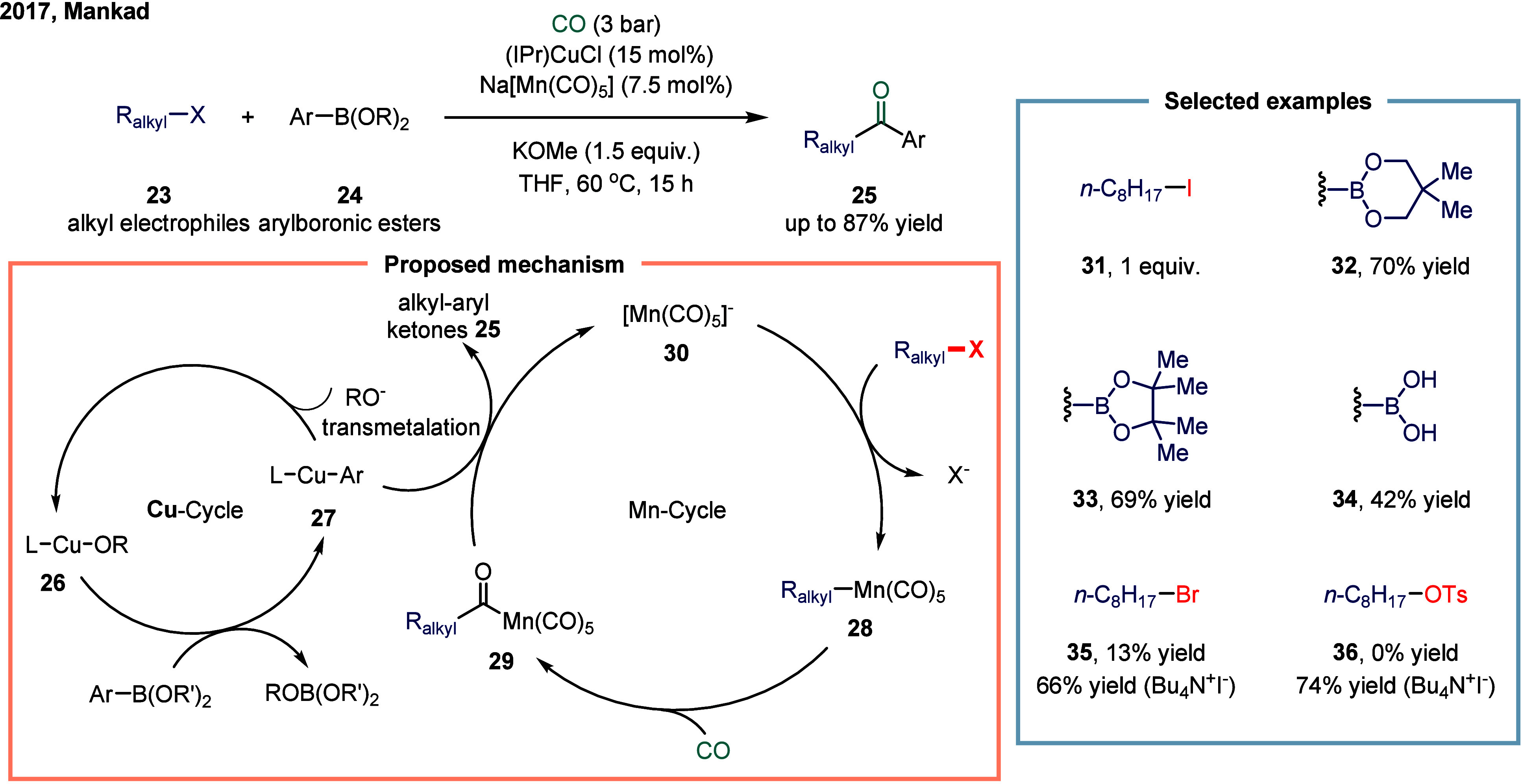
Manganese and Copper Bimetallic Catalysis Enables Carbonylative Suzuki-Miyaura Coupling

In 2024, Wu and co-workers demonstrated a manganese-catalyzed aminocarbonylation of alkyl iodides under visible blue LED light, providing a mild and efficient route to amide products. (Scheme 4).^82^ Mechanistic studies revealed that under blue light irradiation, the Mn–Mn bond in Mn_2_(CO)_10_ underwent homolytic cleavage to generate the carbonyl manganese radical species **40**. This radical abstracted an iodine atom from the alkyl iodide, forming Mn­(CO)_5_I **41** and an alkyl radical. The alkyl radical subsequently underwent carbonylation with Mn­(CO)_5_I **41** to produce the acyl manganese intermediate alkyl-(CO)­Mn­(CO)_4_I **42**. Finally, intermediate **42** reacted with the amine to yield the alkyl amide product, regenerating the catalyst through reductive elimination and closing the catalytic cycle. The method operated under mild reaction conditions and facilitated the efficient synthesis of a diverse range of alkyl amides from aryl amines without the need for additional ligands.

**Scheme 4:**
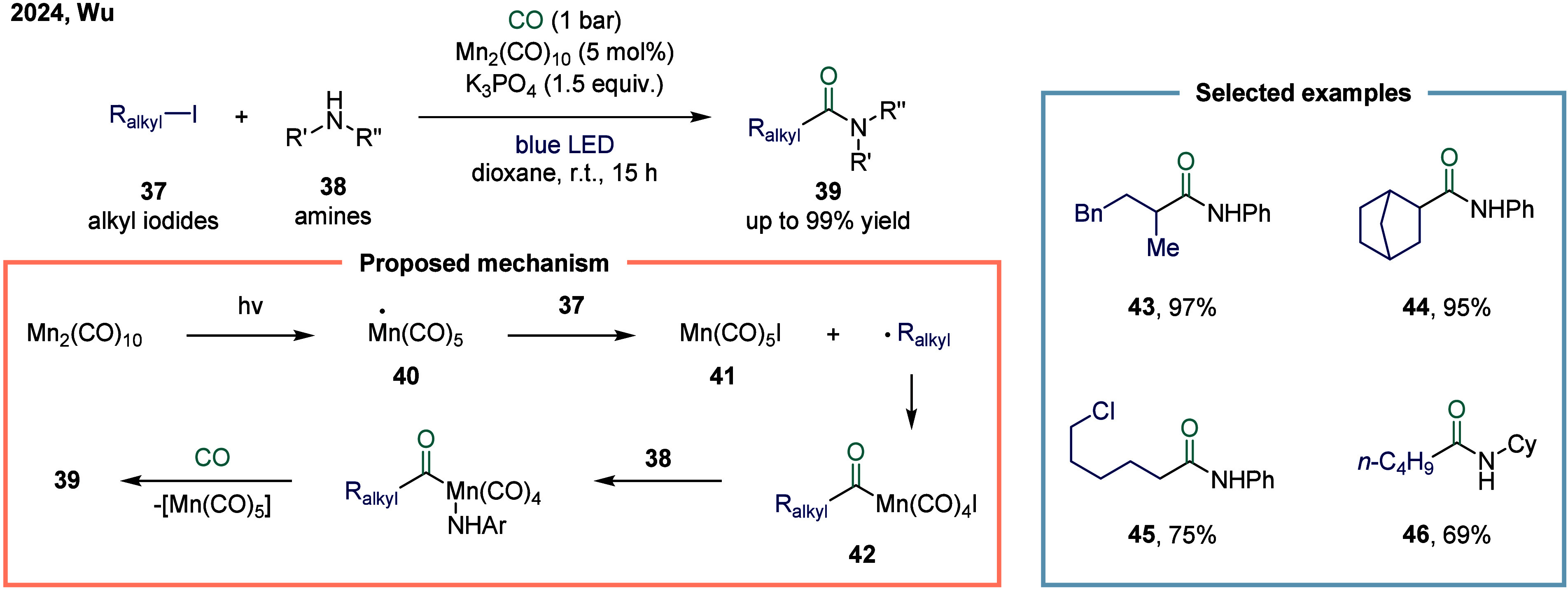
Visible-Light-Induced Manganese-Catalyzed Aminocarbonylation of Alkyl Iodides

#### 2.3.2 Unsaturated Bonds

The Alexanian group reported a manganese-catalyzed carboacylation of alkenes with alkyl iodides (Scheme 5).^83^ The reaction was initiated by the homolysis of the Mn–Mn bond in manganese carbonyl, generating the •Mn­(CO)_5_ radical and triggering the catalytic cycle. The key step involved the carbon-centered radical **50** underwent addition to the alkene, generating a new alkyl radical **51**. At this stage, the resulting radical **48** could undergo carbonylation to provide the final products **49**. This reaction demonstrated a broad substrate scope in both carbocycle and heterocycle synthesis, such as **52**, **53**, and **54**, showing promising levels of diastereocontrol during the carboacylation process. Examples illustrating the successful synthesis of five-, six-, and seven-membered rings were presented. The prevalent functional group compatibility and mild reaction conditions associated with this alkene difunctionalization were anticipated to enable its application in the synthesis of complex molecules.

**Scheme 5:**
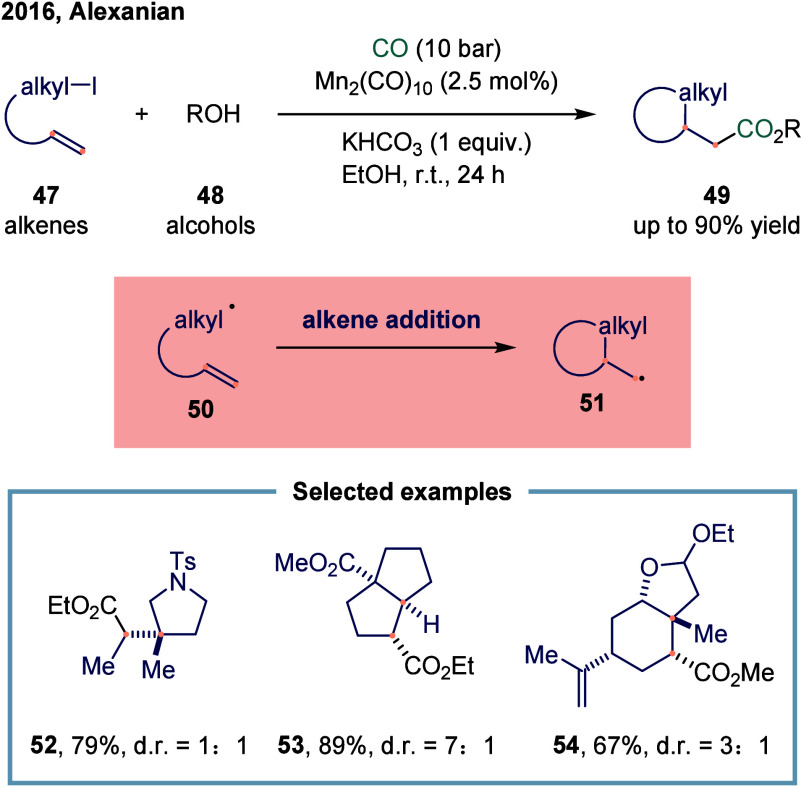
Manganese-Catalyzed Carboacylations of Alkenes with Alkyl Iodides

#### 2.3.3 Carbon–Oxygen Bond

In 2019, Wu and co-workers reported a manganese-catalyzed ring-opening carbonylative transformation of cyclobutanol derivatives through cyclic C–C bond cleavage (Scheme 6).^84^ The reaction proceeded via a radical-mediated pathway to selectively generate 1,5-ketoesters. A variety of substrates bearing substituents on the aromatic ring successfully reacted with linear alcohols of varying chain lengths. Mechanistic studies revealed that the hypervalent iodine reagent (OIDA) oxidized the catalyst and, in the presence of cyclobutanol **55**, facilitated the formation of the Mn­(V) species **58**. Subsequent SET released the cyclobutyloxy radical **59**, which underwent a radical clock-type ring-opening tautomerization to generate the alkyl radical. Under the applied CO pressure, this radical readily captured CO, leading to the formation of the corresponding carbonylated product **57**.

**Scheme 6:**
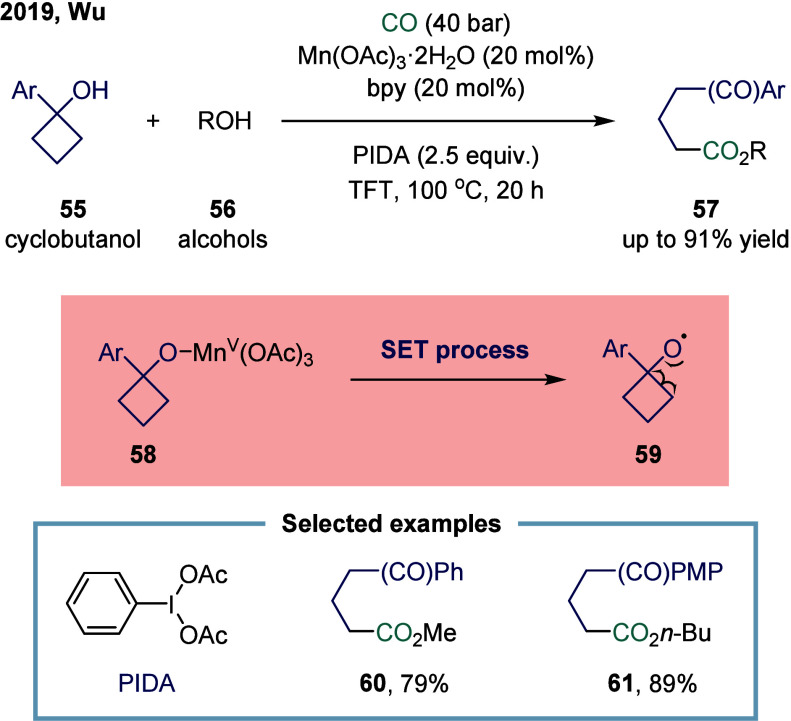
Manganese-Catalyzed Ring-Opening Carbonylation of Cyclobutanol derivatives

#### 2.3.4 Manganese-Promoted System

Potassium alkyltrifluoroborates are readily available and inexpensive starting materials, and Mn­(OAc)_3_·2H_2_O is an nontoxic manganese salt. In 2021, Wu and co-workers reported a manganese-promoted double carbonylation of amines for the synthesis of α-ketoamides (Scheme 7).^85^ Mechanistically, the alkyl trifluoroborate first underwent a SET process mediated by Mn­(III), generating an alkyl radical **64**. This alkyl radical then captured CO to form an acyl radical **65**. In parallel, aniline reacted with Mn­(III) to produce an anilino radical **66**. Subsequently, the anilino radical also captured CO, yielding a carbamoyl radical **67** that was stabilized by the manganese complex. Finally, the resulting acyl radical was quenched by the carbamoyl manganese complex to furnish the α-ketoamide product **63**. However, the authors noted that they could not completely exclude an alternative pathway involving α-ketoacyl radicals and amino radicals. A broad range of alkyl α-ketoamide derivatives (**68**, **69**, and **70**) were synthesized in moderate to good yields with excellent selectivity. In addition to alkyl trifluoroborates, Hantzsch esters **71** could also successfully undergo this double carbonylation transformation, delivering a variety of α-ketoamide derivatives in good yields.

**Scheme 7:**
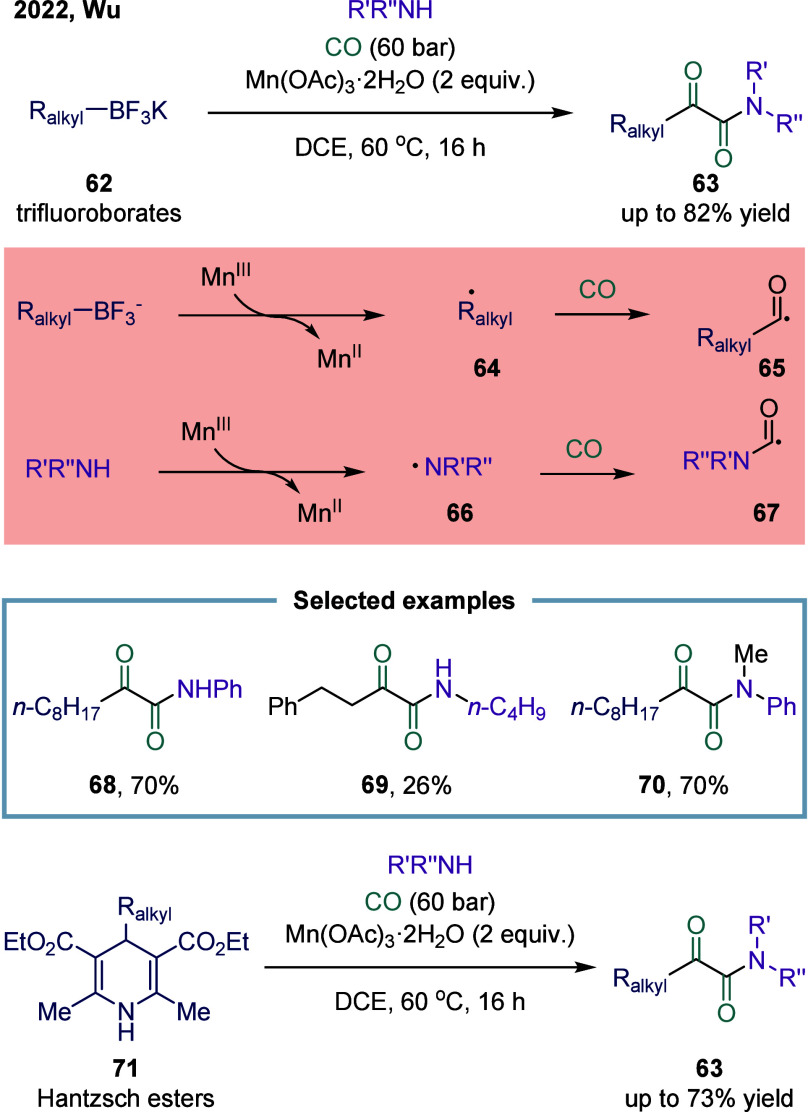
Manganese-Promoted Double Carbonylation of Anilines toward *α*-Ketoamides Synthesis

### 2.4 Iron-Catalyzed System

Iron is one of the most abundant elements on Earth, constituting approximately 4.75% of the Earth’s crust and ranking as the fourth most prevalent element after oxygen, silicon, and aluminum. Although iron can exist in a wide range of oxidation states from −2 to +6, the +2 and +3 states are the most commonly encountered and studied. Of particular interest are low-valent iron complexes, which have demonstrated remarkable catalytic capabilities across diverse chemical transformations.^86–89^ Since the mid-20th century, iron-promoted carbonylation reactions have garnered significant attention. Among these, sodium tetracarbonylferrate (Na_2_Fe­(CO)_4_), commonly known as the Collman reagent, has been widely employed as a stoichiometric reagent in carbonylation processes.^90^ Building upon this foundation, numerous stoichiometric iron-mediated carbonylation reactions have been developed, greatly expanding the synthetic utility and versatility of iron carbonyl complexes.^91–96^ A major breakthrough occurred in 2009 when Beller and co-workers reported the first catalytic carbonylation protocol utilizing iron as the catalyst, marking a pivotal shift from stoichiometric to catalytic approaches.^97^ Since then, iron-catalyzed carbonylation via two-electron transfer pathways has undergone extensive development,^98^ especially in carbonylative cyclization^99,100^ and aminocarbonylation of alkynes.^101,102^ More recently, attention has shifted toward alternative mechanistic paradigms. In contrast to the well-established two-electron transfer pathways, emerging studies have highlighted the growing significance of iron-catalyzed carbonylation reactions proceeding via SET mechanisms. These SET-mediated processes offer distinct reactivity patterns and selectivity profiles, opening new avenues for reaction design and broadening the scope of iron-catalyzed carbonylation chemistry.

#### 2.4.1 Carbon–Hydrogen Bonds

In 2019, Wu and co-workers reported an iron-catalyzed carbonylation for the synthesis of lactams (Scheme 8).^103^ This transformation was particularly noteworthy for its use of SET processes to access reactive radical intermediates under mild conditions. Mechanistic investigations revealed that the iron catalyst promoted a SET reduction of readily available oxime esters, generating the corresponding iminyl radical species **74**. This radical then underwent an intramolecular 1,5-HAT, leading to the formation of a thermodynamically favored tertiary carbon-centered **75**. Subsequent carbonylation and cyclization steps furnished the desired lactam scaffolds, highlighting the power of radical cascade processes in complex molecule construction. A broad range of oxime esters, easily synthesized from simple ketone precursors, were successfully transformed into structurally diverse lactams in moderate yields, demonstrating good functional group tolerance. Notably, the cyclopentane-derived oxime ester provided the corresponding tetra-substituted lactam **76** as the major product in 54% yield.

**Scheme 8:**
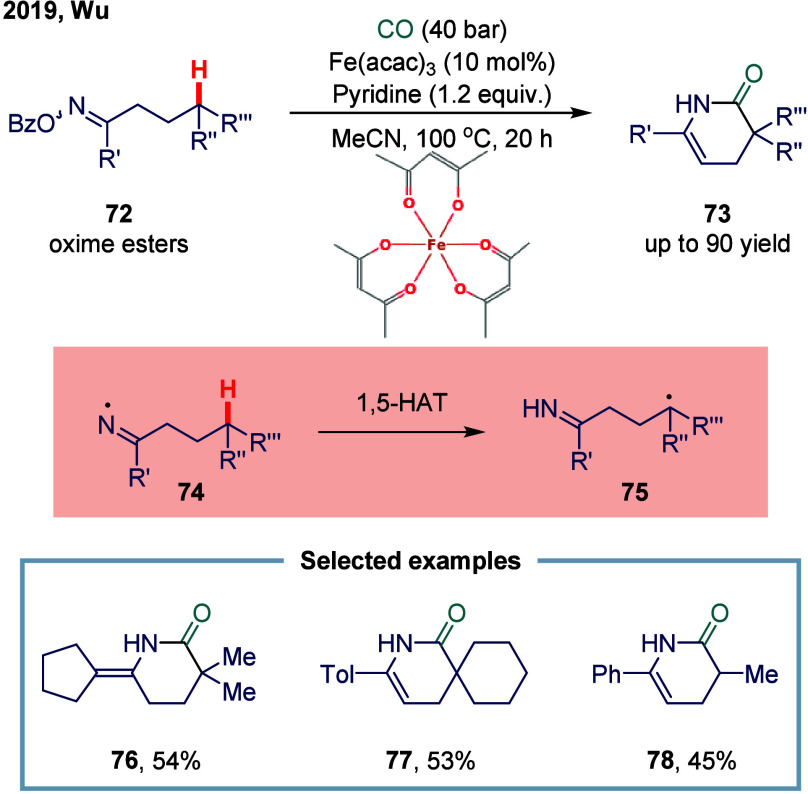
Iron-Catalyzed Carbonylation of Tertiary Carbon Radical via 1,5-HAT Process

Despite the prevalence of C­(sp^3^)-hybridized carbon atoms in organic molecules, developing efficient methods for their direct C–H functionalization has remained a significant challenge. This difficulty is especially pronounced for light alkanes, which possess some of the strongest C–H bonds known. Their activation typically requires harsh conditions that are often incompatible with complex or sensitive substrates. In 2023, Noël and co-workers tackled this challenge by devising a general, mild, and scalable protocol for the direct C­(sp^3^)-H carbonylation of saturated hydrocarbons (Scheme 9).^104^ Their strategy employed a photocatalytic hydrogen atom transfer (HAT) approach under a carbon monoxide atmosphere, enabling the functionalization of both light and heavy hydrocarbons. A key feature of this method was the use of flow technology, which greatly enhanced gas–liquid mass transfer and accelerated reaction kinetics. These improvements were crucial in minimizing side reactions while ensuring scalability and operational safety. Mechanistically, upon light irradiation, the iron species was photoexcited to form the active iron catalyst. This catalyst underwent a ligand-to-metal charge transfer (LMCT) process to generate highly reactive chlorine radicals. These chlorine radicals then initiated the HAT process by abstracting a hydrogen atom from methane, producing methyl radicals that subsequently underwent carbonylation to afford the desired carbonylated products **81**. Nonetheless, due to the exceptionally high bond dissociation energy of methane’s C–H bonds, the conversion efficiency remained modest. As a result, carbonylation products **82** and **83** were obtained in moderate yields of 31% and 33%, respectively. Despite these limitations, this work represents a significant advance in the direct functionalization of challenging C­(sp^3^)-H bonds under mild and scalable conditions.

**Scheme 9:**
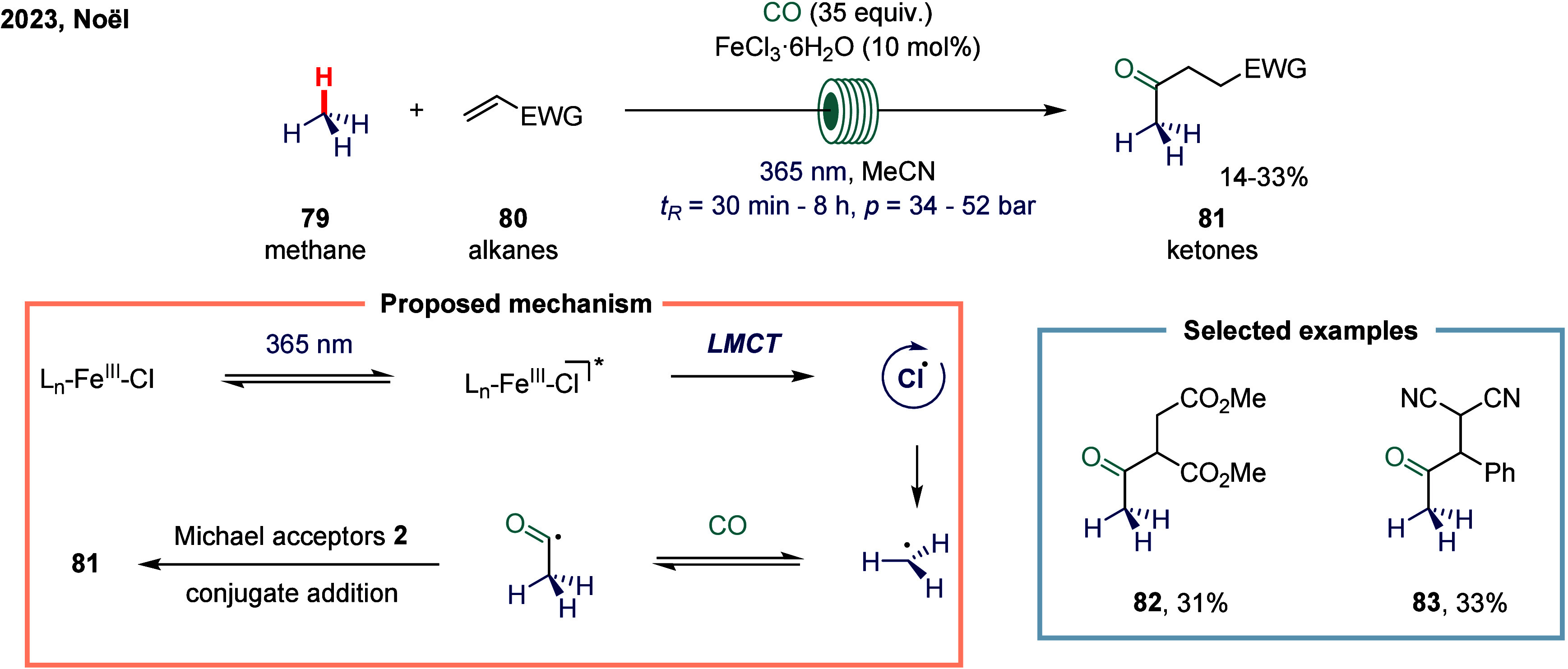
Iron-Catalyzed Photo-Induced Methane Carbonylation in Flow

The aerobic carbonylation of methane to acetic acid remained a particularly challenging transformation, primarily due to the rapid oxidation of methyl radicals that led to undesired C1 oxygenates. In 2025, Zuo and co-workers introduced a pioneering iron terpyridine photocatalyst that leveraged LMCT to precisely balance methyl radical generation and capture, thereby achieving exceptional C2/C1 selectivity under mild, aerobic conditions (Scheme 10).^105^ This catalyst operated through a unique combination of one- and two-electron redox steps: upon photoexcitation, the Fe­(III) species underwent LMCT to generate alkoxy radical **85**, which abstracted hydrogen atoms from methane via HAT process, producing methyl radical. Concurrently, the resulting Fe­(II) species **86** coordinated CO, facilitating efficient methyl radical carbonylation through a radical rebound-like mechanism. The formation of iron species **84** was a pivotal step in this mechanism, bridging radical generation and carbonylation. Extensive mechanistic studies, including isotope labeling and isolation of iron alkoxide complexes, underscored the crucial role of iron–carbonyl intermediates in preventing unwanted methyl radical oxidation, a major obstacle in aerobic methane functionalization. Remarkably, this iron terpyridine catalyst achieved turnover numbers (TON) as high as 61,300 and selectivity ratios up to 26:1 (C2/C1), outperforming many precious metal catalysts that often required harsher conditions and exhibited lower selectivity.

**Scheme 10:**
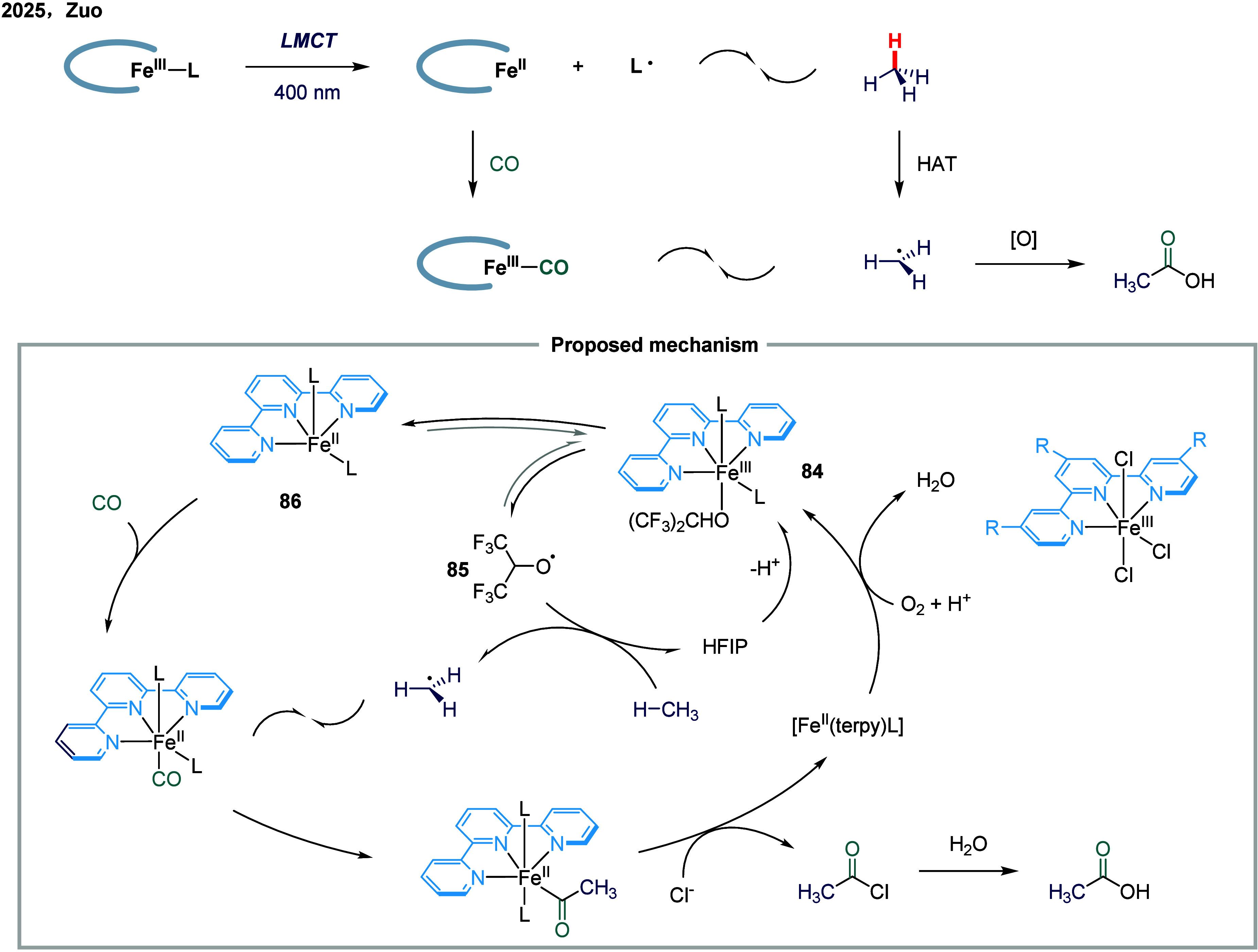
Iron-Catalyzed Aerobic Carbonylation of Methane via Ligand-to-Metal Charge Transfer Excitation

#### 2.4.2 Carbon–Halogen Bonds

Carbonylative Suzuki reactions have traditionally relied on noble metals such as palladium, which are limited by their scarcity, high cost, and significant toxicity.^106,107^ Compared with palladium, iron-catalyzed carbonylative Suzuki reactions offer a mild and economical alternative for the synthesis of diaryl ketones. The first examples of iron-catalyzed carbonylation of aryl iodides, employing Co_2_(CO)_8_ as a cocatalyst to access symmetrical biaryl ketones, were reported by Brunet and co-workers.^108^ However, these pioneering studies suffered from several major limitations, including a severely restricted substrate scope, low chemoselectivity, and poor functional group tolerance, which collectively limited their practical utility in organic synthesis. In 2014, Han and co-workers reported an iron-catalyzed carbonylative Suzuki reaction under an atmospheric pressure of carbon monoxide (Scheme 11).^109^ Mechanistic studies revealed that the key organoiron species **90** was generated from the highly nucleophilic organoiron complex via intramolecular CO migratory insertion. The resulting organoiron intermediate **90** then reacted with aryl iodides through an S_N_R1-type nucleophilic oxidative addition to furnish organoiron complex **91**. This protocol enabled the efficient synthesis of a broad array of unsymmetrical biaryl ketones, such as compounds **92**, **93**, and **94**, in high yields and with excellent chemoselectivity.

**Scheme 11:**
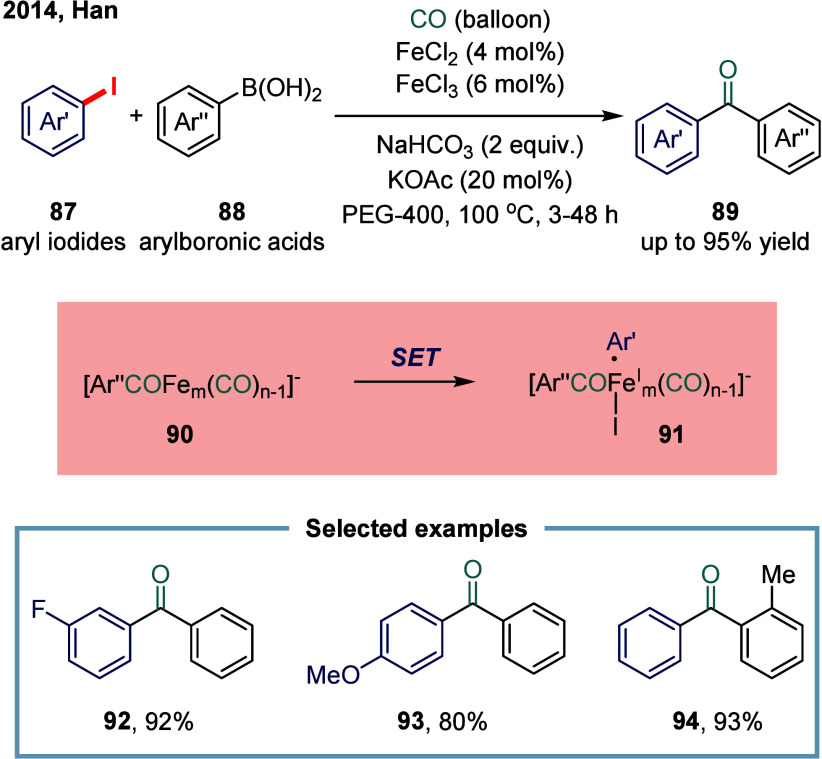
Iron-Catalyzed Carbonylative Suzuki Reactions of Aryl Iodides and Arylboronic Acids

Subsequently, Han and co-workers developed an iron-catalyzed carbonylative Suzuki–Miyaura coupling of aryl halides with arylboron reagents, employing stoichiometric chloroform as the carbon monoxide source (Scheme 12).^110^ Chloroform has attracted considerable attention as a widely available, stable, and inexpensive alternative to CO, and has been successfully utilized in carboxylation and carbonylative Sonogashira couplings.^111–113^ The *in situ* release of CO is achieved via hydrolysis of CHCl_3_ in the presence of strongly basic hydroxides.^114,115^ Notably, this strategy enables efficient ^13^C labeling simply by using commercially available ^13^C-labeled chloroform, for example, compounds **96**.

**Scheme 12:**
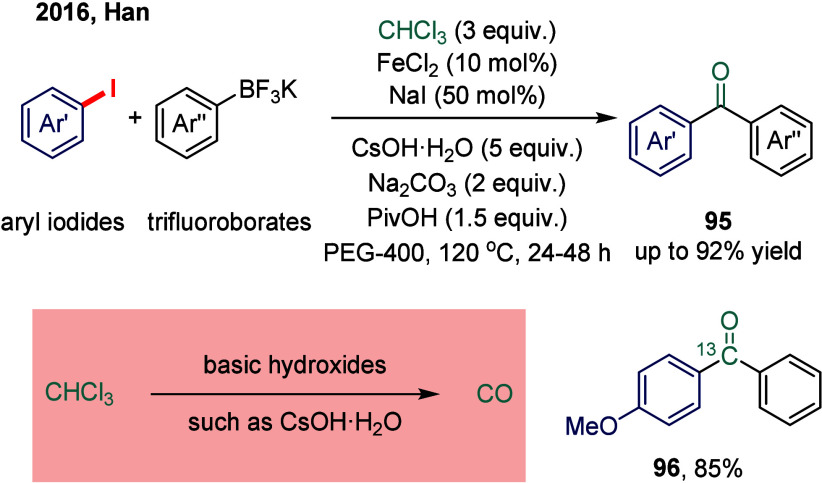
Iron-Catalyzed Carbonylative Suzuki Reactions of Aryl Iodides and Trifluoroborates Using Stoichiometric Chloroform as the CO Source

Transition metal-catalyzed carbonylative cross-coupling reactions of alkyl halides are among the most widely employed strategies for the synthesis of aliphatic carboxylic acid derivatives. In recent years, a variety of carbonylative protocols involving alkyl halides have been developed, particularly those utilizing palladium and copper catalysts. Despite these advances, iron-catalyzed carbonylative cross-coupling remains highly attractive to the sustainability-oriented chemical community, owing to iron’s low cost, earth abundance, and its potential to exhibit unique and complementary reactivity profiles. In 2022, Wu and co-workers reported an iron-catalyzed alkoxycarbonylation of alkyl bromides and iodides, enabling the synthesis of a wide range of esters (Scheme 13a).^116^ Notably, the reaction conditions required no reoptimization to accommodate diverse electrophiles, including alkyl iodides **99**, **100**, and **101**, tosylates **105**, and even mesylates **106**, all of which provided the corresponding esters in good yields. Moreover, unactivated secondary alkyl bromide **103** was also tolerated, albeit affording the ester in somewhat diminished yield. At the current state of the art, alkyl chloride **104** remains unreactive under these conditions. Mechanistic investigations revealed that the low-valent iron species activated alkyl bromides via a distinctive two-electron transfer (TET) pathway, while alkyl iodides underwent activation through a single-electron transfer (SET) mechanism-consistent with earlier mechanistic precedents. To further probe these divergent activation modes, Wu and colleagues examined iron-catalyzed aminocarbonylation in 2023 (Scheme 13b).^117^ A radical clock experiment employing 6-iodohex-1-ene delivered the cyclized product **108** in 76% yield, whereas the corresponding 6-bromohex-1-ene predominantly afforded the linear amide **107**. These results provided compelling evidence supporting the operation of an SET pathway in the carbonylation of alkyl iodides. This protocol offered a practical and efficient route to a diverse array of amides, imides, and *N*-acylindoles derived from amines, amides, and indoles. Mechanistically, the catalytically active iron species was generated in situ under a CO atmosphere in the presence of base. This complex reacted with alkyl iodides via SET to generate an alkyl radical, which was subsequently captured by the iron center to form the alkyliron intermediate **109**. Carbonylation of this intermediate furnished the desired products. In contrast, for alkyl bromides, an intermediate arose via a TET pathway. Notably, the final acyliron species could also be accessed through capture of an in situ formed acyl radical.

**Scheme 13:**
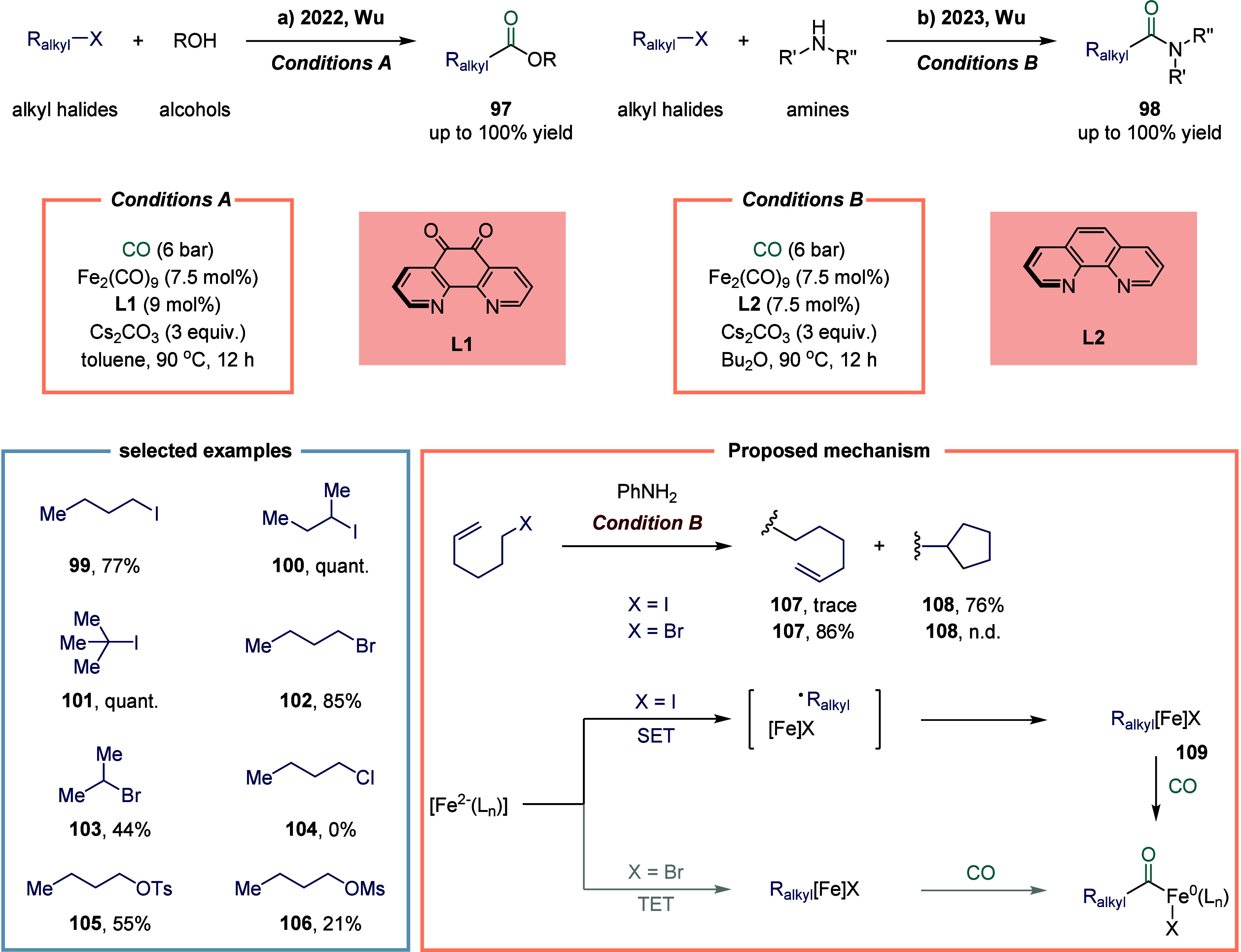
Iron-Catalyzed Alkoxycarbonylation and Aminocarbonylation of Alkyl Bromides or Alkyl Iodides

Thioesters constitute a fundamental class of compounds that are widely encountered in pharmaceuticals, natural products, bioactive molecules, and even in food chemistry.^118,119^ In 2024, Wu and co-workers reported an iron-catalyzed carbonylative coupling of alkyl iodides for the synthesis of tert-alkyl thioesters (Scheme 14).^120^ In this reaction, various sterically hindered alkyl thioesters were synthesized from unactivated iodides and *S*-aryl thioesters. The effects of different S-aryl thioester precursors on the reaction outcome were systematically evaluated. A range of primary **110-a**, secondary **110-b**, and aromatic **110-c** thioesters were successfully employed as sulfur sources, affording the target products in yields exceeding 80%. However, no desired product was obtained when substrate **110-d** was tested under the standard conditions. In addition to sterically hindered tertiary alkyl iodides, unactivated secondary alkyl iodides also provided the corresponding products **114** in good yields.

**Scheme 14:**
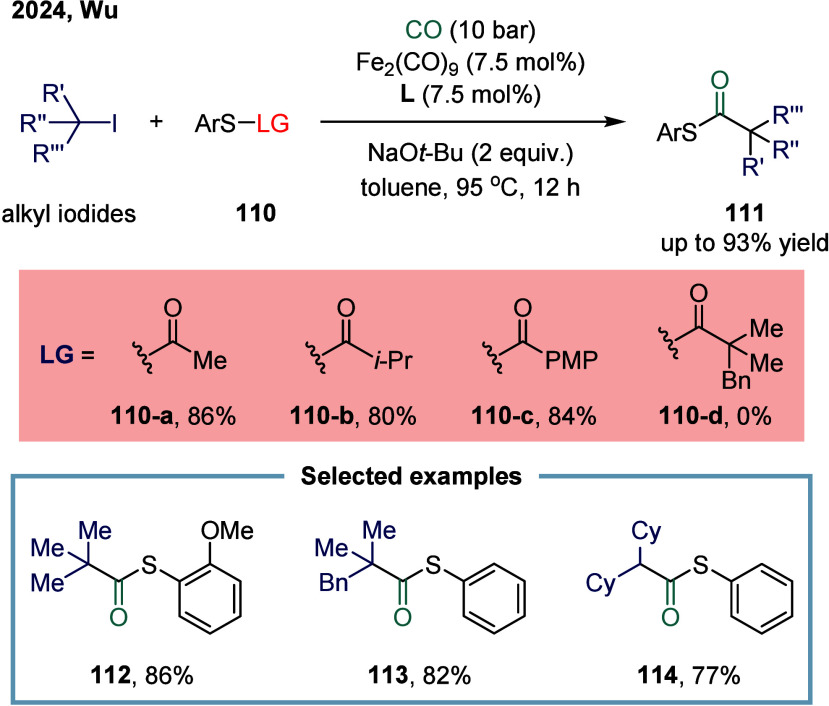
Iron-Catalyzed Carbonylative Synthesis of *tert*-Alkyl Thioesters

Although iron is an ideal metal for carbonylation reactions owing to its high natural abundance and low cost, the strong affinity of CO for iron often results in the formation of saturated iron carbonyl complexes that inhibit catalytic activity. The combination of iron and copper catalysts offers several potential advantages. Beyond sustainability and economic benefits, the cooperative interactions between copper and iron can facilitate the activation of added or *in situ* generated iron carbonyl complexes and stabilize reactive carbonyl-metal intermediates.

Motivated by this state-of-the-art approach, Wu and co-workers developed a copper/iron cocatalyzed alkoxycarbonylation of unactivated alkyl bromides (Scheme 15).^121^ In the presence of catalytic quantities of both iron and copper catalysts, a variety of primary and secondary alkyl bromides, such as **116** and **117**, were efficiently converted to the corresponding aliphatic esters in good yields. A proposed single-electron transfer (SET) mechanism involved the initial, irreversible abstraction of bromine from alkyl bromides 1 by the copper species, generating a carbon-centered radical and a copper complex **118**. Fu and co-workers have also demonstrated a similar species in a photochemical protocol.^122^ The resulting copper complex **118** then reacted with the iron species, leading to the formation of an acyl-iron complex **119** through transmetalation and subsequent CO insertion steps.

**Scheme 15:**
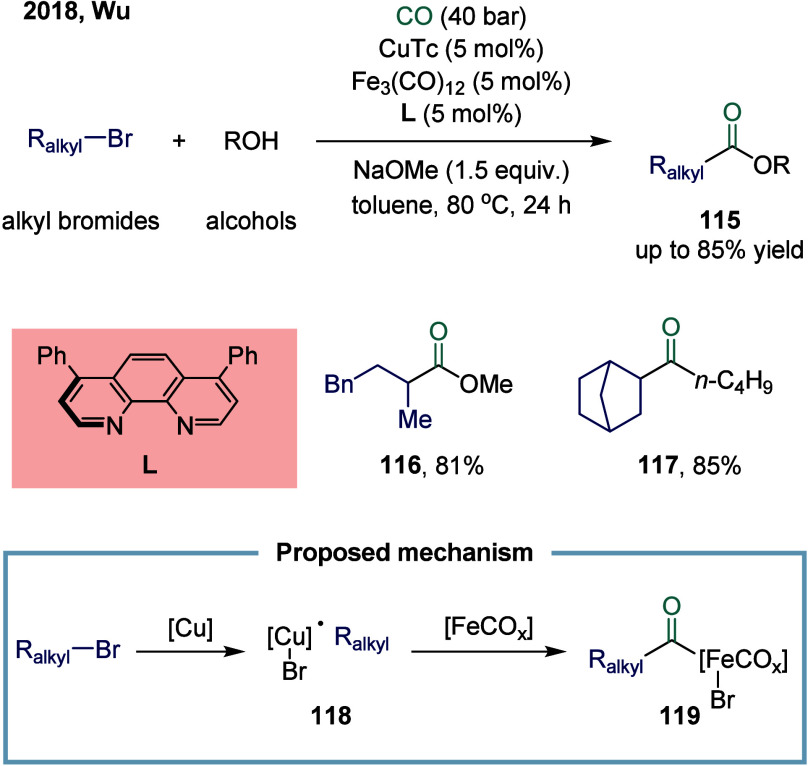
Copper/Iron Co-Catalyzed Alkoxycarbonylation of Unactivated Alkyl Bromides

#### 2.4.3 Unsaturated Bonds

In 2020, Wu and co-workers developed an iron-catalyzed carbonylative cyclization of *γ,δ*-unsaturated aromatic oxime esters with amines for the synthesis of β-homoproline amide derivatives (Scheme 16).^123^ In this reaction, a variety of β-homoproline amides and their derivatives were prepared with excellent functional group tolerance via an iminyl radical-mediated intramolecular 1,5-cyclization followed by an intermolecular carbonylation triggered by a carbon radical. Unsubstituted, monosubstituted, and disubstituted substrates **121**, **122**, and **123** could all participate in this transformation smoothly. However, the reaction exhibited strict requirements regarding the length of the olefinic carbon chain. For example, substrates **125** and **126** did not react efficiently, primarily due to limitations in the cyclization step.

**Scheme 16:**
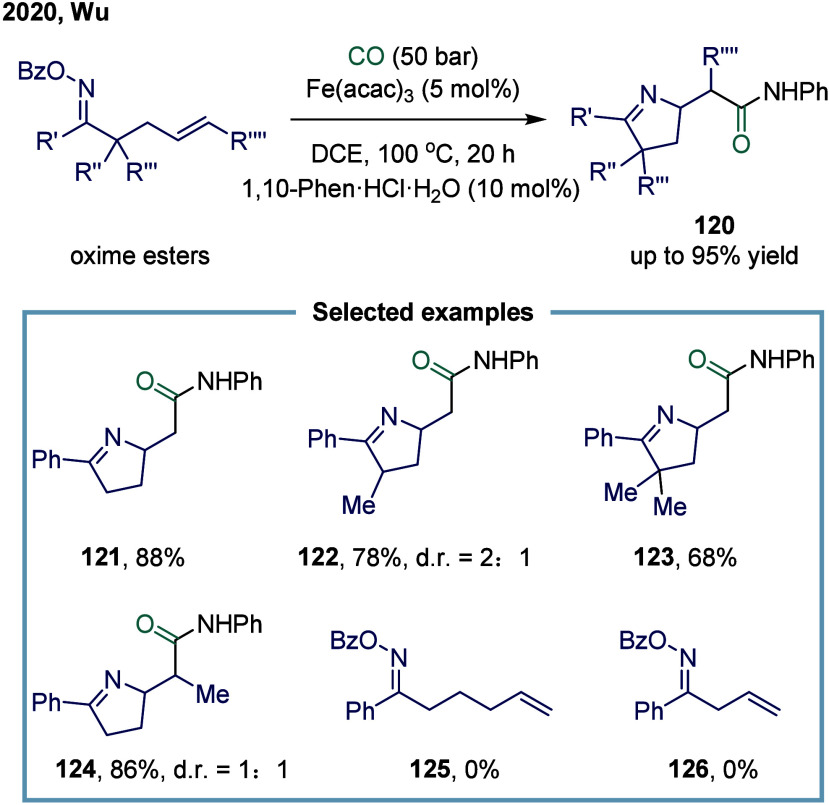
Iron-Catalyzed Carbonylative Cyclization of Unsaturated Aromatic Oxime Esters with Amines

### 2.5 Cobalt-Catalyzed System

Cobalt commonly exhibits +2 and +3 oxidation states, although oxidation states ranging from −3 to +5 have also been reported. A notable feature of cobalt complexes is their strong affinity for π-bonded systems. The application of this metal in the carbonylation coupling reactions is a relatively well-established field. The earliest application of cobalt in carbonylation was described by BASF in 1960. It is widely accepted that cobalt precursors react with carbon monoxide and a proton source to generate the catalytically active species [HCo­(CO)_4_]. In 1961, Heck and Breslow conducted a systematic study on the reaction of cobalt hydrotetracarbonyl with olefins.^124^ Despite its long and well-established history, cobalt-catalyzed carbonylation still faces several challenges. First, carbon monoxide coordinates strongly to cobalt, often leading to the formation of stable but catalytically inactive cobalt carbonyl complexes. Second, the catalytic activity of cobalt is relatively insensitive to ligand modification, which limits opportunities for fine-tuning reactivity and selectivity. Cobalt exhibits excellent catalytic performance in single-electron transfer (SET) processes due to its unique electronic structure and multivalent oxidation states. Cobalt catalysts facilitate the generation, transfer, and carbonyl insertion of single-electron species. Specifically, cobalt catalysts typically activate substrates such as halogenated hydrocarbons or olefins via a SET mechanism to generate radical intermediates. Additionally, cobalt stabilizes acyl radical intermediates and promotes their subsequent transformations, including nucleophilic attack, to afford various carbonyl-containing products.

#### 2.5.1 Carbon–Hydrogen Bonds

In sharp contrast to the well-developed metal-catalyzed C­(sp^3^)-H carbonylation of alkanes, the carbonylation of C­(sp^3^)-H bonds in α-heteroatom-substituted alkanes remains significantly more challenging, primarily due to hyperconjugative and polar effects. Nevertheless, Wu and co-workers achieved an elegant cobalt-catalyzed direct aminocarbonylation of ethers, forming various α-oxy amides (Scheme 17a).^125^ A variety of cyclic and linear ethers proved efficient in this carbonylation coupling, delivering the corresponding products in up to 93% yields. Further mechanistic studies revealed that, in the presence of a peroxide, a carbon-centered radical **128** adjacent to the ether oxygen was generated and subsequently captured by Co­(II), forming a Co­(III) intermediate **129**. This intermediate underwent ligand exchange with an amine, followed by coordination and insertion of CO to yield the acyl cobalt species **131**, which then underwent reductive elimination to furnish the desired product **127**. Alternatively, radical **128** may directly trap CO to generate an acyl radical **130**, which coordinates with Co­(II), underwent ligand exchange, and proceeds through a similar pathway to afford product **127**. A wide range of α-oxy amides has been efficiently synthesized using cobalt catalysis, demonstrating excellent functional group tolerance. When asymmetric ethers (**133**-**136**) containing two or more oxygen α-C­(sp^3^)-H bonds in distinct environments were employed, carbonylation occurred with high regioselectivity at a specific site. This regioselectivity is likely governed by a combination of steric hindrance and radical polarity effects. Notably, this method has also been successfully applied to the synthesis of the drug molecule Alfuzosin in a concise two-step sequence. Next, Wu and co-workers successfully applied this catalytic system to achieve C­(sp^3^)-H alkoxycarbonylation reactions (Scheme 17b).^126^ Remarkably, the method proved effective not only for simple alkanes and ethers but also for more complex substrates such as crown ethers **138**, demonstrating broad substrate compatibility. This work highlights the versatility of the catalytic system in functionalizing otherwise inert C­(sp^3^)-H bonds under mild conditions.

**Scheme 17:**
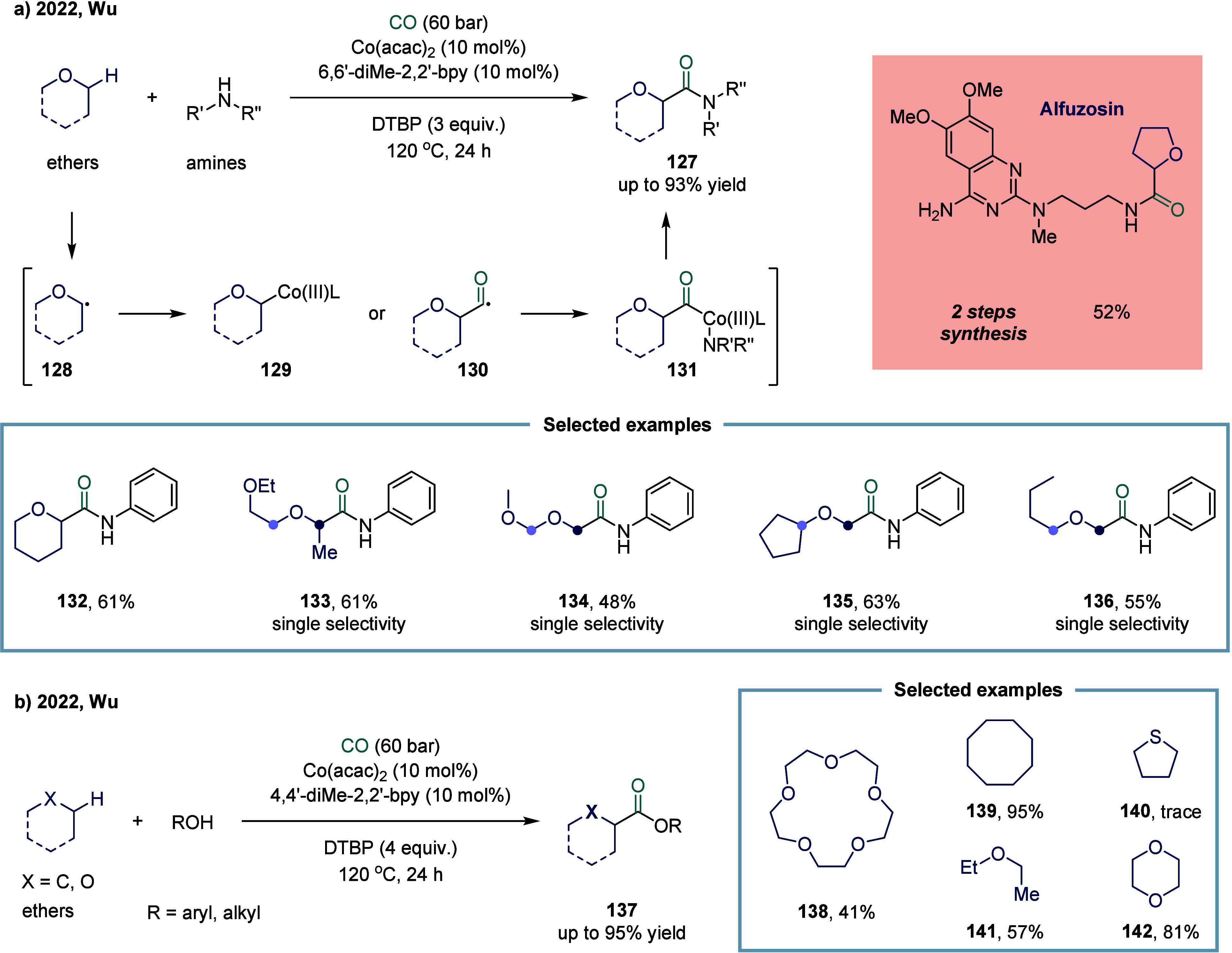
Cobalt-Catalyzed Direct Aminocarbonylation and Alkoxycarbonylation of Ethers

In 2022, Lei and co-workers reported that the oxidative mono- or double-carbonylation of alkanes with CO could be selectively tuned by choosing either a cobalt or copper catalyst (Scheme 18a).^127^ Notably, the cobalt catalytic system exclusively afforded monocarbonylation products. This study underscored the critical role of the metal center in modulating both the reaction pathway and selectivity in alkane carbonylation chemistry. Using Co­(acac)_2_ and 2,9-dimethyl-1,10-phenanthroline as the catalytic system, a variety of alkanes (primary, secondary, and cyclic alkanes) and amines were efficiently converted into the corresponding monocarbonylated amides **143** up to 99% yield under relatively mild conditions. Particular emphasis was placed on the broad compatibility of amines, including aliphatic, aromatic, primary, secondary, and heterocyclic derivatives, demonstrating excellent functional group tolerance. In contrast, the scope of alkanes was largely confined to cycloalkanes, as linear alkanes posed challenges due to limited site selectivity.

**Scheme 18:**
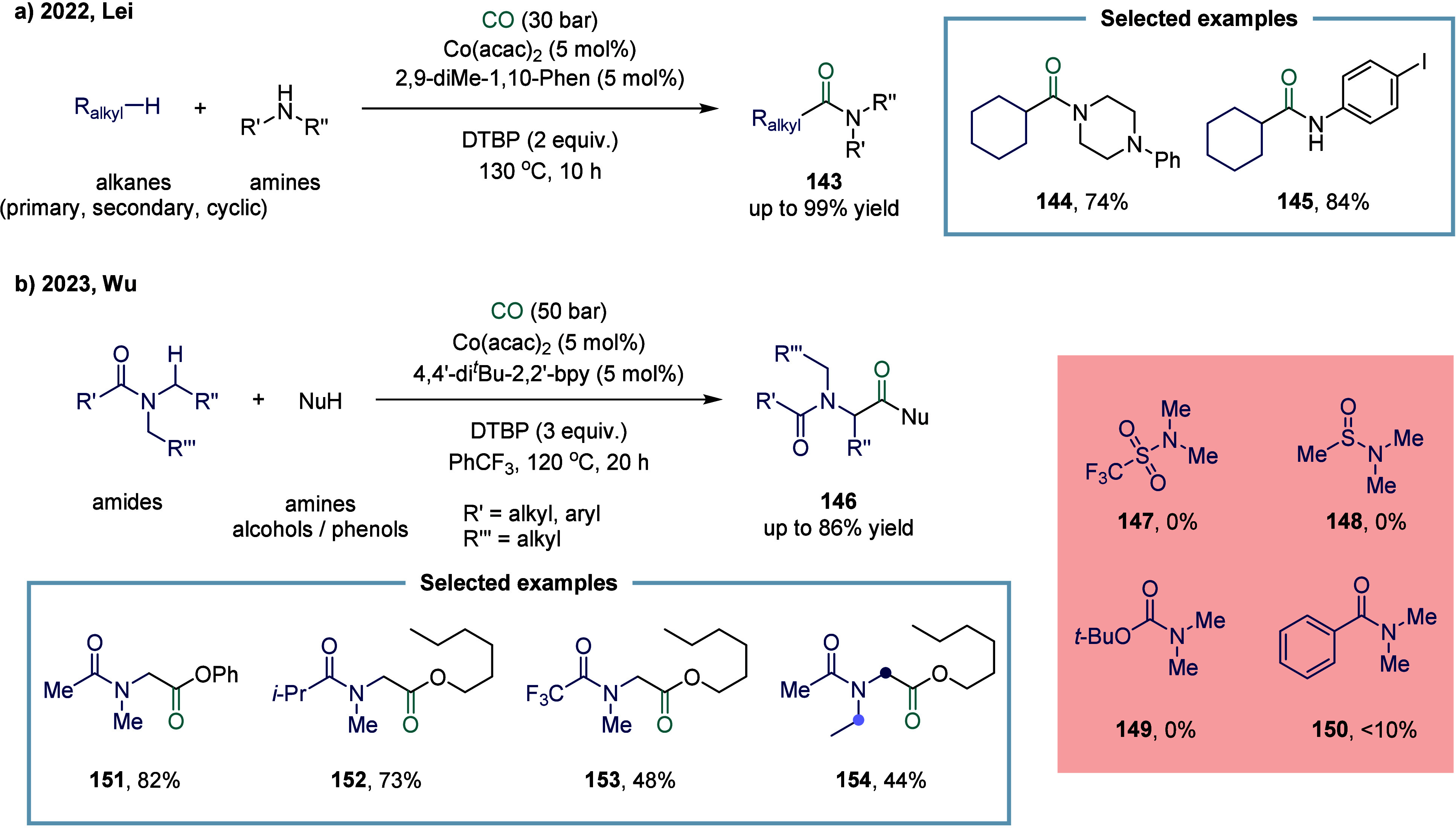
Cobalt-Catalyzed Direct Carbonylation of Alkanes or Amides

α-amino acid derivatives, which constitute the structural foundation of peptides and proteins, represent some of the most important amino acids.^128^ Notably, 22 α-amino acids are encoded by the genetic code.^129^ Moreover, as the simplest class of amino acids, α-amino acids are extensively employed in the biosynthesis of diverse peptides and proteins in living organisms. In 2023, Wu and co-workers reported a cobalt-catalyzed carbonylation of α-aminoalkyl radicals for the synthesis of α-amino acid derivatives (Scheme 18b).^130^ The key to the success of this model lay in introducing an appropriate electron-withdrawing group to modulate the nucleophilicity of the α-aminoalkyl radical, thereby providing a suitable polarity match for the subsequent carbonylation reaction. This catalytic strategy exhibited broad substrate applicability and excellent tolerance toward sensitive functional groups, offering a general and practical approach for the synthesis of α-amino acid derivatives, such as compounds **151**, **152**, **153**, and **154**. A variety of electron-withdrawing groups, including sulfonyl **147**, sulfinyl **148**, and *tert*-butoxycarbonyl **149**, were evaluated; however, the carbonylative reaction did not proceed in the presence of any of these groups. Benzoyl group **150** afforded yield below 10%, whereas alkyl acyl groups were found to be the most suitable for this reaction. Therefore, the *N*-acyl group played a pivotal role in the success of this α-aminoalkyl carbonylation.

#### 2.5.2 Carbon–Halogen Bonds

Since the seminal work by Foa and co-workers in 1985, which described the cobalt-catalyzed carbonylation of aromatic and heteroaromatic halides into (hetero)­aryl formate esters using anionic cobalt complexes, the carbonylative coupling of C­(sp^2^)-X bonds catalyzed by cobalt has witnessed significant development.^131^ Over the following decades, an increasing number of reaction modes have been explored. In 2020, Alexanian and co-workers reported a cobalt-catalyzed, visible-light-promoted aminocarbonylation of (hetero)­aryl halides (Scheme 19).^132^ This transformation employed a simple cobalt catalyst under visible light irradiation, enabling the efficient synthesis of a variety of amides. Mechanistically, the process began with the disproportionation of octacarbonyldicobalt upon coordination with either the amine or TMP (2,2,6,6-tetramethylpiperidine), generating a cobaltate anion **156**.^133,134^ Coordination of this active cobaltate species to the (hetero)­aryl or vinyl electrophile formed a donor–acceptor complex **157**, which underwent a reversible charge-transfer event upon visible light exposure. Subsequent *S*_RN_1 resulted in the departure of the halide (or triflate), generating a radical pair that recombined within the solvent cage to form a (hetero)­aryl or vinyl-cobalt intermediate **158**.^135^ Carbon monoxide insertion into this species yielded an acyl-cobalt intermediate, which then underwent nucleophilic substitution with the amine, affording the final amide product **155** and regenerating the catalyst. This methodology tolerated a broad array of (hetero)­aryl and vinyl halides and triflates, coupled with multiple amine nucleophiles, notably including ammonia surrogates.

**Scheme 19:**
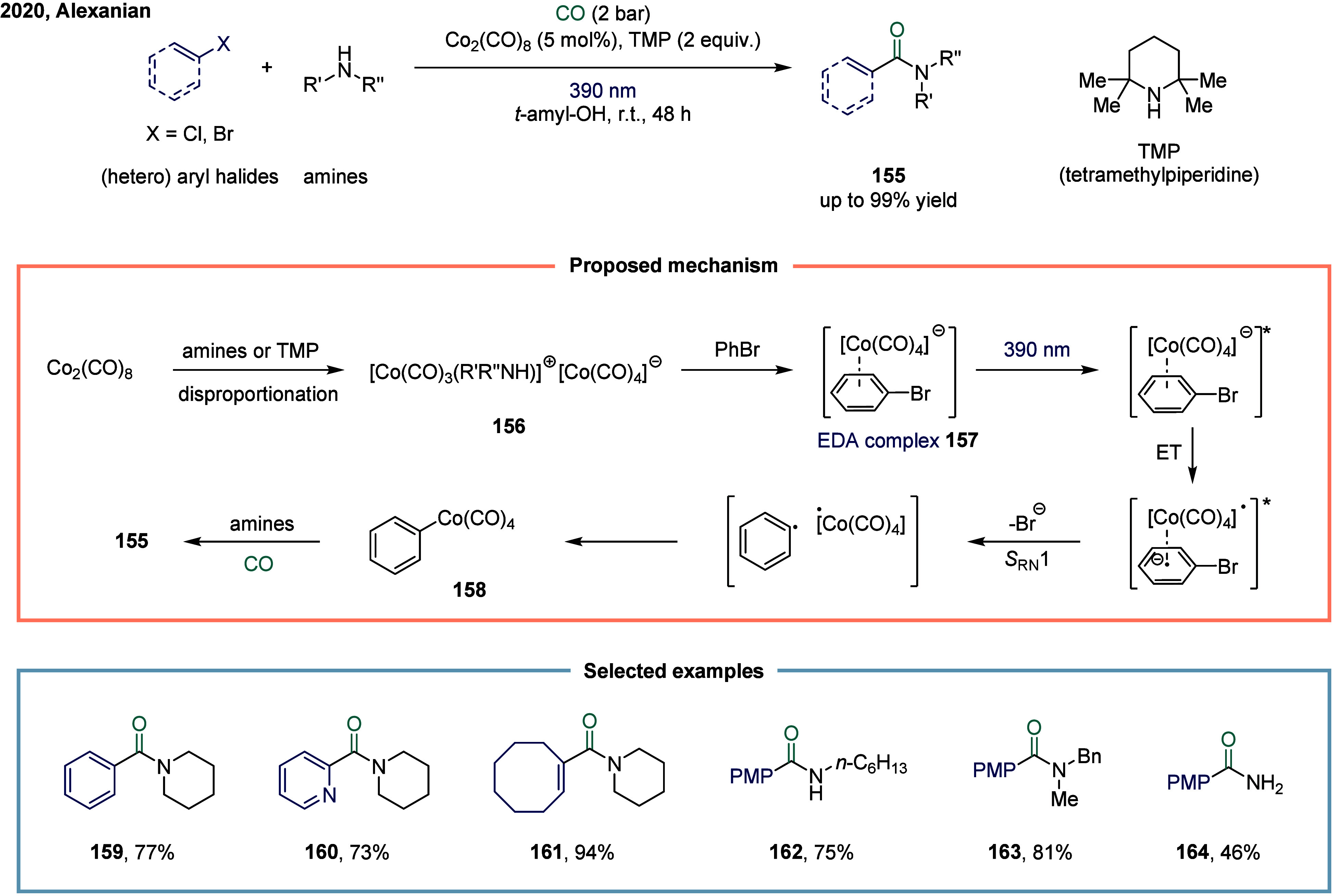
Cobalt-Catalyzed Aminocarbonylation of (Hetero)­aryl Halides Promoted by Visible Light

Inspired by the unique and widespread properties of thioesters, considerable attention has been devoted to their synthesis. However, harsh reaction conditions and the requirement for stoichiometric oxidants have continued to limit their broader application. In 2012, Li and co-workers developed a method employing simple thioesters as surrogates for thiols in the carbonylative thioalkylation of alkyl iodides, using a dual catalytic system of dithiane and isopropyl copper chloride (IPrCuCl).^136^ This catalytic protocol exhibited good functional group tolerance. Overall, this study provides an instructive example for the nonprecious metal-catalyzed carbonylative synthesis of sulfur-containing compounds.

Very recently, Wu and co-workers reported a cobalt-catalyzed *N,N,N*-tridentate ligand promoted carbonylation coupling of chloroacetonitrile for the synthesis of 2-cyano substituted acetates and amides (Scheme 20).^137^ 2-Cyano-*N*-acetamides and 2-cyanoacetates are of significant interest in the pharmaceutical industry, driving the development of novel synthetic methodologies.^138^ In the present reaction, the desired 2-cyano-substituted acetates and amides **165** were obtained in good to excellent yields. The protocol was scalable, and the resulting compounds can be readily transformed into bioactive molecules. The proposed mechanism suggested that chloroacetonitrile underwent a rapid radical process facilitated by a Co­(I) species. This transformation exhibited high functional group tolerance toward aromatic amines and alcohols, affording the corresponding products in excellent yields. However, aliphatic amines and phenols failed to undergo the desired reaction.

**Scheme 20:**
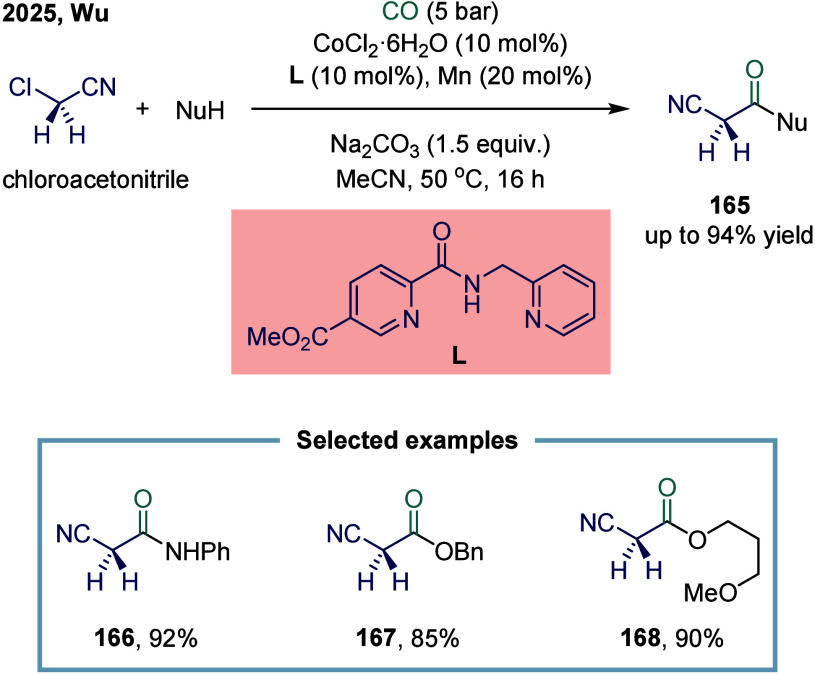
Ligand-Promoted Cobalt-Catalyzed Direct Carbonylation of Chloroacetonitrile to 2-Cyano Substituted Acetates and Amides

#### 2.5.3 Unsaturated Bonds

γ-Amino acids and their peptide analogues are widely utilized as key building blocks in the synthesis of biologically active molecules, pharmaceuticals, and natural products.^139–141^ In addition, γ-amino acids offer greater structural diversity in peptide design, as the presence of three carbon atoms between the amino and carbonyl groups allows for extended residue spacing and the incorporation of novel backbone conformations in encoded peptides.^142^ In 2023, Wu and co-workers described a cobalt-catalyzed aminoalkylative carbonylation of alkenes for the synthesis of γ-amino acid derivatives and peptides (Scheme 21).^143^ Mechanistically, the reaction was initiated by either thermal decomposition or 1e^–^ oxidation to generate a *tert*-butoxyl radical. This radical underwent a HAT process with substrate C­(sp^3^)-H, yielding the corresponding α-aminoalkyl radical intermediate **170**. The α-aminoalkyl radical then preferentially added to the terminal position of the alkene, affording a new carbon-centered radical species **172**. In pathway a, radical **172** was intercepted by a Co­(I) species to form the organometallic intermediate **173**, which subsequently coordinated with CO to afford the acyl-metal complex **175**. Alternatively, in pathway b, radical **172** first reacted with CO to generate an acyl radical species **176**, which was then trapped by the metal center. In both cases, the final reductive elimination step furnished the desired γ-amino acid derivatives and regenerated the Co­(I) catalyst, completing the catalytic cycle. Compared to iminium ions, the synthetic utility of α-amino radical remains limited. Intermolecular additions of α-amino radicals to simple unsaturated bonds often proceed with low efficiency, primarily due to polarity mismatch between the reacting species. Notably, the incorporation of electron-withdrawing groups, particularly alkyl acyl groups, effectively prevents the overoxidation of aminoalkyl radicals to iminium ions. This reaction integrated readily available amides, alkenes, and the feedstock gas carbon monoxide to construct architecturally complex and functionally diverse γ-amino acid derivatives in a single step via a radical relay catalytic strategy. This methodology exhibited excellent substrate compatibility, affording the desired products **176**-**180** in high yields. In particular, product **180** was formed with absolute selectivity.

**Scheme 21:**
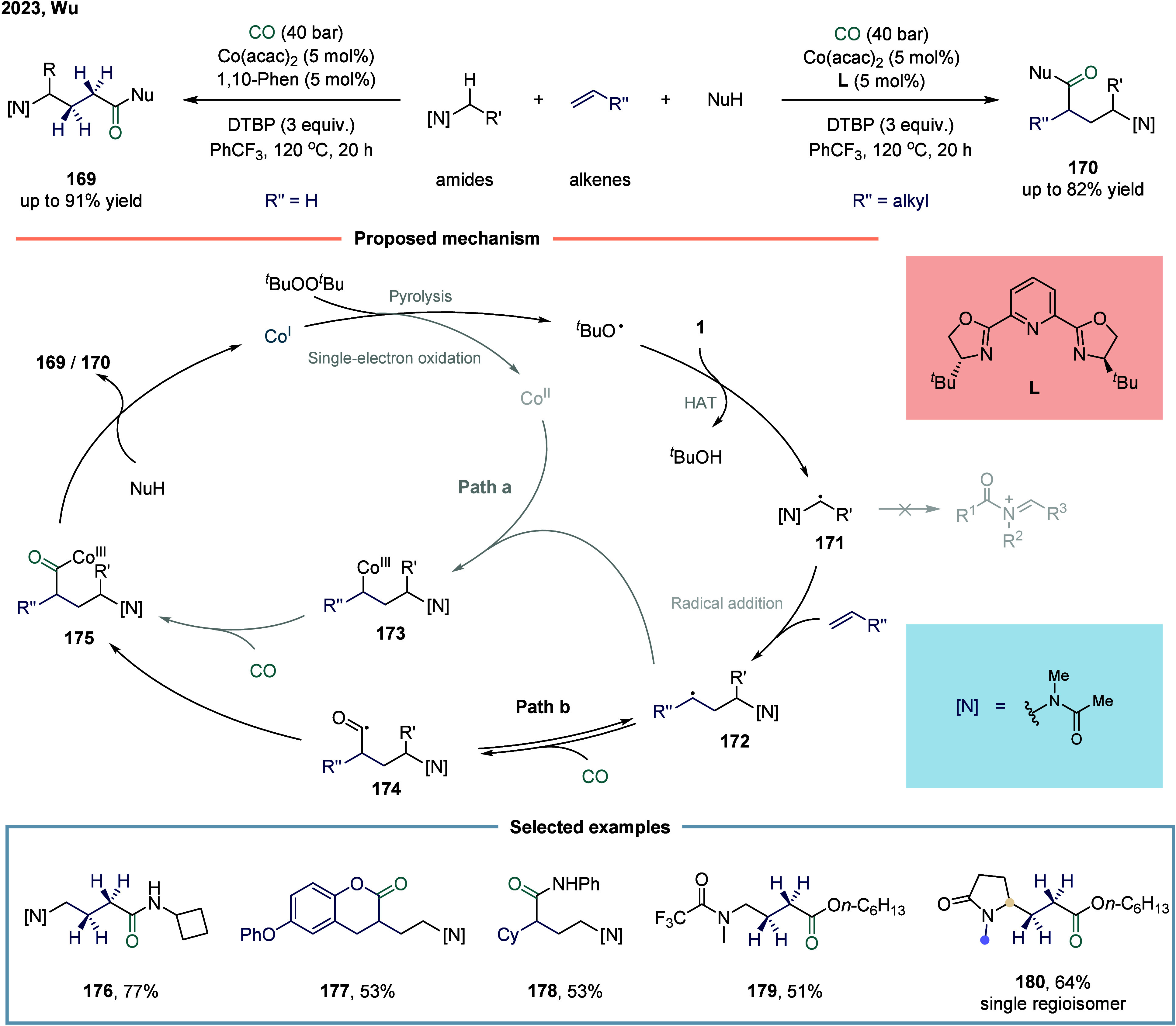
Cobalt-Catalyzed Aminoalkylative Carbonylation for γ-Amino Acid Derivatives and Peptides Synthesis

In 2022, Wu and co-workers applied methylarene as radical precursors in cobalt-catalyzed four-component carbonylation (Scheme 22a).^144^ A series of γ-aryl carboxylic acid esters **181** were obtained in moderate yields with high selectivity through this multicomponent reaction. The use of ethylbenzene as a substrate led to a significantly reduced yield, with only 30% of the target product **183** being obtained. In 2023, Wu and co-workers disclosed a catalytic approach for the direct difunctionalizative carbonylation of ethylene motifs, simultaneously installing amide and ester groups (Scheme 22b).^145^ Under ligand-free conditions and with a simple cobalt catalyst, this method provided access to a range of 4-oxobutanoates **184** from formamide, ethylene, and alcohols or phenols in moderate to good yields. This approach overcame the long-standing challenge of regioselectively and simultaneously introducing both ester and amide groups onto ethylene. By enabling the controlled installation of two distinct functional groups in a single step, it significantly streamlined synthetic routes and broadened the toolkit for constructing complex molecular architectures. However, only disubstituted formamides were compatible with this transformation, while ester group **187** was not tolerated.

**Scheme 22:**
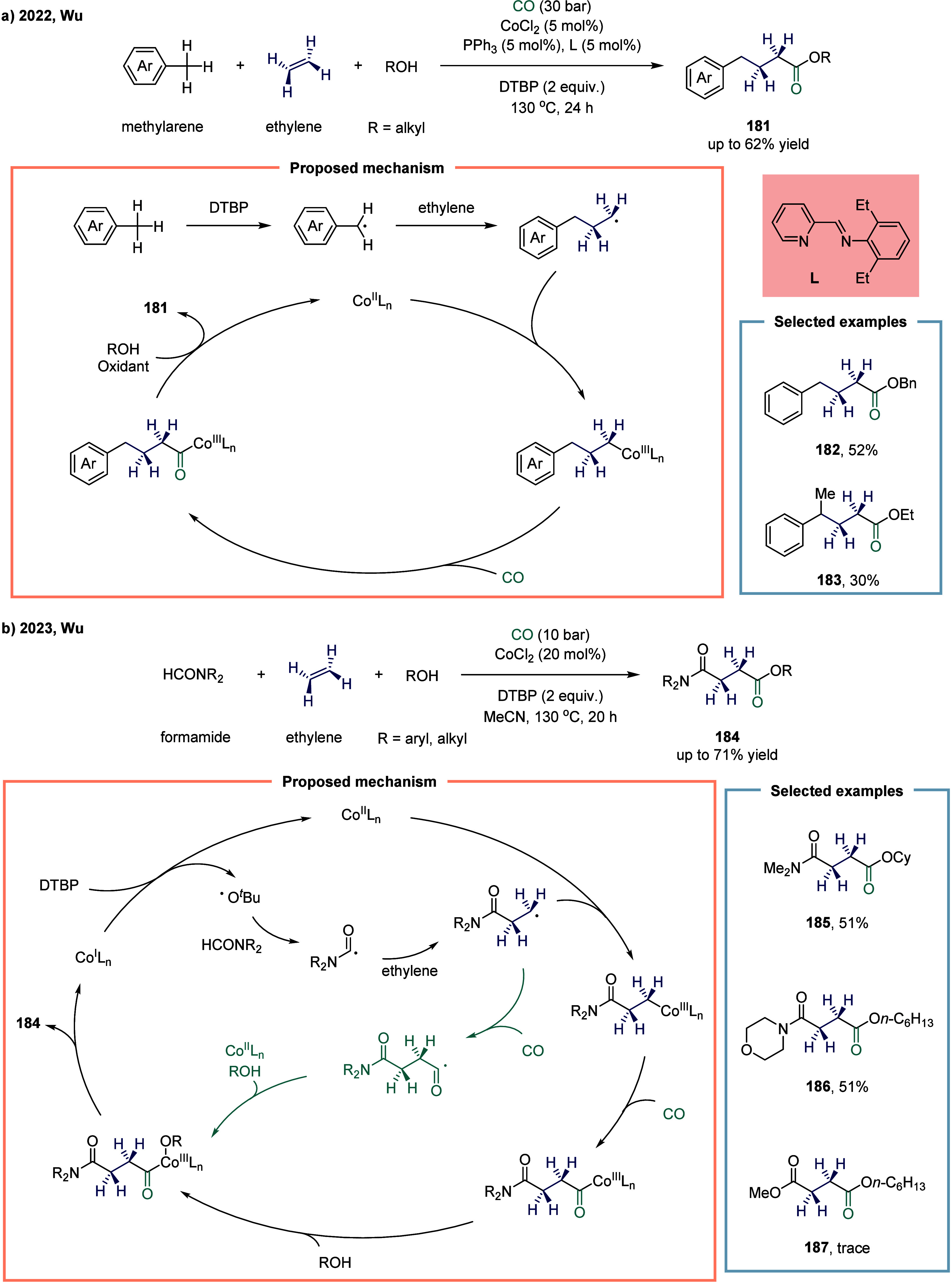
Cobalt-Catalyzed Four-Component Carbonylation of Methylarenes with Ethylene and Alcohols

The perfluoroalkylative carbonylation of alkenes represents an efficient and versatile approach for the synthesis of perfluoroalkyl carboxylic acid derivatives, key structural motifs found in high-performance materials and pharmacologically relevant molecules.^146^ Moreover, over 100 perfluoroalkyl carboxylic acid derivatives are in use today in diverse sectors, including industrial manufacturing and consumer products.^147^ In 2023, Beller and co-workers employed the direct activation of perfluoroalkyl iodides using a cobalt catalyst to achieve a multicomponent carbonylative coupling of alkenes, perfluoroalkyl iodides, and CO (Scheme 23).^148^ This protocol enabled the one-pot synthesis of β-perfluoroalkyl-substituted amides, esters, and related derivatives **188** using a variety of nucleophiles, including poorly nucleophilic amides and urea derivatives. Notably, carbonylation reactions involving weaker nucleophiles, such as amides, as coupling partners remain particularly scarce, primarily due to the inherently low reactivity associated with the final acylation step.^149,150^ To date, only a limited number of palladium-catalyzed carbonylations of aryl or vinyl halides with amides to afford the corresponding imides have been reported.^151–153^ In this reaction, a broad range of nucleophiles, including aliphatic and aromatic amines, alcohols, as well as more challenging weak nucleophiles such as (sulfon)­amides and ureas, were efficiently converted into the corresponding carboxylic acid derivatives with high regioselectivity. However, the highest yield for this transformation was obtained using the chiral ligand **L1**, although no enantioenrichment was observed. Notably, the use of the achiral ligand **L2** also afforded the desired product **188** in 57% yield.

**Scheme 23:**
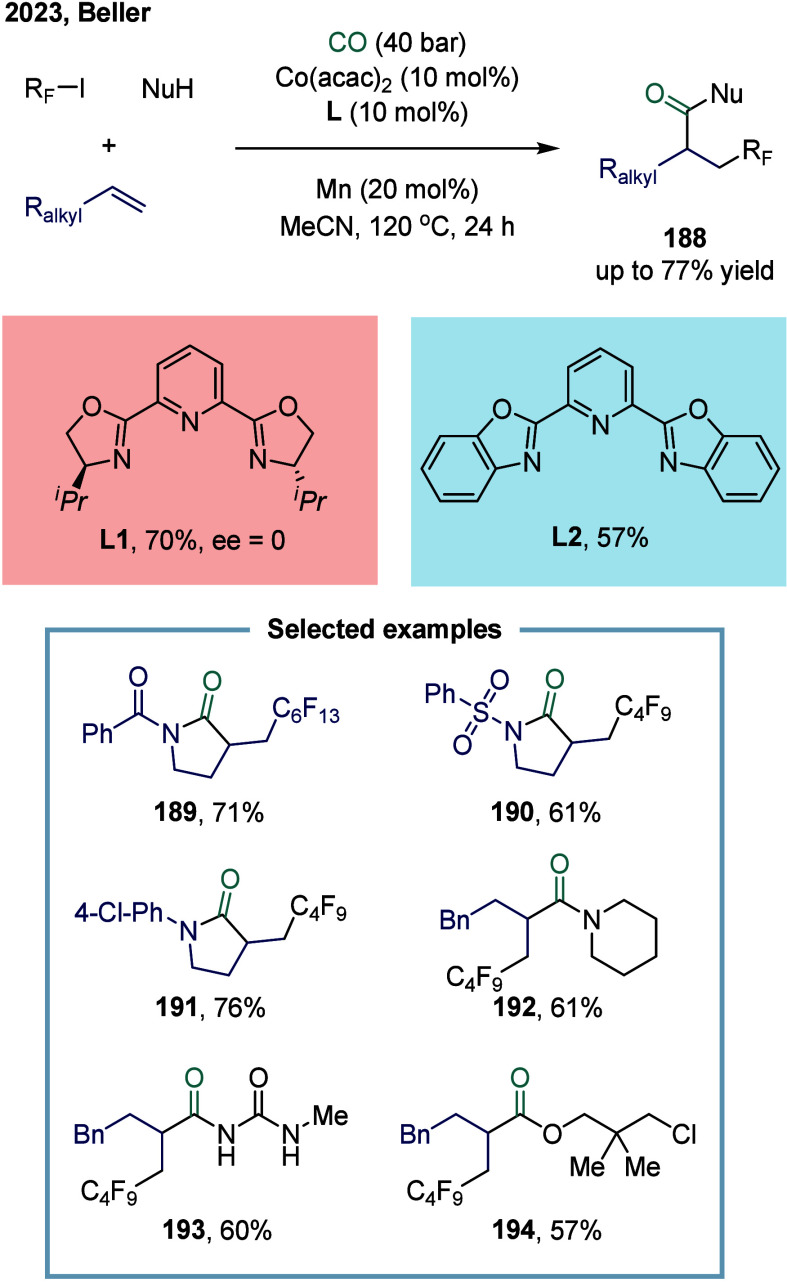
Cobalt-Catalyzed Carbonylation of Olefins: Efficient Synthesis of β-Perfluoroalkyl Imides, Amides, and Esters

Very recently, Li, He, and Guo coauthored a groundbreaking report on cobalt-catalyzed multicomponent carbonylation of alkenes enabling the divergent synthesis of unsymmetric ketones with excellent regio- and chemoselectivity (Scheme 24).^154^ The utilization of a tridentate *NNN*-type pincer ligand was critical in preventing the formation of catalytically inactive Co^0^(CO)_n_ species and suppressing oxidative carbonylation of organozinc reagents. This ligand effectively modulated the catalytic activity of the cobalt center, facilitating a fully cobalt-catalyzed four-component carbonylation process. The methodology proceeded under mild conditions (1 atm CO, 23 °C), exhibited a broad substrate scope, and demonstrated compatibility with a wide range of electrophilic radical precursors, including compounds **200**, **201**, **203**, **204**, and polyhalogenated compounds **204**-**207**. A key innovation of this study was the tandem electro-thermo-catalysis platform, which enabled the direct utilization of CO_2_ as a sustainable C1 source via in situ electrochemical reduction to CO, maintaining high efficiency and selectivity. Mechanistic investigations employing radical-trapping experiments, EPR spectroscopy, X-ray crystallography, and DFT calculations revealed a radical relay mechanism involving acyl-Co intermediates and a selective radical-type substitution pathway over oxidation. Initially, owing to the relatively low reducibility of OPiv-supported arylzinc reagents, the reduction of the Co­(II)­(bpp) complex by stoichiometric amounts of arylzinc pivalates selectively generated the Co­(I)­(bpp) species rather than the Co(0) complex, thereby preventing formation of catalytically inactive Co^0^(CO)_n_ species. Subsequent transmetalation between Co­(II) and arylzinc pivalates afforded the aryl-Co­(II)­(bpp) intermediate, which rapidly underwent 1,1-insertion of CO to yield the acyl-Co­(II)­(bpp) intermediate **197**. Alternatively, a radical addition of the benzyl radical to alkene occurred. The following radical substitution between the benzyl radical and intermediate **197** proceeded via transition state **198**, furnishing the final product.

**Scheme 24:**
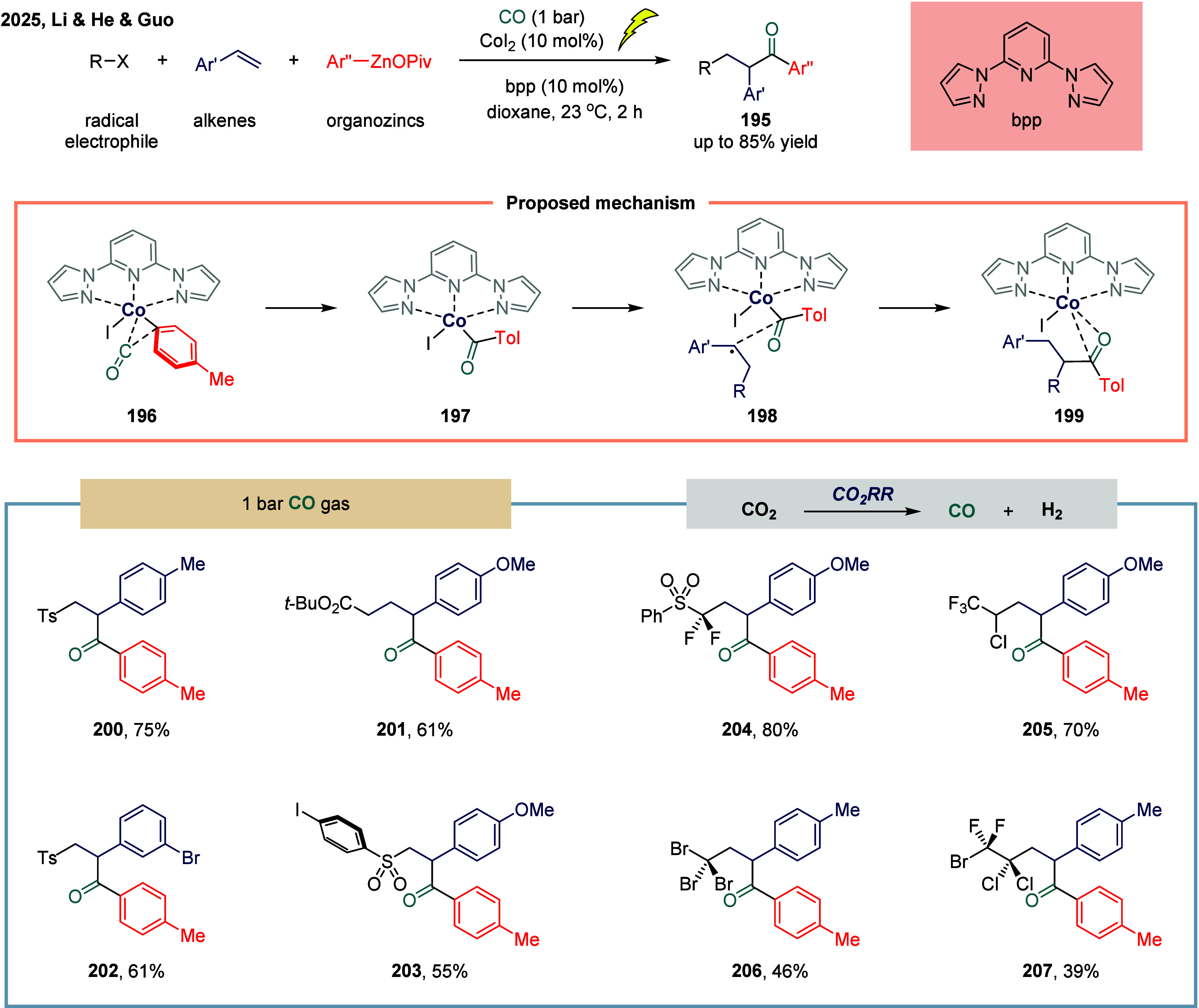
Pincer-Cobalt Boosts Divergent Alkene Carbonylation under Tandem Electro-Thermo Catalysis

Compared to monosubstituted alkenes, Markovnikov hydrocarbonylation of di- and trisubstituted alkenes remained challenging due to their lower binding affinity to the metal center and the sluggish insertion of the alkenes into the metal-hydride bond. These factors resulted in the formation of sterically hindered and unstable alkylmetal intermediates.^155^ In 2024, Cheng and co-workers reported a cobalt-catalyzed intramolecular Markovnikov hydrocarbonylation of unactivated alkenes (Scheme 25a).^156^ This protocol enabled the synthesis of a variety of α-alkylated γ-lactones and α-alkylated γ-lactams **208** in good yields. The mild reaction conditions tolerated mono-, di-, and trisubstituted alkenes bearing diverse functional groups. Mechanistic studies revealed that addition of Co­(III)-H to the alkene via a hydrogen atom transfer (HAT) process generated a metallo-/alkyl radical pair, which was subsequently trapped by CO to form a metallo-/acyl radical pair. This intermediate then underwent a radical-polar crossover (RPC) process to afford the target products. The optimized catalyst **[Co]-I** was crucial for the highly chemo- and regioselective formation of product **208**, achieving yields up to 98%. For 1,1-di- and 1,1,2-trisubstituted alkenes, **[Co]-II** proved to be the most efficient catalyst, delivering products such as **211** and **212** in good yields. Besides lactones, γ-lactams were also synthesized efficiently under these conditions **213**, and **214**.

**Scheme 25:**
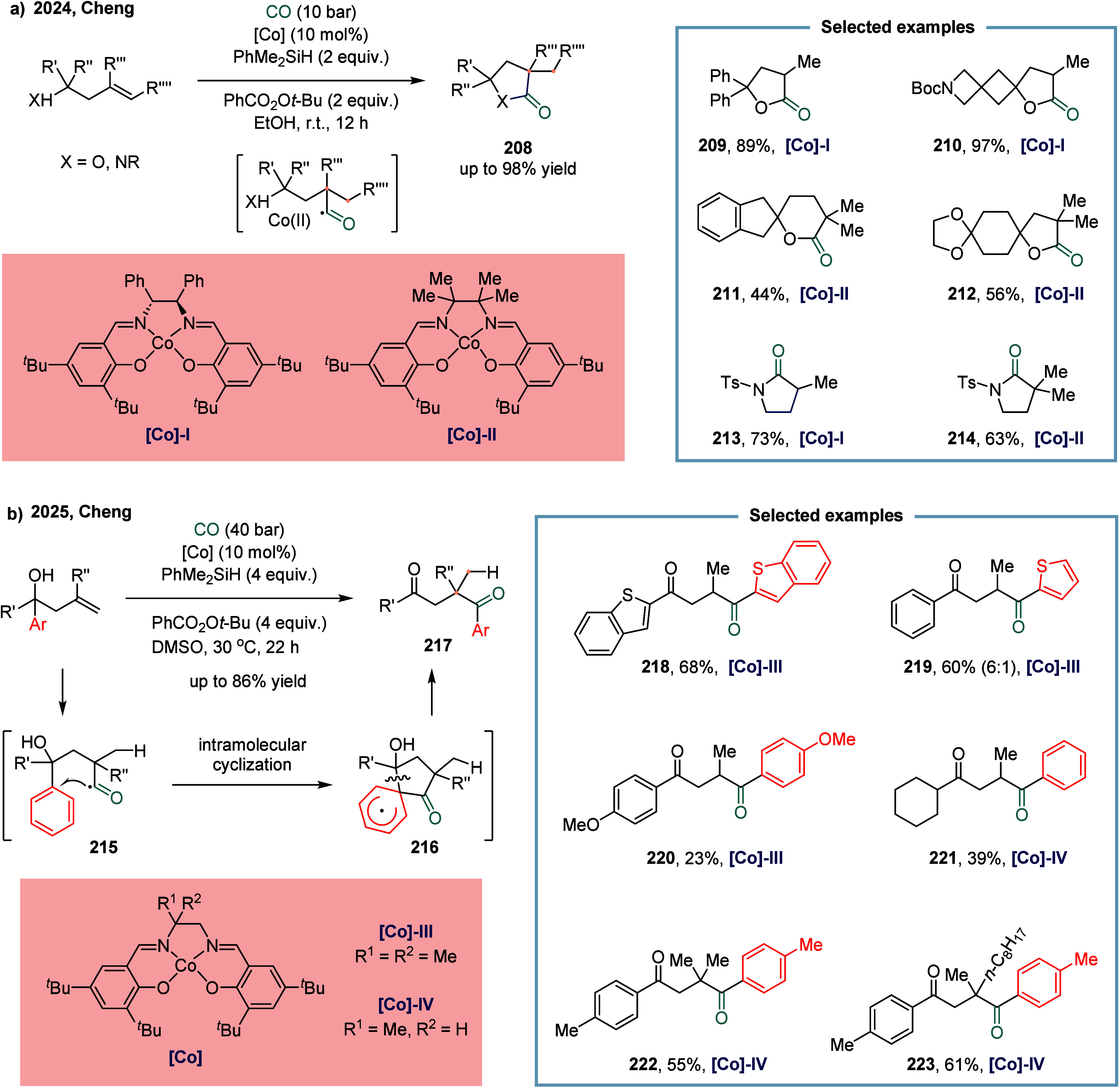
Cobalt-Catalyzed Markovnikov Hydrocarbonylation of Alkenes via HAT or Distal Aryl Migration

Later in 2025, Cheng and co-workers reported a cobalt-catalyzed Markovnikov hydroarylcarbonylation of unactivated alkenes via distal aryl migration (Scheme 25b).^157^ In this reaction, the Markovnikov hydroarylcarbonylation of unactivated alkenes was accomplished by integrating HAT catalysis with a distal aryl migration process. Specifically, the acyl radical **215**, generated from a homoallylic alcohol via HAT under a CO atmosphere, initiated a kinetically favorable five-membered cyclic transition state **216** that facilitated distal aryl migration. This sequence ultimately yielded α-alkylated 1,4-diketones through C–C bond cleavage. In this protocol, CO served a dual function: it not only acted as a one-carbon (C1) source for carbonylation but also mediated the transformation of the homoallylic alcohol from a thermodynamically disfavored 1,3-aryl migration pathway to a more favorable 1,4-aryl migration via one-carbon chain extension. Under the optimized reaction conditions, heteroaromatic groups such as thiophene underwent preferential chemoselective migration over aryl groups, yielding compound **219** as the major product in a 6:1 ratio relative to its isomer. However, the benzene ring bearing an electron-donating group yielded the migration product **220** in low yield, primarily due to the formation of a benzocyclohexanone byproduct. This side product resulted from the intramolecular cyclization of the acyl radical onto the electron-rich arene. Substrates bearing only a single aromatic ring capable of migration were compatible with the reaction. Although the ketyl intermediate was less stabilized by alkyl substituents, these more challenging migrations proceeded smoothly. The 1,4-diketone product containing α-quaternary carbon centers **222** was successfully obtained in 55% yield using **[Co]-IV** as the catalyst.

#### 2.5.4 Others

Ketenes have long intrigued chemists due to their unique physical properties and exceptionally diverse chemical reactivity.^158,159^ In 2013, Bruin and co-workers reported a cobalt-porphyrin-catalyzed carbene carbonylation reactions (Scheme 26a).^160^ This reaction demonstrated that [Co­(II)­(Por)] complexes functioned as effective metallo radical catalysts for carbene carbonylation, enabling the formation of ketenes from carbon monoxide and diazo compounds under mild conditions. The [Co­(II)­(Por)]-catalyzed process proceeded via a low-energy barrier carbene carbonylation step and provided a valuable synthetic alternative for ketene generation. The in situ generated ketenes were efficiently trapped by amines or imines to yield amides **224** or β-lactams **225** in up to 75% and 67% yields, respectively, in a one-pot cascade transformation. This concise methodology featured a broad substrate scope and accommodated diverse combinations of diazo compounds with nucleophiles or imines.

**Scheme 26:**
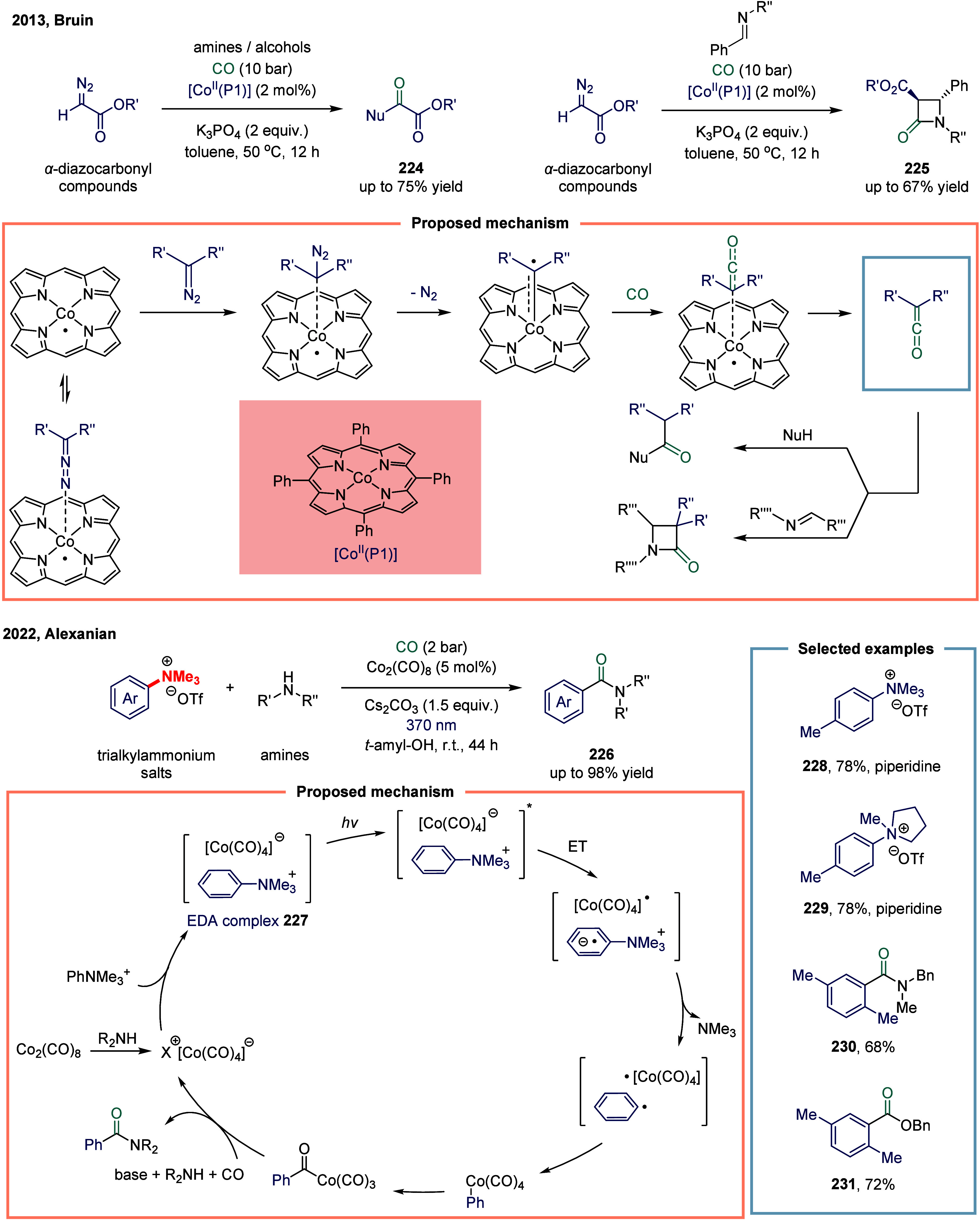
Cobalt-Catalyzed Carbene Carbonylation and Deaminative Amino- and Alkoxycarbonylation

In 2022, Alexanian and co-workers developed a cobalt-catalyzed photoinduced deaminative amino- and alkoxycarbonylation of aryl trialkylammonium salts (Scheme 26b).^161^ The reaction proceeded under mild conditions, making it suitable for late-stage functionalization and amenable to telescoped carbonylation processes initiated directly from anilines. A broad range of alkylamines functioned as effective coupling partners **228**-**231**, and the feasibility of alkoxycarbonylation was also demonstrated. Mechanistic studies, bolstered by DFT calculations, provided deep insights into the catalytic cycle. These investigations uncovered a novel carbonylation pathway unique to aryl electrophiles under the cobalt catalytic system. A key mechanistic highlight is the involvement of a visible-light-induced carbonyl photodissociation step, which generates reactive cobalt–carbonyl intermediates critical for facilitating the coupling.

Unsymmetrical ureas are ubiquitous structural motifs in a wide range of clinically approved pharmaceuticals, including antipsychotic agents, anti-HIV drugs, antibiotics, and various other therapeutics.^162,163^ In 2024, Lei and co-workers reported a cobalt- and copper-catalyzed oxidative carbonylation for the synthesis of unsymmetrical ureas (Scheme 27).^164^ This reaction utilized a synchronous recognition strategy that integrated both radical and nucleophilic activation to differentiate between secondary and primary amines. Specifically, a copper catalyst selectively oxidized secondary amines to generate aminyl radicals, while a cobalt catalyst carbonylated primary amines to form cobalt amide intermediates. The coupling of these two reactive species through cooperative catalysis facilitated the efficient and selective synthesis of unsymmetrical ureas **232**. This strategy capitalizes on the inherent difference in oxidation potentials between secondary and primary amines. Secondary amines are more readily oxidized, as evidenced by their lower oxidation potential compared to primary amines.^165^ Moreover, the lower steric hindrance of primary amines likely enhances their ability to coordinate with metal centers, rendering them more reactive in metal-mediated transformations than secondary amines.^166^ Additionally, the authors also developed a tandem electrocatalytic-thermocatalytic system that first electroreduced CO_2_ to CO, followed by oxidative carbonylation to synthesize unsymmetrical ureas (pathway II), providing an alternative to the direct use of CO in pathway II. The yields of compound **234** was 91% via pathway I and 88% via pathway II, while product **235** was obtained in 83% and 76% yields, respectively, demonstrating comparable reactivity between the two pathways. A diverse series of biologically active urea derivatives were successfully synthesized under optimized reaction conditions, affording moderate to good yields across a broad range of substrates. These compounds exhibited promising structural characteristics relevant to their targeted biological activities **238**, **239**, and **240**, underscoring the efficiency and versatility of the synthetic methodology.

**Scheme 27:**
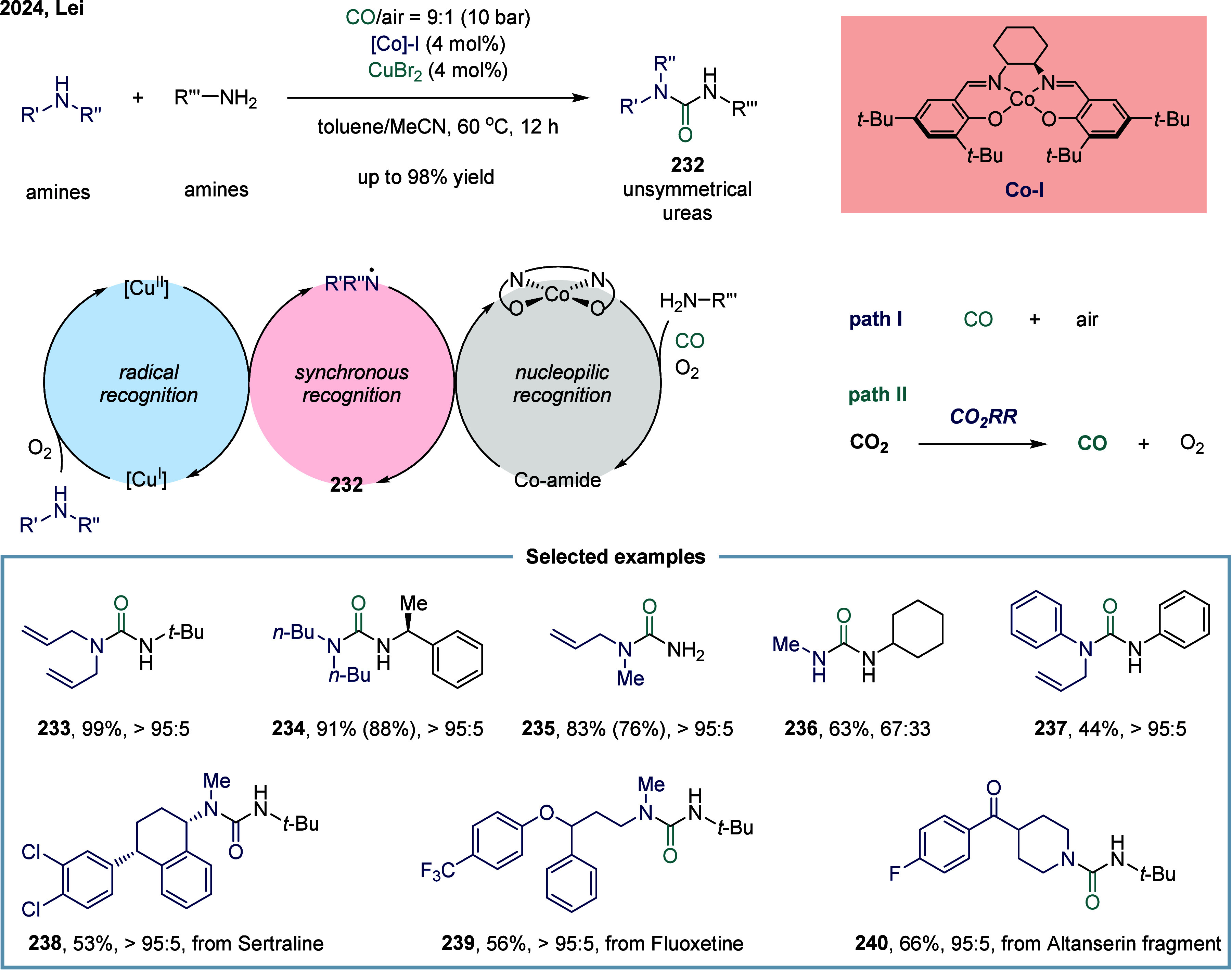
Synchronous Recognition of Amines in Oxidative Carbonylation toward Unsymmetrical Ureas

### 2.6 Nickel-Catalyzed System

The application of nickel in carbonylation chemistry dated back to 1890, when German chemist Ludwig Mond and his collaborators discovered that CO reacted with nickel powder at 50 °C under atmospheric pressure to form Ni­(CO)_4_, a colorless liquid at room temperature.^167^ This pioneering discovery not only marked the beginning of nickel carbonyl chemistry but also laid the foundation for its industrial application. Ni­(CO)_4_ was subsequently produced on a large scale as a key intermediate in the Mond process, which was widely employed for the purification and refining of nickel.^168^ Industrially, BASF established the first commercial process for acetic acid synthesis via nickel-catalyzed methanol carbonylation in the 1950s. The formation of Ni­(CO)_4_ is attributed to the strong binding affinity between π-acidic carbon monoxide and nickel.^169^ However, due to its saturated coordination sphere, Ni­(CO)_4_ exhibits limited reactivity in oxidative addition of C-X bonds and in the migratory insertion of CO into Ni–C bonds.^170^ As a result, the direct use of inexpensive and abundant CO gas in nickel-catalyzed carbonylation reactions has remained a significant challenge. To overcome this limitation, various strategies have been developed. One common approach involves the use of carbon monoxide surrogates, such as chloroformates, metal carbonyl complexes, and formic acid, in nickel-catalyzed carbonylative cross-coupling reactions that proceed.^171–174^ In addition, the controlled, gradual release of carbon monoxide (CO) significantly enhanced the nickel-catalyzed carbonylation reaction. More recently, notable advances have been achieved in SET-mediated carbonylation processes, wherein nickel catalysts enable radical-type carbonylative transformations under milder and more versatile conditions.

#### 2.6.1 Carbon–Hydrogen Bonds

Huang and co-workers realized the utility of accessing high-valent arylacetic acids under earth-abundant metal catalysts in the coupling of alkylarenes. Arylacetic acids serve as valuable intermediates in the organic synthesis of pharmaceuticals, agrochemicals, and fragrances.^175^ In the pharmaceutical industry, they are particularly important for the production of compounds such as penicillin and dimethoate.^176^ In 2022, a nickel-catalyzed oxidative carbonylation of alkylarenes with H_2_O for the efficient synthesis of arylacetic acids was reported by Huang’s laboratory (Scheme 28).^177^ In this transformation, a catalytic system comprising NiBr_2_ and diphenylphosphine oxide was developed, enabling the direct synthesis of value-added arylacetic acids from readily available alkylarenes and water via oxidative carbonylation. This protocol exhibited broad substrate scope, accommodating both primary and secondary benzylic C–H bonds. Notably, this method provided a concise and practical route to pharmaceutically relevant compounds, including the commercial drugs ibuprofen **244**. However, it should be noticed that this method demands a large excess of alkylarenes. Further mechanistic investigations revealed that the reaction proceeds via two distinct pathways, wherein the nickel­(I) catalyst intercepts either acyl radicals (path I) or benzyl radicals (path II).

**Scheme 28:**
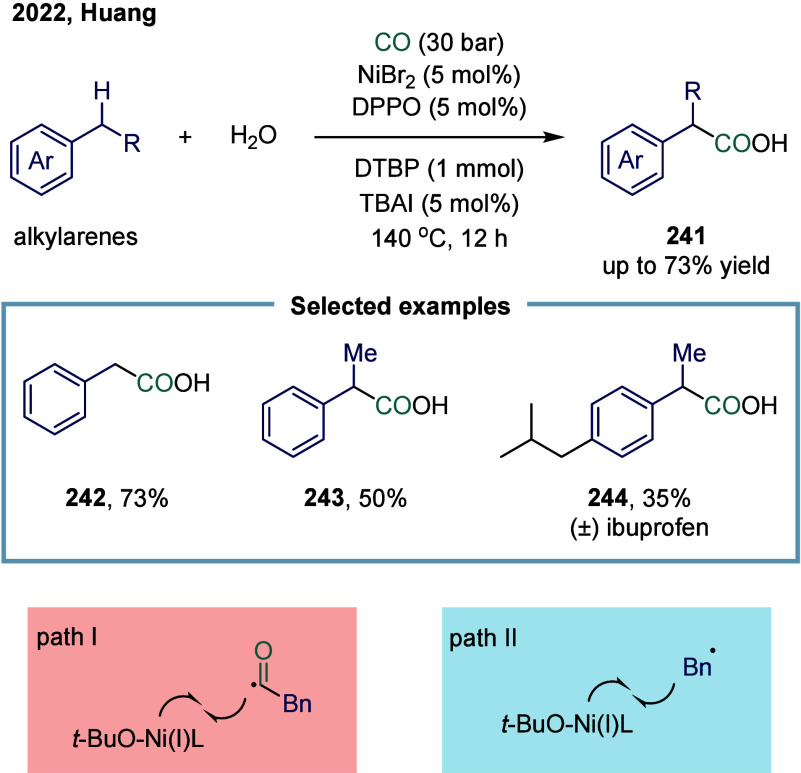
Nickel-Catalyzed Oxidative Carbonylation of Alkylarenes to Arylacetic Acids

In 2023, Liang and co-workers developed a novel and efficient method for the catalytic installation of CO via remote radical coupling (Scheme 29).^178^ The transformation proceeded through a sequential single-electron transfer, 1,5-hydrogen atom transfer, and subsequent CO insertion. Notably, the reaction was performed under ambient pressure and redox-neutral conditions, exhibiting broad functional group tolerance and exceptional site-selectivity.

**Scheme 29:**
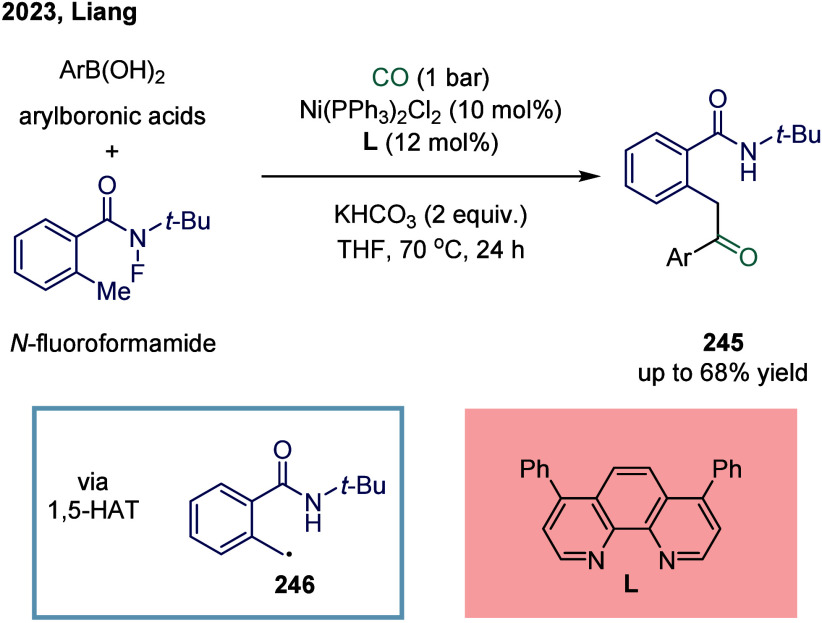
Nickel-Catalyzed Remote C–H Carbonylation via Redox Neutral Radical-Relay Carbonylation

#### 2.6.2 Carbon–Halogen Bonds

Although significant progress has been made in nickel-catalyzed carbonylation of C­(sp^2^)-halogen bonds,^179^ the corresponding nickel-catalyzed carbonylative transformations of aliphatic electrophiles remain underdeveloped and warrant further investigation. Even within the context of well-established palladium-catalyzed protocols, the carbonylation of secondary alkyl electrophiles with CO to generate alkyl ketones remains elusive, primarily due to the intrinsic tendency toward undesired β-hydride elimination, which competes with the targeted reductive elimination step.^180–182^

In recent years, several research groups have employed carbon monoxide surrogates to enable the gradual release of CO, thereby mitigating the formation of inactive Ni­(CO)_4_ species.^183^ Ogoshi and co-workers effectively employed benzoic acid as a CO surrogate in a Ni(0)-catalyzed aza-Pauson-Khand reaction for the synthesis of lactams, while direct exposure to CO gas was found to impede the carbonylative cycloaddition process.^184,185^ Similarly, the Troupel and Weix groups demonstrated proof of concept for Ni(0)-catalyzed carbonylative cross-electrophile coupling between aryl and alkyl halides, employing Fe­(CO)_5_ as a carbon monoxide source.^186,187^ In 2017, Skrydstrup and co-workers reported a strategy to prevent catalyst poisoning by leveraging the strong tridentate coordination of a pincer ligand to the nickel­(II) center, while simultaneously regulating the release of carbon monoxide. (Scheme 30a).^188^ Using this nickel­(II) pincer complex as a catalyst, the carbonylative Negishi coupling of benzyl bromide with alkyl zinc reagent was successfully accomplished. This study represented the first documented example of a nickel-catalyzed carbonylative coupling between two sp^3^-carbon fragments. In this transformation, nickel­(II) pincer complex **248** was particularly suitable owing to the strong tridentate coordination of the pincer ligand to the nickel center, which likely prevents the binding of multiple CO units to certain reactive intermediates. The mechanism suggested that a SET from complex **249** to an electrophile generated a nickel­(III) complex and a benzyl radical. This radical recombined with a second nickel­(II) acyl species **249** to form the nickel­(III) acylalkyl complex **250**. A range of benzyl alkyl ketones were successfully synthesized employing a two-chamber technology. This two-chamber technology enables controlled and gradual release of CO, thereby preventing the formation of less reactive nickel species.

**Scheme 30:**
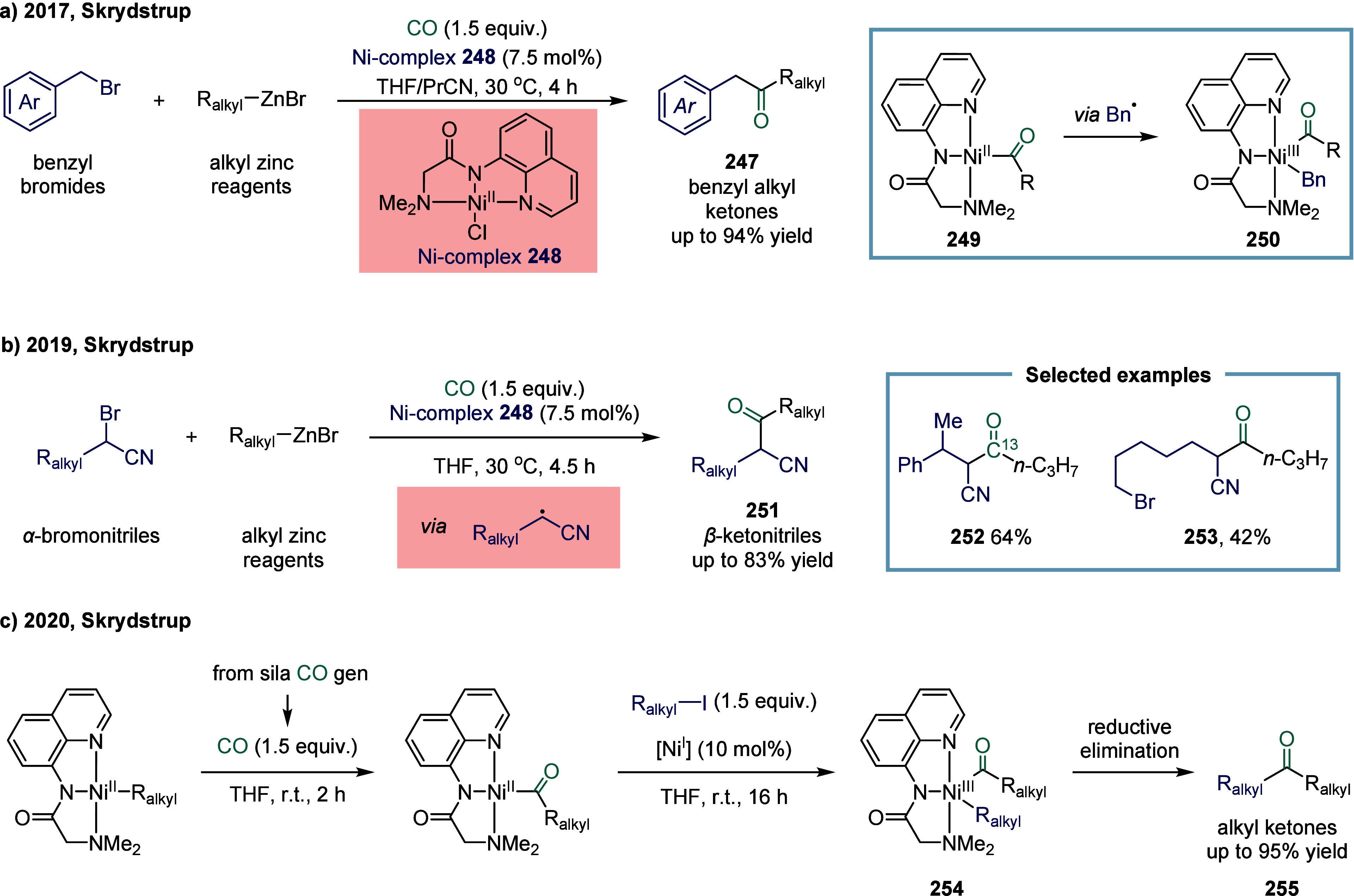
Carbonylative Coupling Reaction Catalyzed by a Nickel/NN_2_ Pincer Ligand Complex

Subsequently, the Skrydstrup group expanded the substrate scope from alkyl bromides to α-bromonitriles, affording β-ketonitriles in up to 83% yield (Scheme 30b).^189^ This transformation was catalyzed by a readily accessible and stable nickel­(II) pincer complex **249**. The developed protocol demonstrated broad functional group tolerance, effectively overcoming limitations associated with previous synthetic methodologies. Furthermore, the authors illustrated the method’s applicability for carbon isotope labeling via the synthesis of ^13^C-labeled β-ketonitriles **252**. Mechanistic studies indicated that the reaction proceeded via bromide abstraction from the α-bromonitrile substrate, generating a nickel­(III) complex alongside a nitrile-stabilized carbon-centered radical.

Both aforementioned studies by Skrydstrup employed NN_2_ pincer nickel catalyst for the carbonylative coupling of activated aliphatic halides with alkyl zinc reagents. The key to the success of this transformation was the utilization of a two-chamber technology that gradually released one equivalent of CO, which was subsequently inserted into the pincer Ni­(II)-alkyl complex to form the corresponding Ni­(II)-acyl species. However, attempts to extend this protocol to unactivated alkyl iodides had thus far proven unsuccessful. To overcome this limitation, Skrydstrup and co-workers subsequently reduced the pincer Ni­(II) halide to the corresponding Ni­(I) complex, a more active reducing agent (Scheme 30c). This complex readily reacted with alkyl iodides to generate an alkyl radical, followed by the formation of a Ni­(III)-(alkyl)­acyl complex **254**.^190^ Subsequent reductive elimination yielded the desired keto product **255** (up to 95% yield) and regenerated the initial Ni­(I) species. This reaction effectively enabled the synthesis of a diverse array of functionalized ^12^C- and ^13^C-labeled aliphatic ketones via Ni­(I)-mediated activation of NN_2_ pincer Ni­(II)-acyl complexes and primary or secondary alkyl iodides.

An alternative approach to overcoming the aforementioned limitations in nickel-catalyzed carbonylation reactions involves the use of organic CO surrogates. In 2019, Hu and co-workers developed an efficient carbonylative coupling of two alkyl halides with ethyl chloroformate (ClCOOEt), a safe and easily handled CO source (Scheme 31a).^191^ A variety of asymmetric **257–259** and symmetric **261, 262** dialkyl ketones were successfully delivered in good to excellent yields, whereas tertiary halide afforded lower yield **260**. These carbonylation reactions proceeded under mild conditions and exhibited broad substrate scope as well as high functional-group tolerance. Mechanistically, ethyl chloroformate underwent oxidative addition to Ni(0), affording Ni­(II) species, which subsequently underwent decarbonylation to yield the Ni­(II)-carbonyl complex **263**. Reduction of intermediate **263** by zinc produced the Ni(0)-carbonyl species **264**. Species **264** then activated the alkyl halide via a SET process, generating the Ni­(II)-alkylcarbonyl intermediate **265**, which underwent CO insertion to form the Ni­(II)-acyl complex **266**. Concurrently, a Ni­(I) species activated a second alkyl halide, producing an alkyl radical that was intercepted by complex **266** to afford the Ni­(III)-alkylacyl species **267**. Finally, reductive elimination delivered the ketone product and regenerated the Ni­(I) species. Notably, noncarbonylative alkyl–alkyl coupling was not observed, suggesting that alkyl halide activation by the Ni(0)-CO species and subsequent CO insertion to form complex **266** occurred faster than activation of the second alkyl halide by the Ni­(I) species. In the initial activation step, primary alkyl halides likely exhibited higher reactivity than secondary alkyl halides due to reduced steric hindrance. Conversely, in the latter step, secondary alkyl halides may have been more reactive than primary alkyl halides for thermodynamic reasons. Furthermore, the resulting acyl species preferentially reacted with primary alkyl radicals over secondary radicals, leading to enhanced selectivity toward unsymmetrical dialkyl ketone formation.

**Scheme 31:**
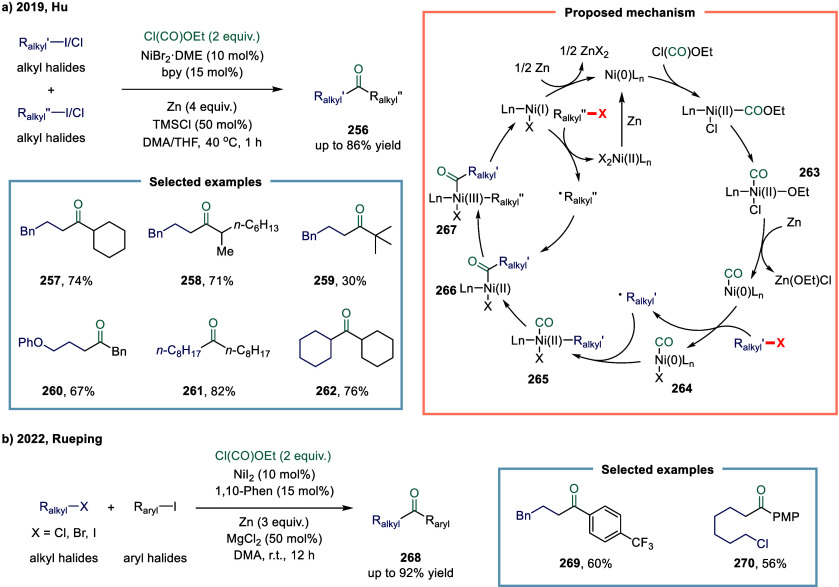
Nickel-Catalyzed Reductive Carbonylation of Alkyl Halides Utilizing Ethyl Chloroformate as the Carbonyl Source

Reductive cross-electrophile carbonylative coupling has emerged as a powerful and efficient strategy for constructing structurally complex organic molecules. Inspired by Hu’s work on nickel-catalyzed reductive carbonylation using ethyl chloroformate as a carbonyl source, Rueping and co-workers developed a nickel-catalyzed system for multicomponent sequential reductive cross-coupling reactions (Scheme 31b).^192^ The carbonylation reaction proceeded under mild conditions, exhibited broad applicability, and demonstrated high functional-group tolerance, offering an efficient and practical strategy for the synthesis of aryl-alkyl ketones **268** in up to 92% yield. DFT calculations, supported by experimental results, provided mechanistic insights into the complex sequence involving three distinct electrophiles, suggesting that oxidative addition occurred first with aryl halides, followed by ethyl chloroformate, and finally alkyl bromides. These findings were expected to inform future advances in synthetic planning and the development of multicomponent cross-coupling reactions.

Since *C*-glycosides had a longer lifetime and retained their functionality under biological conditions, they attracted extensive attention as alternatives to bioactive sugars in the design of carbohydrate-based therapeutic candidates.^193,194^ In 2022, Koh and co-workers developed a nickel-catalyzed reductive carbonylation multicomponent synthesis of *C*-acyl glycosides from glycosyl halides, organic iodides, and commercially available isobutyl chloroformate as a CO surrogate (Scheme 32a).^195^ This method demonstrated broad functional group compatibility, and the resulting products exhibited high diastereoselectivity. It also enabled the rapid assembly of otherwise challenging *C*-acyl glycosides and facilitated late-stage keto-glycosidation of oligopeptides. This transformation enabled the sequential activation of three substrates, thereby achieving two consecutive cross-electrophile coupling events and suppressing the formation of unwanted carbonylation self-coupling byproducts. By employing distinct nickel catalysts and ligands, carbonylative coupling reactions of glycosyl halides with aryl iodides and alkyl iodides, respectively, can be achieved in the presence of isobutyl chloroformate delivering the corresponding products **271** and **272** in 72% and 62% yields, respectively.

**Scheme 32:**
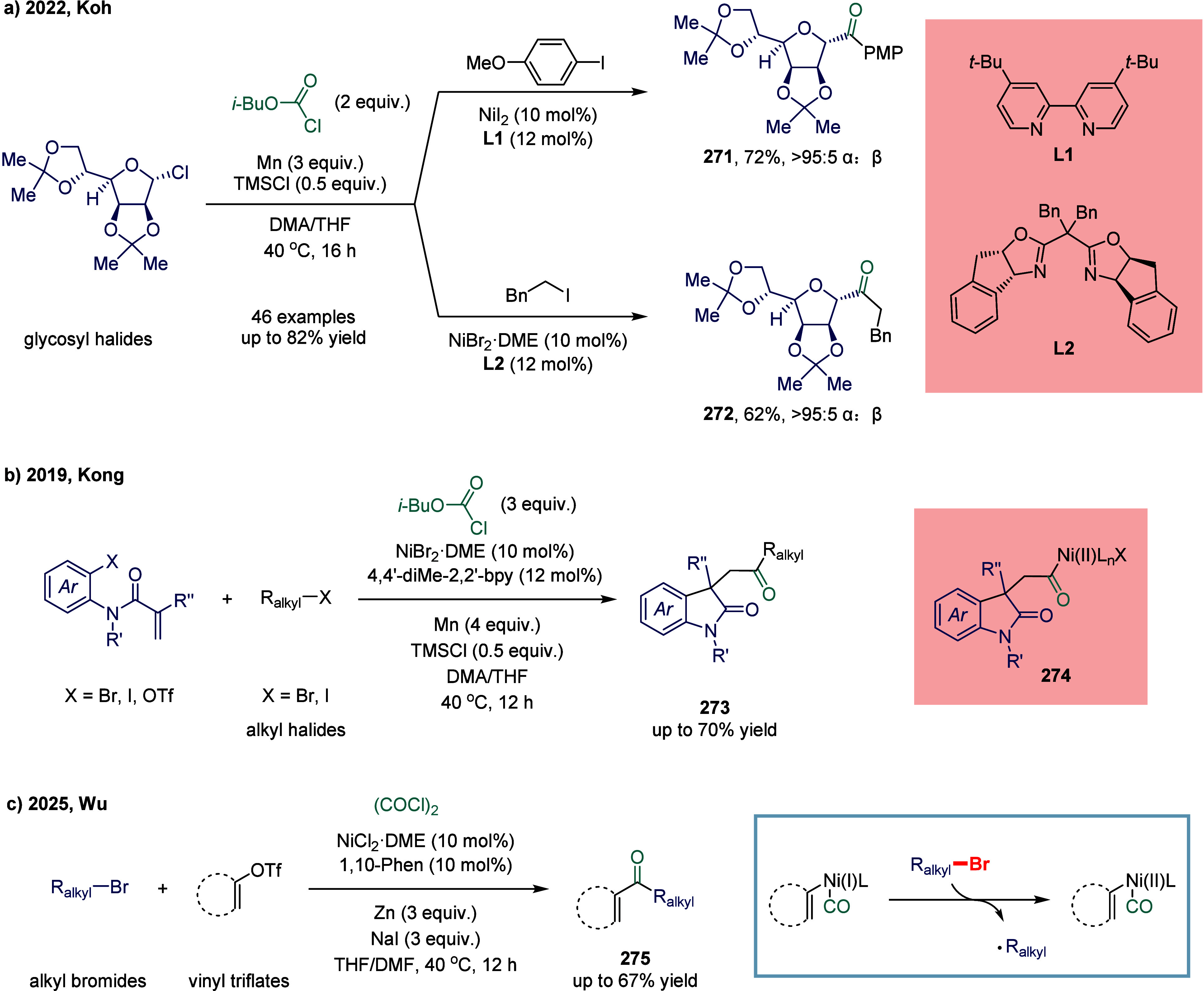
Nickel-Catalyzed Reductive Carbonylation of Glycosyl Halides/Alkyl Halides

In 2019, Kong and co-workers reported a nickel-catalyzed reductive arylacylation of alkenes toward carbonyl-containing oxindoles (Scheme 32b).^196^ This reaction proceeded under mild conditions without the need for toxic carbon monoxide gas or metal carbonyl reagents. Moreover, the method enabled the efficient synthesis of 3,3-disubstituted oxindoles **273** bearing an all-carbon quaternary stereocenter and a ketone functional group, delivering good yields across a broad substrate scope. The alkyl radical rapidly reacted with acyl species **274**, leading to the selective formation of dialkyl ketones. In 2025, Wu and co-workers developed a nickel-catalyzed reductive carbonylation of vinyl triflates and alkyl bromides for the synthesis of enones **275** in up to 67% yield (Scheme 32c).^197^ Using oxalyl chloride as a convenient and bench-stable carbonyl source, a range of alkyl alkenyl ketones was synthesized in moderate to good yields under mild reaction conditions. Mechanistic investigations revealed that the synergistic effect of DMF and zinc played a critical role in promoting in situ CO release from oxalyl chloride.

In 2020, Zhang and co-workers disclosed a nickel-catalyzed carbonylation of aliphatic electrophiles with arylboronic acids under 1 bar of carbon monoxide (Scheme 33a).^198^ This protocol exhibited broad substrate scope and excellent functional group tolerance, accommodating a variety of secondary alkyl iodides and benzyl bromides bearing trifluoromethyl **277**, difluoromethyl **278**, and other electron-withdrawing groups **279**. The method provided a practical and cost-efficient approach for the synthesis of alkyl ketones **276**, particularly α-trifluoromethylated ketones, which hold considerable significance in pharmaceutical chemistry. Mechanistic investigations suggested that the catalytic cycle commenced with a transmetalation between an arylboronic acid and a nickel­(II) precursor, forming an aryl-nickel­(II) intermediate **280**. Subsequent insertion of carbon monoxide afforded an acyl-nickel­(II) species **281**, which then underwent single-electron oxidation with a secondary alkyl halide to generate both an alkyl radical and a nickel­(III) intermediate **282**. The alkyl radical subsequently combined with another acyl-nickel­(II) complex to form a key nickel­(III) species **283**, which delivered the final ketone product via reductive elimination. This transformation proceeded via transmetalation between a nucleophile and a nickel species to generate a [Ni^x^(L_n_)-Nu] intermediate, which simultaneously suppressed the formation of catalytically inactive species such as Ni­(CO)_4_ or NiL­(CO)_3_. However, the reaction was limited to secondary aliphatic electrophiles, with primary or tertiary counterparts proving ineffective under the optimized conditions. In 2019, Zhang and co-workers also reported a nickel-catalyzed carbonylation of difuoroalkyl bromides with arylboronic acids under 1 bar of CO (Scheme 33b).^199^ This methodology exhibited broad substrate compatibility, efficiently coupling various arylboronic acids with difluoroalkyl bromides while maintaining excellent tolerance to diverse functional groups. It provided an economical and straightforward route to access difluoroalkyl ketones **284**. Notably, a substantial portion of the synthesized difluoroalkyl ketones featured alkynyl functionalities that had not been previously documented, positioning them as valuable intermediates for the synthesis of a wide array of fluorinated molecules with important applications in medicinal chemistry and materials science.

**Scheme 33:**
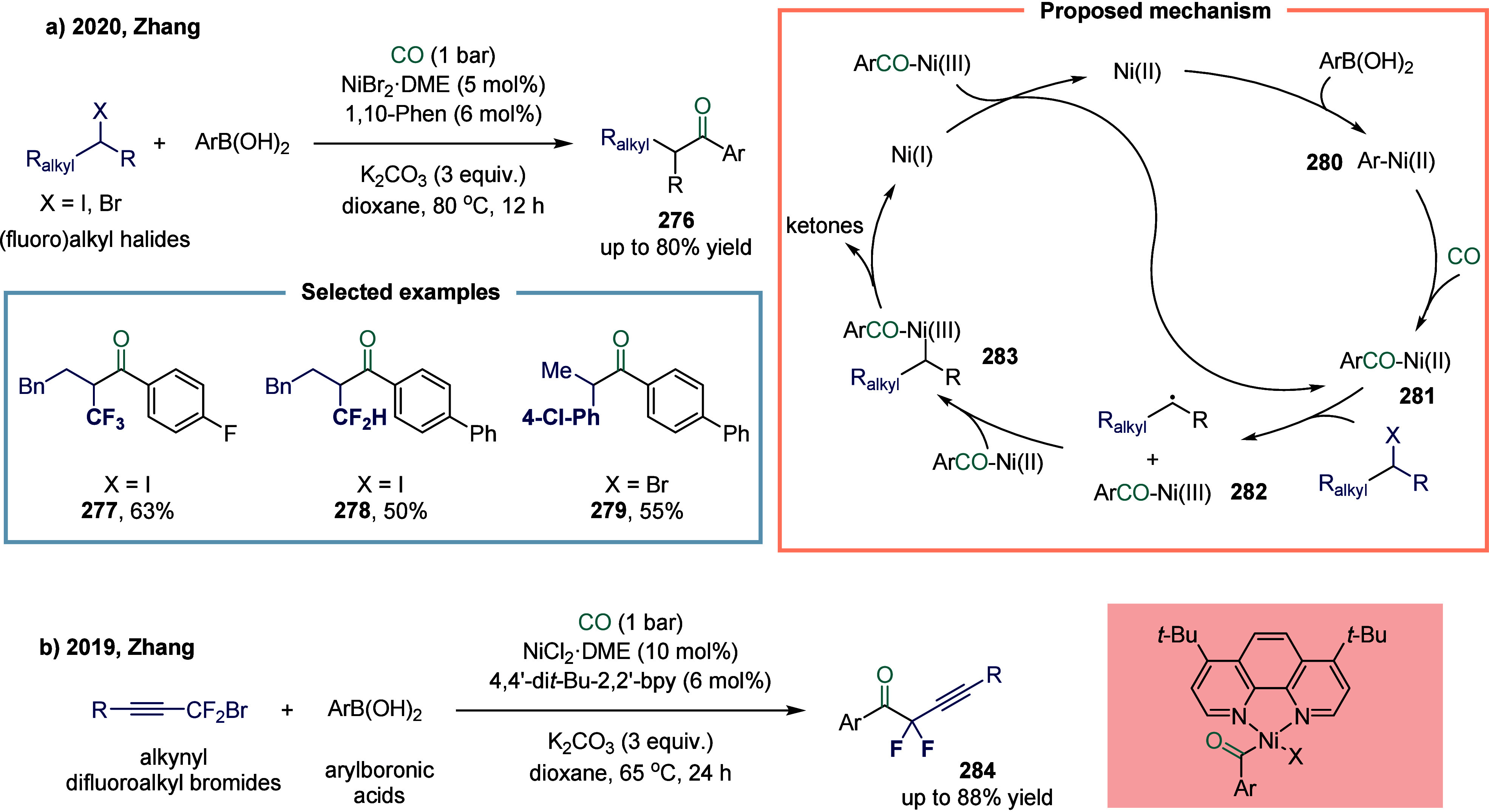
Nickel-Catalyzed Carbonylation of Trifluoromethylated, Difluoromethylated, and Nonfluorinated Aliphatic Electrophiles with Arylboronic Acids

In 2023, Arndtsen and co-workers demonstrated nickel-catalyzed photoinduced carbonylation for the synthesis of acid chlorides from alkyl halides (Scheme 34a).^200^ In this transformation, the combination of high-bite-angle Xantphos ligands with nickel(0) generated a photoactive catalyst system that efficiently promoted the activation of alkyl iodides and activated alkyl bromides for carbonylation under blue light irradiation at ambient temperature. The complex XantphosNi­(CO)_2_ provided a stable, easily handled, and reproducible catalyst for the near-quantitative carbonylative synthesis of acid chlorides. This catalytic system facilitated the reductive elimination of otherwise high-energy acyl chloride intermediates. In contrast to classical nickel-catalyzed carbonylation protocols, where coordination of carbon monoxide typically deactivates the catalyst, the CO-bound nickel species remained catalytically competent in this process. The methodology enabled the synthesis of electrophilic acyl chlorides **285** in up to 97% yield, which could be readily converted into a range of carboxylic acid derivatives, including thioesters **289**, under mild conditions. The successful engagement of nickel(0) complexes in visible-light photoredox catalysis expands the synthetic utility of Group 10 metals in carbonylative cross-coupling chemistry and provides a robust platform for leveraging existing nickel catalysts in diverse and synthetically valuable transformations. Very recently, Arndtsen and co-workers realized XantphosNi­(CO)_2_-catalyzed carbonylation transformation of alkyl halides with sodium azide, affording aliphatic isocyanates **290** in up to 99% yield (Scheme 34b).^201^ Mechanistic investigations indicated that visible-light excitation of a Xantphos-bound nickel catalyst facilitated a radical-mediated carbonylation of alkyl halides, while the CO-bound nickel species promoted the formation of reactive acyl azide intermediates, enabling a rapid Curtius rearrangement. Nevertheless, the involvement of a radical chain mechanism or photochemical pathways mediated by in situ generated Ni­(I) or Ni­(II) species cannot be excluded. Integration of this transformation with subsequent nucleophilic trapping provided a modular and versatile platform for the synthesis of structurally diverse, unsymmetrical ureas **292**, carbamates **293**, and amines **294**.

**Scheme 34:**
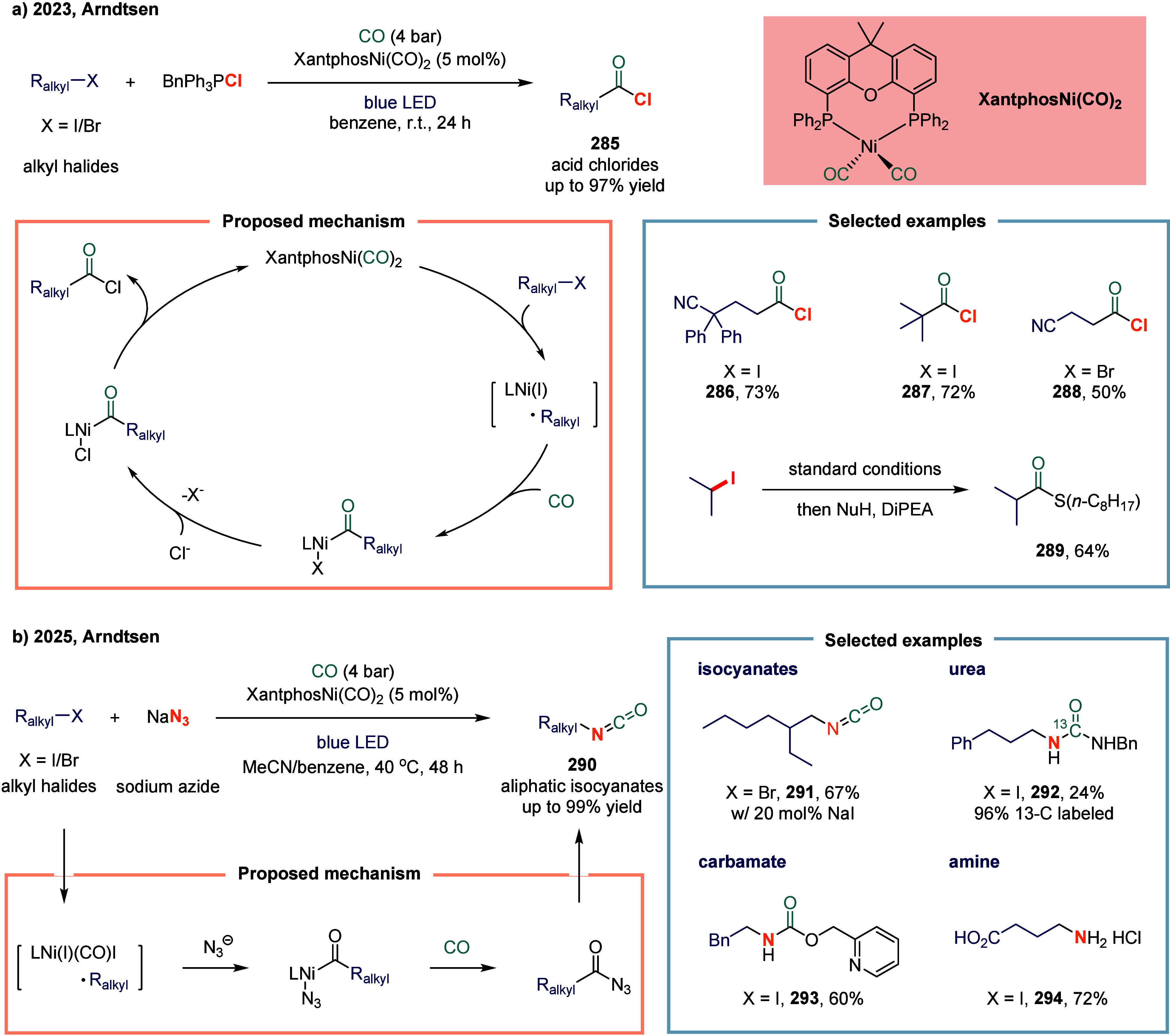
Visible Light Driven Nickel Carbonylation for the Synthesis of Acid Chlorides and Aliphatic Isocyanates

Phosphorus is an essential element for life and plays a vital role in numerous biological processes. Organophosphorus compounds, in particular, are not only fundamental structural components of genetic material but also find broad applications across diverse fields, including medicinal chemistry, agrochemicals, materials science, and organic synthesis.^202–205^ In 2024, Wu and co-workers disclosed the α-C­(sp^3^)-X carbonylation of α-phosphorus-, α-sulfur-, and α-boron-substituted alkyl halides with amines or alcohols using a Nickel/photoredox catalytic system (Scheme 35).^206^ The unique electronic properties of heteroatoms, including electron density distribution, bond dissociation energy, and resonance effects, allow heteroatom-containing substituents to exert a profound influence on the physicochemical properties and reactivity of neighboring molecular sites. In particular, α-heteroatoms can effectively stabilize adjacent carbon-centered radicals, thereby promoting decarbonylation to yield thermodynamically favored radical species, especially under conditions of low carbon monoxide pressure or elevated temperature. Furthermore, the enhanced polarity of carbon–halogen bonds in α-heteroatom-substituted alkyl halides facilitates dehaloprotonation, further modulating their reactivity profiles in catalytic transformations. Utilization of this nickel-based photocatalytic system effectively circumvented the aforementioned challenges and enabled the efficient synthesis of a diverse array of heteroatom-containing phosphine and sulfur carbonyl compounds **296**-**298** with high yields and broad functional group compatibility. However, the incorporation of heteroatom-substituted boron species resulted in unstable intermediates, and no isolable boron-containing products were obtained. Instead, only deboronated acetate derivatives were observed as the major products.^207,208^

**Scheme 35:**
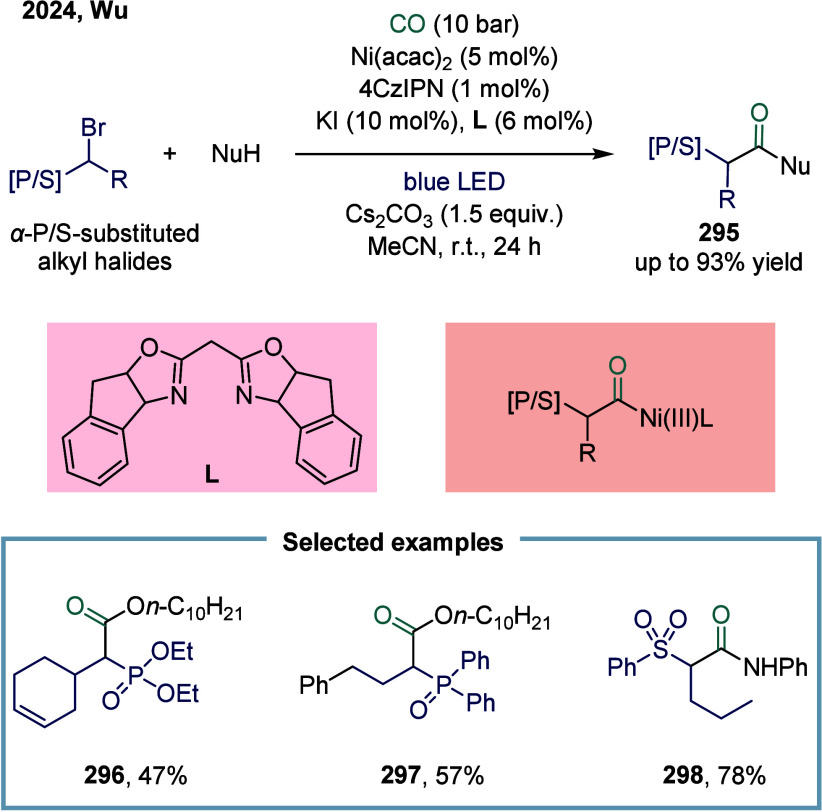
Nickel/Photoredox-Catalyzed Carbonylative Transformations of *α*-Phosphorus- and *α*-Sulfur-Substituted Alkyl Halides

Unsymmetric dialkyl ketones represent a fundamental structural motif prevalent in natural products, pharmaceuticals, and functional materials.^209^ Owing to their broad synthetic utility, they serve as versatile intermediates capable of undergoing diverse chemical transformations. Among the numerous strategies for ketone construction, carbonylative cross-coupling has emerged as a particularly powerful and practical approach. In 2024, Chen and co-workers accomplished a carbonylative synthesis of unsymmetric dialkyl ketones, with NiCl_2_·DME/*NN*_2_-pincer type ligand as the catalytic system (Scheme 36).^210^ The newly developed *NN*_*2*_-pincer ligand proved essential for enabling this transformation. Its key advantage lies in effectively suppressing competing side reactions, including undesired Negishi-type coupling, unfavorable β-hydride elimination, and dehalogenation of the alkyl iodide side chain. As a result, the system facilitates a chemoselective three-component carbonylation reaction with high efficiency. The catalytic cycle was proposed to begin with transmetalation between the Ni­(I) species and organozinc reagent, forming an alkyl-Ni­(I) intermediate **300** that undergoes CO insertion to yield an acyl-Ni­(I) complex **301**. This acyl species then engaged in a SET process with an unactivated alkyl electrophile, generating a high-valent Ni­(III) intermediate **302** that underwent reductive elimination to deliver the unsymmetric dialkyl ketone **299**. However, the potential involvement of an acyl radical species during the process cannot be definitively ruled out. In addition, the reaction exhibited a pronounced substrate limitation, showing high efficiency only with secondary alkyl electrophiles (**303–304** and **306–308**); in contrast, primary halides or pseudohalides afforded only trace amounts of the desired product **305**.

**Scheme 36:**
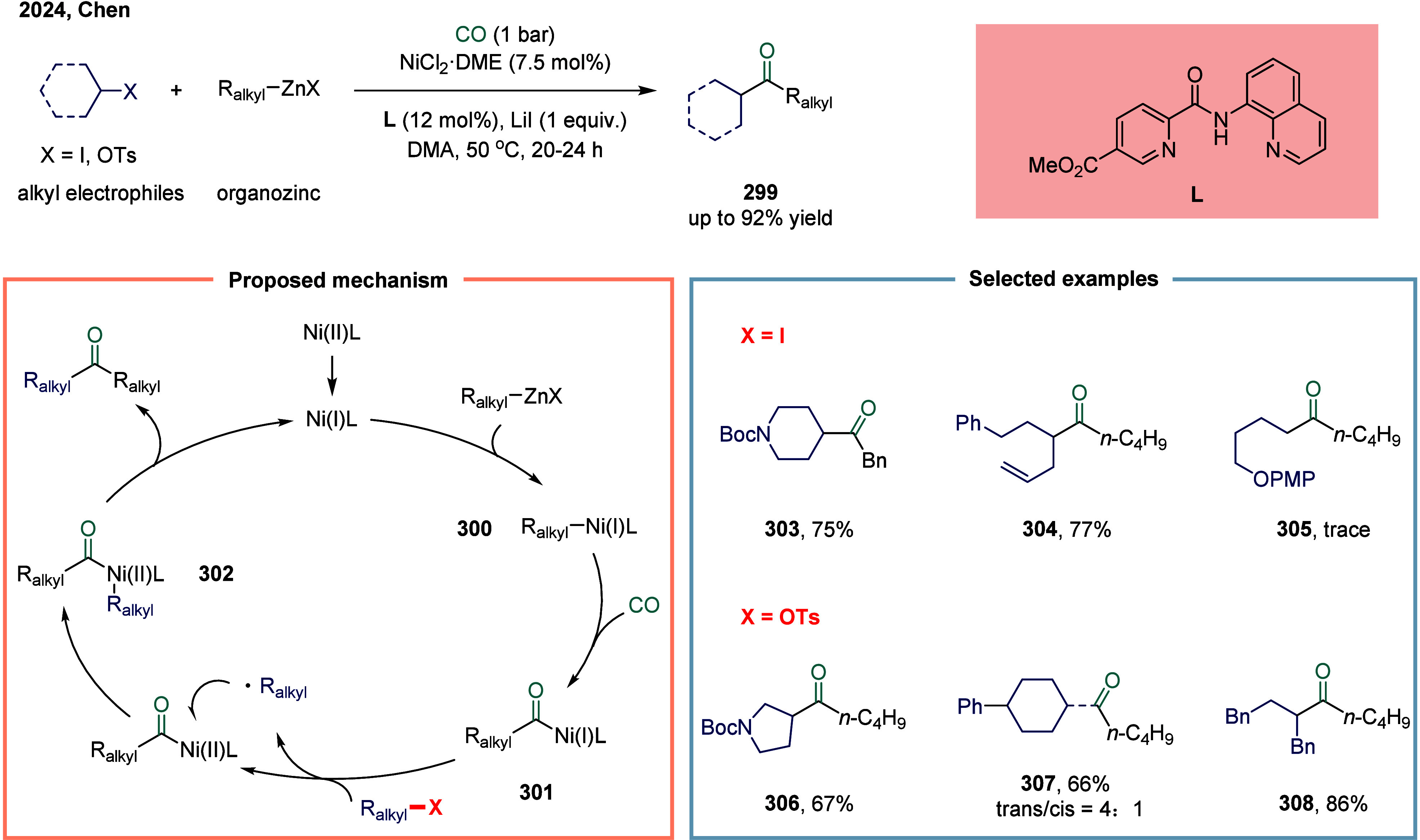
Nickel-Catalyzed Carbonylative Cross-Coupling of Secondary Alkyl Electrophiles and Organozinc Compounds

The enantioselective carbonylative coupling of alkyl halides with nucleophiles represents an ideal strategy for accessing α-chiral centers, which are prevalent in pharmaceutical compounds. Recently, Chu and co-workers reported a conceptually distinct strategy to address this challenge, employing a dual catalytic system that combines photoredox and chiral nickel catalysis, thereby separating reactivity from stereocontrol (Scheme 37).^211^ In this approach, the coupling of benzyl halides with amines afforded a range of chiral amides **309** with excellent enantioselectivity (up to 82% yield, up to 95% ee). Mechanistically, the excited-state Ir­(III)* complex underwent SET process with the amine to generate an aminium radical cation **310** and a reduced Ir­(II) species. The Ir­(II) complex then initiated another SET with the benzyl halide to form a C­(sp^3^) radical **313**. In a parallel catalytic cycle, the chiral Ni catalyst captured the amine-derived radical in the presence of CO to form a stabilized Ni­(I)–carbamoyl complex **312**, thereby preventing undesired side reactions and enabling the enantioselective trapping of the C­(sp^3^) radical to afford the key high-valent Ni­(II) intermediate **313**. A variety of functional groups, such as internal alkyne **314** and bromo substituent **315**, are well tolerated under the reaction conditions. In contrast, α-tertiary alkyl amine afforded the product **317** with significantly lower yield, which is likely attributed to the reduced efficiency of radical coordination to the nickel center due to steric hindrance.

**Scheme 37:**
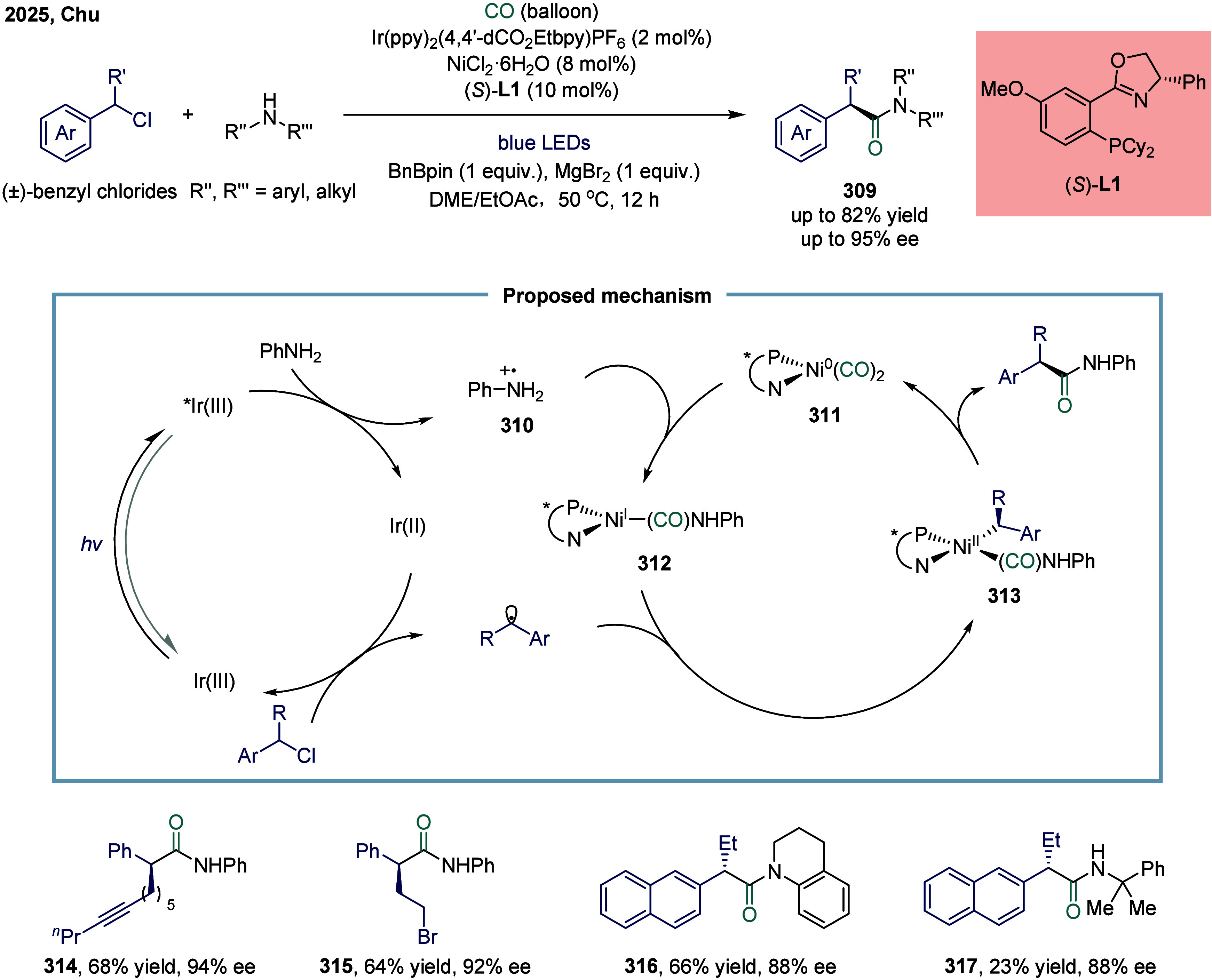
Nickel/Photo-Catalyzed Enantioselective Carbonylative Coupling Reactions of Benzyl Chlorides and Amines

#### 2.6.3 Unsaturated Bonds

In 2020, Zhang and co-workers reported a nickel-catalyzed one-pot cascade reaction that enables the incorporation of carbon monoxide, arylboronic acids, and difluoroalkyl electrophiles across carbon–carbon double bonds, providing a streamlined strategy for the synthesis of structurally diverse ketones **318** (Scheme 38a).^212^ Building on their prior findings that CO could undergo smooth insertion into aryl-Ni­(II) bonds under ambient conditions (1 atm, room temperature) to afford acylnickel­(II) complexes [Ar­(CO)­Ni­(II)],^198,199^ the authors developed a multicomponent carbonylation strategy that effectively suppressed the formation of catalytically inactive Ni­(CO)_4_ and minimized undesired byproducts. In this system, the Ni­(II) catalyst initially formed an aryl-Ni­(II) carbonyl complex [Ar­(CO)­Ni­(II)] **319**, which underwent SET with a difluoroalkyl bromide to generate both a difluoroalkyl radical and a Ni­(III) species [Ar­(CO)­Ni­(III)]. The difluoroalkyl radical subsequently added to the alkene, forming a new carbon-centered radical, which was intercepted by another Ni­(II) complex to afford the key Ni­(III) intermediate [Ar­(CO)­Ni­(III)­(alkyl)] **320**. Notably, the carbonyl group on the alkene-derived fragment was proposed to coordinate with the nickel center, thereby stabilizing the high-valent species. Moreover, the reaction exhibited broad substrate scope, accommodating a wide range of arylboronic acids, alkenes, and alkyl electrophiles, including difluoroalkyl bromides **321, 323, 324**, and bromoacetate **322** derivatives.

**Scheme 38:**
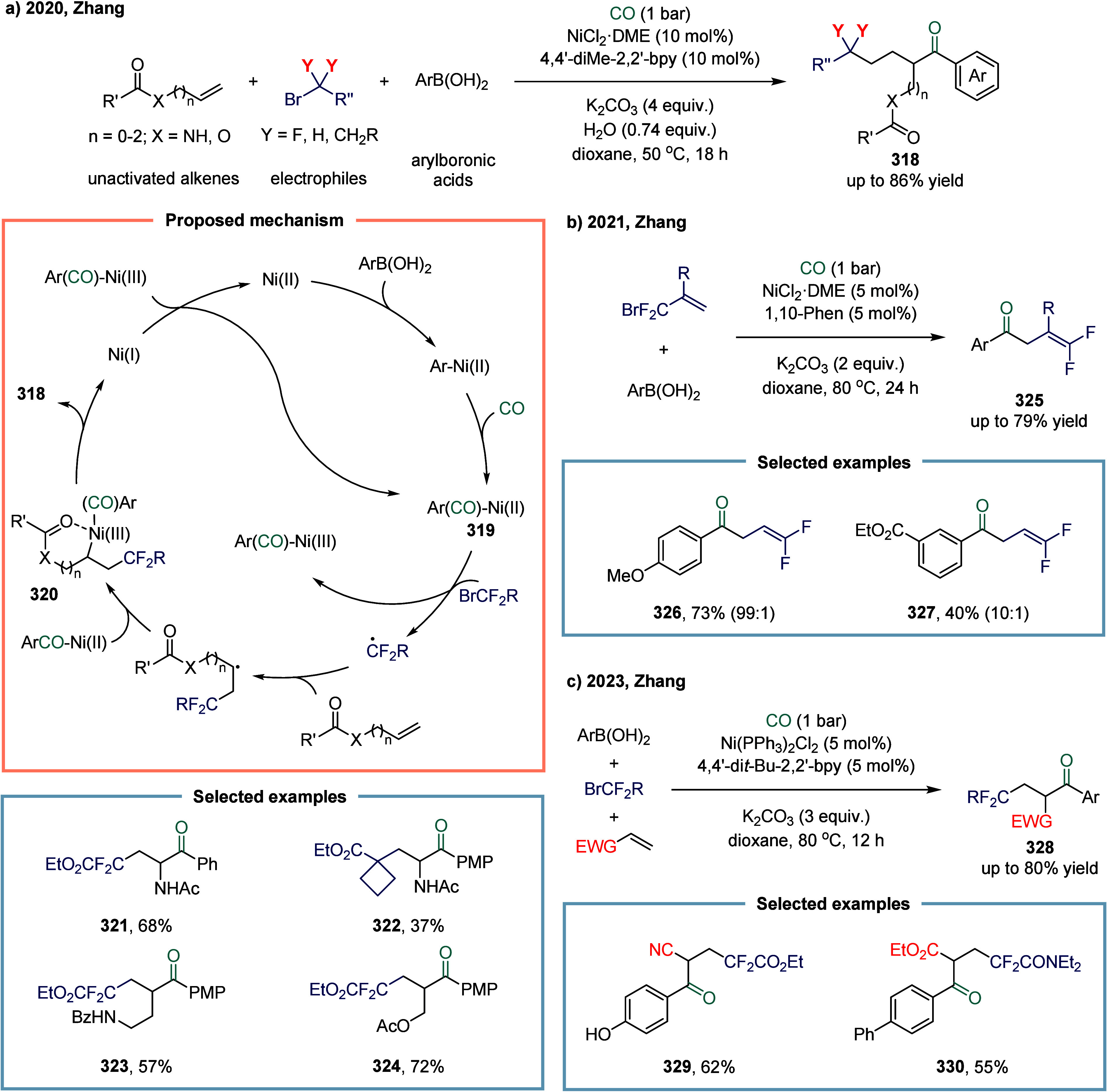
Nickel-Catalyzed Multicomponent Carbocarbonylation of Alkenes, Arylboronic Acids, and Electrophiles

Zhang and co-workers, following up their initial work on nickel-catalyzed carbonylative coupling of alkenes,^212^ reported a highly γ-selective carbonylative arylation of 3-bromo-3,3-difluoropropene (Scheme 38b).^213^ The reaction was conducted under 1 atm of CO using NiCl_2_·DME as the catalyst and 1,10-phenanthroline as the ligand, efficiently affording the γ-selective carbonylation of 3-bromo-3,3-difluoropropene (BDFP) with γ/α selectivity ranging from 6.7:1 to 99:1. A broad range of arylboronic acids, including both electron-rich and electron-deficient variants, proved compatible with the reaction conditions, demonstrating good functional group tolerance (**326** and **327**). While both radical and nonradical pathways are conceivable, current evidence favors a radical-based mechanism. The laboratory of Zhang, likewise, aiming to expand the substrate scope beyond traditional alkenes, focused on the nickel-catalyzed carbonylation of electron-deficient alkenes. In 2023, Zhang and co-workers achieved a nickel-catalyzed multicomponent carbodifluoroalkylation of electron-deficient alkenes (Scheme 38c).^214^ Although acrylonitrile and acrylates are well-established Michael acceptors, the α-electron-withdrawing group-substituted alkyl radicals generated through radical addition are highly prone to reduction, often resulting in undesired hydrogenated byproducts. Despite recent progress in radical difluoroalkylation chemistry, catalytic multicomponent carbodifluoroalkylation of electron-deficient olefins, such as acrylonitrile and acrylates, remains largely underdeveloped.^215^ Arylboronic acids bearing a variety of functional groups exhibited good compatibility with the reaction conditions (**329** and **330**); notably, substrates containing free phenol functionalities underwent the cascade carbonylation smoothly without the formation of phenol esters.

1,3-Enynes are valuable synthetic building blocks that can efficiently serve as radical acceptors, enabling both 1,2- and 1,4-difunctionalization reactions. In a recent report, Guo and co-workers demonstrated a related nickel-catalyzed carbonylative coupling method of 1,3-enynes to access tetra-substituted CF_3_-allenyl ketones **331**, without the use of noble metal catalysts (Scheme 39a).^216^ This elegant strategy combined arylboronic acids, cyclobutanone-derived oxime esters, and CF_3_-enynes in a cascade sequence involving aryl-Ni­(II) formation, CO insertion to generate acyl-nickel­(II) species, SET-induced activation of oxime esters, and radical relay via β-scission and 1,4-addition. A key mechanistic highlight was the direct SET activation of the oxime ester by the acylnickel intermediate, enabling the generation of propargyl radicals, which then engaged in Ni­(III)-mediated coupling to afford allenyl ketones. This work not only expanded the synthetic utility of 1,3-enynes as radical acceptors in multicomponent settings but also demonstrated a rare example of 1,4-difunctionalization in nickel catalysis, overcame long-standing issues such as radical polarity mismatch and β-hydride elimination, and showcased excellent chemoselectivity, broad substrate compatibility, and potential for late-stage derivatization, thus underscored the synthetic value of nickel-enabled multicomponent carbonylation cascades. Moreover, the success of this cascade relied on the effective suppression of undesired three-component coupling and carbonylative side reactions, achieved by fine-tuning the reactivity of the oxime esters via appropriate leaving group selection and optimizing the polarity match between alkyl radicals and 1,3-enynes.

**Scheme 39:**
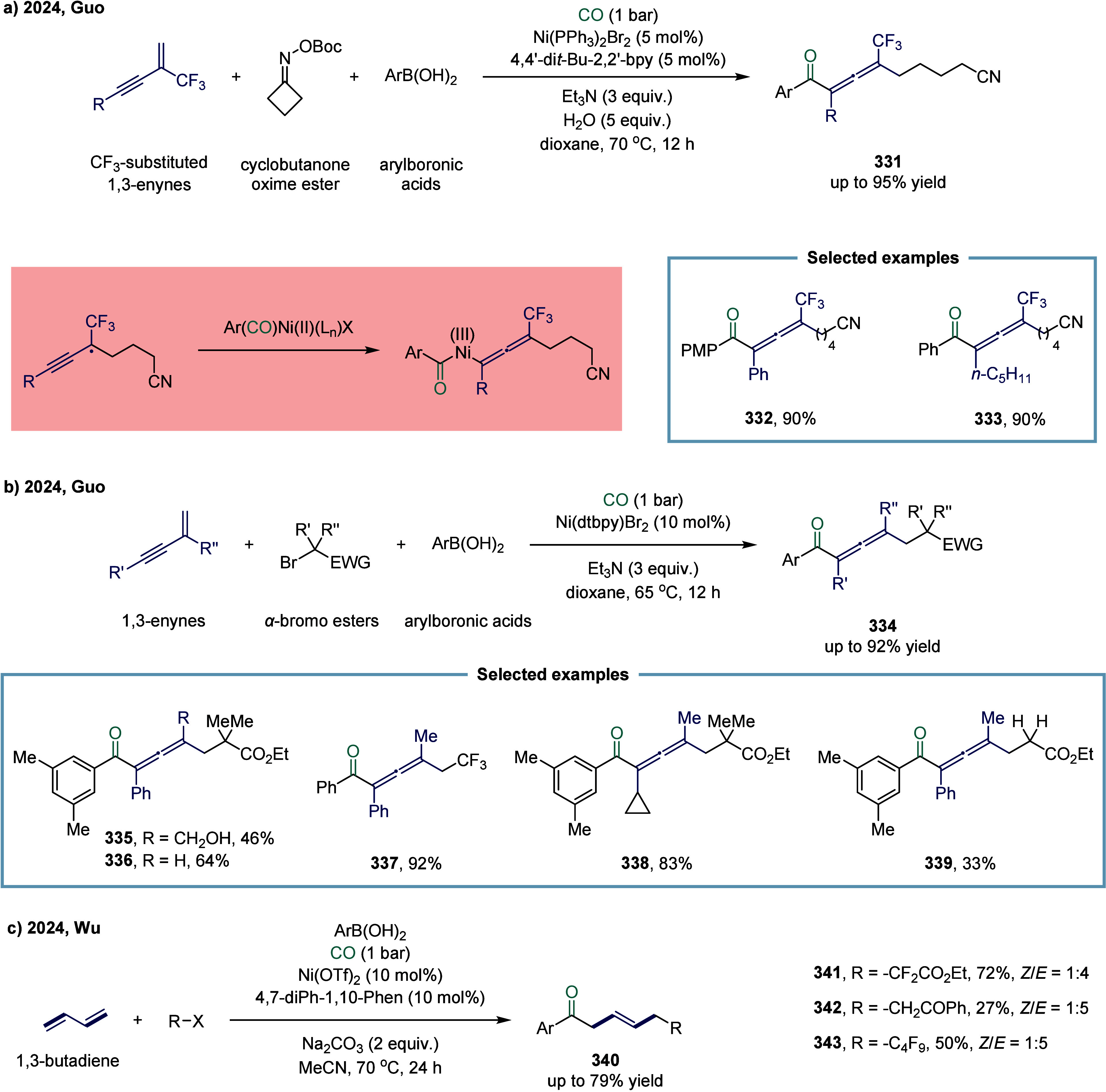
Nickel-Catalyzed Multicomponent Carbonylation of 1,3-Enynes or 1,3-Butadiene

In addition to CF_3_-substituted 1,3-enynes, alkyl-substituted 1,3-enynes also serve as efficient radical acceptors, enabling the transformation of 1,3-enynes and carbon monoxide into tetrasubstituted allenyl ketones. Guo and co-workers applied this strategy to nickel-catalyzed carbonylative four-component 1,4-dicarbofunctionalization of 1,3-enynes with activated alkyl halides and arylboronic acids under atmospheric pressure of CO (Scheme 39b).^217^ By finely tuning the electronic and steric characteristics of alkyl radicals, the developed cascade reaction exhibited broad compatibility with a variety of 1,3-enynes bearing substituents such as hydroxymethyl **335**, hydrogen **336**, and alkyl groups **338**, delivering moderate to excellent yields. This protocol operated under mild conditions, featured an extensive substrate scope, and demonstrated remarkable tolerance toward diverse functional groups. In addition to α-bromoesters, fluoroalkyl iodide **337** was also a viable coupling partner. Mechanistic investigations indicated that the acyl-Ni­(II) intermediate played a critical role in facilitating both the coupling process and the generation of alkyl radicals. Collectively, this work significantly extended and complemented prior nickel-catalyzed methodologies for the synthesis of tetra-substituted CF_3_-allenyl ketones.

1,3-Butadiene, the simplest naturally occurring conjugated diene, is an inexpensive and readily available carbon feedstock derived from petroleum cracking.^218^ Consequently, 1,3-butadiene serves as a valuable surrogate for the synthesis of allyl-containing compounds.^219,220^ In 2024, Wu and co-workers successfully developed a nickel-catalyzed four-component carbonylation of 1,3-butadiene, enabling efficient access to *β,γ*-unsaturated ketones **340** (Scheme 39c).^221^ This strategy provided a sustainable alternative for the production of such valuable compounds. A wide range of radical precursors participated smoothly in the transformation, including 2-bromo-2,2-difluoroacetate **341**, nonfluorinated substrate **342**, and perfluoroalkyl iodide **343**. The protocol featured a low-cost catalytic system, high step economy, mild reaction conditions, and excellent 1,4-regioselectivity. Notably, it represented a significant advance in applying 1,3-butadiene as a carbon synthon in carbonylation chemistry.

In 2024, Liang and colleagues reported a novel nickel-catalyzed three-component carbonylation reaction that enables the sequential formation of C­(sp^3^)-N and C­(sp^3^)-C­(sp^2^) bonds, thereby facilitating the efficient synthesis of acyl-substituted pyrroline derivatives (Scheme 40).^222^ The key mechanism involved the regioselective 5-exo-trig cyclization of imine radical **345**, which generated a new alkyl radical **346**. A broad spectrum of arylboronic acids and *γ,δ*-unsaturated oxime esters has been recognized as efficient reactants, facilitating the synthesis of a diverse array of pyrroline derivatives **347–349** with good yields.

**Scheme 40:**
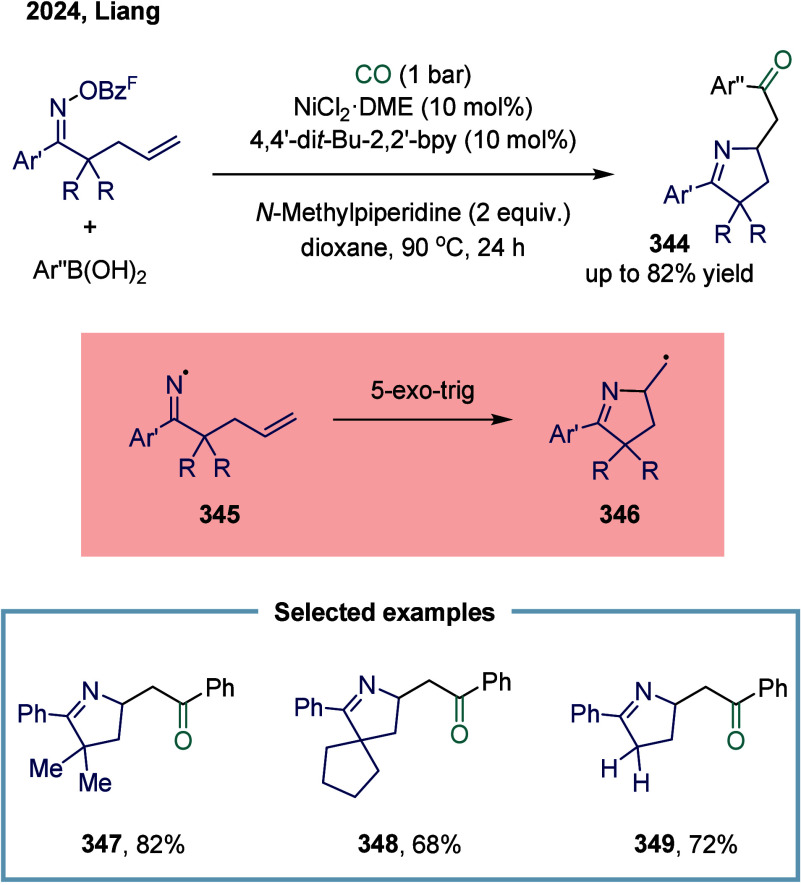
Nickel-Catalyzed Narasaka-Heck Cyclization Carbonylation of Unsaturated Oxime Esters with Arylboronic Acids

Aryl-nickel species, generated via the transmetalation of arylboronic acids with metallic nickel, have been widely employed as key intermediates in the carbonylative coupling of unsaturated bonds, wherein carbon monoxide inserts into the aryl-Ni­(II) bond. Besides, Wu and co-workers developed a nickel-catalyzed radical-relay carbonylation of alkenes with ethers (Scheme 41).^223^ The reaction enabled the concurrent formation of carbon–carbon or carbon-heteroatom bonds and carbonylation across double bonds, providing an efficient and modular strategy for synthesizing γ-substituted carbonyl compounds **350**. A notable feature of this work was its operation under low carbon monoxide pressure (1 atm), which improved safety and practicality, particularly relevant to both laboratory and industrial applications. The method demonstrated broad substrate scope and excellent functional group tolerance, accommodating a variety of alcohols, phenols, and amines. Various alkenes, including the less reactive ethylene, were successfully converted under the reaction conditions. However, the formation of asymmetric ethers exhibited low regioselectivity, attributed to differences in ether C–H bond dissociation energies and the relative rates of alkene addition (**355–356**). The authors compellingly demonstrated the synthetic utility of the method through the one-step synthesis of Naftidrofuryl, a clinically approved drug used to treat cerebrovascular disease (CVD).^224,225^ This was particularly significant given that the conventional synthesis of Naftidrofuryl required 5 steps, whereas the new protocol afforded the target molecule in a 46% yield via a single transformation. This highlighted the method’s potential to streamline pharmaceutical synthesis and minimize resource consumption.

**Scheme 41:**
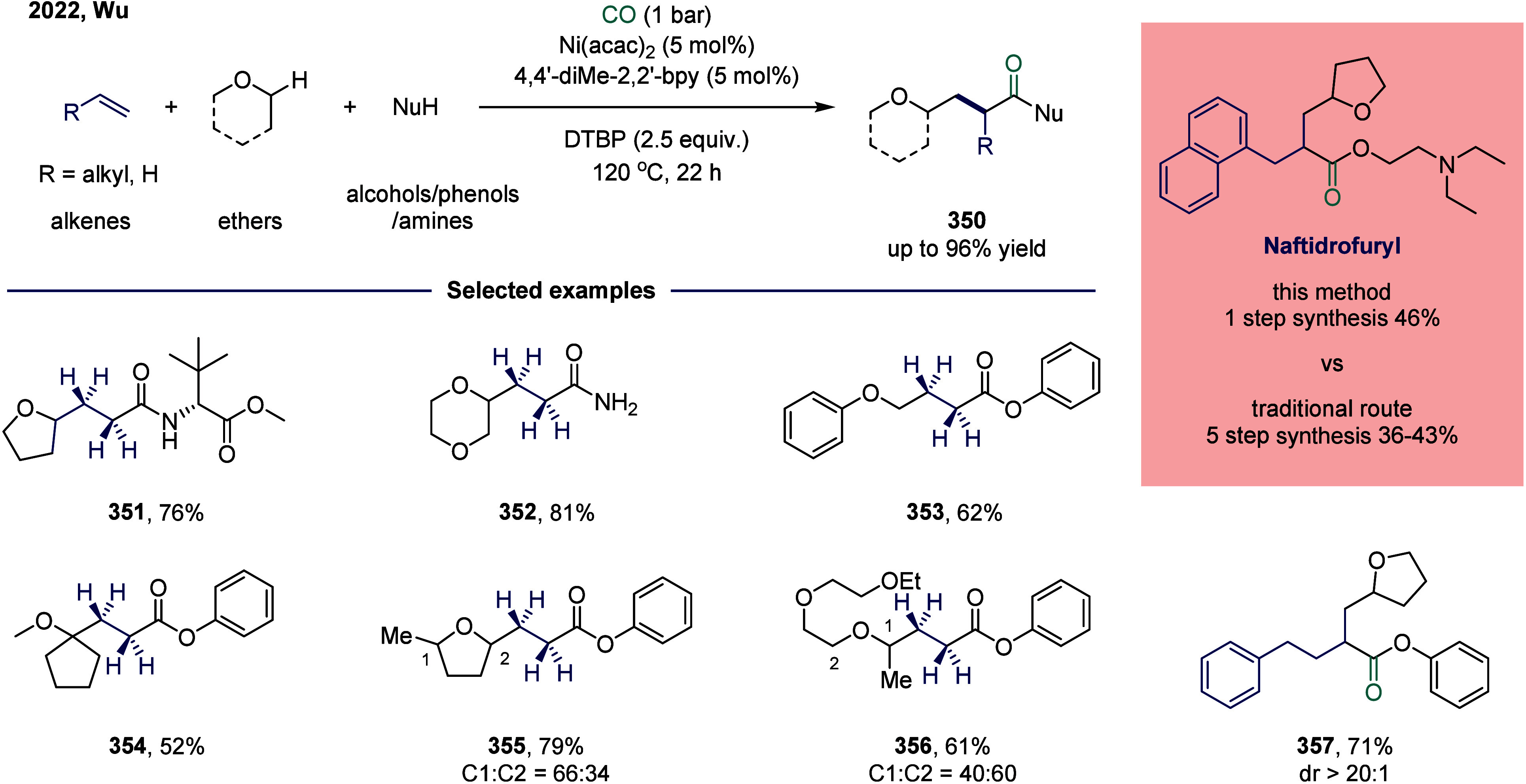
Nickel-Catalyzed Four-Component Carbonylation of Ethers and Alkenes

Recently, Shu and co-workers reported a nickel-catalyzed asymmetric cross-coupling strategy for the enantioselective synthesis of α-N-heteroaryl ketones from alkenes and enamines in the presence of a carbon monoxide surrogate.^226^ The success of this transformation relies on the differentiation of two distinct alkenes as well as precise control over both regioselectivity and enantioselectivity. This reductive oxidative carbonylation, using a CO surrogate, enables the enantioenriched assembly of α-N-heteroaryl ketones from two distinct olefins under gas-free and operationally simple conditions. Subsequently, the same group developed a nickel-catalyzed adaptive migratory asymmetric hydroacylation using chloroformates as CO sources, allowing for the synthesis of enantioenriched α-aryl ketones.^227^ In this reaction, a single alkene undergoes adaptive migration, serving as the precursor for two distinct alkylmetal intermediates, thereby providing a highly direct route to enantioenriched α-aryl ketones.

In 2021, Chu and co-workers reported a nickel-catalyzed, metal photoredox-enabled strategy for the selective and divergent aminocarbonylation of alkynes under 1 atm of CO (Scheme 42).^228^ This protocol enabled a Markovnikov-selective hydroaminocarbonylation of alkynes to afford *α,β*-unsaturated amides **358**, and also accommodates a sequential four-component hydroaminocarbonylation/radical alkylation in the presence of alkylboronates, providing direct access to structurally diverse amides **359**. Mechanistically, aniline underwent SET oxidation by the excited-state photocatalyst to generate an anilinium radical cation, which then underwent deprotonation to yield the aniline radical **360**. This radical could be trapped by CO to form a carbamoyl radical. The resulting carbamoyl radical could be intercepted by Ni­(I) species, forming a Ni­(II)-carbamoyl complex **361**. Alternatively, the aniline radical **360** may first coordinate to Ni­(I), followed by CO insertion to yield the same intermediate **361**. This Ni complex underwent Markovnikov-selective migratory insertion into the alkyne to generate a vinyl-nickel species **362**, which then underwent protodemetalation to furnish the hydroaminocarbonylation product **358**. In addition, under basic conditions, another SET event between the excited photocatalyst and the alkylboronate generated an alkyl radical, which reacts with the Ni-intermediate to deliver the alkylated product **359**. Notably, sensitive functional groups such as bromo and hydroxy substituents were tolerated, affording products **363** and **364** in 64% and 62% yield, respectively. When 1,2-diphenylacetylene, an internal alkyne, was employed, the corresponding trisubstituted acrylamide **365** was obtained in 30% yield.

**Scheme 42:**
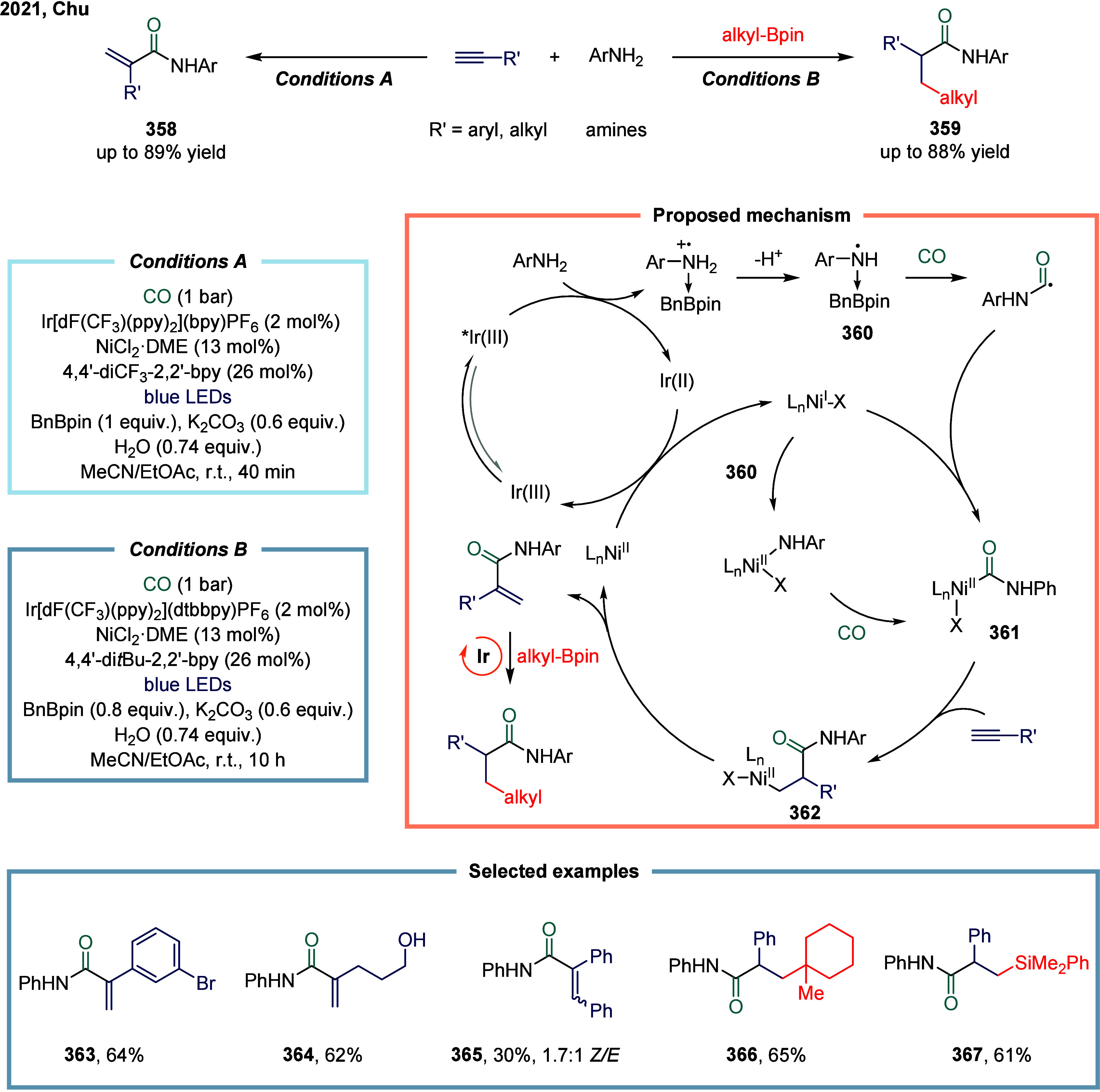
Divergent Aminocarbonylations of Alkynes Enabled by Photoredox/Nickel Dual Catalysis

#### 2.6.4 Others

The C–N bond is among the most prevalent chemical linkages, widely found in numerous organic compounds and naturally occurring biomacromolecules.^229,230^ The direct insertion of small molecules or unsaturated bonds into the C–N bond is highly appealing, particularly for the late-stage functionalization of structurally complex molecules.^231^ In this regard, the insertion of carbon monoxide (CO) into the C–N bond of amines via C–N bond activation has emerged as a highly efficient and straightforward strategy for the synthesis of valuable amide derivatives (Scheme 43).^232^ Mechanistic studies indicated that the amine-I_2_ charge transfer (CT) complex **369** was initially formed, which subsequently underwent oxidative addition to the active Ni(0) center, generating the radical-containing Ni­(I) complex **370** through a radical pathway. Subsequently, carbon monoxide (CO) migrated and inserted into the complex, resulting in the release of I_2_. This reaction offered a straightforward and efficient approach to the synthesis of arylacetamides from benzylamines in the presence of catalytic amounts of I_2_ and a nickel catalyst at 140 °C for 18 h. Various tertiary benzylamines were well-suited substrates for this transformation. In contrast, α-methyl-substituted benzylamines afforded the product **372** in only 15% yield, whereas primary benzylamines **374** yielded only trace amounts of product.

**Scheme 43:**
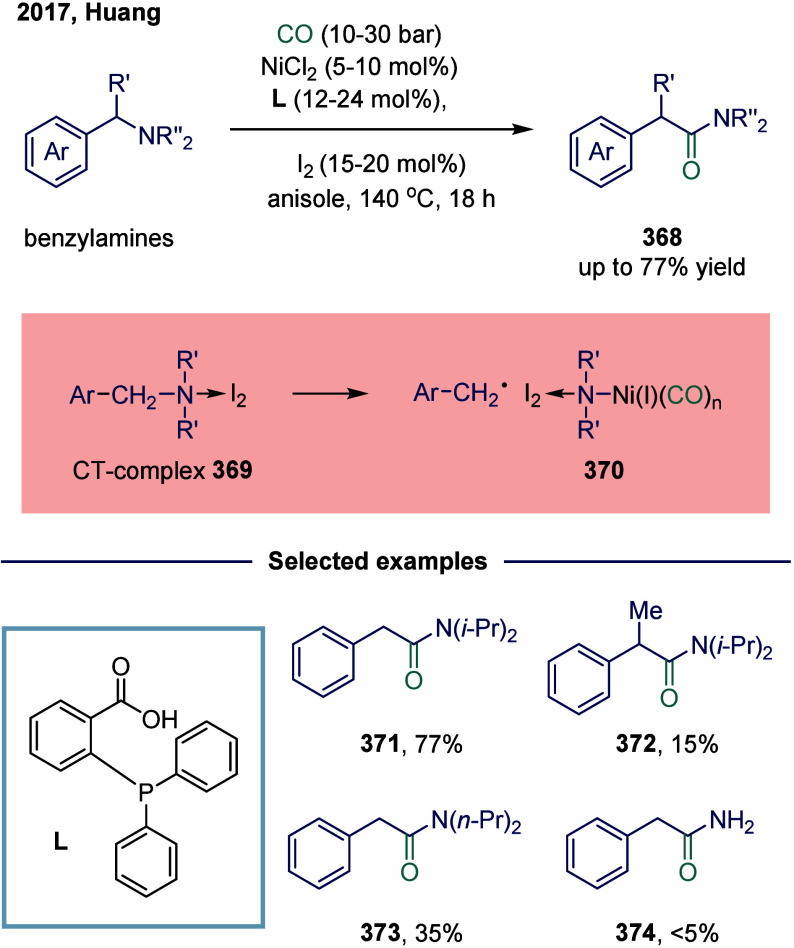
Charge-Transfer Complex Promoted C–N Bond Activation for Nickel-Catalyzed Carbonylation

In 2024, Shi and co-workers reported a nickel-catalyzed electroreductive cross-electrophile carbonylation strategy from readily available epoxides, aryl halides, and ClCO_2_Pr under mild conditions (Scheme 44).^233^ This methodology offers a concise and practical route to value-added *β-/γ*-hydroxy ketones from readily available epoxides, which serve as versatile electrophiles derived from abundant feedstock chemicals.^234–238^ Mechanistically, the Ni­(II) precatalyst was electrochemically reduced at the cathode to generate Ni­(I) **376**, which underwent oxidative addition with ClCO_2_Pr to form a Ni­(III) intermediate. Subsequent reductions and decarbonylation furnished the active Ni(0) species **377**. Oxidative addition of aryl iodide to Ni(0) gave aryl–Ni­(II) **378**, followed by CO insertion to afford acyl–Ni­(II) intermediate **379**. Electroreduction of **379** yielded Ni­(I) **380**, which reacted with epoxide 1 and TMSI to generate the TMSO-substituted alkyl radical **382**. This radical was intercepted by acyl-Ni­(II) (**381**) to form Ni­(III) species **383**, which underwent reductive elimination and hydrolysis to deliver the desired hydroxy ketone product **375**, while regenerating Ni­(I). Only trace amounts of product were obtained with unsymmetrical epoxide **387**, while aryl epoxide **388** failed to afford any carbonylated products under various conditions.

**Scheme 44:**
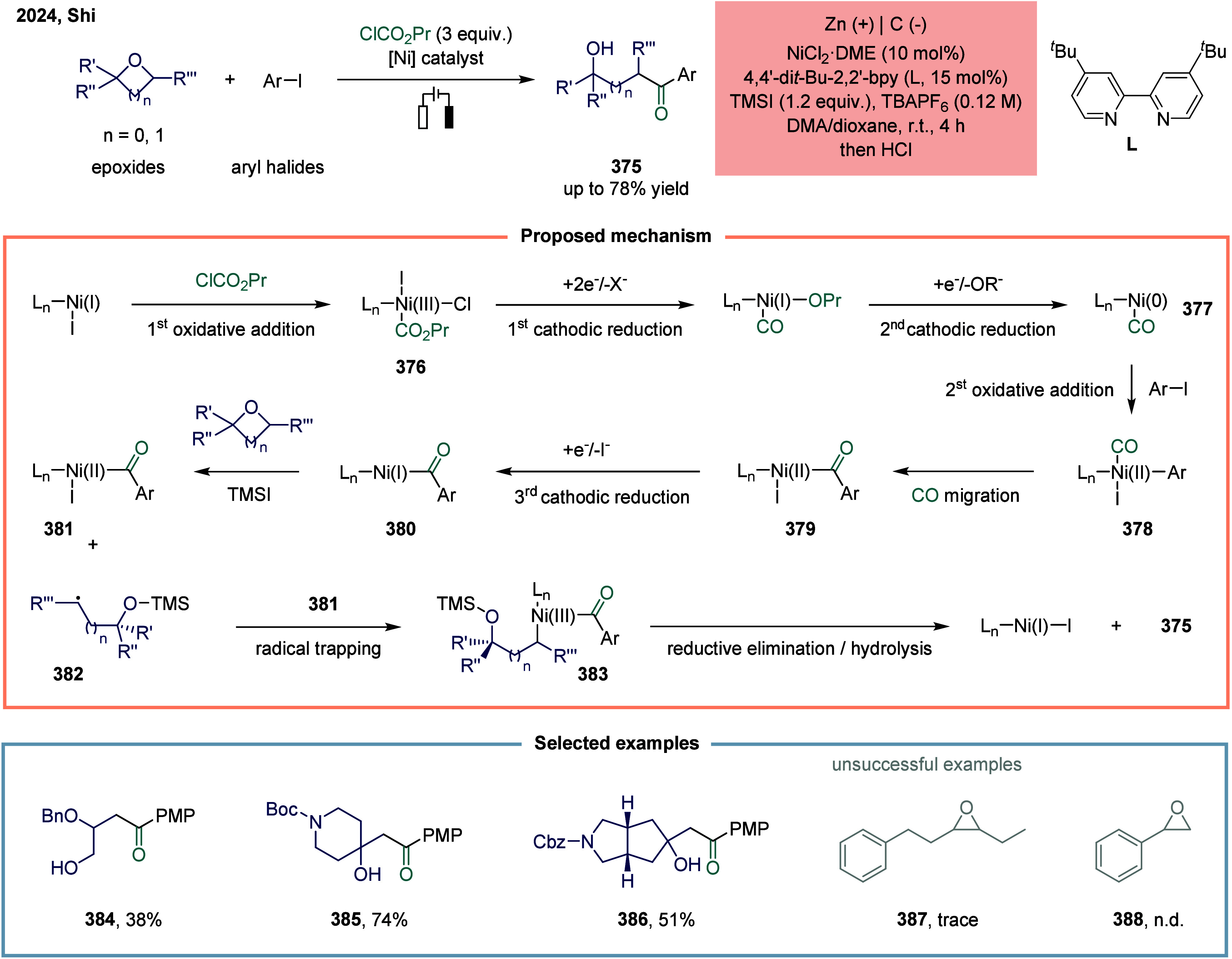
Nickel-Catalyzed Electroreductive Cross-Electrophile Carbonylation to *β*/*γ*-Hydroxy Ketones

Recently, Wu and Qi collaboratively explored a novel nickel-catalyzed carbonylation reaction for the synthesis of β-aminoketones from aryl N-tosylaziridines and arylboronic acids (Scheme 45).^239^ Using formic acid as a CO surrogate, a wide range of β-aminoketones were synthesized in moderate to excellent yields. This method features high regioselectivity, broad functional group tolerance, and avoids the direct handling of CO gas. In the presence of KI, aryl N-tosylaziridines undergo ring-opening to afford β-iodosulfonamide intermediate. Subsequently, an aryl-Ni­(I) species engages in a SET process with aryl *N*-tosylaziridine to generate radical intermediate **390**. Coordination and insertion of CO into intermediate II affords the acyl–Ni­(II) species III, which then undergoes radical addition with intermediate to deliver intermediate **391**. This strategy offers a valuable complement to existing nickel-catalyzed radical carbonylation approaches for aziridine ring-opening transformations.

**Scheme 45:**
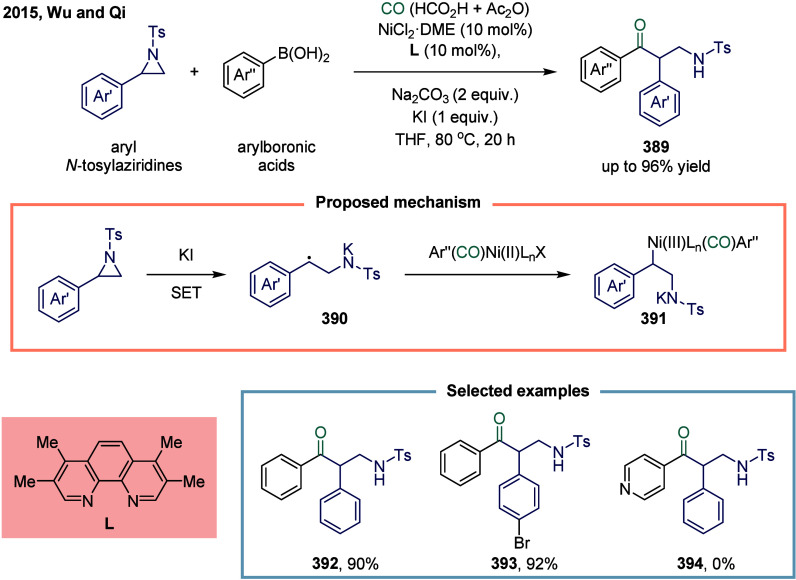
Nickel-Catalyzed Carbonylation Reaction of Aryl *N*-Tosylaziridines with Arylboronic Acids

### 2.7 Copper-Catalyzed System

Copper complexes are widely employed as catalysts in organic transformations owing to their accessible oxidation states, typically ranging from 0 to +3, which facilitate new bond formations through either single-electron pathways or two-electron transfer mechanisms involving organometallic intermediates.^240–251^ For example, low-valent copper can reduce electrophilic reagents to generate single-electron species. Conversely, copper­(II) species, serving as effective single-electron oxidants, can oxidize radicals via an inner-sphere mechanism to form high-valent copper­(III) intermediates-a process that proceeds without interference from carbon monoxide.^242^ Due to copper’s natural abundance in the Earth’s crust, low toxicity, cost-effectiveness, and outstanding catalytic performance in single-electron processes, copper-catalyzed carbonylation reactions have experienced significant advancements over the past few decades.^243^

#### 2.7.1 Carbon–Hydrogen Bonds

C–H carbonylation offers an efficient strategy for streamlining synthetic routes by eliminating the need for substrate preactivation and enabling the direct formation of carbonyl-containing compounds in fewer steps. In contrast to the elegant σ-bond metathesis mechanism for C–H activation, the use of a single-electron species to induce C–H bond cleavage is defined as SET-mediated C–H activation, and thus the carbonylation transformation involving single-electron transfer from a C–H substrate is referred to as SET-mediated C–H carbonylation.^244–247^ In 2016, Wu’s group reported a study on the interactions between copper complexes and alkyl radicals, initiated by hydrogen atom abstraction from C–H compounds using DTBP, followed by CO capture as an approach to C­(sp^3^)-H carbonylation at 120 °C (Scheme 46a).^248^ Mechanistically, the reaction is initiated by copper­(I) mediated or thermal homolytic cleavage of DTBP to give a *tert*-butoxy radical, which reacts with alkanes via a hydrogen atom transfer (HAT) process. The resulting alkyl radical undergoes a single-electron oxidation with copper to afford the corresponding copper­(III)-alkyl intermediate **405**; subsequently, a nucleophilic attack occurs, followed by a further carbon monoxide insertion. The vast majority of reported carbonylation reactions predominantly yield esters, amides, and ketones. In contrast, the use of amides as nucleophiles to access imides has received limited attention, primarily due to their inherently low nucleophilicity.^249,250^

**Scheme 46:**
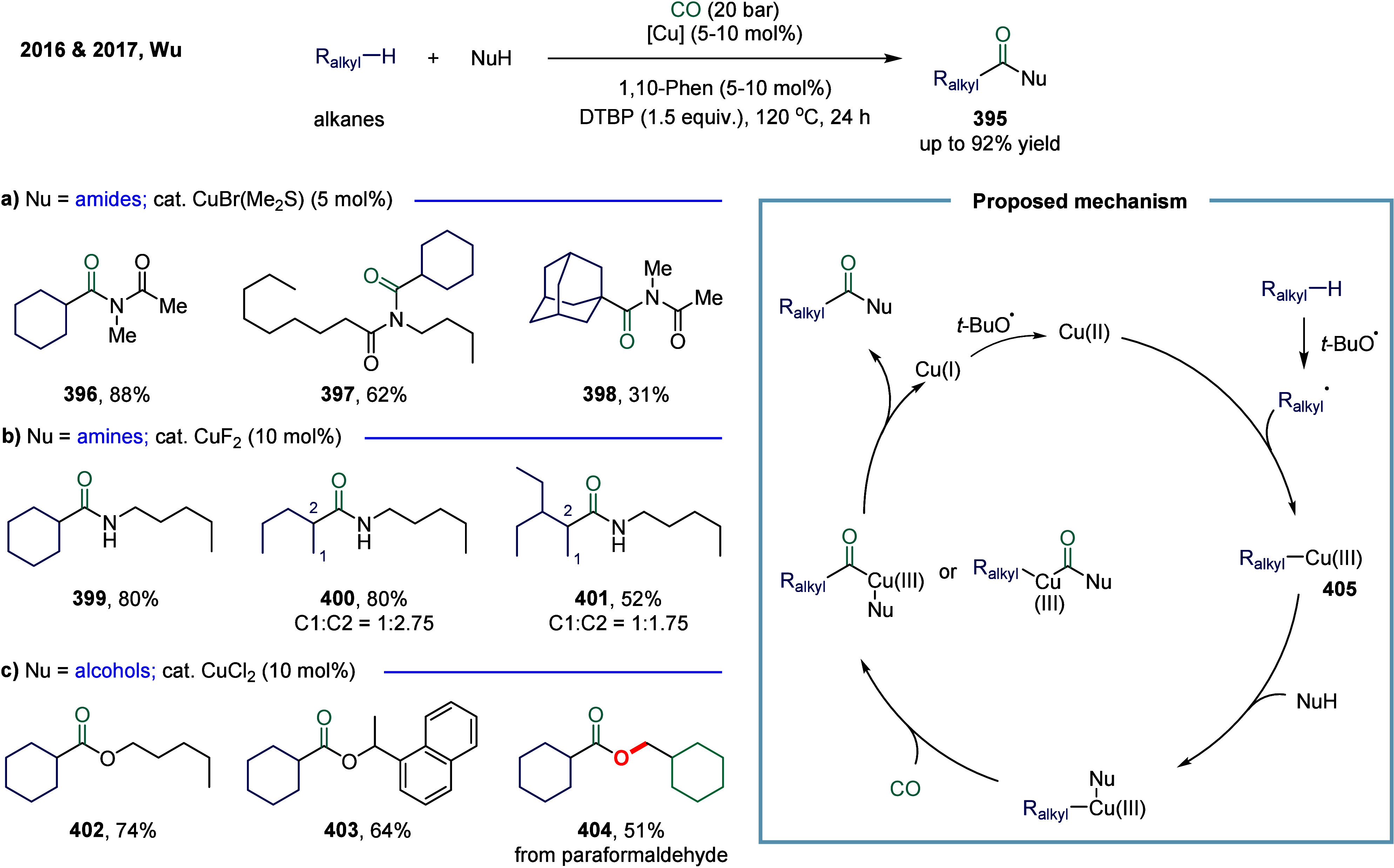
Copper-Catalyzed Carbonylative Coupling of Alkanes

In the same year, Wu’s group demonstrated that this catalytic system is also capable of generating amides using amines as nucleophiles (Scheme 46b).^251^ Several features of regioselectivity were found to differ from those observed in conventional C–H carbonylation reactions. For instance, in the case of 3-ethylpentane **401**, selective carbonylation at the primary and secondary C–H bonds was favored over the tertiary position. Although bond dissociation energies (BDEs) indicate that the tertiary C–H bond has the lowest bond energy (*tertiar*y C–H = 94.9 kcal/mol *vs secondary* C–H = 95.6 kcal/mol and *primary* C–H = 99.2 kcal/mol), the combination of steric hindrance and radical stability renders tertiary carbon radicals unfavorable for carbonylation reactions.^252–254^ In the following year, Wu’s group demonstrated the applicability of alcohols in this reaction, achieving esters in high yields. They also validated the practicality of employing paraformaldehyde as an in situ source of alcohols for this transformation (Scheme 46c).^255^

In 2019, Wu and co-workers reported a copper-catalyzed intra- and intermolecular carbonylative transformation of remote C­(sp^3^)-H bonds in *N*-fluorosulfonamides, offering an efficient and selective approach to the synthesis of δ-lactams **406** (Scheme 47).^256^ This transformation harnesses the unique reactivity of *N*-fluorosulfonamides to generate amidyl radicals under mild conditions. The key step involves a 1,5-hydrogen atom transfer (1,5-HAT), enabling remote functionalization of inert aliphatic C–H bonds, which are typically unreactive under standard conditions. Following HAT, the resulting carbon-centered radical **410** undergoes efficient carbonylation in the presence of carbon monoxide to furnish valuable heterocyclic δ-lactams or esters with high regioselectivity. This protocol demonstrates a powerful strategy for the selective functionalization of unactivated C–H bonds, contributing to the development of radical-mediated C–H activation and carbonylation methodologies.

**Scheme 47:**
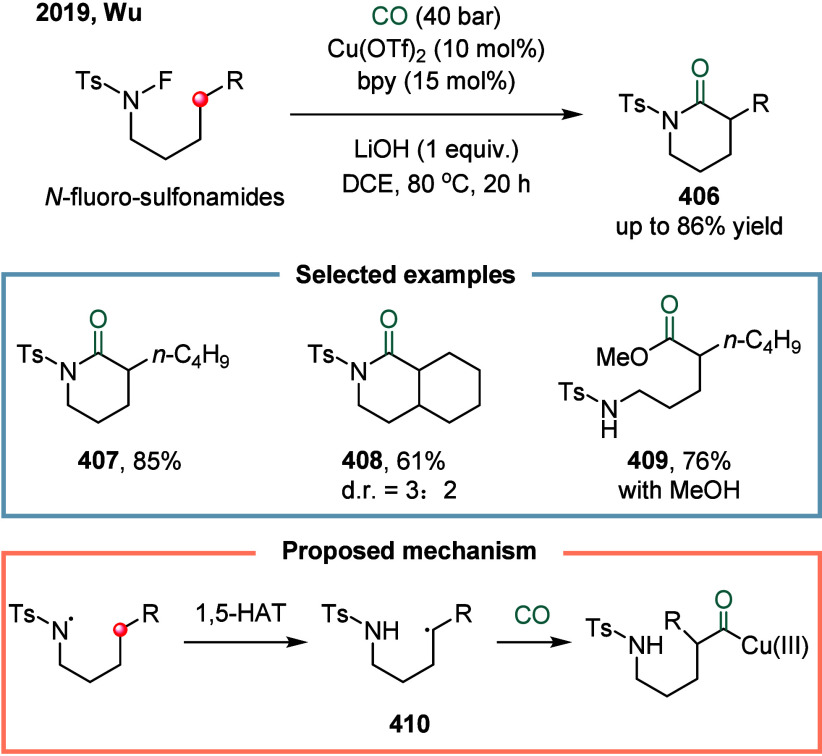
Copper-Catalyzed Intra- and Intermolecular Carbonylative Transformation of Remote C­(sp^3^)-H Bonds in *N*-Fluoro-sulfonamide

Lei and co-workers then reported an approach to deliver alkyl α-ketoamides through the C­(sp^3^)-H carbonylation of cycloalkanes with amines under 60 bar CO atmosphere (Scheme 48).^127^ Initially, monocarbonylation was observed as the major pathway, with only 8% yield of the double-carbonylation product **412** detected. Upon switching the catalyst to CuI, the ligand to 2,9-dimethyl-1,10-phenanthroline (**L2**), and increasing the CO pressure, the reaction afforded the desired alkyl α-ketoamide **412** in 50% yield. A variety of amines, including alkyl amines **413**, aryl amines **414**, and secondary amines **415**, proved to be competent coupling partners. However, with respect to C–H substrates, only a few common cycloalkanes were described, likely due to the challenges associated with the similar reactivity of C–H bonds in comparable chemical environments.^257–259^ In this transformation, the *tert*-butoxy radical is proposed to act as a hydrogen atom transfer (HAT) reagent, generating alkyl radicals from alkanes. Under high CO pressure, a reversible equilibrium is established among the alkyl radical, the corresponding acyl radical, and the α-ketoacyl radical. The acyl radical undergoes CO insertion and subsequently coordinates with intermediate copper­(I) to afford intermediate **416**, an alkyl α-ketoacyl-copper­(II) species. Alternatively, intermediate **417** may also form via direct capture of the α-ketoacyl radical by copper­(I). This intermediate then couples with the piperidyl *N*-centered radical to furnish the double-carbonylation products **412**. Additionally, the authors demonstrated that the combination of copper­(I) and 2,9-dimethyl-1,10-phenanthroline, although seemingly inert, exhibited unique reactivity. Unlike most metal–ligand pairs, CuI and this ligand preferentially formed the alkyl α-ketoacyl-copper­(II) intermediate, which stabilized the alkyl α-ketoacyl radical and facilitated the formation of the double-carbonylation products.

**Scheme 48:**
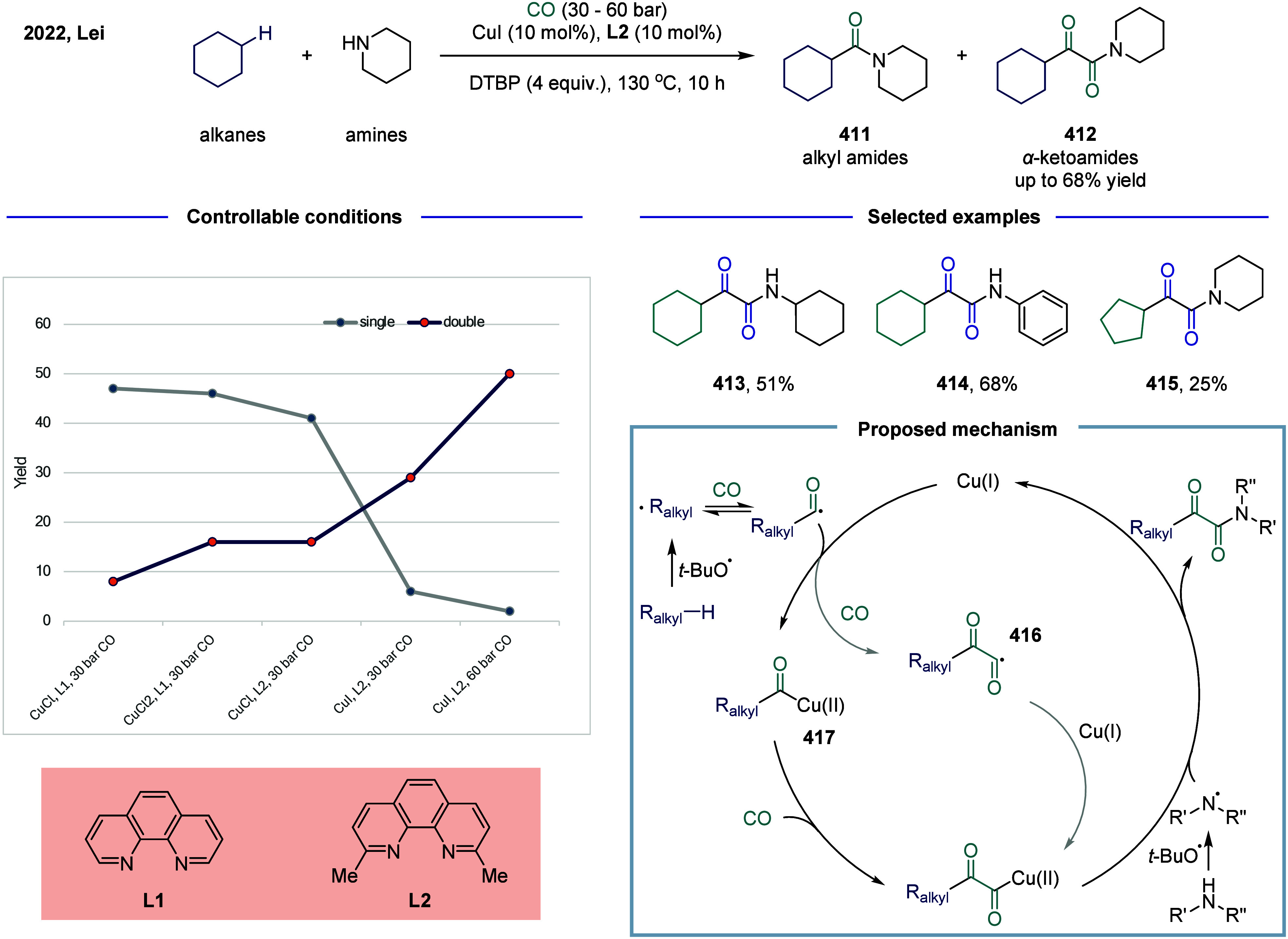
Copper-Catalyzed Double-Carbonylation of Alkanes

Due to the absence of general strategies, most protocols afford only a single type of carbonyl compound, such as esters or amides. Building on this precedent, Wu and co-workers further expanded the scope of copper-catalyzed photoinduced carbonylation of C­(sp^3^)-H bonds to encompass a variety of functionalized alkanes and diverse coupling partners (Scheme 49).^260^ This general and practical photochemical strategy that enables the efficient synthesis of a wide range of carbonyl products **418** under unified conditions. This method avoids extensive optimization, tolerates diverse coupling partners, and utilizes abundant aliphatic C–H bonds, offering a complementary and versatile tool for modern carbonyl synthesis. Ketones **419**, ethers **420**, nitriles **423**, silanes **424**, and even halogen-containing compounds **421** and **422** (Cl, Br) were found to be competent C–H substrates, with no α-site carbonylation products observed. In contrast to other radical-mediated C­(sp^3^)-H functionalizations of functionalized alkanes, where the hydrogen atom transfer (HAT) process typically occurs at α–C-H or tertiary C–H sites,^261,262^ this method demonstrated no preferential carbonylation at these positions. In addition, this copper photocatalytic strategy enables C­(sp^3^)-H carbonylation with a variety of coupling partners, affording synthetically valuable thioesters **429**, amides **430**, selenic esters **428**, ketones **425–427**, acyl halides **433**, and other derivatives in moderate to good yields. The reaction is initiated by photoexcitation of the CuCl_2_ catalyst, generating the excited species. This excited-state copper complex underwent an intramolecular ligand-to-metal charge transfer (LMCT) process to produce a highly reactive chlorine radical and a reduced CuCl species. The chlorine radical got promptly engaged in a HAT with the C–H substrate, yielding a key carbon-centered radical intermediate. In the presence of carbon monoxide, this intermediate rapidly equilibrates with the corresponding acyl radical. Finally, various radical acceptors trap the acyl radical to furnish the desired carbonylated products.

**Scheme 49:**
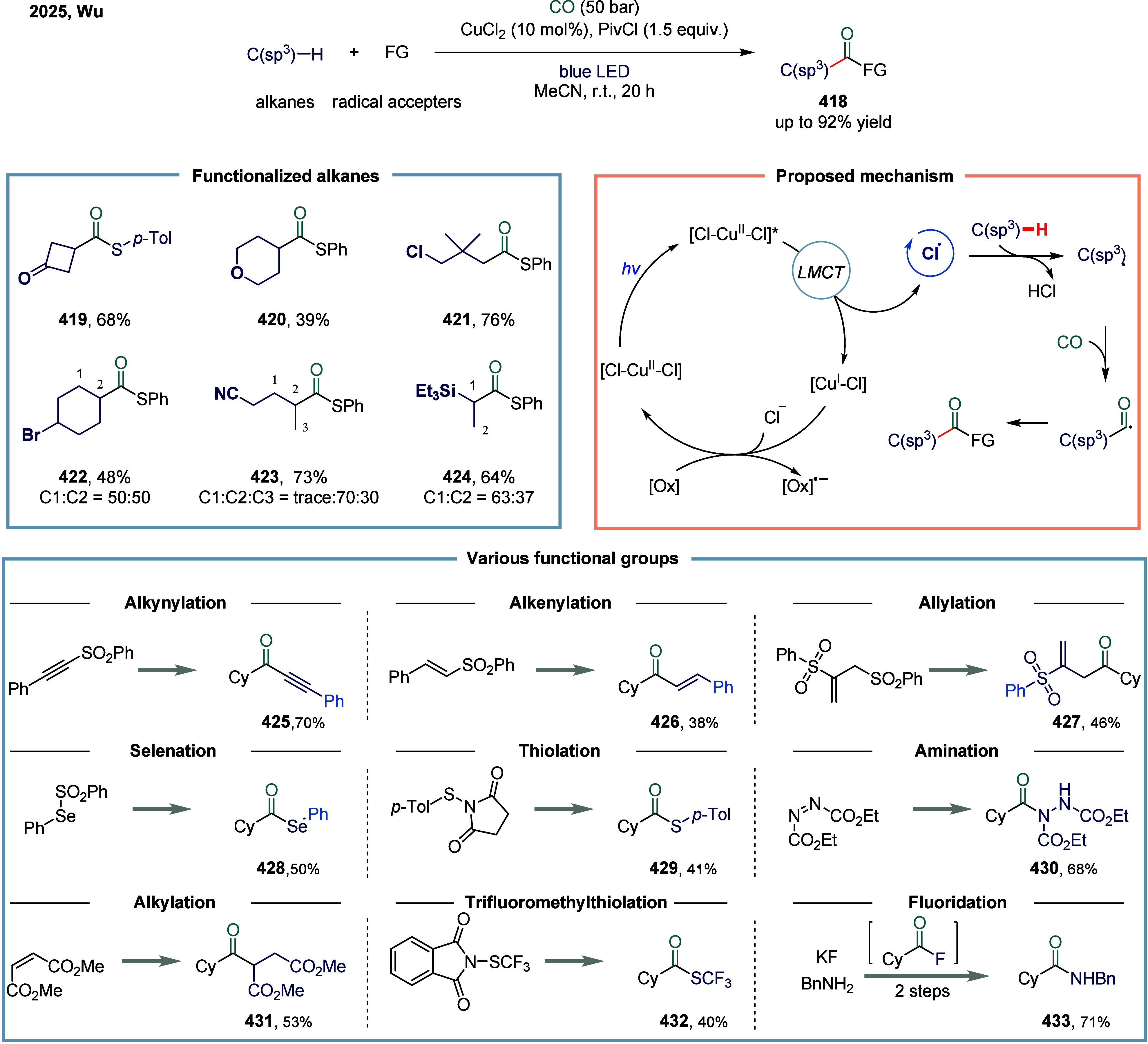
Copper-Catalyzed Photo-Induced C­(sp^3^)-H Carbonylation to Access Various Carbonyl Products

Moreover, the Wu group developed a copper-catalyzed photoinduced carbonylation of C1–C3 gaseous alkanes (Scheme 50).^263^ Although a variety of liquid alkanes have been successfully utilized as standard substrates in carbonylation reactions, the carbonylation of gaseous alkanes remains a long-standing challenge due to several intrinsic factors. One major obstacle is their inherently low reactivity, which arises from the high bond dissociation energies (BDE = 99–105 kcal/mol) of C–H bonds in gaseous alkanes.^264^ Moreover, the limited gas–liquid mass transfer of gaseous reagents in organic solvents significantly hampers effective collisions with catalysts and coupling partners. These difficulties are further compounded when multiple gaseous components are involved, as competitive solubility and the heterogeneous distribution of gas-phase species can adversely affect the reaction efficiency and selectivity.^265^ To solve this serious problem, Wu and collaborators investigated a copper-catalyzed photoinduced C­(sp^3^)-H carbonylation of methane, ethane, and propane with sulfinate salts for converting C1–C3 gaseous alkanes into high value-added acetic acid, propionic acid, or butyric acid derivatives **435–437**. Importantly, the direct carbonylation of ethane represents a promising and economically advantageous strategy for the synthesis of methyl methacrylate (MMA). This transformation is enabled by a copper-catalyzed system, wherein chlorine radicals generated through a ligand-to-metal charge transfer (LMCT) mechanism promote the efficient activation of gaseous alkanes under mild conditions with excellent atom economy.

**Scheme 50:**
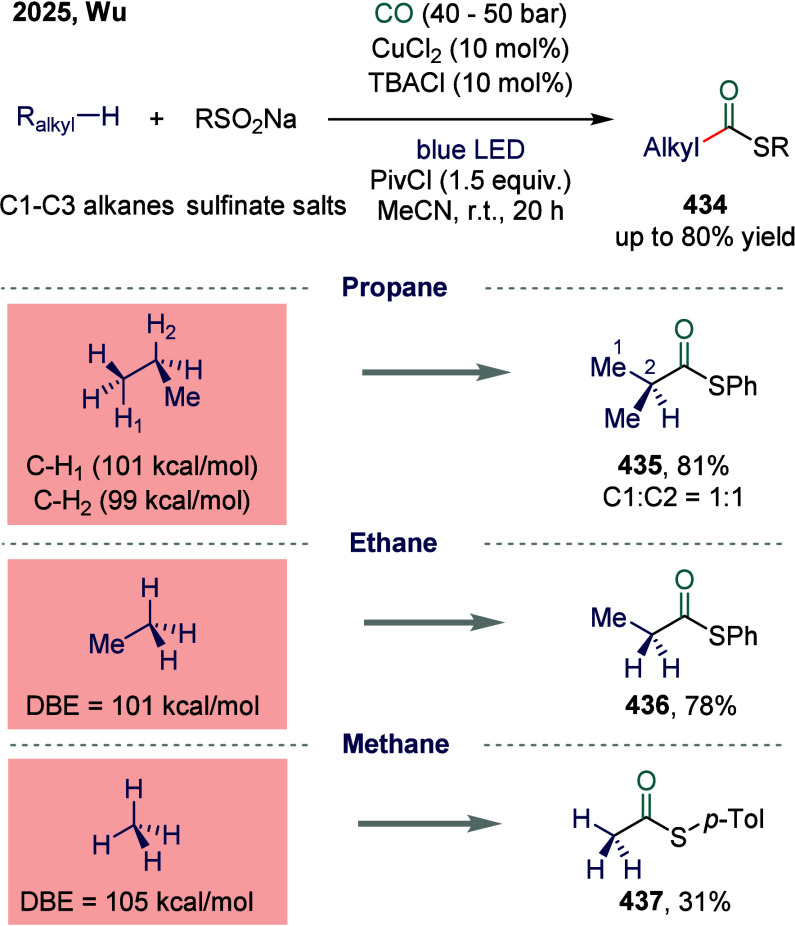
Copper-Catalyzed Photo-Induced Carbonylation of C1–C3 Gaseous Alkanes

#### 2.7.2 Carbon–Halogen Bonds

Direct conversion of organic halides into acyl derivatives offers a versatile and programmable strategy to access reactive synthetic intermediates for subsequent chemical transformations. The carbonylation of organic halides has long attracted significant research interest. Despite significant advances, carbonylation of C­(sp^3^)-X bonds, especially in unactivated alkyl halides, remains more challenging than that of aryl halides due to competing pathways such as oxidative addition and β-elimination. Benefiting from continuous efforts in the organic chemistry community-including pioneering work by Heck, Alper, Ryu, and others-as well as recent comprehensive studies by Arndtsen, Mankad, and our research group, various transition-metal-catalyzed and photoinduced single-electron transfer (SET) strategies for the carbonylation of alkyl halides have been successfully realized.

In 2019, Gong and co-workers described copper-catalyzed and indium-mediated methoxycarbonylation of unactivated alkyl iodides with balloon CO (Scheme 51).^266^ Initial mechanistic studies revealed the participation of alkyl radicals, while a cooperative interplay among Cu, In, and CO was identified as critical for promoting the carbonylation reaction. This work constitutes the first demonstration of methoxycarbonylation of tertiary alkyl iodides to construct quaternary carbon centers, using carbon monoxide as the carbonylation source. Notably, for tertiary alkyl iodides, the study examined only methanol as the nucleophile, while a broader investigation of nucleophilic partners was conducted exclusively with secondary iodides. The generation of an alkyl radical can be achieved via single-electron reduction or iodine abstraction by In­(Cu)_*x*_(CO)_*y*_, resulting in the formation of In­(Cu)_m_(CO)_n_I. Then, the carbonylation of the alkyl radical may proceed through its interaction with In­(Cu) _*x*_(CO)_*y*_ or In­(Cu)_m_(CO)_n_I, leading to the formation of acyl-In­(Cu) intermediates. Metholysis of these intermediates affords the corresponding esters, with concomitant regeneration of copper­(I) species.

**Scheme 51:**
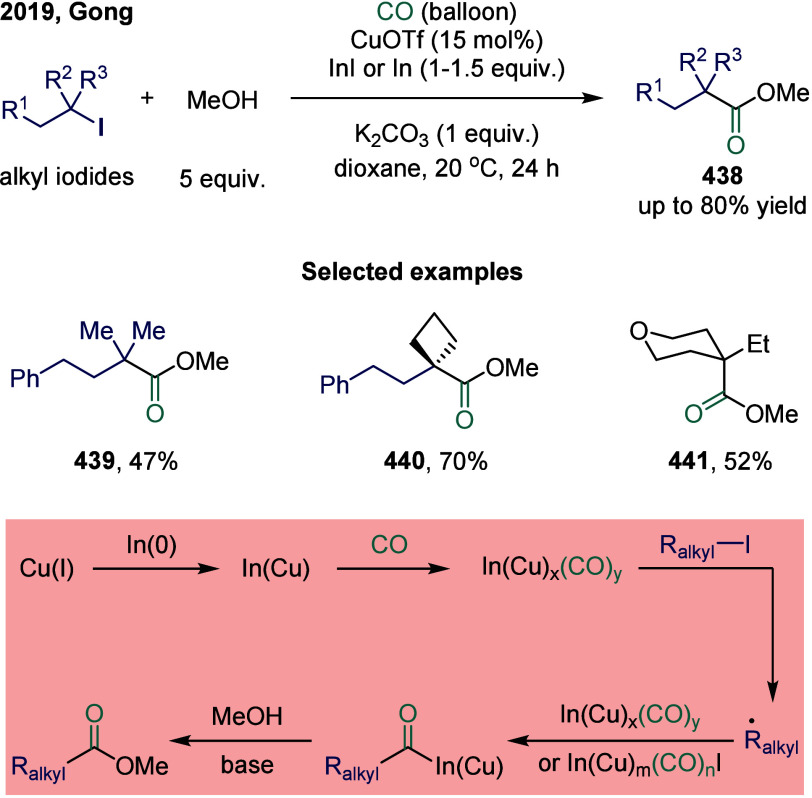
Copper-Catalyzed and Indium-Mediated Methoxycarbonylation of Unactivated Alkyl Iodides

An efficient copper-catalyzed strategy for the carbonylation of alkyl iodides via a SET mechanism was developed by Wu and co-workers (Scheme 52a).^267^ In this reaction, no additional additives were required for the activation of alkyl iodides, and phenols were demonstrated to be suitable coupling partners, affording the desired products **443–445** with excellent yields. In 2022, Evano and co-workers developed a straightforward catalytic system consisting of copper­(I) chloride and *N,N,N’,N”,N”*-pentamethyldiethylenetriamine (PMDTA) to promote the aminocarbonylation of alkyl iodides with amines for the synthesis of amides **446** in up to 97% yield (Scheme 52b).^268^ It was further investigated that alkyl iodide compounds can be carbonylative transformed by using a copper catalyst through a single-electron reduction process. Further mechanistic investigations revealed that the carbonylation of alkyl iodides proceeds via a single-electron reduction pathway mediated by the copper catalyst.

**Scheme 52:**
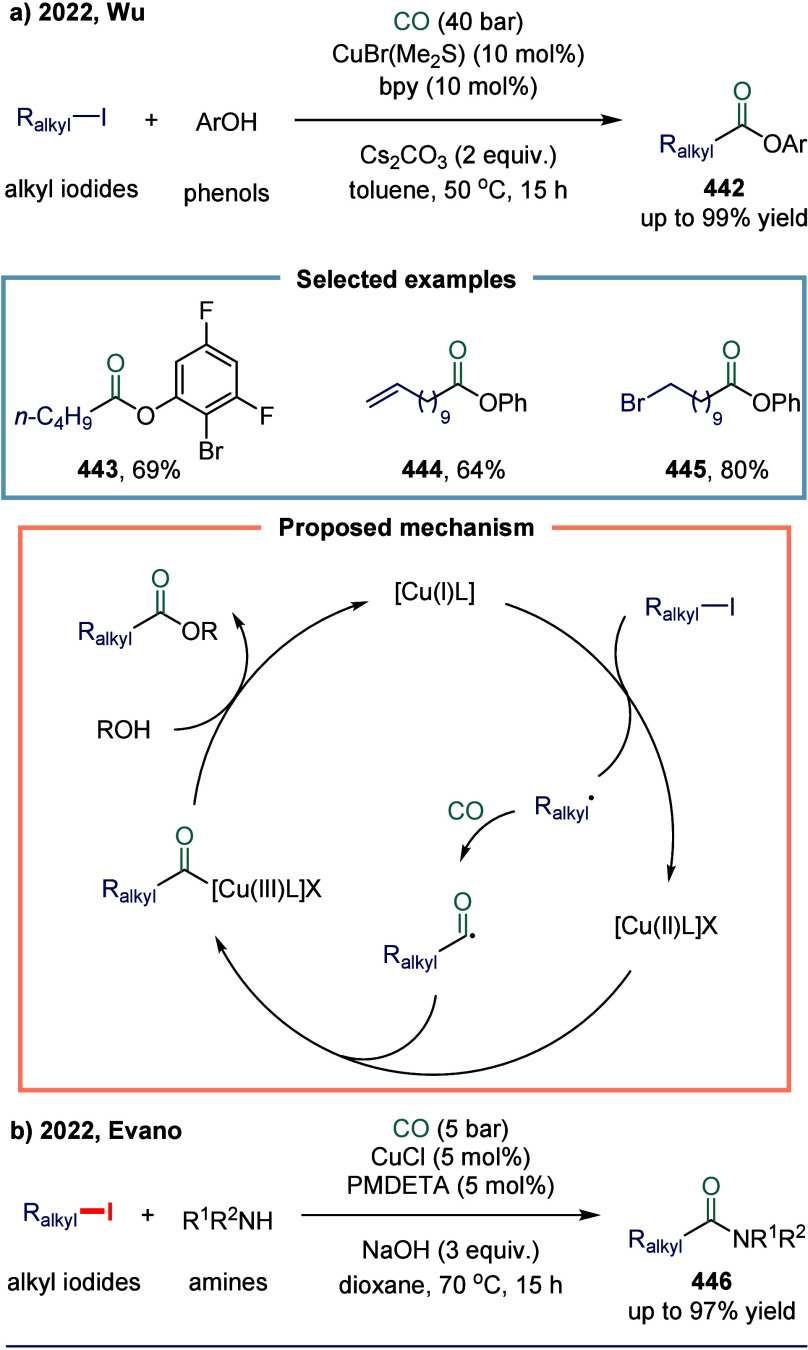
Copper-Catalyzed Carbonylative Coupling of Alkyl Iodides

Mankad and co-workers developed a photochemically driven copper-catalyzed carbonylation of alkyl halides, enabling the synthesis of aliphatic anhydrides **447** in up to 99% yield (Scheme 53).^269^ This transformation utilizes readily available copper salts and abundant bases to generate a heterogeneous Cu^0^ photocatalyst *in situ*. It exhibits excellent efficiency and selectivity even on a scale-up and proceeds via a SET mechanism featuring several advantageous attributes. This work establishes a foundation for the development of efficient and sustainable bulk processes for the production of commodity anhydrides, which are valuable intermediates in the chemical industry and are broadly employed in the synthesis of polymers, pharmaceuticals, and other fine chemicals.^270–272^ A variety of unactivated alkyl iodides, alkyl bromides, and alkyl tosylates were efficiently converted into the corresponding anhydrides **448–450** in good to nearly quantitative yields in the presence of a NaI additive. Upon photoexcitation, the heterogeneous Cu catalyst generated an excited state **451** capable of reducing alkyl halides via a SET process, yielding an iodine-adsorbed intermediate and an alkyl radical. Carbonylation of alkyl radical formed the corresponding acyl radical, which recombined with **452** to give intermediate **453**, bearing coadsorbed acyl and iodine groups. Subsequent C­(O)-I coupling afforded acyl iodide, which, upon reaction with K_2_CO_3_ via decarboxylation, formed carboxylate.

**Scheme 53:**
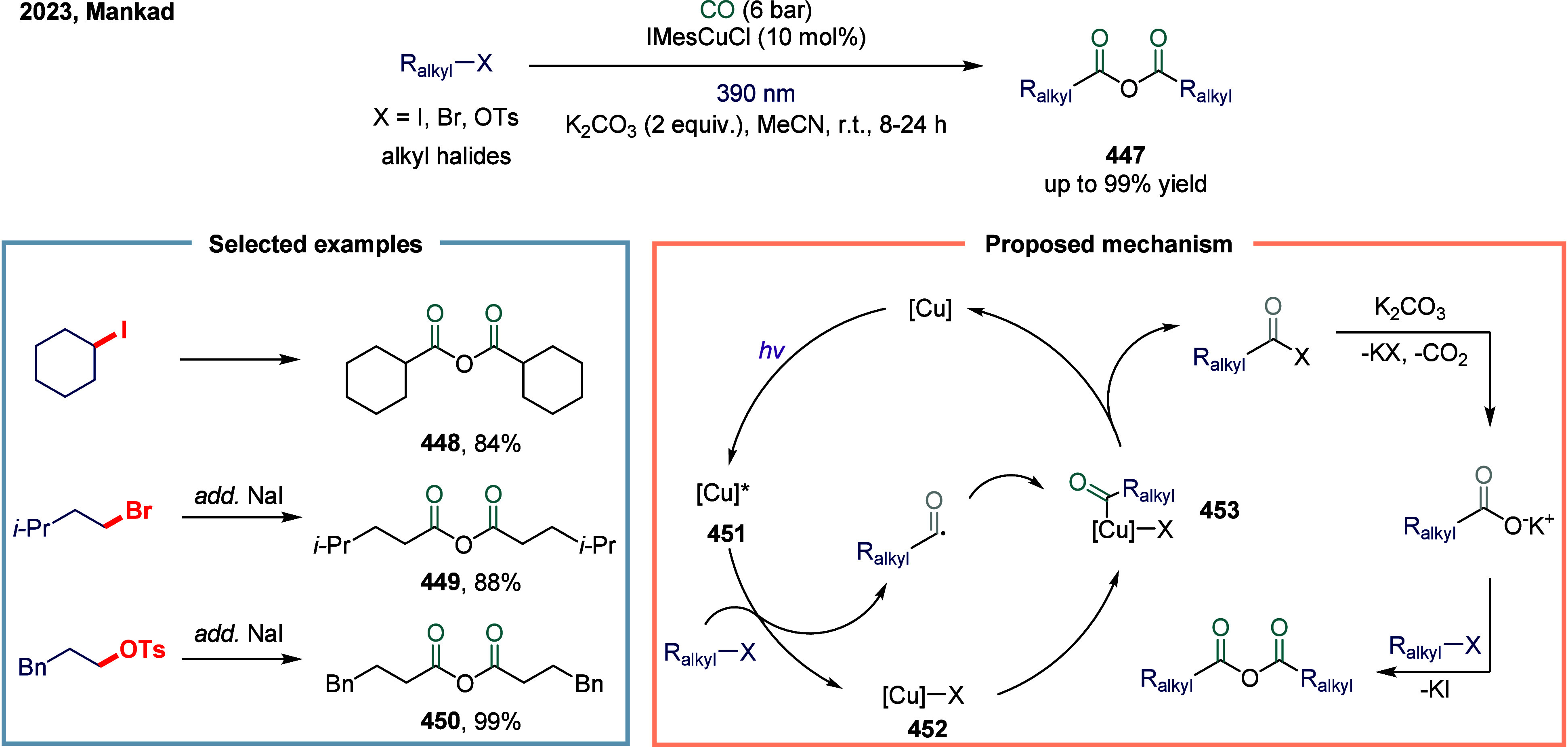
Light-Mediated Synthesis of Aliphatic Anhydrides by Cu-Catalyzed Carbonylation of Alkyl Halides

The selective incorporation of one or more CO molecules into a single substrate has long been a highly attractive objective in the field of carbonylation chemistry. Although numerous catalytic carbonylation methods have been developed via both ionic and radical pathways, a fundamental challenge remains in achieving high selectivity between mono- and double-carbonylation. In 2022, Wu and co-workers induced the use of a copper catalyst to generate and utilize alkyl radicals from alkyl bromides and alkyl iodides (Scheme 54).^273^ This strategy enabled highly controllable and selective double- and monocarbonylation reactions for the synthesis of α-ketoamides **454** and amides **455** under different reaction conditions. In particular, α-ketoamides are key structural units commonly found in natural products and are widely present in the design of various biologically active inhibitors.^274–277^ In this reaction, highly selective double carbonylation of alkyl bromides, as well as both double and monocarbonylation of alkyl iodides, can be achieved under distinct reaction conditions. The authors carried out mechanistic studies and proposed a plausible reaction mechanism to explain the selectivity differences between alkyl bromides and alkyl iodides in the carbonylation process. The reaction was proposed to proceed via the formation of a (carbonyl)copper species, followed by nucleophilic attack by an amine to generate a (carbamoyl)­copper intermediate. A subsequent single-electron transfer (SET) between the copper complex and alkyl bromides or iodides led to the formation of an acyl radical. This radical then reacted with the copper species to afford an acyl­(carbamoyl)copper intermediate, which underwent reductive elimination to produce α-ketoamides or amides, thereby regenerating the catalyst. Due to their lower activation energy, alkyl iodides were more readily activated than alkyl bromides, potentially altering the reaction sequence and leading to different selectivity outcomes. Furthermore, carbon monoxide acted not only as a carbonyl source but also as a reductant to convert the copper­(II) precursor into the catalytically active copper­(I) species.

**Scheme 54:**
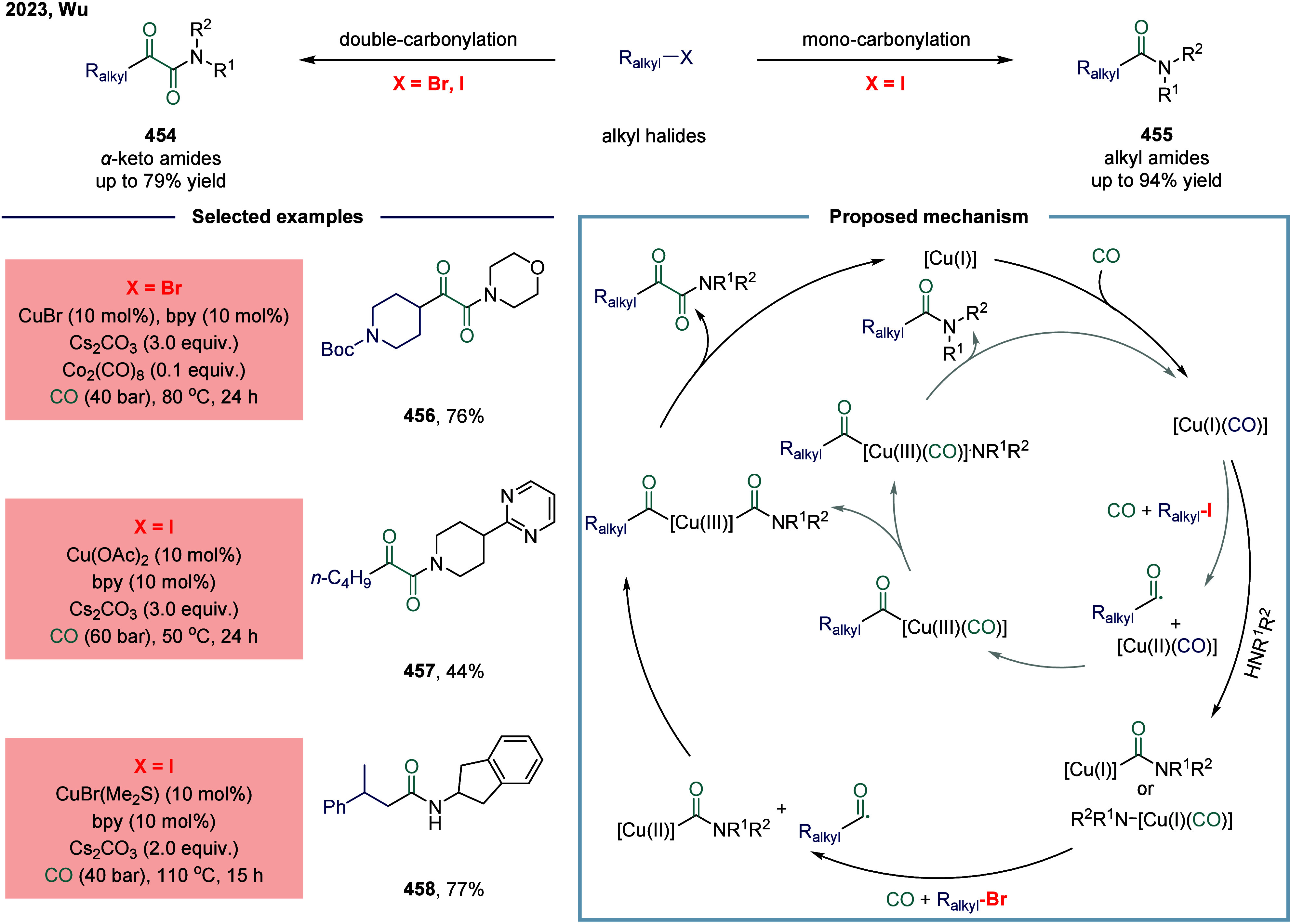
Copper-Catalyzed Substrate-Controlled Carbonylative Synthesis of *α*-Keto Amides and Amides

Shortly after, Wu and co-workers reported a dicarbonylative cyclization strategy for the synthesis of 1,4-diketones **459**, in which four carbon–carbon bonds were formed via two sequential carbon monoxide additions (Scheme 55).^278^ With the CuBr­(Me_2_S) complex as the optimal catalyst and tris­(2,4-di-*tert*-butylphenyl) phosphite as the ligand, 1,4-diketones could be generated in up to 67% yield. Remarkably, with a simple copper catalyst, two molecules of carbon monoxide were incorporated into the double bond, resulting in the formation of four new C–C bonds and a newly constructed ring. The reaction was proposed to initiate with a one-electron reduction of the alkyl bromide by copper, followed by two CO-trapping events. Subsequent oxidation and rearomatization via deprotonation furnished the final products **459**. When the reaction was conducted under a mixed gas atmosphere of 35 bar CO and 5 bar ^13^CO, the ^13^C-labeled product **462** was obtained in 30% yield. This result not only confirms the origin of the carbonyl group in the product but also demonstrates a practical approach for incorporating carbon isotopic labels into molecular frameworks, which holds significant potential in pharmaceutical research.

**Scheme 55:**
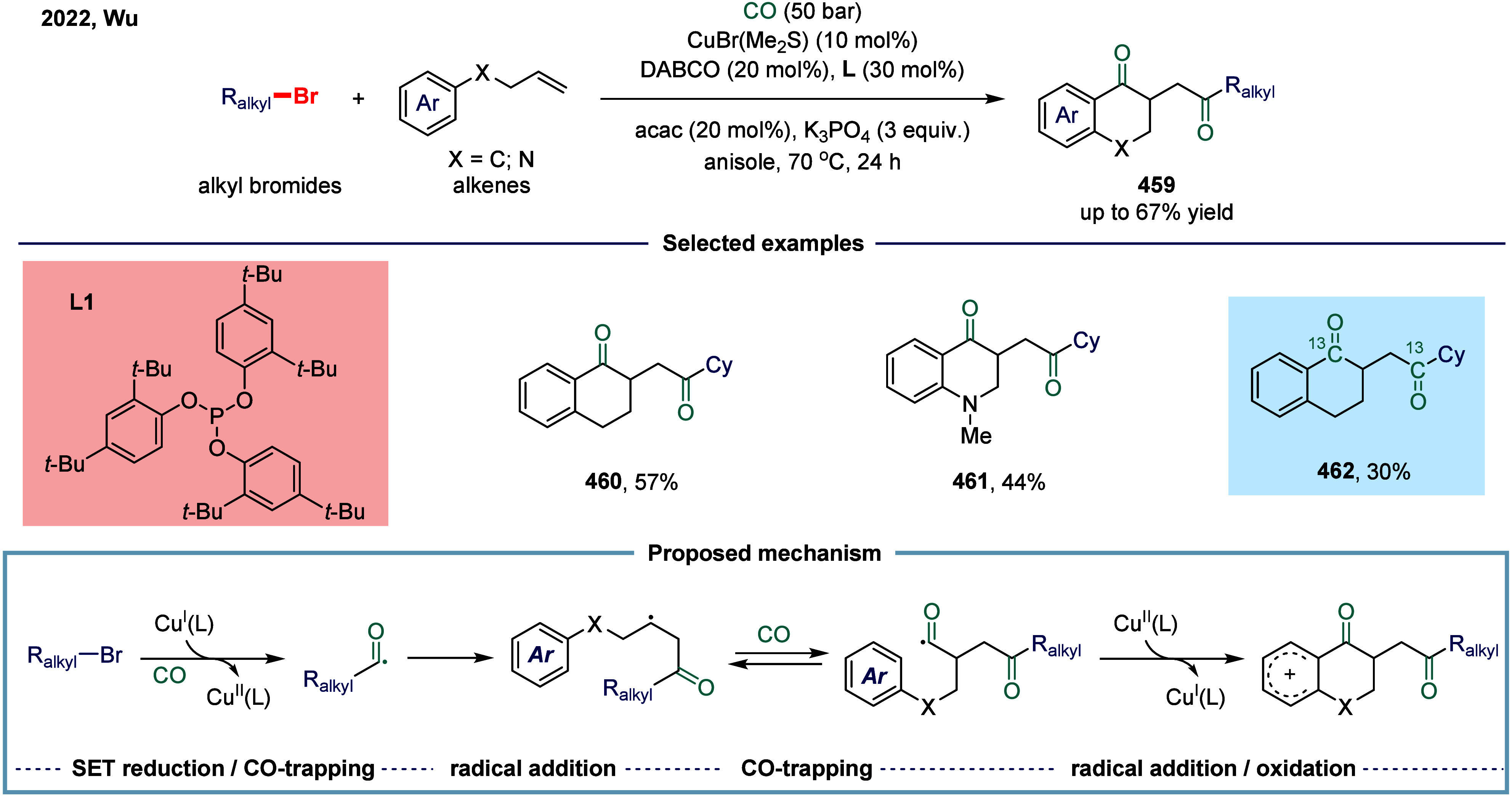
Copper-Catalyzed 1,2-Dicarbonylative Cyclization of Alkenes with Alkyl Bromides

Fluorine is the most electronegative element in nature, and the carbon–fluorine (C–F) bond is widely recognized as the strongest single bond in organic chemistry. The bond dissociation energy of the alkyl-F bond reaches up to 485 kJ/mol, significantly surpassing that of other carbon–halogen bonds. Consequently, traditional chemical methods often struggle to selectively cleave C–F bonds under mild conditions.^279–281^ In 2024, Wu and co-workers reported a carbonylation protocol for unactivated alkyl fluorides using readily available MgI_2_ (Scheme 56). This strategy enabled efficient C–F bond activation and subsequent carbonylation with carbon monoxide.^282^ Various phenols **464–466** were found to be suitable coupling partners; however, when alcohols were employed, only a relatively low yield of the corresponding alkyl carboxylate **467** was obtained. Mechanistic studies revealed that magnesium iodide played a crucial role in this protocol as an additive by promoting halide exchange and facilitating the removal of fluoride ions from the reaction system.

**Scheme 56:**
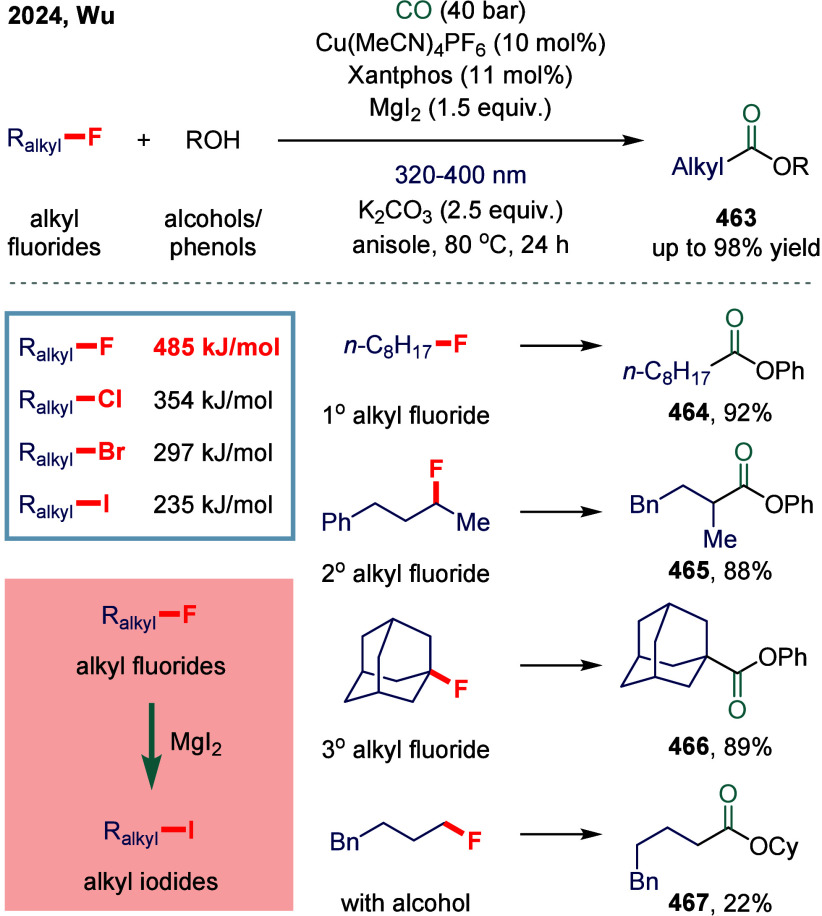
Copper-Catalyzed Alkoxycarbonylation of Alkyl Fluorides

Organocopper complex-driven carbonylation reactions generally utilize (NHC)­Cu-E catalysts. Similar to most reports in organic metal chemistry, imidazole-derived carbene ligands are the most commonly employed, and their electronic and steric properties can be tuned by modifying the substituents on the phenyl or imidazole rings. The development of *N*-heterocyclic carbenes (NHCs) as catalytic ligands has significantly advanced copper-catalyzed carbonylation reactions. Owing to their strong electron-donating ability and high binding affinity for copper, NHC ligands have become essential components in organocopper catalysis. Figure. [Fig fig3] lists some of the most widely used NHC ligands in organocopper-catalyzed carbonylation reactions.

**Figure 3:**
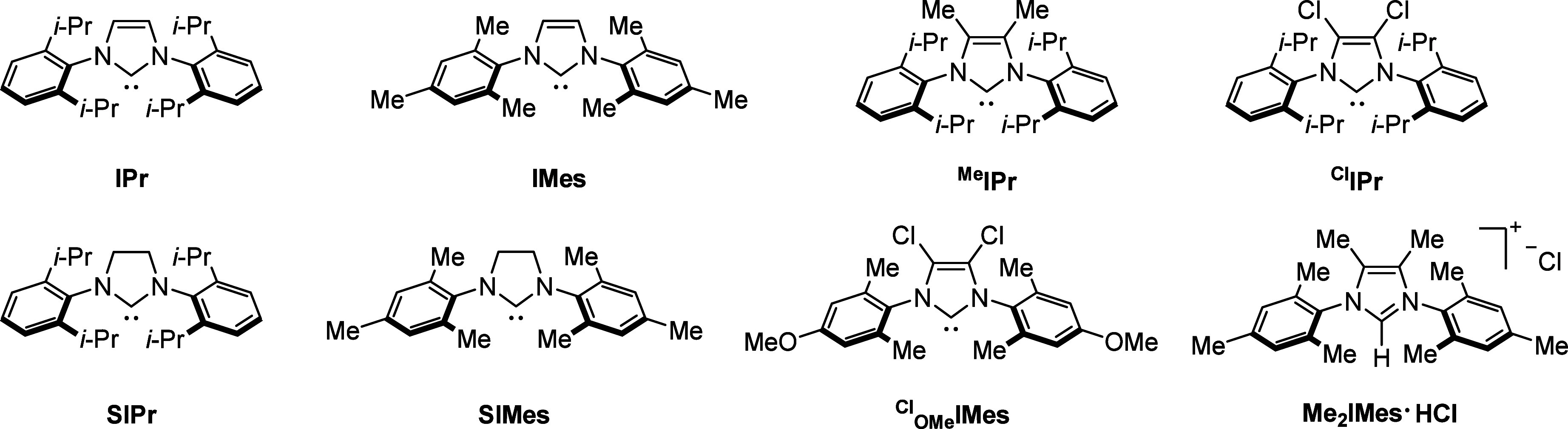
Representative NHC Ligands in organocopper-catalyzed carbonylation reactions.

The Mankad group disclosed a copper-catalyzed hydroxymethylation of unactivated alkyl iodides with CO access to one-carbon-extended alcohols (Scheme 57).^283^ In the presence of multiple functional groups (such as chloro substituents or unsaturated bonds), the reaction proceeds selectively at the C–I bond, affording the corresponding alcohol **469**. Mechanistically, the transformation was proposed to follow an ATC pathway, in which an acyl iodide serves as the key intermediate. This acyl halide was subsequently reduced to the corresponding alcohol via a tandem (NHC)­CuH-catalyzed sequence involving an aldehyde intermediate **468**. However, due to the thermal instability of (NHC)­CuH species, direct mechanistic validation remains challenging. Therefore, the involvement of a Cu-mediated SET pathway (as previously described) could not be definitively ruled out. This consideration was particularly relevant for primary alkyl iodide substrates, where the atom transfer step leading to acyl radical formation was thermodynamically unfavorable. It was thus conceivable that primary alkyl iodides underwent hydroxymethylation via a mechanistically distinct pathway, different from that proposed for secondary and tertiary substrates.

**Scheme 57:**
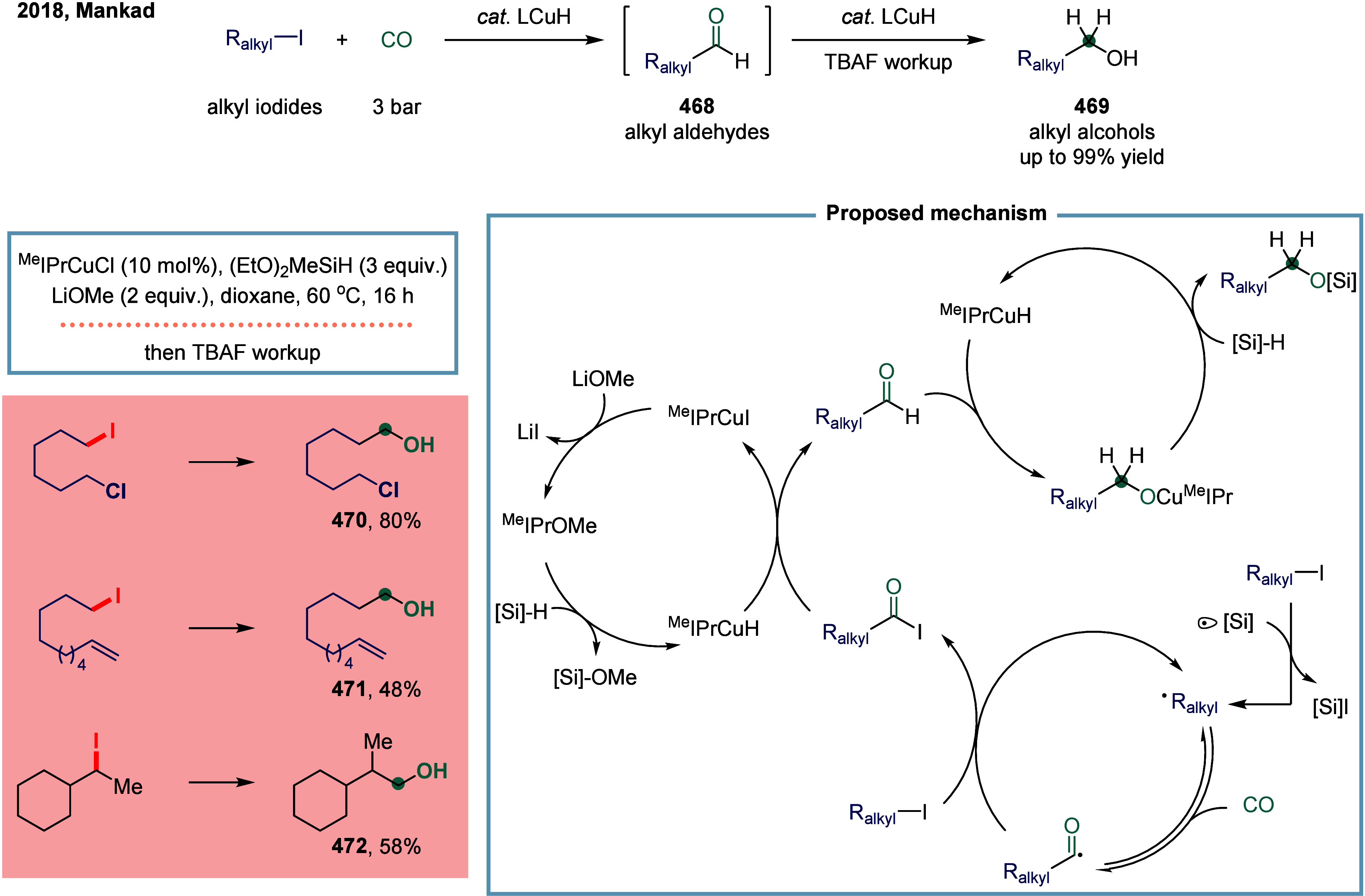
Copper-Catalyzed Hydroxymethylation of Alkyl Iodides with CO to Access One-Carbon-Extended Alcohols

The mode of activation through Cu–H species was then applied toward the carbonylation transformations of alkyl halides by Mankad and Wu laboratories (Scheme 58). Nitroarenes, serving as amine precursors, were readily converted to amines via Cu–H mediated nitroarene reduction, followed by coupling with various alkyl iodides and carbon monoxide to afford the corresponding amides (Scheme 58a).^284^ When nitroarenes bearing cyanide groups were used, no desired product **476** was obtained, possibly due to deactivation of the Cu–H species by the cyanide groups. Subsequently, the Wu group developed a copper-catalyzed alkoxycarbonylation of alkyl iodides for the synthesis of aliphatic esters, employing hydrogen instead of silanes (Scheme 58b).^285^ NaO*t*-Bu served dual roles as both a nucleophile and a base. Furthermore, the introduction of additional alcohols enabled the synthesis of various aliphatic esters **478–480** in moderate to good yields.

**Scheme 58:**
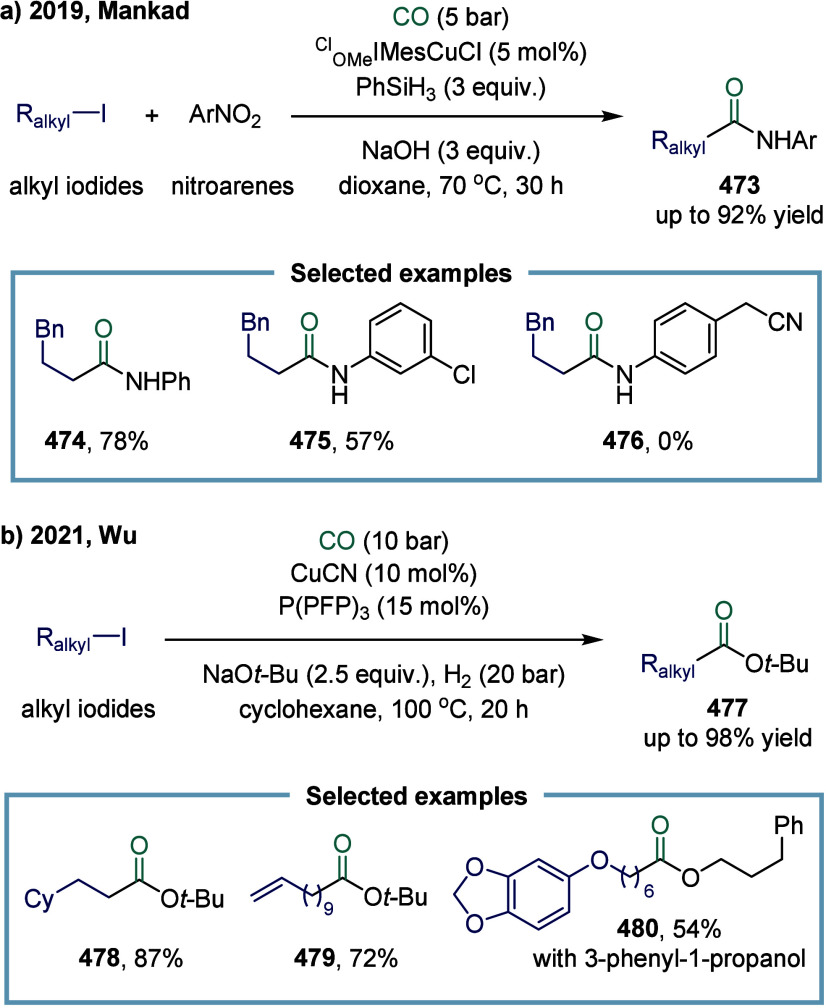
Copper–Hydrogen-Catalyzed Carbonylation of Alkyl Iodides

Acylsilanes,^286–288^ featuring a silicon moiety bonded to a carbonyl group, have emerged as versatile synthetic building blocks. In recent years, they have found increasing application in diverse organic transformations, particularly those involving Brook-type rearrangements that are uniquely facilitated by the acylsilane motif.^289–292^ Mankad’s group envisioned a straightforward carbonylation strategy for the construction of the acylsilane framework **481**, employing commercially available PhMe_2_Si-Bpin as a silicon source (Scheme 59a).^293^ It was anticipated that the alkyl radical, generated from alkyl iodides or alkyl bromides via a silylcopper­(I) complex-mediated SET process, added to CO to form a new acyl radical, which was subsequently trapped by the silylcopper­(II) complex to yield the key copper­(III) intermediate **489**. Moreover, the cleavage of the carbon–halogen bond is not involved in the rate-determining step. A variety of functional groups are well tolerated under mild reaction conditions, and primary **483–484** and tertiary alkyl halides **485** were all compatible. Notably, at elevated temperatures, the scope of Si-based substrates extends to include Et_3_Si-Bpin.

**Scheme 59:**
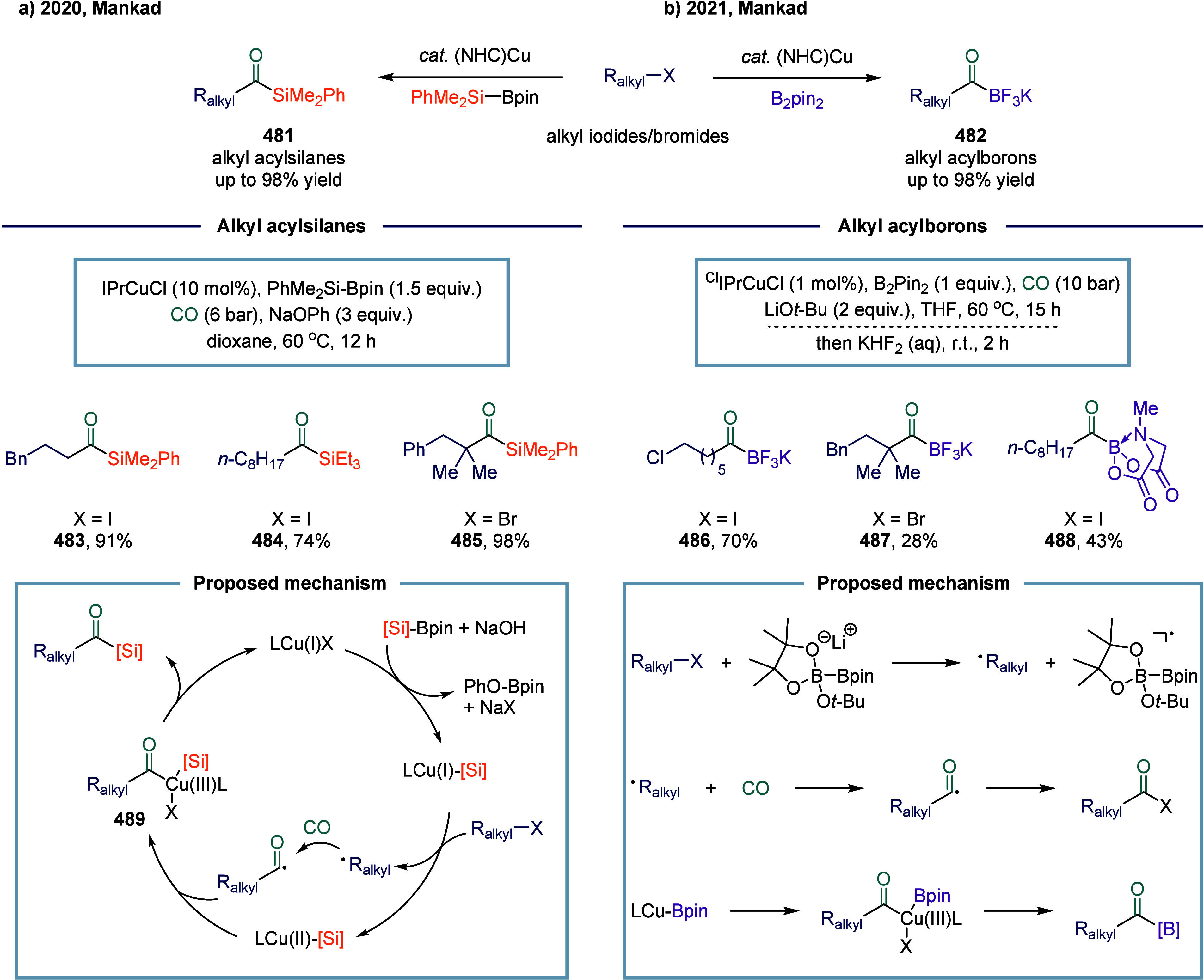
Copper-Catalyzed Carbonylative Silylation and Carbonylative Borylation of Alkyl Halides

Building on this precedent, Mankad and co-workers used ^Cl^IPrCuCl and B_2_pin_2_ to generate acylboron compounds from alkyl iodides or alkyl bromides (Scheme 59b).^294^ Organoboron compounds are versatile intermediates widely utilized in synthetic transformations;^295^ however, acylboron compounds - a relatively underexplored subclass - have received limited attention due to their intrinsic instability.^296–298^ Specifically, tricoordinate acylborons are prone to decomposition, posing a significant synthetic challenge.^299–301^ To circumvent this limitation, the authors developed a strategy to convert the *in situ* generated tricoordinate species into more stable tetracoordinate acylboron derivatives. By quenching the reaction mixture with aqueous KHF_2_, potassium acyltrifluoroborates (KATs) were efficiently obtained. This one-step approach enables direct access to a broad range of aliphatic KATs that previously required multistep synthesis. The methodology tolerates a wide range of functional groups and exhibits broad substrate scope, including primary and tertiary alkyl halides, which deliver the desired products **486–487** in good yields when ^Cl^IPrCuCl is used as the catalyst. Beyond KAT formation, the Bpin group could also be successfully converted into B­(MIDA) derivatives **488** using *N*-methyliminodiacetic acid. Mechanistically, the initiation step is proposed to involve the reaction of LiO*t*-Bu with B_2_pin_2_, generating a B­(sp^2^)-B­(sp^3^) species capable of single-electron transfer to alkyl halides, thereby producing alkyl radicals.^302^ Given that atom transfer from primary alkyl iodides to acyl radicals is thermodynamically disfavored, the Cu catalyst is believed to facilitate this step, possibly via an unidentified mechanism. Following the carbonylative borylation, the combination of the acylboron intermediate with LiO*t*-Bu to form a more stable tetracoordinate complex is critical for suppressing side reactions typically associated with tricoordinate acylborons.

In 2025, Wu and co-workers developed a copper-catalyzed carbonylative Suzuki-Miyaura coupling alkyl bromides and aryl boronates for the synthesis of C­(sp^3^)-C­(sp^2^) ketones in up to 83% yield (Scheme 60).^303^ The aryl copper species **491**, formed via a transmetalation step, underwent single-electron reduction with alkyl bromides under a carbon monoxide atmosphere to afford the high-valent acyl copper intermediate **492**. Both primary and secondary alkyl bromides were compatible with this transformation, furnishing the corresponding carbonylation products **493** and **494** in 60% and 63% yield, respectively. In contrast, tertiary alkyl bromides proved incompatible with the system, delivering the desired product **495** in only low yield.

**Scheme 60:**
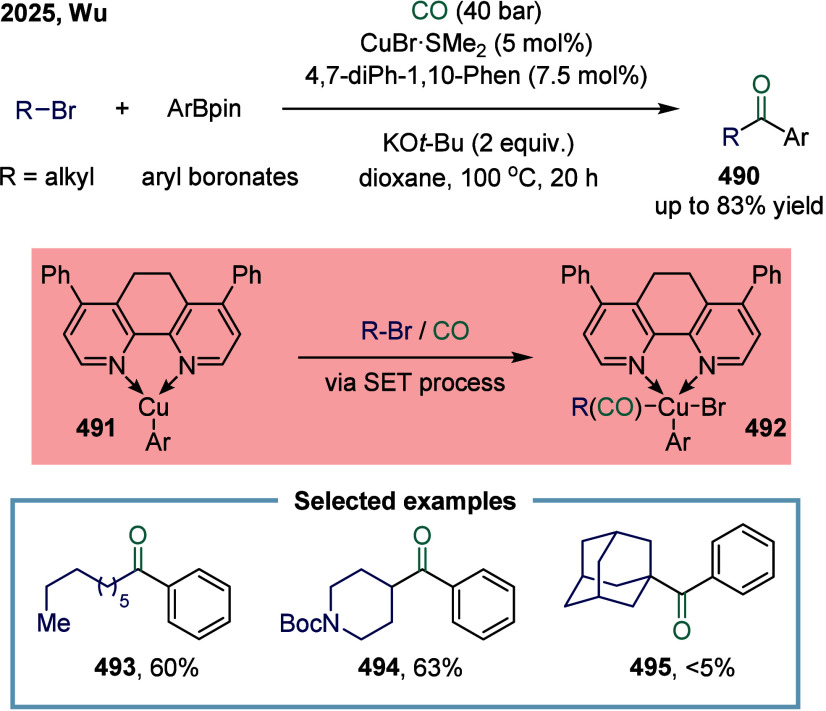
Copper-Catalyzed Carbonylative Suzuki-Miyaura Coupling of Alkyl Bromides with Aryl Boronates

#### 2.7.3 Unsaturated Bonds

Unsaturated bond compounds constitute a significant class of chemical compounds with applications in the bulk, pharmaceutical, or perfume industry.^304–306^ The first to be mentioned are alkene polymerization reactions that enable the production of indispensable plastics. Additionally, unsaturated bond compounds can undergo addition, coupling, oxidative cleavage, or cycloaddition reaction to obtain valuable organic compounds. Among various functionalization strategies of unsaturated bonds, the significance of carbonylation lies in the simultaneous introduction of a carbonyl group and another functional group (sometimes hydrogen) onto carbon frameworks, enabling facile access to value-added chemicals. Generally, SET-mediated carbonylation of unsaturated bonds occurs via two primary pathways: one involves the selective addition of a Cu-E catalyst to the unsaturated bond, accompanied by single-electron reduction of the electrophile; the other proceeds through radical relay carbonylation between single-electron active species and the unsaturated bond. In this section, we focus on copper-catalyzed SET-mediated carbonylation reactions of unsaturated bonds.

In 2017, Mankad and co-workers achieved copper-catalyzed dydrocarbonylative C–C coupling of terminal alkynes with alkyl iodides (Scheme 61a).^307^ This transformation was accomplished using IPrCuCl as the sole catalyst, with KOMe serving as the base and polymethylhydrosiloxane (PMHS) as the reducing agent, demonstrating broad compatibility with both primary and secondary alkyl iodides. A diverse range of unsymmetrical dialkyl ketones **496–499** were synthesized in good to excellent yields. A plausible reaction mechanism was proposed as follows: the catalytic cycle was initiated by the generation of the active IPrCuCl species, which underwent hydrocupration with the alkyne substrate to yield an alkenylcopper intermediate. Subsequent reaction with an alkyl iodide likely produces an alkyl radical via a SET process. This radical rapidly added to carbon monoxide, generating an acyl radical that then recombined with a Cu­(II) species to form a transient Cu­(III) intermediate. Reductive elimination from this intermediate affords the corresponding *α,β*-unsaturated ketone and regenerates IPrCuI. In the second step of the tandem sequence, the in situ formed *α,β*-unsaturated ketone was promptly reduced by IPrCuH to generate a copper enolate. This intermediate undergoes σ-bond metathesis with the silane, regenerating IPrCuH and forming a silyl enol ether. Upon aqueous workup, the silyl enol ether is hydrolyzed to yield the final dialkyl ketone product **496**.

**Scheme 61:**
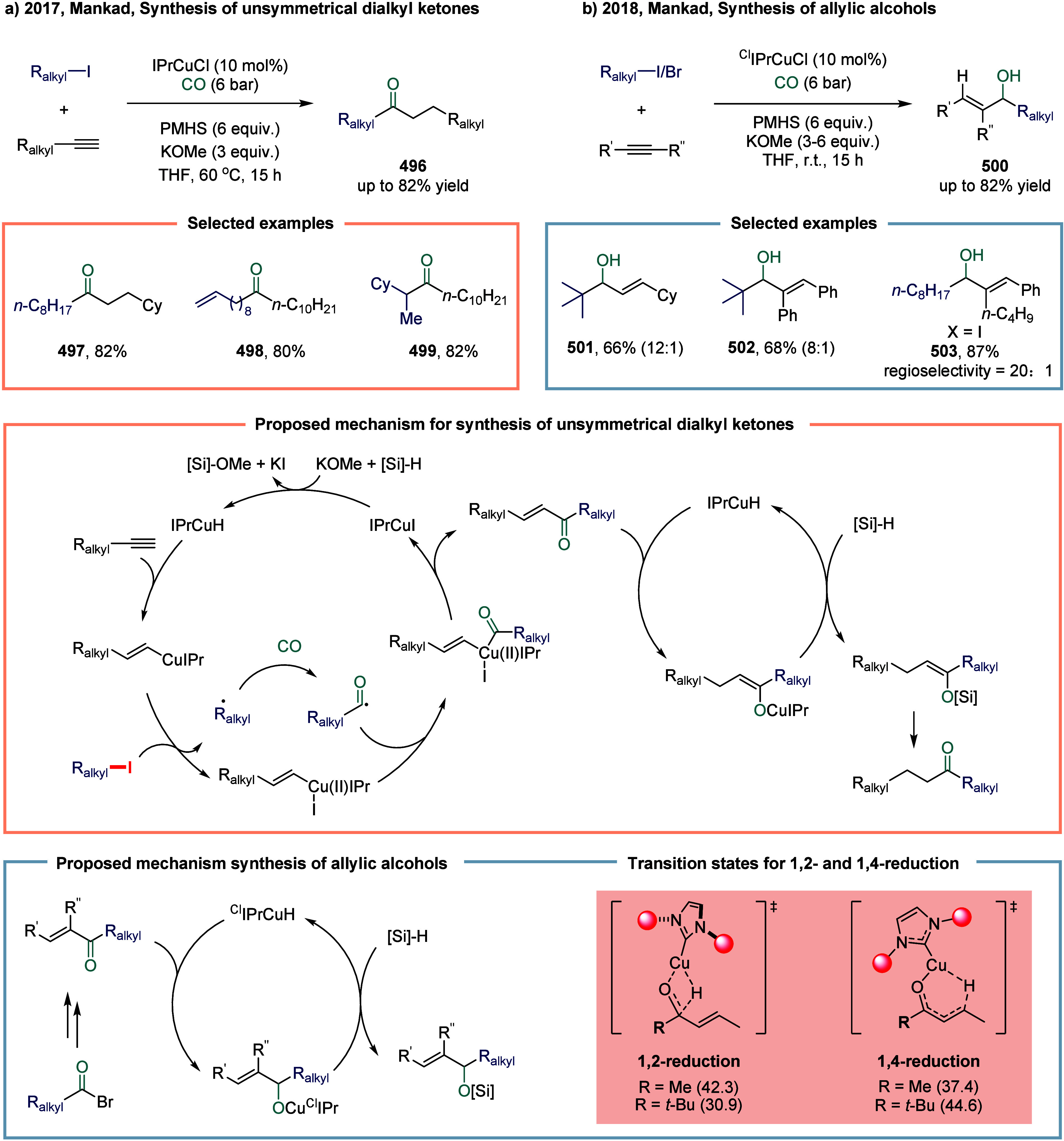
Copper-Catalyzed Hydrocarbonylative of Alkynes with Alkyl Iodide Access to Ketones and Allylic Alcohols

In subsequent studies, Mankad and co-workers demonstrated the selective 1,4-reduction of *α,β*-unsaturated ketone intermediates to access a broad range of allylic alcohols (Scheme 61b).^308^ The allylic alcohol motif is widely found in complex organic molecules and is regarded as a privileged functional group due to the versatile reactivity of both the alkene and hydroxyl moieties.^309,310^ In this system, allylic alcohols **500** were obtained in high yields using ^Cl^IPrCuCl as the catalyst and tertiary alkyl bromides as coupling partners under mild, room-temperature conditions. Both terminal **501** and internal alkynes **502** proved to be compatible with the optimized reaction parameters. Moreover, a variety of primary and secondary alkyl iodides underwent efficient carbonylation coupling with internal alkynes to afford the corresponding allylic alcohols. Notably, these products represent a significant structural class frequently found in natural products and bioactive compounds, and they also serve as valuable intermediates for the construction of quaternary carbon centers. Mechanistic investigations revealed that tertiary alkyl halides underwent atom-transfer carbonylation via a SET pathway, which differs fundamentally from the mechanism operative for primary and secondary electrophiles. Although *α,β*-unsaturated ketones were confirmed as key intermediates, they could be generated in high yield only through the stoichiometric coupling of alkenylcopper with pivaloyl bromide - not with *tert*-butyl bromide - highlighting the essential role of hydrosilane in initiating alkyl radical formation and further emphasizing the mechanistic divergence between tertiary and nontertiary halides. To probe the origin of regioselectivity in the enone reduction step, DFT calculations were performed on methyl- and *tert*-butyl-substituted enones using a model (NHC)­Cu–H complex. The results indicated a lower activation barrier for 1,4-reduction in the methyl enone (ΔΔG^⧧^ = 4.9 kcal/mol), while the *tert*-butyl enone favored 1,2-reduction (ΔΔG^⧧^ = 13.7 kcal/mol), in agreement with experimental data. This selectivity is attributed to steric effects in the transition state and is further supported by independent computational studies from Chen and co-workers.^311^

In 2018, Mankad and co-workers discussed a copper-catalyzed borocarbonylative coupling of internal alkynes with unactivated alkyl halides for the synthesis of tetrasubstituted β-borylenones **504** (Scheme 62).^312^ To enable efficient isolation and purification, the borylated enone intermediates were subsequently reduced to the corresponding oxaboroles using NaBH_4_. Ligand screening revealed that SIMes was optimal for diaryl-substituted alkynes, whereas MeIMes delivered improved performance with aryl-alkyl disubstituted substrates. Notably, the reaction conditions exhibited broad substrate scope, accommodating primary, secondary, and even sterically hindered tertiary alkyl halides. The reaction began with the formation of LCu-Bpin, followed by borocupration of the alkyne substrate to generate the β-boroalkenylcopper intermediate **508**. This copper­(I) species subsequently underwent a single-electron transfer (SET) with the alkyl iodide, affording a copper­(II) intermediate. The latter then captured an acyl radical, which was generated from the corresponding carbon-centered radical under a CO atmosphere, leading to the formation of the key coupling intermediate **509**.

**Scheme 62:**
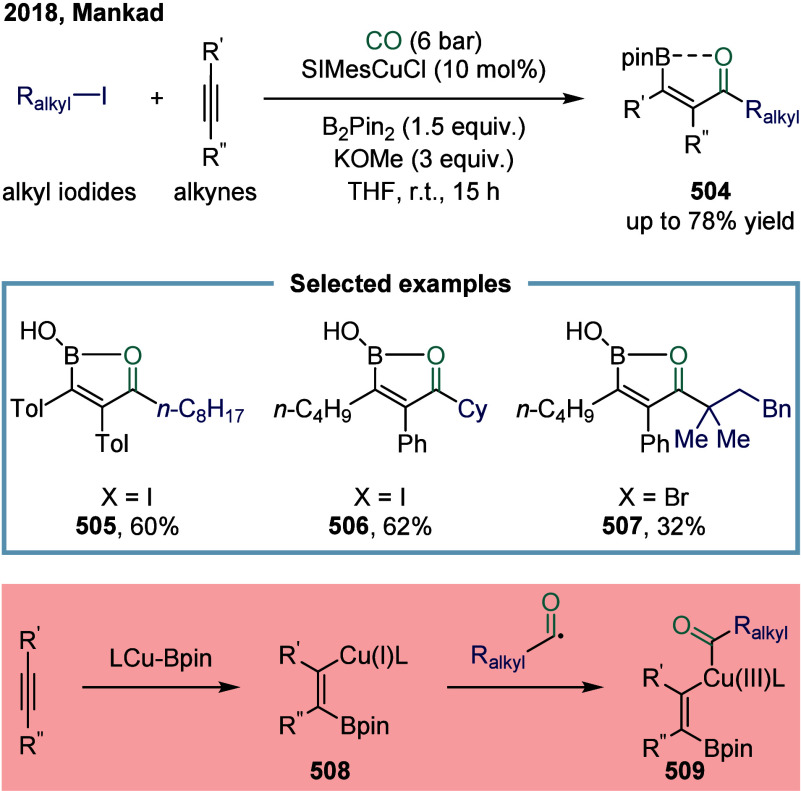
Copper-Catalyzed Borocarbonylative Coupling of Internal Alkynes with Unactivated Alkyl Halides

Inspired by the above work, Wu and co-workers also successfully achieved a copper-catalyzed regioselective borocarbonylative coupling of unactivated alkenes with alkyl halides (Scheme 63a).^313^ Notably, in addition to the IPrCuCl catalyst, the inclusion of Xantphos as an additive was essential for achieving satisfactory yields (up to 95% yield). The reaction proceeded efficiently with a range of functionalized primary alkyl iodides (**511–513**); however, only trace amounts of product were observed when a secondary alkyl iodide was employed. However, in contrast to the mechanism previously proposed by Mankad, which involves the generation of acyl radicals or acyl halide intermediates from alkyl halides, an alternative pathway has been suggested. In this mechanism, single-electron reduction of the alkyl halide by Cu­(I) was followed by carbon radical capture, leading to the formation of an oxidative adduct intermediate. Subsequent CO migratory insertion into this complex generates acylcopper species, which then undergo reductive elimination to furnish the final products. Subsequently, Wu’s group has developed a copper-catalyzed 1,2-borylcarbonylation of unactivated olefins, delivering products **514** with complementary regioselectivity (Scheme 63b).^314^ Mechanistic studies suggest that the reaction proceeds via a radical relay carbonylative borylation pathway.

**Scheme 63:**
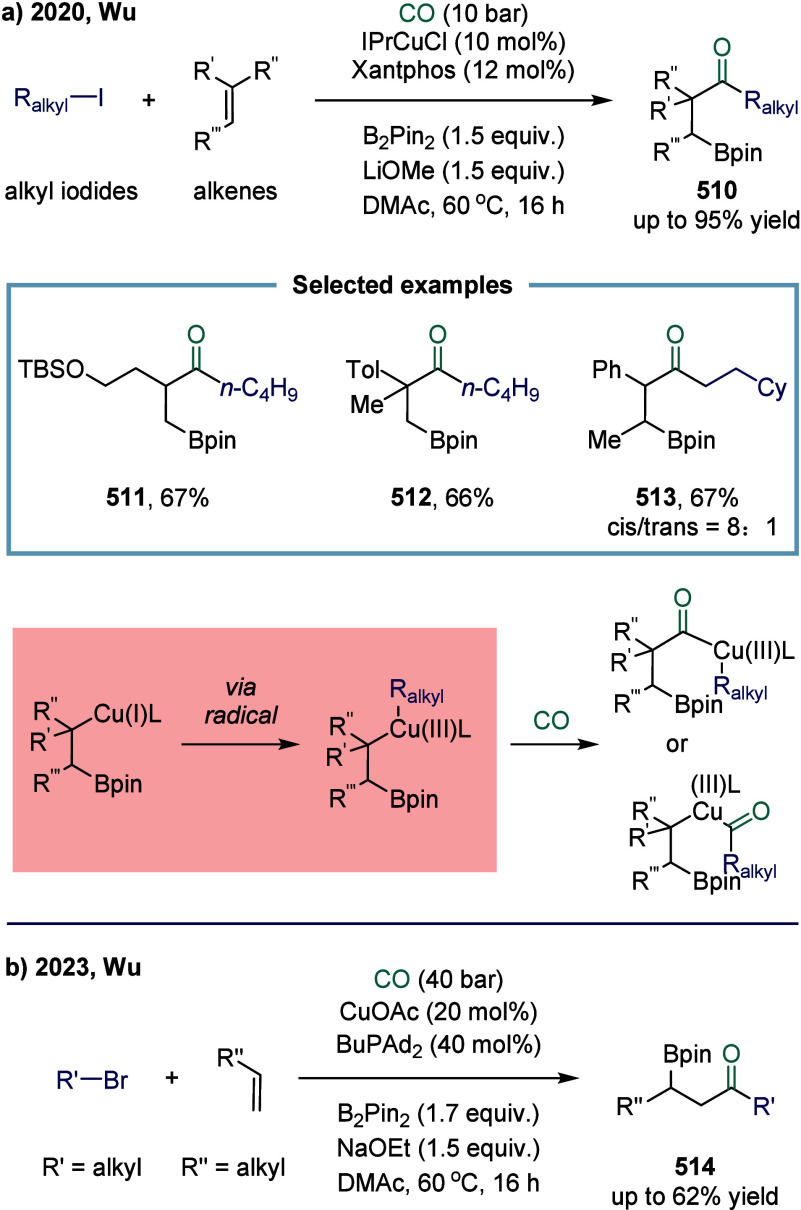
Copper-Catalyzed Borocarbonylative Coupling of Unactivated Alkenes with Alkyl Halides

Radical-relay carbonylation has emerged as a powerful strategy for constructing carbonyl-containing molecules from readily available starting materials. This approach typically involves the generation of a carbon-centered radical, followed by sequential relay steps that enable the incorporation of carbon monoxide (CO) into the molecular framework. Compared to classical transition-metal-catalyzed carbonylation reactions that proceed through two-electron pathways, radical-relay mechanisms offer distinct advantages in terms of functional group tolerance, substrate diversity, and mild reaction conditions. Notably, the radical relay enables site-selective transformations even with unactivated alkenes, expanding the synthetic utility of carbonylative cross-couplings.

In recent years, Wu’s research group has made significant advances in the field of radical relay carbonylation, contributing several innovative strategies that expand the scope and mechanistic understanding of this emerging transformation. A copper-catalyzed carbonylation synthesis of β-homoprolines **515** from *N*-fluoro-sulfonamides was disclosed by Wu and co-workers (Scheme 64a).^315^ The catalytic cycle commenced with the generation of amidyl radical **516** via copper­(I)-induced SET reduction, which proceeded through cleavage of the N–F bond and oxidation to a copper­(II) species. Subsequently, radical **516** underwent intramolecular cyclization to furnish a new carbon-centered radical. This intermediate was then captured by carbon monoxide and the Cu­(II) species to form the acyl-copper complex. Finally, reductive elimination from acyl-copper complex afforded the desired product and regenerated the active Cu­(I) catalyst, thus completing the catalytic cycle. In addition to N–F bond cleavage, aromatic oxime esters have also been employed as effective precursors for cyclizative carbonylation in the presence of copper catalysts (Scheme 64b).^316^ More than 60 structurally diverse *N*-heterocycle-substituted amides **517** were synthesized in moderate to excellent yields by employing a broad array of readily accessible amines.

**Scheme 64:**
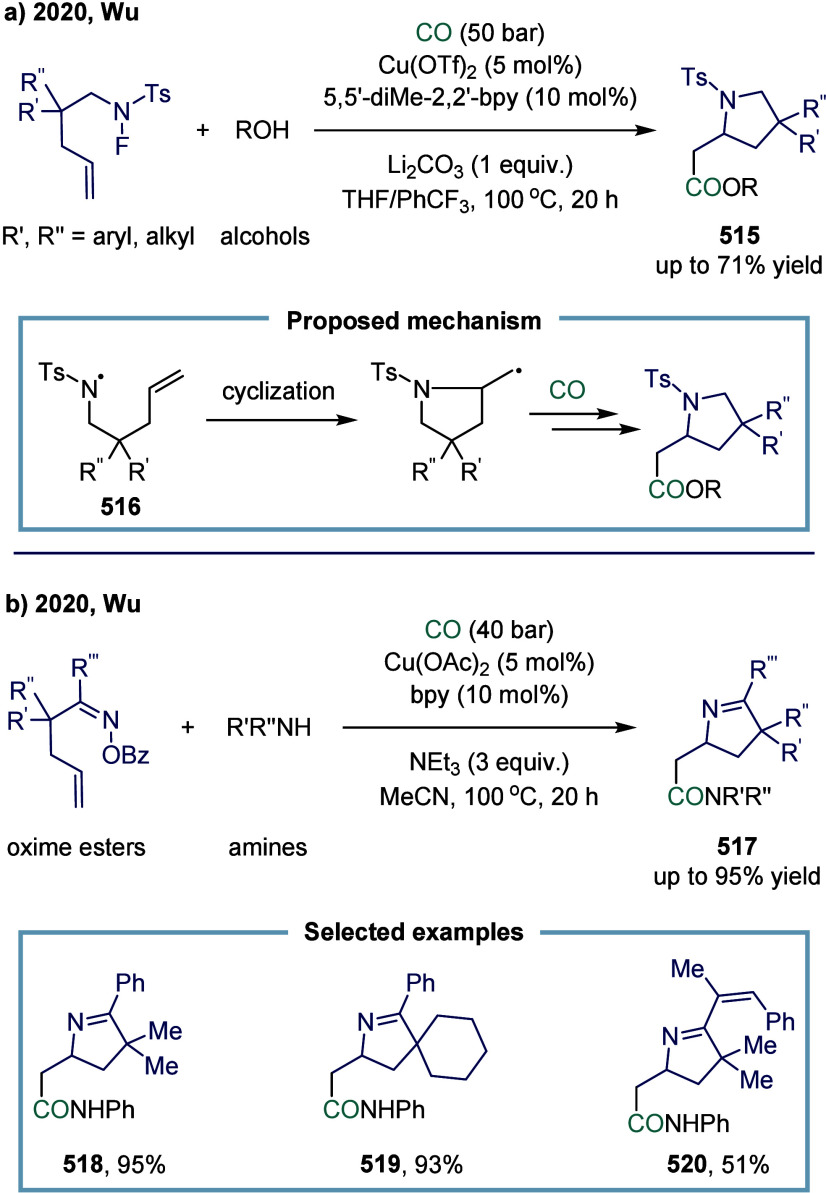
Copper-Catalyzed Carbonylative Synthesis of *β*-Homoprolines and Pyrrolidine-Containing Amides

Ethylene (C_2_H_4_), the simplest alkene, is a highly accessible and industrially significant C2 building block widely utilized in organic synthesis.^317^ Its high atom economy, low cost, and reactive π-bond render it an attractive substrate for a broad spectrum of transition-metal-catalyzed transformations, including hydrofunctionalization, difunctionalization, and carbonylation reactions. Ethylene’s capacity to undergo regioselective and chemoselective additions under mild conditions has enabled its application in the synthesis of value-added fine chemicals, heterocycles, and structurally complex molecules. Moreover, its incorporation into multicomponent reactions offers valuable opportunities to enhance molecular diversity and synthetic efficiency. However, electrophilic carbon-centered radicals exhibit relatively low reactivity toward ethylene, with an addition rate constant of approximately 10^3^ M^–1^ s^–1^, substantially lower than that for more substituted alkenes.^318^ Consequently, ethylene is generally classified as a less reactive substrate in radical addition processes. In addition, both ethylene and carbon monoxide are susceptible to undesired polymerization under elevated temperatures and pressures, posing further challenges to achieving selective and controlled transformations.^319^

In the realm of copper-catalyzed radical-relay carbonylation, Wu and co-workers reported a four-component carbonylative transformation for the synthesis of γ-cyanocarboxylic acid derivatives **521** without the need for noble metal catalysts (Scheme 65a).^320^ In this system, direct C–H activation of acetonitrile was achieved using DTBP as a highly reactive oxidant, enabling the generation of α-cyanoalkyl radical via SET. However, due to the electron-deficient nature of the α-cyanoalkyl radical, direct coupling via either thermal or copper-catalyzed pathways proved inefficient. Instead, the radical readily underwent intermolecular addition to alkenes, generating the corresponding alkyl radicals. These alkyl radicals subsequently engaged in coupling with nucleophiles in the presence of a catalytic amount of copper complex. Under CO atmosphere, a variety of γ-cyanocarboxylates **521** were obtained in moderate to good yields by employing ethylene or other aliphatic olefins, alcohols, and acetonitrile as substrates.

**Scheme 65:**
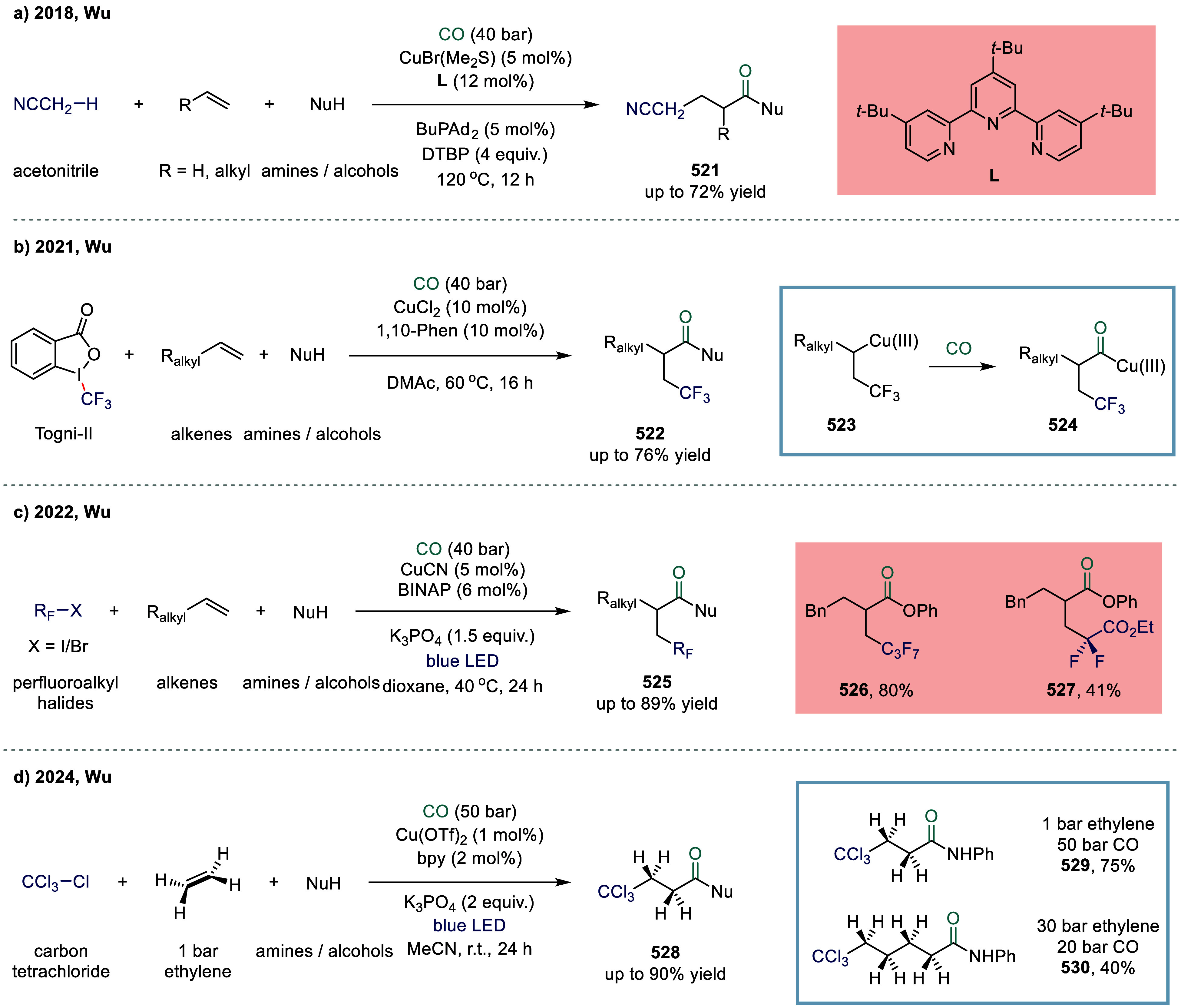
Copper-Catalyzed Multicomponent Carbonylation of Alkenes via Radical-Relay Pathway

Fluorine-containing compounds have garnered significant attention in organic chemistry due to their unique physicochemical properties and broad applications across pharmaceuticals, agrochemicals, and materials science.^321^ The incorporation of fluorine atoms or fluorinated functional groups into organic molecules often imparts enhanced metabolic stability, increased lipophilicity, and improved bioavailability, making them indispensable in drug design and development.^322^ Received this inspiration, Wu and co-workers reported a copper-catalyzed 1,2- trifluoromethylation carbonylation of unactivated alkenes for the synthesis of β-trifluoromethylated aliphatic carboxylic acid derivatives (Scheme 65b).^323^ The key intermediate **523** was formed via a coupling reaction between a copper catalyst and an alkyl radical, the latter being generated through a radical-relay pathway. Subsequent CO coordination and migratory insertion afforded the corresponding acyl-copper species **524**, followed by reductive elimination to afford the carbonylated product. A wide variety of β-trifluoromethylated carboxylic acid derivatives **522** were synthesized from simple alkenes with excellent regioselectivity, delivering moderate to excellent yields. After completing 1,2-trifluoromethylation carbonylation, Wu and co-workers then explored the copper-catalyzed perfluoroalkylative carbonylation of unactivated alkenes (Scheme 65c).^324^ Various perfluoroalkyl substrates, including perfluoroalkyl iodides (**526**) and ethyl difluoroiodoacetate (**527**), were well tolerated under the reaction conditions. In this transformation, perfluoroalkyl halides underwent single-electron reduction mediated by a copper catalyst under blue light irradiation, generating perfluoroalkyl radicals that subsequently engaged in the radical-relay carbonylation process. In addition to fluoroalkyl groups, chloroalkyl moieties represent important structural motifs that modulate the biological activity of organic compounds and are widely found in natural products, pharmaceuticals, and bioactive molecules.^325^ In 2024, the laboratory reported a copper-catalyzed trichloromethylative carbonylation of ethylene by employing commercially available CCl_4_ and CO as trichloromethyl and carbonyl sources, respectively (Scheme 65d).^326^ Using this protocol, a variety of nucleophiles, including amines, phenols, and alcohols, were efficiently converted into β-trichloromethyl carboxylic acid derivatives with good functional group tolerance. Additionally, bis-vinylated γ-trichloromethyl amides **530** were obtained by modulating the pressures of CO and ethylene.

#### 2.7.4 Others

A pioneering study on copper-catalyzed carbonylation of C–C bonds was reported by Wu and co-workers in 2017 (Scheme 66a).^327^ In this work, *N*-acetyl amides **531** were obtained in good yields using a copper catalyst under CO pressure, with a peroxide serving both as the oxidant and the methyl radical source. Remarkably, this represented the first example of carbonylative acetylation. The reaction was proposed to proceed via copper­(II)-catalyzed or thermally induced homolytic cleavage of the peroxide to generate an alkoxy radical. Subsequent β-scission of this intermediate afforded a methyl radical, which then reacted with a copper­(II) species to generate the corresponding Cu­(III)-methyl complex. Subsequent work by Xiao and co-workers disclosed a copper-catalyzed carbonylation strategy involving the selective cleavage of C–C bonds in a series of cycloketone oxime esters (Scheme 66b).^328^ This transformation provided an efficient approach to functionalize carbon–carbon bonds under mild conditions, thereby expanding the scope of copper-catalyzed carbonylative methodologies. The key step in this transformation involves the selective β–C-C bond cleavage of the iminyl radical **533**, leading to the formation of a cyanoalkyl radical **534**. This radical intermediate is subsequently intercepted by a Cu­(II) species to generate a high-valent Cu­(III) complex, which then undergoes carbon monoxide coordination and migratory insertion to furnish the corresponding acylcopper intermediate. This method demonstrated a wide substrate scope and excellent functional group compatibility for both cycloketone oxime esters and alkyl/aryl amines, offering a practical and mild approach to the synthesis of structurally diverse cyanoalkyl-substituted amides **535–537**. In 2024, Guo and co-workers reported a photoinduced copper-catalyzed alkoxyl triggered C–C bond cleavage/aminocarbonylation cascade (Scheme 66c).^329^ By fine-tuning the structure of alkoxyl radical precursors, a diverse array of valuable lactones and carbonyl-functionalized amides **539–541** were efficiently synthesized under mild conditions, exhibiting good yields and excellent tolerance toward various functional groups. Notably, this reaction represents a significant advancement in the synthesis of macrocyclic carboxylic acid derivatives.

**Scheme 66:**
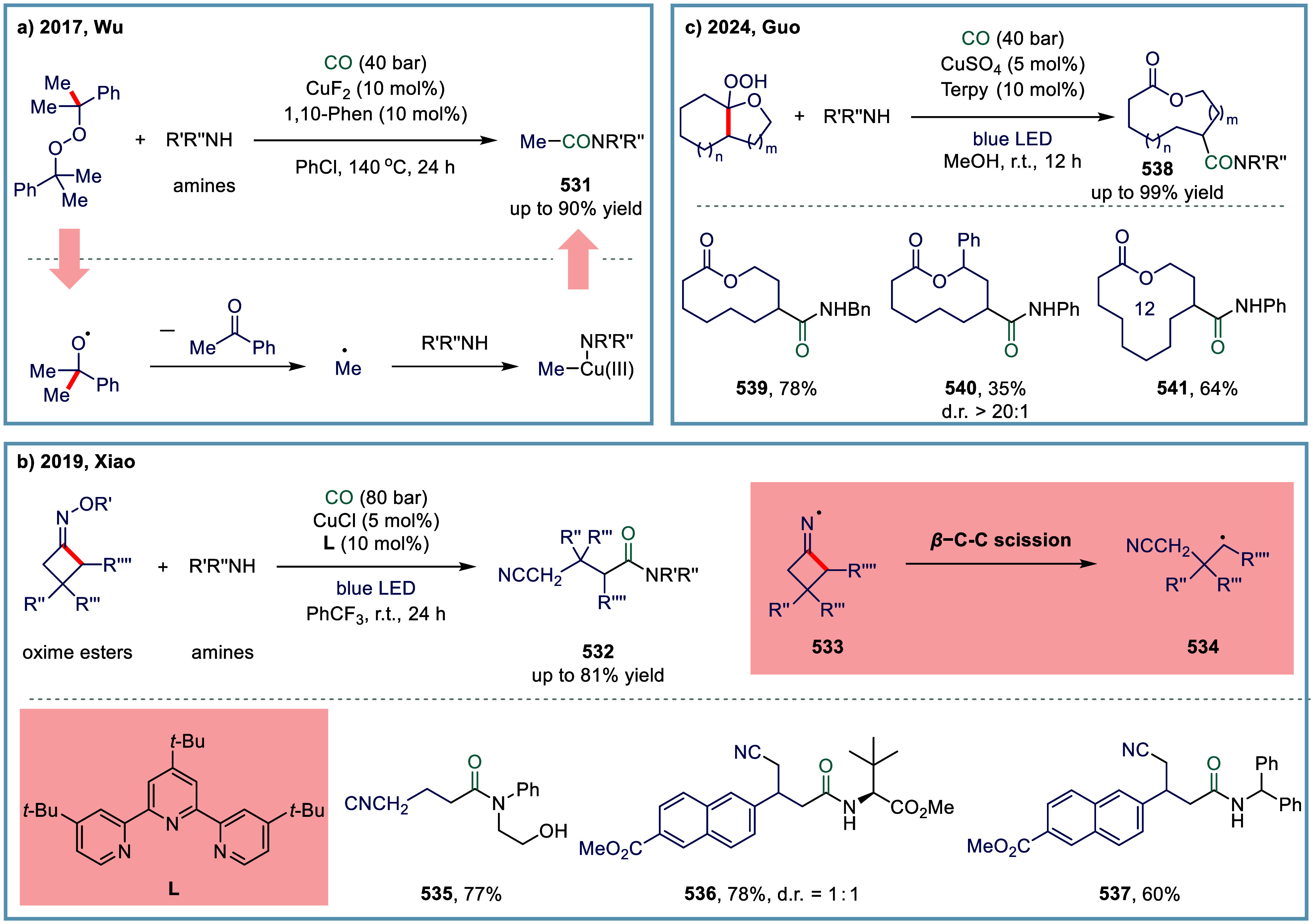
Copper-Catalyzed C–C Bond Cleavage/Aminocarbonylation Cascade

Sulfonium salts constitute a valuable class of organosulfur intermediates that have found widespread application in modern synthetic chemistry for the assembly of structurally diverse, functionalized compounds.^330,331^ Particularly, sulfonium salts have emerged as efficient electrophilic coupling agents in both transition-metal-mediated and photoinduced cross-coupling processes, where the selective cleavage of the exocyclic C–S bond enables the cyclic thioether moiety to act as a traceless functional group transfer handle. Owing to the distinctive reactivity and structural features of sulfur-containing motifs, the resulting thioether frameworks are frequently encountered in pharmaceuticals, natural products, and materials science. Consequently, the regioselective activation and transformation of cyclic sulfonium C–S bonds under carbonylative conditions has become a powerful synthetic strategy for accessing a wide range of sulfur-functionalized carboxylic acid derivatives. In 2023, Wu and co-workers described the copper-catalyzed photoinduced ring-opening carbonylation of sulfonium salts (Scheme 67).^332^ This strategy employed photoredox catalysis to achieve selective cleavage of the C­(sp^3^)-S bond in sulfonium salts, enabling sequential functionalization to construct vicinal C–C and C-X (X = O or N) bonds in the presence of carbon monoxide and nucleophiles. A wide range of substrates was successfully converted into the corresponding carbonylated products **543–545** in moderate to good yields, exhibiting excellent chemoselectivity and broad functional group tolerance.

**Scheme 67:**
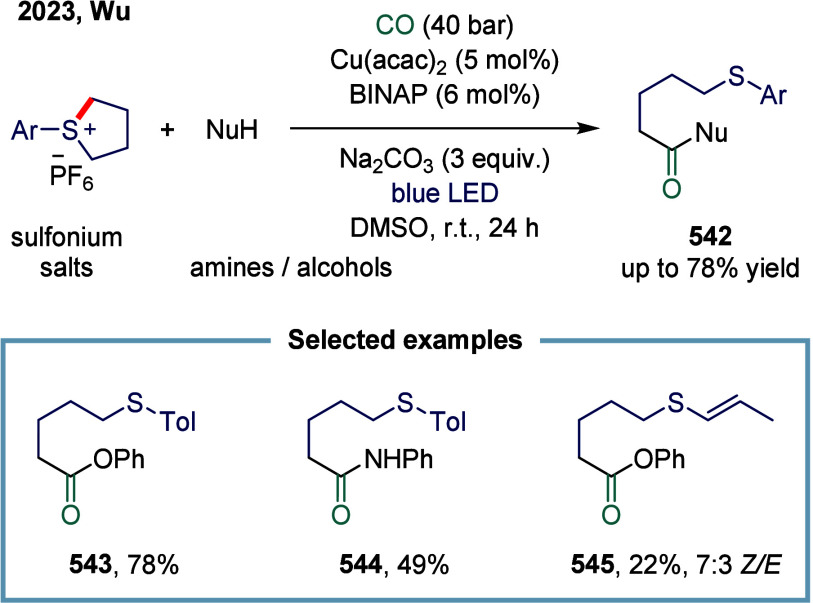
Copper-Catalyzed Visible-Light-Induced Ring-Opening Carbonylation of Sulfonium Salts

In 2017, a novel copper-catalyzed carbonylative cross-coupling reaction between *N*-chloroamines and arylboronic acids was developed by Wu and co-workers, representing the first example of copper-catalyzed aminocarbonylation employing *N*-chloroamines as the nitrogen source (Scheme 68).^333^ Utilizing Cu_2_O as the catalyst under CO atmosphere, a range of amide products **546–548** were obtained in moderate to good yields. Initially, *N*-chlorodialkylamines underwent SET to generate dialkylamino radicals, accompanied by the oxidation of Cu­(I) to Cu­(II). Under a carbon monoxide atmosphere, the resulting dialkylamino radicals reacted with the Cu­(II) species to afford a carbamoylmetal intermediate. This intermediate subsequently underwent transmetalation with the arylboronic acid, followed by reductive elimination to furnish the desired amide product. Concurrently, the active Cu­(I) catalyst was regenerated, completing the catalytic cycle.

**Scheme 68:**
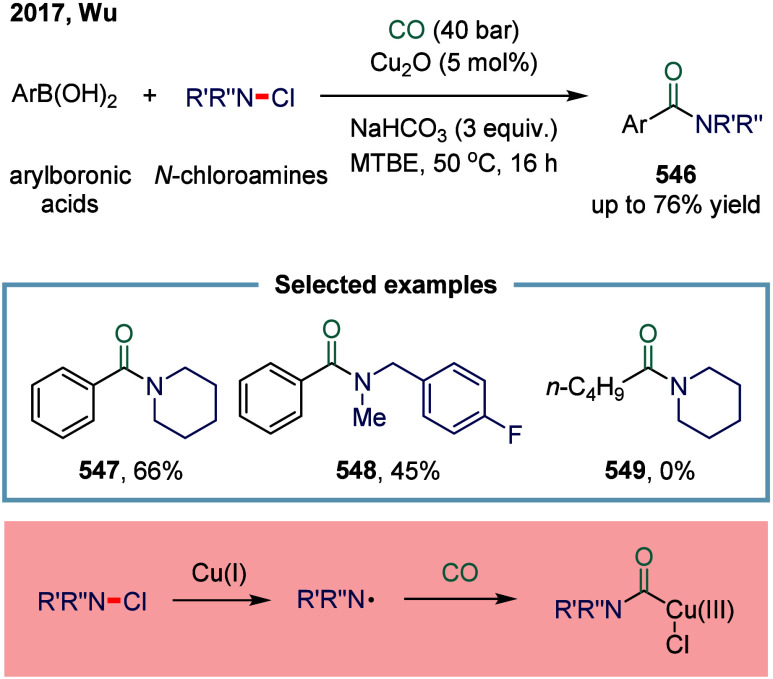
Copper-Catalyzed Carbonylative Coupling of Arylboronic Acids with *N*-Chloroamines

## 3 Second-Row Transition Metals

Second-row transition metals (4d metals), particularly Pd, Rh, and Ru, have become indispensable in modern carbonylation chemistry, with Pd-based catalysts serving as the cornerstone for C­(sp^2^)-halide carbonylation.^334^ Benefiting from their larger *d*-orbitals, strong back-donation ability,^335^ and excellent stabilization of organometallic intermediates, these metals readily promote the oxidative addition of aryl or vinyl halides, followed by CO insertion and nucleophilic capture to generate esters, amides, and ketones. Rhodium, although predominantly used in hydroformylation,^336–338^ has shown potential in C­(sp^2^)–X carbonylation through selective formation of acyl–Rh species in the presence of electron-rich phosphines.^339,340^ Ruthenium-based complexes, such as Ru_3_(CO)_12_, are emerging as alternative catalysts, enabling photochemical or electrochemical C–X activation pathways that complement traditional Pd systems. In these catalytic cycles, Ag­(I) species are frequently utilized as halide scavengers, accelerating oxidative addition and increasing turnover efficiency.

With continuous improvements in ligand design and reaction engineering, 4d metal catalysts have also achieved remarkable progress in single-electron transfer (SET) carbonylation. These systems, often integrating photoredox catalysis, expand the accessible substrate scope by enabling the activation of challenging C­(sp^3^)–halide bonds or inert substrates via radical pathways. Pd-based dual catalytic platforms combining photoredox systems and CO insertion have delivered high selectivity and efficiency in amide and ketone formation, while Ru and Rh catalysts have also been applied to SET-mediated carbonylation strategies to produce structurally complex carbonyl compounds. The synergy between traditional two-electron Pd(0)/Pd­(II) cycles and emerging SET-based pathways underscores the evolving role of second-row transition metals in advancing sustainable, efficient, and versatile carbonylation methodologies.

### 3.1 Ruthenium-Catalyzed System

In 2018, Pelinski and co-workers reported a visible-light-mediated hydroxycarbonylation of diazonium salts (Scheme 69).^341^ In this transformation, irradiation of the photoredox catalyst generated a photoexcited reductive species Ru­(bpy)_3_^2+*^, which underwent a SET process with the diazonium salt to afford the corresponding aryl radical intermediate. Because this aryl radical did not possess sufficient oxidation potential to be oxidized directly by the Ru­(III) complex, it first reacted with carbon monoxide to generate the more readily oxidizable acyl radical species. Subsequent incorporation of water, following oxidation to the corresponding acyl cation intermediate **551**, led to the formation of the anticipated aryl carboxylic acid **550**. Notably, the reaction proceeded efficiently under irradiation with blue LED when 20 mol % methanesulfonic acid and 1.5 equiv of *tert*-butyl nitrite were added to a solution of aniline in acetonitrile.

**Scheme 69:**
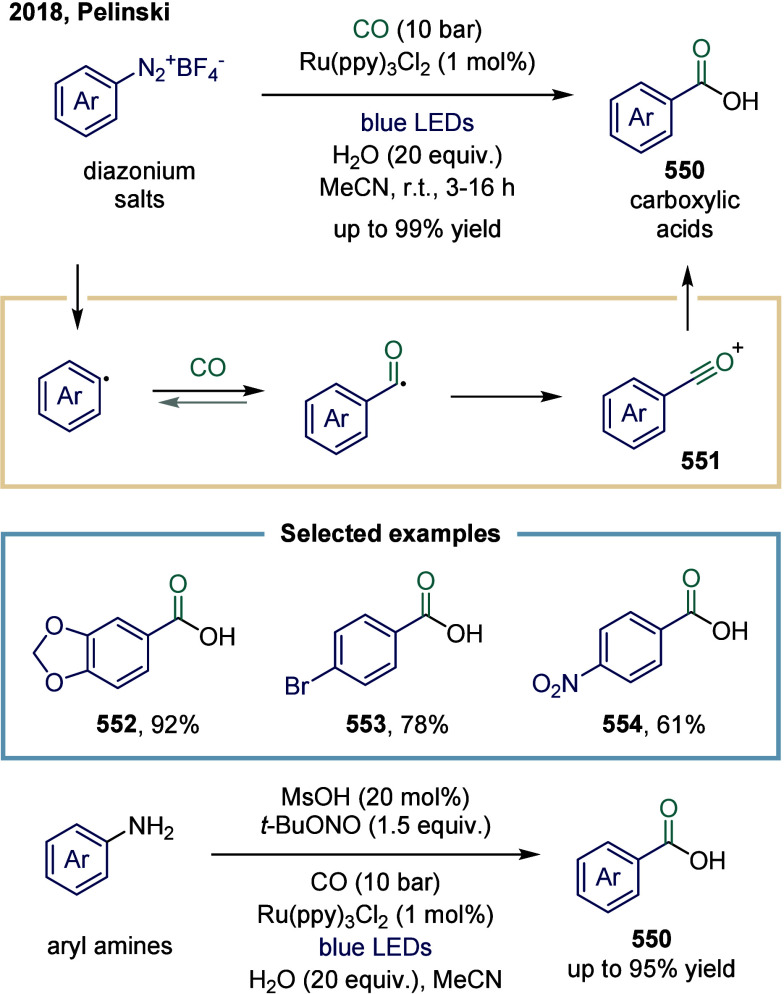
Visible-Light-Mediated Hydroxycarbonylation of Diazonium Salts

### 3.2 Rhodium-Catalyzed System

The selective transformation of CO into valuable formamide derivatives represents a significant challenge in organometallic catalysis. In 2018, Fang and co-workers reported a novel porphyrin rhodium­(II) metalloradical strategy that utilized the photochemical generation of [(por)­Rh­(CO)]•, a rhodium porphyrin carbonyl radical species (Scheme 70).^342^ Unlike conventional π-back bonding approaches, this single-electron pathway enabled the conversion of CO into reactive acyl-like intermediates under mild conditions. The proposed mechanism involved photoinduced homolysis of (por)­Rh­(CO)_2_ to generate [(por)­Rh­(CO)]• radicals **556**, which subsequently reacted stepwise with amines to yield (por)­Rh–C­(O)-NR_2_ intermediates **557**. Through a sequence of hydrogen atom transfer and reductive elimination steps, formamides were produced with excellent atom economy and without sacrificial reagents. Notably, turnover numbers (TON) of up to 224 were achieved for aliphatic amines **558**. This process exemplified an efficient tandem catalytic cycle and provided a foundation for designing one-electron-based carbonylation strategies. This work not only advanced CO utilization but also enhanced the mechanistic understanding of metalloradical pathways in organometallic chemistry.

**Scheme 70:**
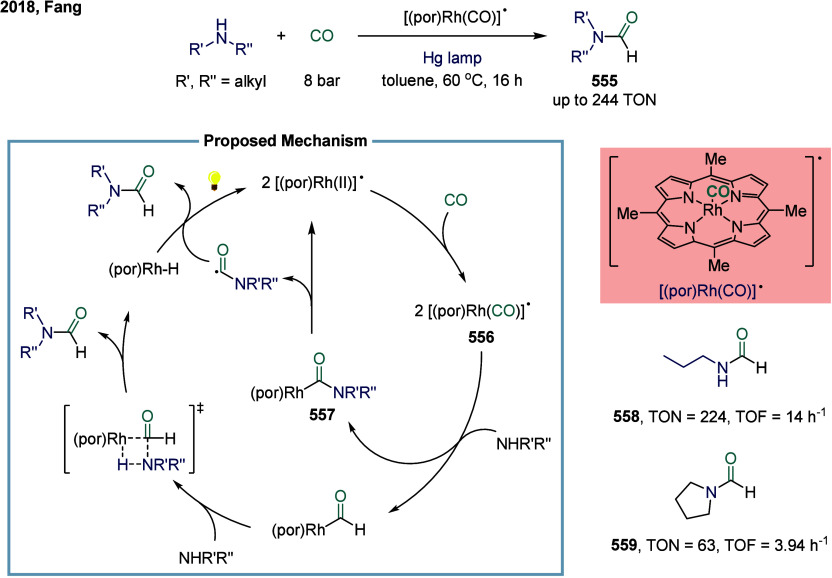
Production of Formamides from CO and Amines Induced by Porphyrin Rhodium­(II) Metalloradical

In 2018, Wu and co-workers developed a rhodium-catalyzed [3 + 2 + 1] cyclization of aromatic sulfides, terminal alkynes, and carbon monoxide for the efficient synthesis of 3-substituted thiochromenones (Scheme 71).^343^ This strategy provided a modular and convergent route to sulfur-containing heterocycles through a radical-mediated carbonylative annulation pathway. Mechanistically, a single-electron transfer (SET) process initiated the reaction by generating a thiophenol radical and a Rh­(II) species. The thiophenol radical selectively added to the terminal alkyne to give a vinyl radical intermediate **561**, which underwent a second SET with Rh­(II) to furnish the organorhodium intermediate **562**. Subsequent coordination of carbon monoxide to this Rh­(III) species enabled the formation of a seven-membered rhodacycle. Reductive elimination from this metallacycle then delivered the target thiochromenone scaffold, regenerating the catalytically active Rh­(I) species. Notably, the protocol exhibited good substrate scope and functional group compatibility, accommodating both alkyl and aryl alkynes, which furnished products **563** and **564** in 71% and 63% yields, respectively.

**Scheme 71:**
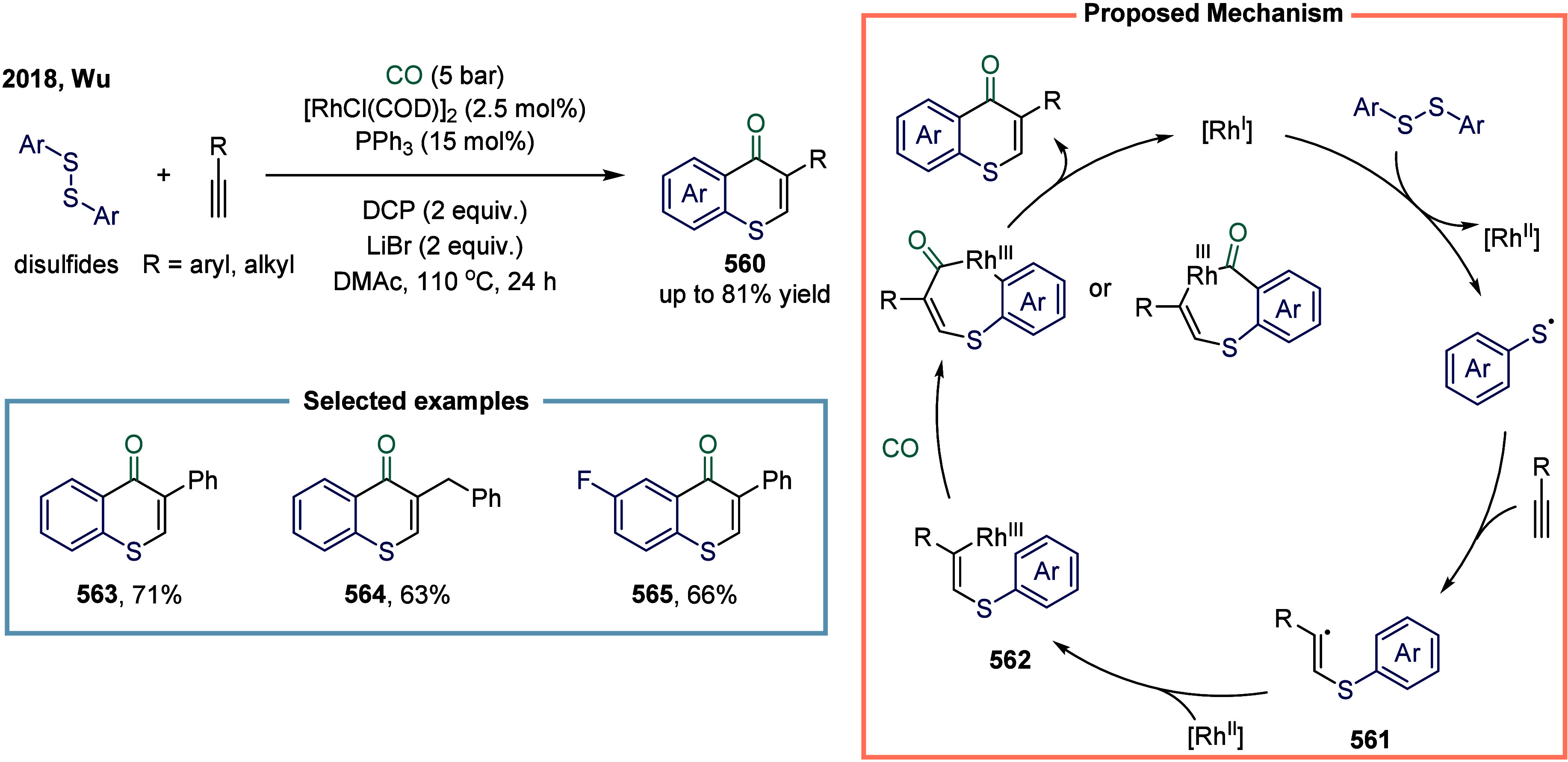
Carbonylative Synthesis of 3-Substituted Thiochromenones via Rhodium-Catalyzed [3 + 2 + 1] Cyclization of Different Aromatic Sulfides, Alkynes, and CO

Building upon this rhodium-catalyzed radical carbonylation framework, Li and co-workers reported a reductive trans-alkylacylation of internal alkynes via a formal carborhodation/C–H carbonylation cascade in 2021 (Scheme 72).^344^ In this reaction, homolytic cleavage of the C–Br bond in the alkyl bromide precursor generated the alkyl radical intermediate **567**. The catalytically active Rh­(I) species underwent single-electron oxidation by intermediate **567** to generate the C­(sp^3^)–Rh­(II) intermediate **568**. This species then participated in antiselective carborhodation across the alkyne to afford the vinyl–Rh­(II) intermediate **569**. Subsequent insertion of carbon monoxide into the Rh–C bond led to the formation of a six-membered cyclorhodium carbonyl intermediate **570**. Reductive elimination from this intermediate furnished the final trans-alkylacylated product. However, the reaction showed limited tolerance for certain alkyl halides, as primary and secondary bromides (**574–575**) failed to deliver the desired products under the standard conditions, likely due to unfavorable radical stability or inefficient oxidative addition.

**Scheme 72:**
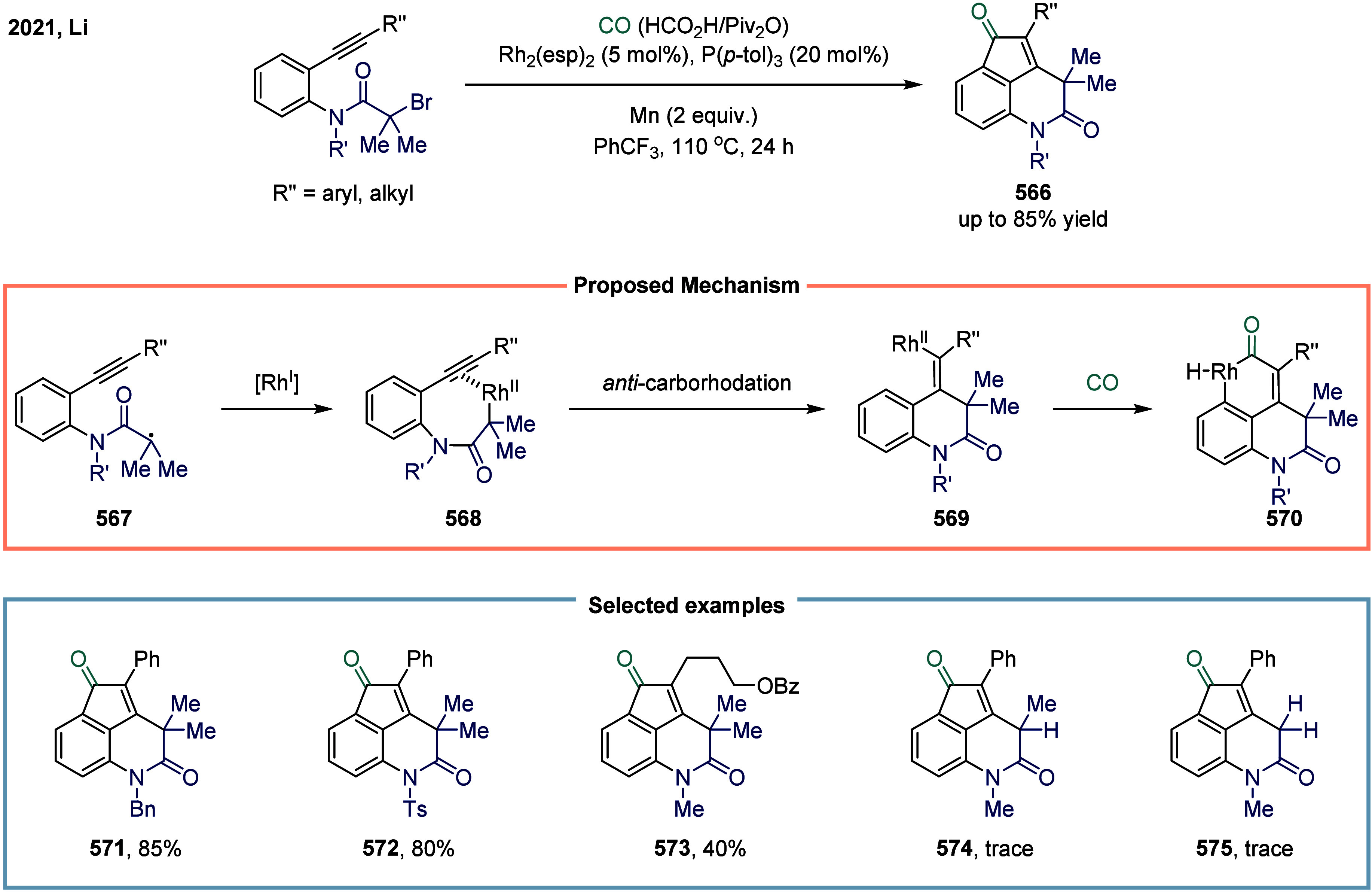
Rhodium-Catalyzed Reductive trans-Alkylacylation of Internal Alkynes via a Formal Carborhodation/C–H Carbonylation Cascade

### 3.3 Palladium-Catalyzed System

#### 3.3.1 Carbon–Hydrogen Bonds

Although significant advances have been made in transition-metal-catalyzed C–H carbonylative coupling reactions, the efficient incorporation of carbon monoxide into benzylic C–H bonds remains a challenging and underdeveloped area. In 2012, Huang and co-workers reported a palladium-catalyzed benzylic C–H carbonylation of aryl alkanes with alcohols, enabling the synthesis of 2-phenylacetic acid derivatives in yields of up to 76% and achieving turnover numbers (TON) as high as 288 (Scheme 73a).^345^ Di-*tert*-butyl peroxide was identified as the optimal oxidant, generating the corresponding oxygen radical via homolytic cleavage of the peroxide bond. The resulting oxygen radical abstracted a hydrogen atom from benzylic C–H bonds through a HAT process to afford the key benzylic radical. In the presence of a palladium catalyst, this benzylic radical combined with palladium to form a carbon–palladium bond, delivering the organopalladium species **577**. The steric bulk of complex **577** retarded CO insertion to generate intermediate **578**, thereby favoring exclusive formation of the less hindered intermediate **579** via anion exchange. Subsequent CO insertion into the carbon–palladium bond afforded the acyl-palladium intermediate, which underwent reductive elimination to furnish the final carbonylation product and regenerate the active palladium species for the next catalytic cycle. The corresponding *tert*-butyl ester was also formed as a minor product via intermediate **578**. A range of 2-phenylacetic acid derivatives bearing diverse substituents on the aromatic ring, such as compounds **580**, **581**, and **582**, was successfully synthesized in moderate to good yields from inexpensive and commercially available starting materials. Notably, ethylbenzene was selectively converted to the corresponding methyl-substituted product **583** in 26% yield, corresponding to a TON of 82. In 2013, the same group also reported a palladium-catalyzed oxidative aminocarbonylative coupling of benzylic C–H bonds with amines to access amides **584** in up to 85% yield (Scheme 73b).^346^

**Scheme 73:**
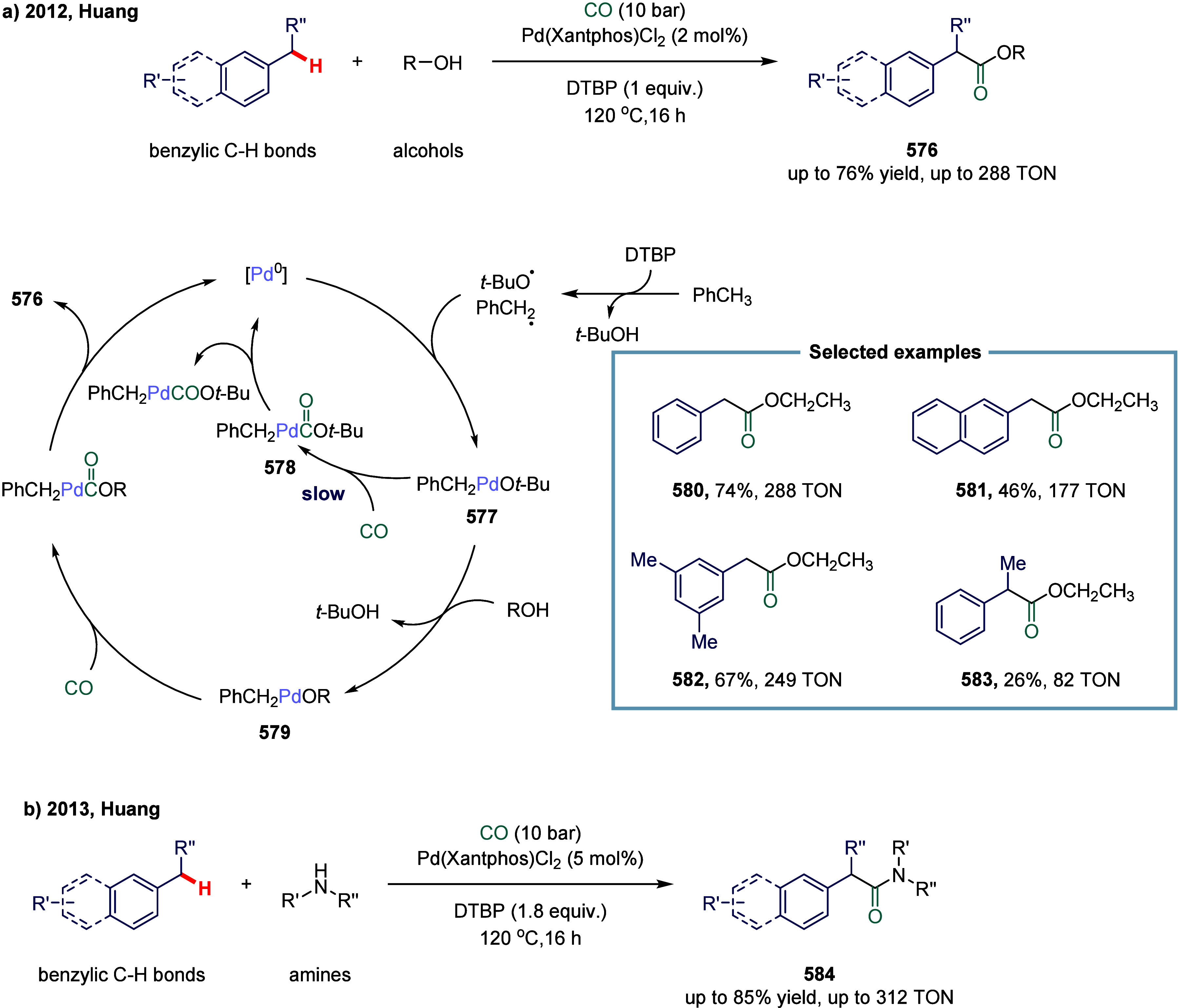
Palladium-Catalyzed Oxidative Carbonylation of Benzylic C–H Bonds

Double C–H bond activation provides an efficient strategy for constructing cyclic frameworks and represents a powerful tool for synthesizing high-value molecular architectures. However, cyclization reactions involving the activation of two C–H bonds at one carbon atom remain exceedingly rare. In 2023, Huang and co-workers reported a formal carbonylative cycloaddition between alkylarenes and imines, enabled by dual benzylic C­(sp^3^)-H bond activation at one carbon center (Scheme 74).^347^ This protocol enabled the direct synthesis of β-lactams **585** from inexpensive and readily available alkylarenes and imines in up to 94% yield. The reaction was initiated by the homolytic cleavage of DTBP, producing two *tert*-butoxy radicals. One of these radicals abstracted a benzylic hydrogen atom to generate a benzylic radical, which subsequently underwent stepwise oxidation by Pd(0) to form a benzyl-palladium species **586**. This complex then underwent CO insertion to yield an acyl-palladium intermediate **587**. Alternatively, the benzylic radical could directly capture CO to generate a phenylacetyl radical **588**, which then combined with complex **589** to furnish the same acyl-palladium intermediate. Cleavage of the second benzylic C­(sp^3^)-H bond was facilitated either by the counteranion (*t*-BuO^–^) associated with the palladium complex or by an added base, leading to the formation of a palladium-ketene intermediate **590**. Nucleophilic addition of the imine to this intermediate afforded a palladium-coordinated zwitterionic species **591**, which subsequently underwent isomerization and palladium-promoted cyclization to generate the desired β-lactam **585** and regenerate the Pd(0) catalyst. Aldimines derived from both pyridylaldehyde and benzaldehyde were compatible with this transformation, affording β-lactam products **592**-**594** in good yields. In addition to aliphatic imines, aryl imines were also evaluated, yielding β-lactam **595** in relatively low yield. Notably, imines derived from *p*-toluenesulfonamide (TsNH_2_) failed to produce the desired β-lactam **597**, likely due to the low nucleophilicity of the sulfonamide-derived imine nitrogen.

**Scheme 74:**
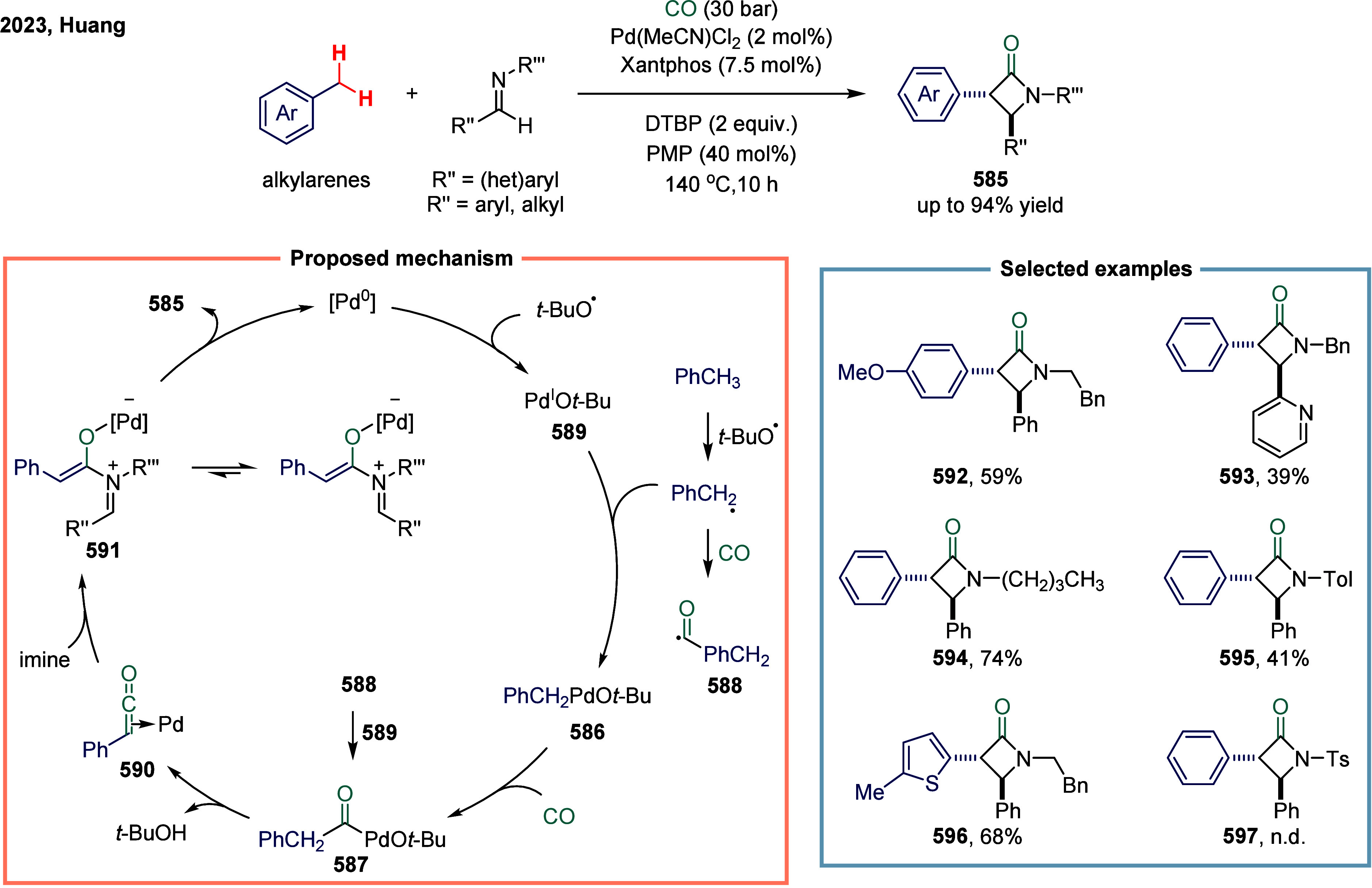
Carbonylative Formal Cycloaddition between Alkylarenes and Aldimines Enabled by Palladium-Catalyzed Double C–H Bond Activation

In 2016, Lei and co-workers reported a palladium complex bearing PPh_3_ as a ligand that enabled highly efficient alkoxycarbonylation of alkanes, affording products **598** in yields up to 94%. (Scheme 75).^348^ This transformation was conducted under a carbon monoxide pressure of 5 atm. Notably, under these pressure conditions, the challenging C­(sp^3^)-H functionalization of ethane was successfully accomplished, affording benzyl propionate as the product **599** in 61% yield. However, despite this achievement, the selectivity for longer-chain alkanes remained suboptimal due to the inherent challenges associated with the HAT mechanism employed in the reaction.

**Scheme 75:**
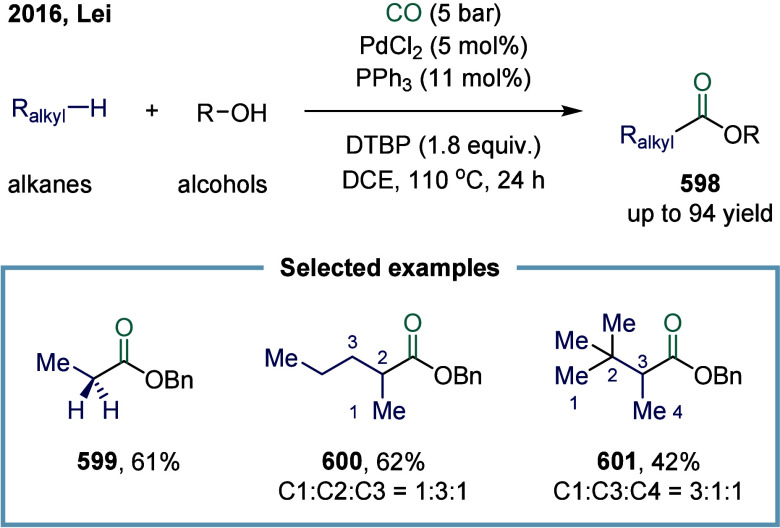
Palladium-Catalyzed Radical Oxidative Alkoxycarbonylation of Alkanes to Prepare Numerous Alkyl Carboxylates

In 2025, Huang and co-workers reported a highly site-selective C­(sp^3^)-H carbonylation strategy enabled by implementing a radical single-out strategy (Scheme 76).^349^ This method leveraged the steric sensitivity of the CO insertion step as a key differentiating element in radical pathways. Mechanistically, the transformation involved the sequential activation of two allylic C­(sp^3^)-H bonds and enabled a carbonylative formal [2 + 2] cycloaddition between imines and alkenes. The reaction was initiated by an allylic HAT process, followed by SET to generate a η^3^-π-allyl palladium intermediate **602** and **603**. Subsequent rapid CO insertion furnished the key acyl-palladium species **604** and **605**. Notably, primary allylic radicals, owing to their reduced steric hindrance, underwent carbonylation with significantly lower activation barriers compared to secondary or tertiary radicals. Monosubstituted alkenes participated smoothly in the reaction, affording the corresponding *trans*-β-lactams **608** in 61% yield. In addition, 1,2-disubstituted aryl internal alkenes were well tolerated, undergoing dual allylic C­(sp^3^)-H bond carbonylation at the same carbon center to afford β-lactam product **609** in 61% yield. Even tri- and tetra-substituted alkenes proved compatible, delivering the desired β-lactams in moderate yields with excellent site-selectivity, such as compounds **609**, **611**, **614**, and **615**. This strategy preferentially functionalized sterically less hindered primary C­(sp^3^)-H bonds, even in the presence of more reactive secondary or tertiary sites, and demonstrated broad compatibility with structurally diverse substrates.

**Scheme 76:**
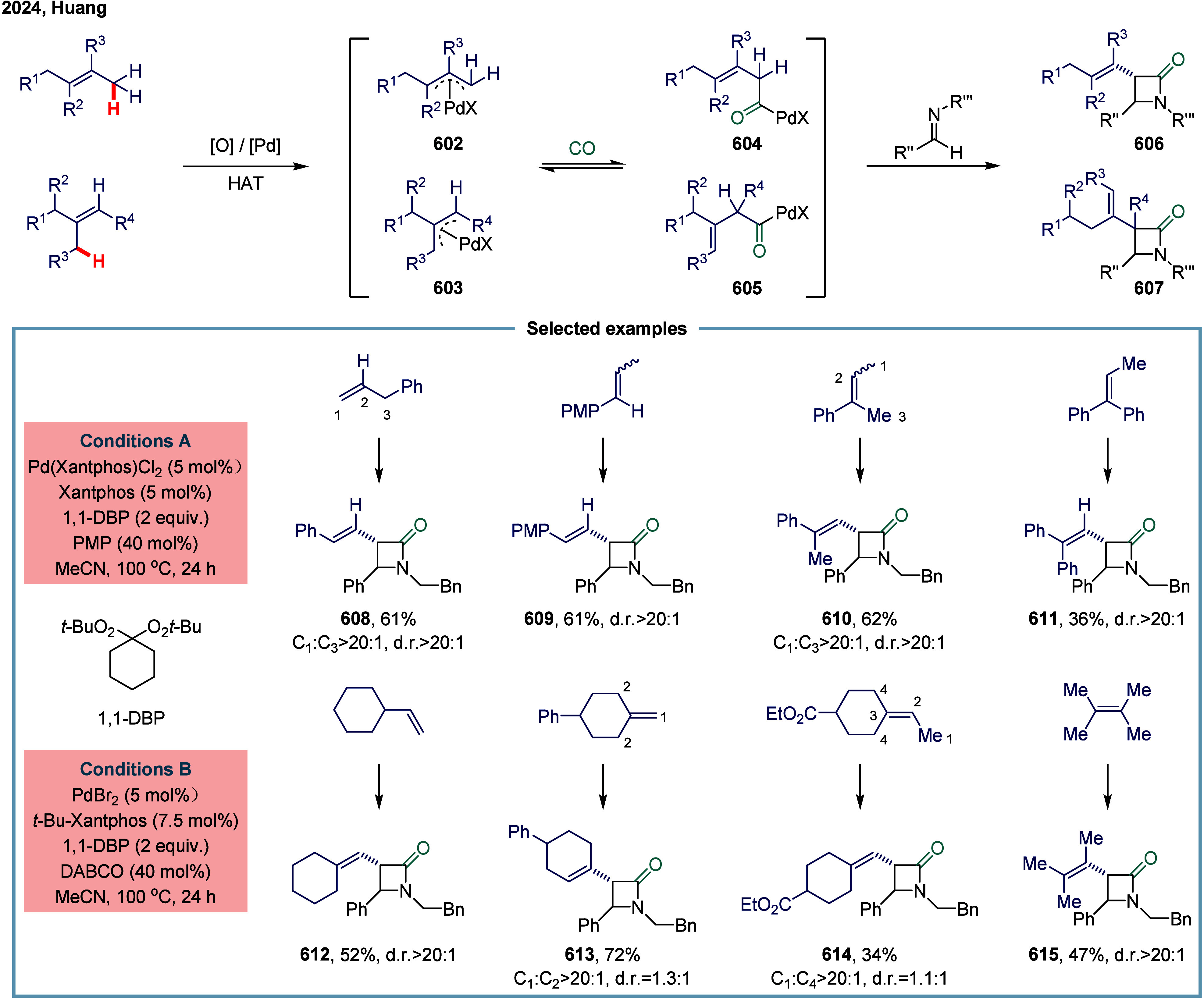
Site-Selective Carbonylative Cyclization with Two Allylic C–H Bonds Enabled by Radical Differentiation

#### 3.3.2 Carbon–Halogen Bonds

Palladium-catalyzed carbonylation reactions provide an efficient and atom-economical approach for constructing carbonyl-containing compounds in organic synthesis. However, compared to aryl halides, the carbonylation of alkyl halides remains significantly more challenging due to the reduced stability of the corresponding palladium intermediates. This difficulty is further exacerbated in the case of activated alkyl halides, where competing nucleophilic substitution pathways often predominate over the desired carbonylation process.^350^ In 2012, Ryu and co-workers accomplished a mild and efficient carbonylative coupling of α-iodoacetate and amines under palladium-light combined conditions, generating carbamoylacetates **616** up to 87% yield (Scheme 77).^351^ Carbamoylacetates function as valuable intermediates in the synthesis of biologically active heterocyclic frameworks.^352,353^ The authors proposed that this reaction likely proceeded through an interplay of radical and organopalladium species. Specifically, the acetate radical was initially generated from α-iodoacetate via cleavage of the C–I bond, a process that was presumably triggered by SET process from the photoexcited palladium complex. Supporting evidence for this step was provided in related studies.^354,355^ Subsequently, coupling of the acetate radical with palladium afforded the key α-pallado ester intermediate **617**, which underwent CO insertion to deliver the target product **616**. Notably, when pyrrole was employed in the reaction, the corresponding half-ester of malonic acid **619** was obtained in 52% yield.

**Scheme 77:**
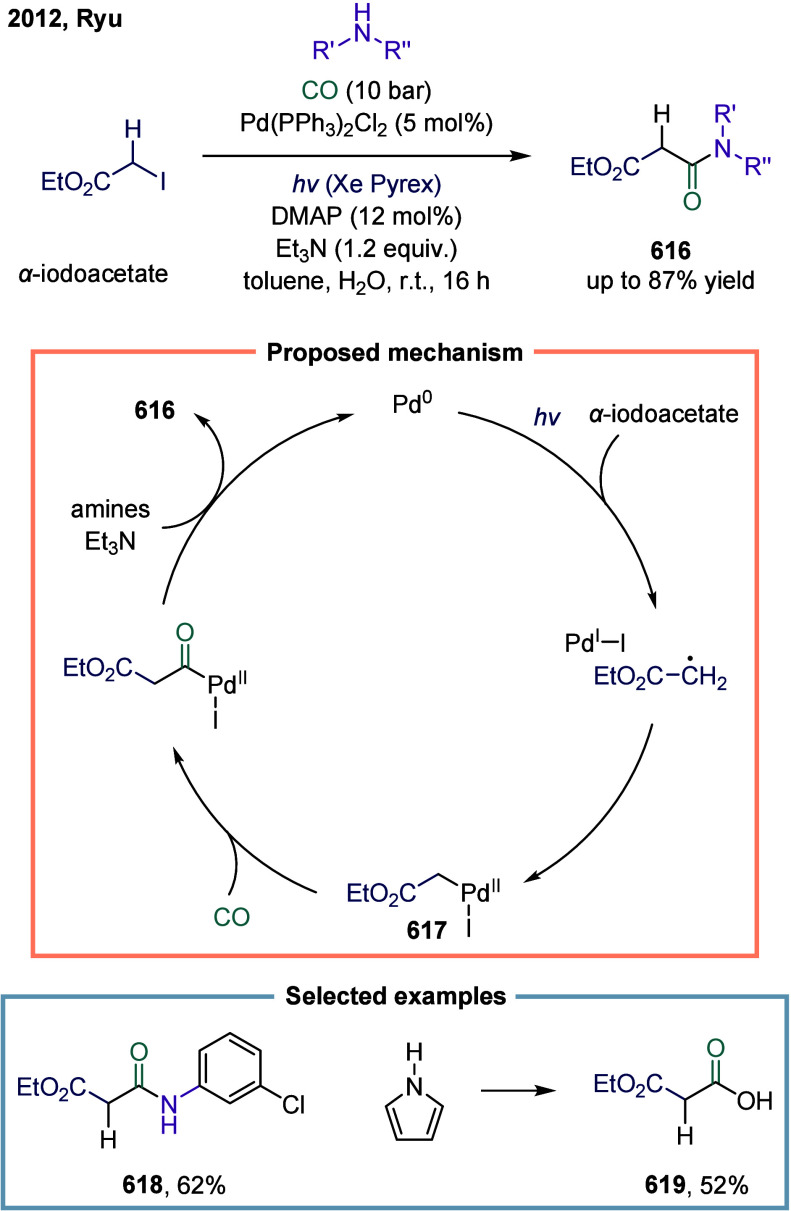
Synthesis of Carbamoylacetates from α-Iodoacetate, CO, and Amines under Pd/Light Combined Conditions

Subsequently, the same group extended this transformation into various α-iodo esters with arylboronic acids for the synthesis aromatic β-keto esters **620** (Scheme 78).^356^ This carbonylative Suzuki-Miyaura coupling provided two distinct catalytic protocols for accessing a broad range of β-keto esters, namely a palladium-light-induced system and a palladium-thermal-induced system. In comparison, 2-iodooctane afforded the keto ester **621** in 34% yield exclusively under thermal conditions, while no desired product was obtained under photochemical conditions. The failure of the reaction under irradiation was attributed to the propensity of linear iodides to undergo E2 elimination. Notably, this carbonylative Suzuki–Miyaura coupling was successfully extended to iodomethyl phenyl sulfone, delivering the α-sulfonyl acetophenone **622**. Under photochemical conditions, the reaction proceeded sluggishly and afforded a moderate yield, whereas heating at 80 °C enabled the formation of **622** in 91% yield.

**Scheme 78:**
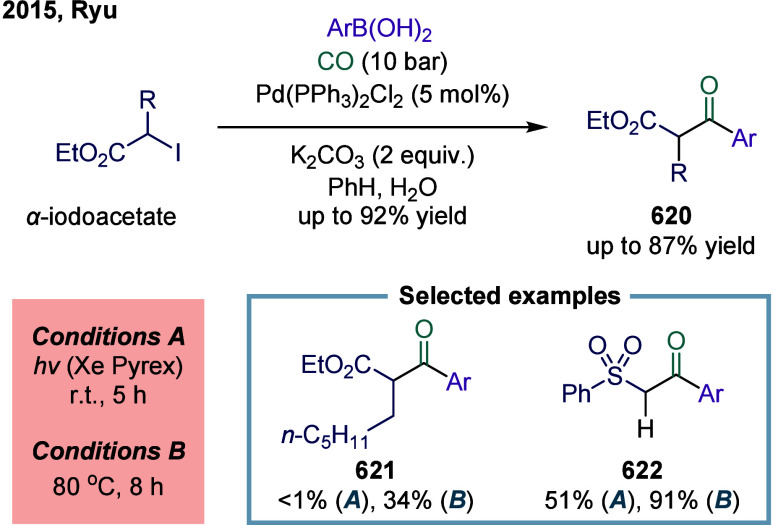
Synthesis of Aromatic *β*-Keto Esters via a Carbonylative Suzuki–Miyaura Coupling Reaction of *α*-Iodo Esters with Arylboronic Acids

In 2016, Skrydstrup and co-workers described palladium-catalyzed carbonylation of (hetero)­aryl boronic acid derivatives to access *α,α*-difluoroacylated arenes **623** in up to 99% yield (Scheme 79a).^357^ In this setup, CO gas was generated in a separate reaction chamber from 9-methyl-*9H*-fluorene-9-carbonyl chloride, which was employed in slight excess (1.6 equiv).^358,359^ The method primarily utilized starting materials that were either commercially available or could be accessed in a few straightforward synthetic steps, enabling the synthesis of a diverse range of fluorinated small molecules. Mechanistic studies provided evidence that radicals were generated at the fluorinated carbon via a SET process. Under standard coupling conditions, the reaction of 3-pyridineboronic acid neopentylglycol ester afforded the corresponding *α,α*-difluorinated β-ketoamide **624** in a moderate 30% yield. Secondary amides **625** and alkyl esters **626** were also compatible substrates, delivering products in high yields. Furthermore, a variety of (hetero)­aryl boronates and boronic acid salts were successfully converted into (hetero)­aryl *α,α*-difluoro-β-ketoamides and *α,α*-difluoro-β-ketoesters with good efficiency. In the same year, the Zhang group reported back-to-back studies on palladium-catalyzed carbonylative coupling of difluoroalkyl bromides (Scheme 79b).^360^ The reaction proceeded efficiently under mild carbon monoxide conditions, displayed a broad substrate scope, and exhibited excellent tolerance toward diverse functional groups. This protocol provided a versatile and practical approach for the synthesis of a wide range of fluorinated compounds **626**, underscoring its synthetic utility. Although the authors attributed the notable performance of Xantphos in this catalytic system to its large bite angle, this feature was not deemed essential for the observed reactivity. Further investigations into the reaction mechanism and related derivative transformations were in progress in the Zhang group.

**Scheme 79:**
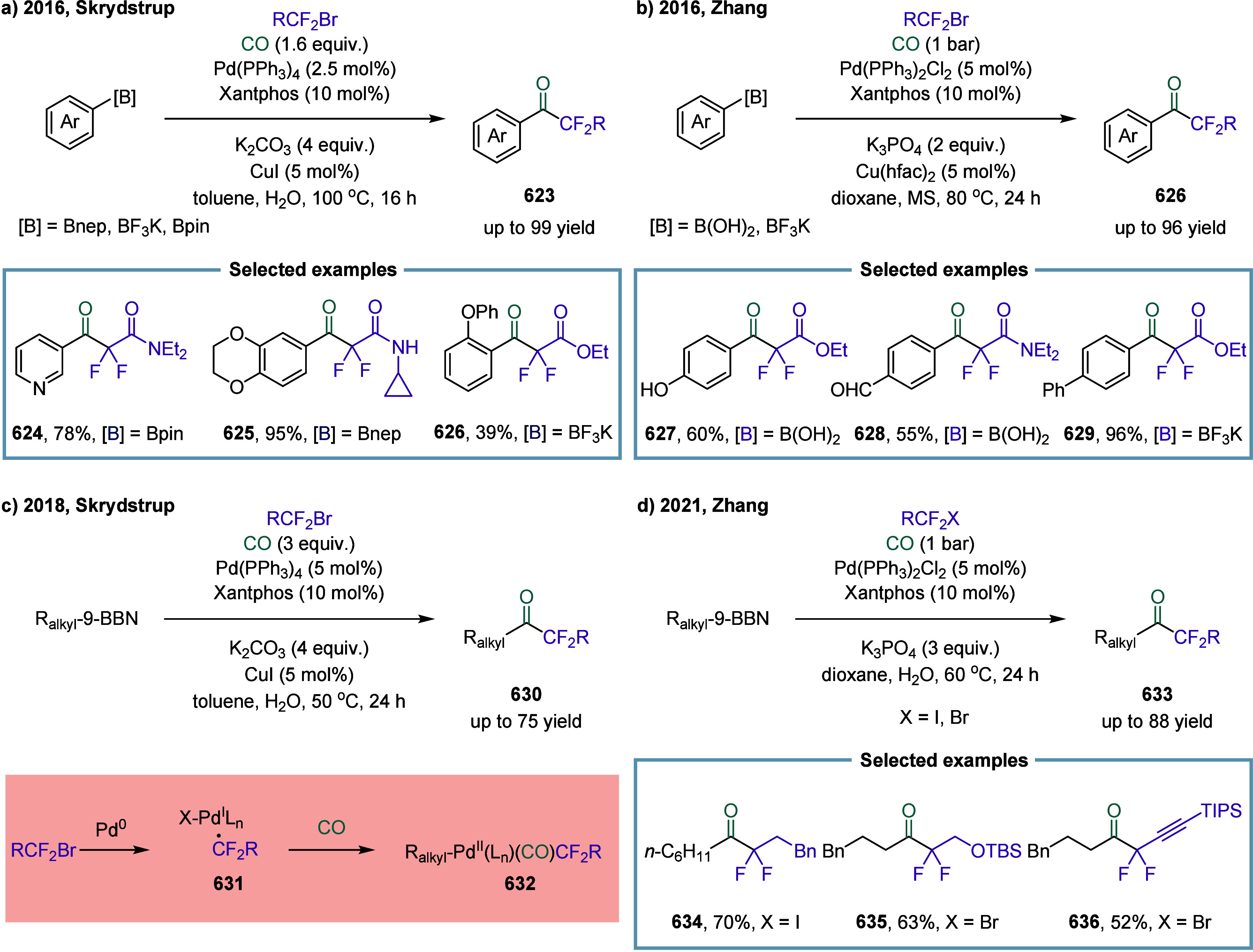
Palladium-Catalyzed Carbonylative Coupling of Organic Boron Reagents with Fluoroalkyl Halides

In 2018, carbonylative Suzuki coupling of alkylboron reagents and bromodifluoroacetamides with COgen as the CO source was described by Skrydstrup group (Scheme 79c).^361^ Detailed mechanistic studies suggested that halide abstraction by Pd^0^ generated a carbon-centered radical **631** along with a Pd^I^ complex. Subsequently, radical **631** was combined with the Pd^I^ complex under a CO atmosphere to form a CO-ligated species, which was then converted into an acyl Pd^II^ complex. Transmetalation with alkyl-9-BBN afforded the key acyl-Pd^II^ intermediate **632**, which underwent reductive elimination to deliver the desired difluoroketoamide. Alternatively, complex **632** might also have formed via the direct combination of radical **631** with a CO-coordinated Pd^I^ species. The reaction enabled a successful extension from aryl boronate to alkyl boron reagents, providing access to a range of *α,α*-difluoro-β-alkyl-β-ketoamides. Later, Zhang and co-workers achieved a palladium-catalyzed carbonylation cross-coupling of difluoroalkyl halides with alkylboranes, generating alkyldifluoroalkyl ketones **633** in up to 88% yield (Scheme 79d).^362^ Alkyl-substituted difluoroalkyl bromides, as well as difluoroalkyl bromides bearing π-systems, such as compounds **634**-**636**, were also compatible with this reaction, demonstrating the broad applicability of the method. These studies demonstrated that palladium-catalyzed carbonylative couplings of difluoroalkyl halides with (hetero)­aryl or alkyl boron reagents proceeded via similar mechanisms involving carbon-centered radicals and Pd^I^ intermediates. Skrydstrup’s group developed efficient methods employing ex situ CO generation and expanded carbonylative couplings to include alkylboron reagents. In parallel, Zhang’s group established practical protocols for the carbonylation of difluoroalkyl bromides under mild conditions, featuring broad substrate scope and excellent functional group tolerance.

Besides the processes involving the carbonylative coupling of fluoroalkyl halides with alkyl boron reagents, alcohols and amines could also participate in the carbonylation transformation of fluoroalkyl halides. In 2024, a palladium-catalyzed direct carbonylative coupling of (fluoro)­bromoacetates for the synthesis of fluoro substituted malonates was reported by Wu and co-workers (Scheme 80a).^363^ Using PdI_2_ as the catalyst and Xantphos as the ligand, various malonate, monofluoromalonate, and difluoromalonate derivatives **637** were synthesized in up to 99% yield. This palladium-catalyzed strategy provided a novel approach for the synthesis of (fluorine)­malonate derivatives **638–640**, effectively circumventing the over fluorination issues encountered in previous methodologies. Later, a general palladium-catalyzed carbonylative coupling of trifluoroethane iodide was disclosed by Wu and co-workers in 2024, leading to a class of α-CF_3_-substituted ketones and carboxylic acid derivatives **641** in good yield (up to 95% yield) (Scheme 80b).^364^ In this study, the high bond dissociation energy of the Csp^3^-X bond was leveraged to enable the efficient synthesis of α-trifluoromethyl-substituted ketones and carboxylic acid derivatives up to 99% yield via palladium-catalyzed activation involving radical intermediates. Under standard reaction conditions, phenols and alcohols were employed as substrates to afford the corresponding ester products **642**. When more nucleophilic arylamines or alkylamines were used, the reaction proceeded smoothly to yield the desired amides **643** with high efficiency. Notably, substrates with lower nucleophilicity, such as sulfonamides and amides, were successfully converted into the corresponding imide compounds in excellent yields. Furthermore, the use of more challenging carbon nucleophiles, such as arylboronic acids, still afforded the α-trifluoromethyl-substituted ketones **644** in good yields. The authors proposed that the reaction between trifluoroiodoethane and Pd^0^ proceeded via a SET pathway involving a carbon radical intermediate, which subsequently generated a divalent palladium complex intermediate to facilitate subsequent transformations.

**Scheme 80:**
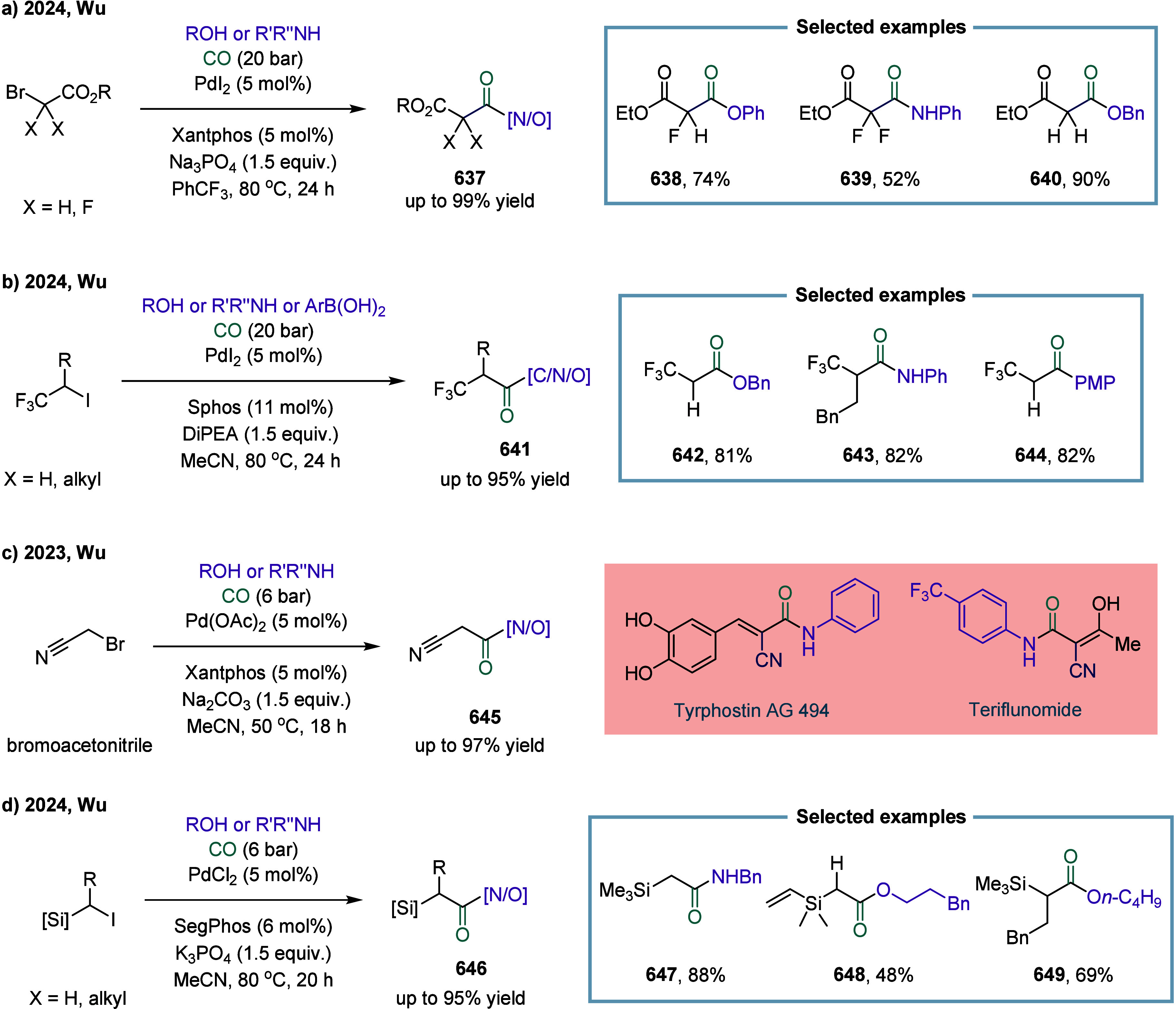
Palladium-Catalyzed Carbonylation of Active Alkyl Halides

Compounds such as 2-cyano-*N*-acetamide and 2-cyanoacetates, containing two functional groups, play a significant role in the pharmaceutical industry. These versatile building blocks serve as key precursors for the direct synthesis of diverse pharmaceutically active compounds.^365–367^ An elegant and efficient synthesis of 2-cyano-*N*-acetamide and 2-cyanoacetate derivatives **645** was disclosed by Wu and co-workers in 2023 via palladium-catalyzed carbonylative coupling with bromoacetonitrile and alcohols or amines (Scheme 80c).^368^ Using this approach, a broad range of valuable 2-cyano-*N*-acetamide and 2-cyanoacetate derivatives were synthesized in excellent yields, exhibiting remarkable functional group tolerance. Furthermore, this transformation was conducted under atmospheric pressure, providing alternative synthetic routes to seven drug precursors, including Tyrphostin AG 494 and Teriflunomide. Mechanistic studies revealed that bromoacetonitrile underwent single-electron reduction by Pd^0^, generating a carbon-centered radical and Pd^I^ intermediates. Later in 2024, The same group achieved an efficient carbonylative process for the synthesis of versatile α-(silyl)­acetates **646** (Scheme 80d).^369^ α-(Silyl)­acetates represent a class of stable and versatile organosilicon compounds, wherein the intrinsic diversity of silicon enables access to reactivity via masked carbanions, carbon nucleophiles, carbon-centered radical intermediates, and silicon electrophiles.^370,371^ Alkyl- and alkenyl-substituted α-iodosilanes furnished the desired products **647** and **648** in 88% and 48% yields, respectively. Additionally, secondary α-bromosilane was successfully converted to the target product **649** in good yield. However, when phenylboronic acid was used as the carbon nucleophile, no desired ketone product was obtained.

Since Suzuki’s pioneering work on the palladium-catalyzed carbonylative cross-coupling reaction of alkyl iodides with 9-alkyl-9-BBN derivatives under irradiation with a 100 W unsmoked tungsten lamp (Scheme 81a),^372^ carbonylative coupling reactions have long been recognized for their remarkable synthetic utility in the synthesis of carbonyl-containing molecules **650** and **651**. In 2002, Ryu and co-workers reported an innovative synthetic approach for preparing five-membered cyclic keto esters and amides **652** via cyclizative multiple CO-trapping reactions (Scheme 81b).^373^ The strategy employed cascade reactions starting from 4-alkenyl iodides under conditions integrating radical initiation via irradiation and palladium catalysis and comprising five steps: homolysis, carbonylation, cyclization, a second carbonylation, and iodine atom transfer. Mechanistic investigations strongly suggested that the cascade involved both 5-exo-trig radical cyclizations and the formation of acylpalladium intermediates, likely arising from coupling between acyl radicals and Pd^I^ species. While the yields were excellent with iodides, bromides provided significantly lower yields. Later in 2006, Ryu and co-workers presented an in-depth study of atom transfer carbonylation (ATC) reactions of alkyl iodides accelerated by palladium catalyst and dimanganese decacarbonyl under photoirradiation (Scheme 81c).^374^ This work built on earlier ATC methods to address limitations, particularly the low reactivity of primary alkyl iodides. Traditional ATC methods proceeded efficiently with secondary and tertiary iodides but performed poorly with primary iodides due to slow iodine atom transfer and competing side reactions, such as decomposition and intramolecular S_N_2 processes. The author systematically investigated whether combining metal complexes, including palladium and Mn_2_(CO)_10_ with photoirradiation could accelerate these transformations. Under irradiation, primary alkyl iodides afforded the corresponding esters in 54%, 87%, and 54% yields under metal-free, palladium-catalyzed, and manganese-catalyzed conditions, respectively. When the reaction was conducted in the presence of amines, the palladium catalyst promoted the formation of double carbonylated products. The second CO incorporation proceeded via an acylcarbamoylpalladium intermediate rather than through further radical processes. Mn_2_(CO)_10_ favored single carbonylation, even in the presence of amines, by promoting atom transfer rather than palladium-mediated CO insertion.

**Scheme 81:**
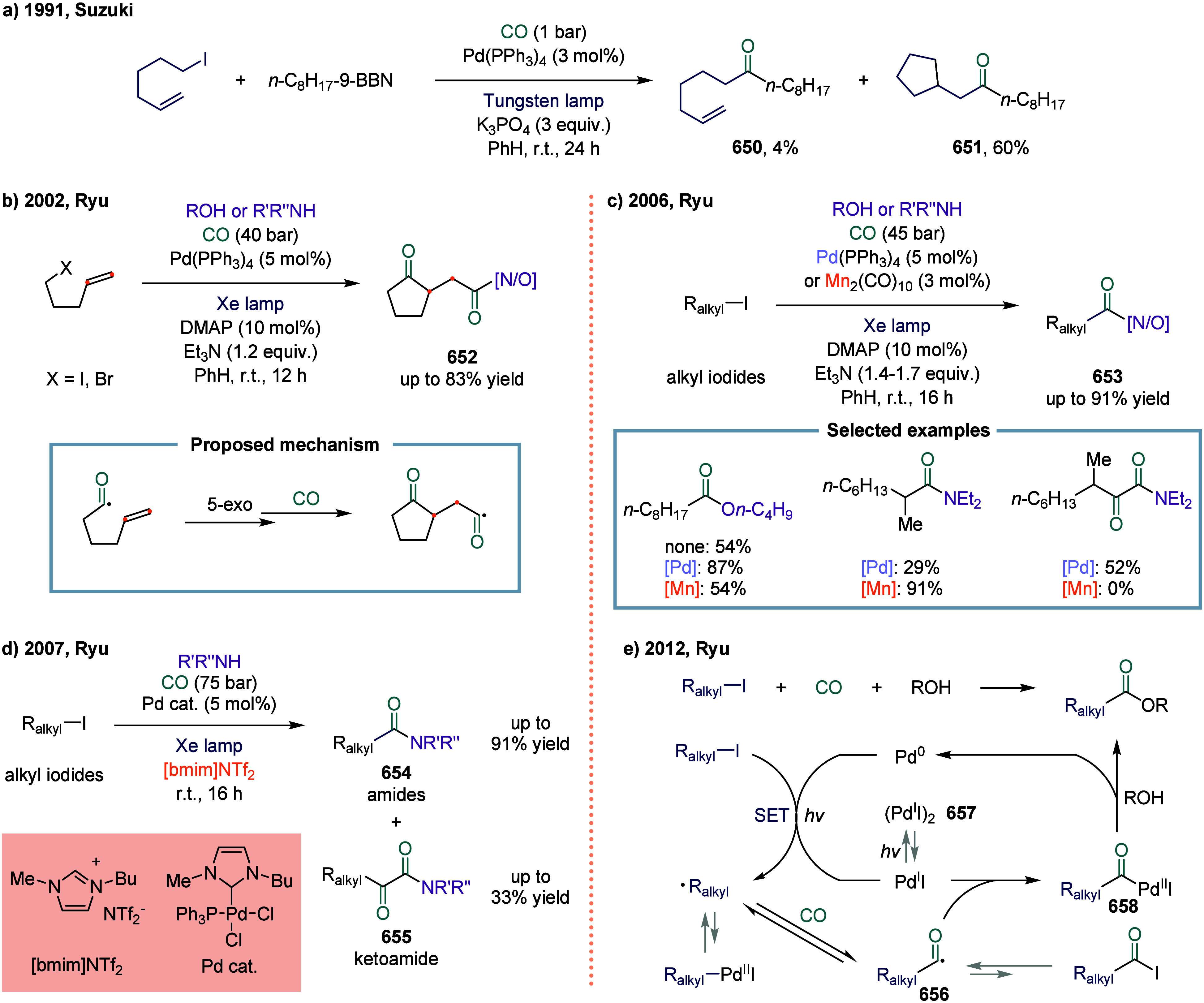
Palladium-Catalyzed Photo-Induced Carbonylation of Alkyl Halides: Synthesis of Amides and Esters

Ionic liquids have recently garnered significant attention as effective reaction media, offering an environmentally friendly alternative to volatile organic solvents without compromising solvent functionality.^375,376^ To date, a broad spectrum of reactions has been investigated in ionic liquids, including transition-metal-catalyzed cross-coupling reactions, enzymatic transformations, among others.^377–379^ In 2007, Ryu and co-workers explored the use of ionic liquids as green reaction media in ATC carbonylation of alkyl iodides with CO and amines to form amides **654**, catalyzed by a Pd-carbene complex under photoirradiation (Scheme 81d).^380^ This approach combined the advantages of ionic liquids, such as low volatility and recyclability, with transition-metal catalysis and SET-mediated carbonylation. The study employed two imidazolium-based ionic liquids, [bmim]­PF_6_ and [bmim]­NTf_2_, as solvents. In this reaction, the ionic liquids suppressed direct S_N_2 in some cases for example [bmim]­NTf_2_ with 1-iodooctane, thereby enhancing selectivity for carbonylation products. Furthermore, the Pd-carbene catalyst’s solubility in ionic liquids enhanced catalytic efficiency and recyclability. The corresponding amide product **654** was isolated in up to 91% yield, whereas the double carbonylation product, ketoamide **655**, was obtained in low yield. In 2012, Ryu and co-workers reported a significant advancement in the field of alkyl halide carbonylation, presenting a photoinduced ATC strategy that effectively converts a broad range of primary, secondary, and tertiary alkyl iodides into valuable carboxylic acid derivatives (Scheme 81e).^381^ The author proposed a mechanism for the palladium/light-assisted ATC of alkyl halides. The key step involved the generation of alkyl radicals from alkyl iodides via SET from the photoexcited Pd^0^ catalyst or from Pd species produced by homolysis of a Pd^I^ dimer complex **657** under irradiation. These Pd species abstracted iodine atoms from alkyl iodides, generating alkyl radicals. The resulting alkyl radical rapidly added to CO, forming an acyl radical intermediate **656**. This acyl radical was subsequently trapped by Pd^I^ species to yield an acylpalladium intermediate **658**, which underwent further transformation to afford the final carbonylated product. In the presence of amines, two molecules of CO were incorporated to produce keto amides via an acylcarbamoylpalladium complex, a pathway well documented in Pd-catalyzed double carbonylation. The radical/palladium hybrid mechanism effectively circumvented common challenges in alkyl halide carbonylations, such as slow oxidative addition and β-hydride elimination.

In 2013, Ryu and co-workers reported a methodology for synthesizing alkyl aryl ketones **658** via the carbonylative cross-coupling of alkyl iodides with arylboronic acids under combined palladium catalysis and photoirradiation conditions (Scheme 82a).^382^ The alkyl radical rapidly captured CO to form acylpalladium intermediates, which, upon subsequent transmetalation with arylboronic acids, yielded acyl­(aryl)­palladium complexes. The hybrid catalytic system efficiently generated alkyl aryl ketones **658** with broad substrate scope and operational simplicity, offering an appealing alternative to traditional carbonylation strategies that relied on aryl halides or sensitive organometallic reagents. Later in 2017, a visible-light mediated palladium-catalyzed carbonylative Suzuki-Miyaura coupling of unactivated alkyl iodides and bromides with aryl boronic acids was disclosed by Odell and co-workers (Scheme 82b).^383^ The method employed molybdenum hexacarbonyl (Mo­(CO)_6_) as a solid CO source, circumventing the use of gaseous CO through a double-chamber reaction setup. A variety of alkyl aryl ketones **659** were prepared in yields of up to 83% from readily available alkyl iodides and bromides.

**Scheme 82:**
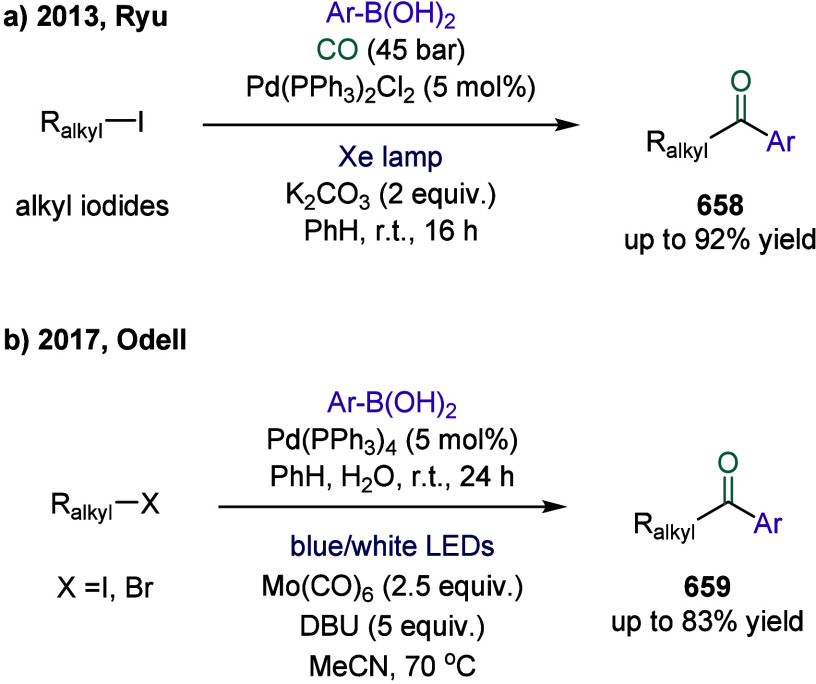
Palladium-Catalyzed Photo-Induced Carbonylation of Alkyl Halides toward Ketone Synthesis

In 2010, a three-component carbonylative coupling approach for the synthesis of alkyl alkynyl ketones **660** from alkyl iodides, CO, and terminal alkynes under visible light and palladium catalysis was reported by Ryu and co-workers (Scheme 83a).^384^ Alkynyl ketones are important motifs in biologically active molecules and serve as valuable intermediates in natural product synthesis and heterocyclic chemistry.^385,386^ In 2015, Ryu and co-workers reported a carbonylative Mizoroki-Heck reaction enabling the synthesis of *α,β*-unsaturated ketones **661** from alkyl halides (Scheme 83b).^387^ While carbonylative Mizoroki-Heck reactions traditionally employed aryl or vinyl halides, this methodology utilized alkyl radicals generated from alkyl halides via a SET process. These alkyl radicals rapidly added CO to form acyl radicals, which subsequently added to alkenes, yielding β-keto radical intermediates. This work represented the first example of an intermolecular carbonylative Mizoroki-Heck reaction of alkyl halides under mild photoirradiation conditions. The use of a radical mechanism effectively circumvented challenges associated with the instability of alkyl-Pd intermediates in conventional Heck reactions. A variety of substrates, including mono- and disubstituted aromatic olefins as well as acrylates, were successfully transformed to yield disubstituted and trisubstituted *α,β*-unsaturated ketones **662**, **663**, **664**.

**Scheme 83:**
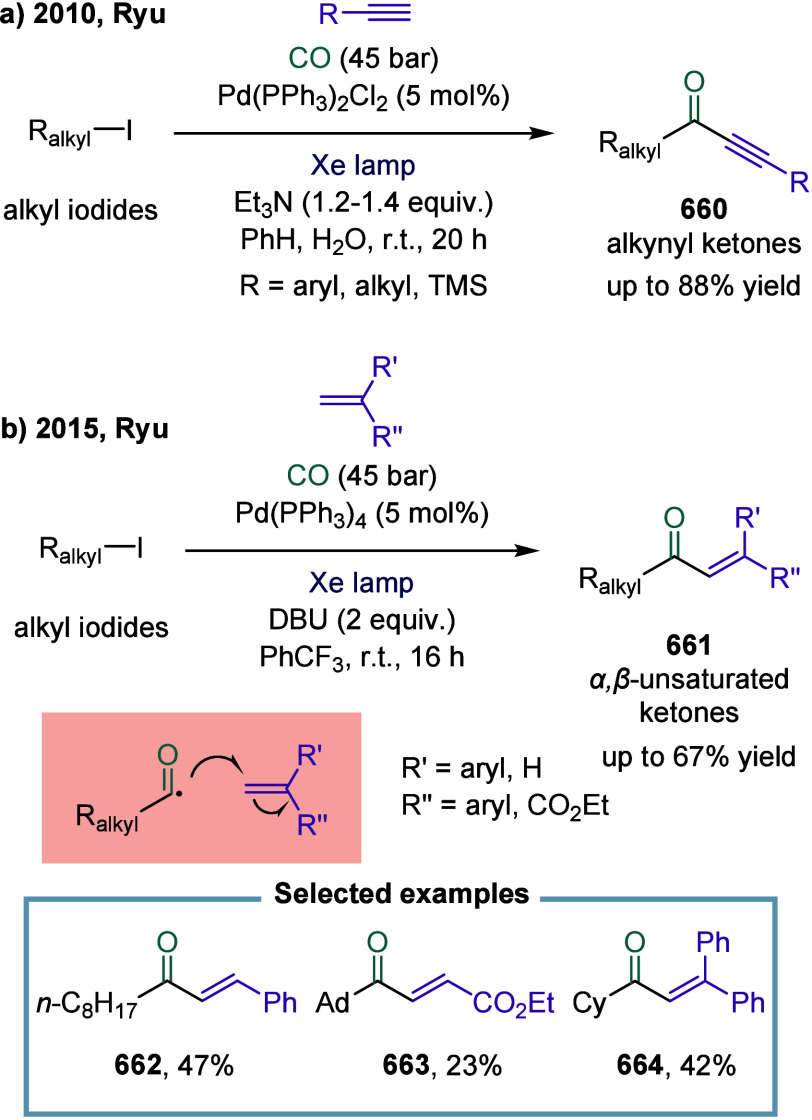
Palladium-Catalyzed Photo-Induced Carbonylation of Alkyl Halides: Synthesis of Alkynyl Ketones and α,β-Unsaturated Ketones

Mühlfenzl and co-workers developed a mild aminocarbonylation method of aryl iodides with amines, employing visible-light irradiation, palladium catalysis, and stoichiometric CO (Scheme 84).^388^ The reaction proceeded efficiently at ambient temperature and low CO pressure, thereby circumventing the harsh conditions typically required in classical carbonylation protocols. Notably, the use of the CO surrogate COgen (9-methyl-fluorene-9-carbonyl chloride) facilitated facile switching between labeled and unlabeled CO, enabling isotope incorporation while minimizing radioactive waste. The corresponding product **667** was obtained in 52% radiochemical yield (RCY) (34 MBq, SA: 0.09 TBq/mmol), consistent with yields observed in the unlabeled aminocarbonylation reactions.

**Scheme 84:**
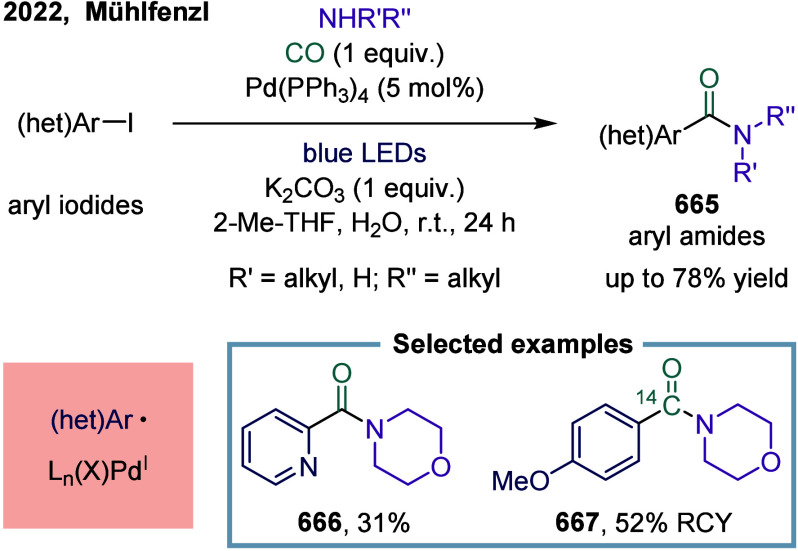
Palladium-Catalyzed Visible-Light Enabled Aminocarbonylation of Aryl Iodides

Historically, the carbonylation of unactivated alkyl halides has required harsh conditions, such as elevated temperatures, irradiation, or high CO pressures, as well as highly reactive iodide substrates. In 2016, Alexanian and co-workers reported a method that overcame these limitations by combining a palladium­(II) catalyst with a strongly electron-donating NHC ligand IMes, enabling efficient alkoxycarbonylation of a wide range of unactivated secondary alkyl bromides at 2 atm CO and moderate temperatures. (Scheme 85).^389^ The palladium catalyst initiated the reaction through irreversible abstraction of a bromine atom from alkyl bromide, generating a carbon-centered radical and a palladium^I^ species **669**. Subsequent steps proceeded via two plausible pathways: (i) recombination of the radical with the palladium center to form an alkylpalladium^II^ intermediate **670**, followed by CO migratory insertion, or (ii) direct radical addition to a coordinated CO ligand to form intermediate **670’**. Both pathways converged at the formation of a common acylpalladium­(II) intermediate **671**, which then underwent nucleophilic substitution by alcohols to deliver the ester product **668**. Despite the authors’ thorough investigations, the precise sequence of events, particularly whether CO insertion preceded or followed radical recombination with palladium, remained unresolved. Further studies are required to definitively elucidate these mechanistic pathways. However, primary alkyl bromides were unreactive under these conditions despite their higher rate of S_N_2 oxidative addition, indicating that radical activation was inherently more selective toward secondary substrates.

**Scheme 85:**
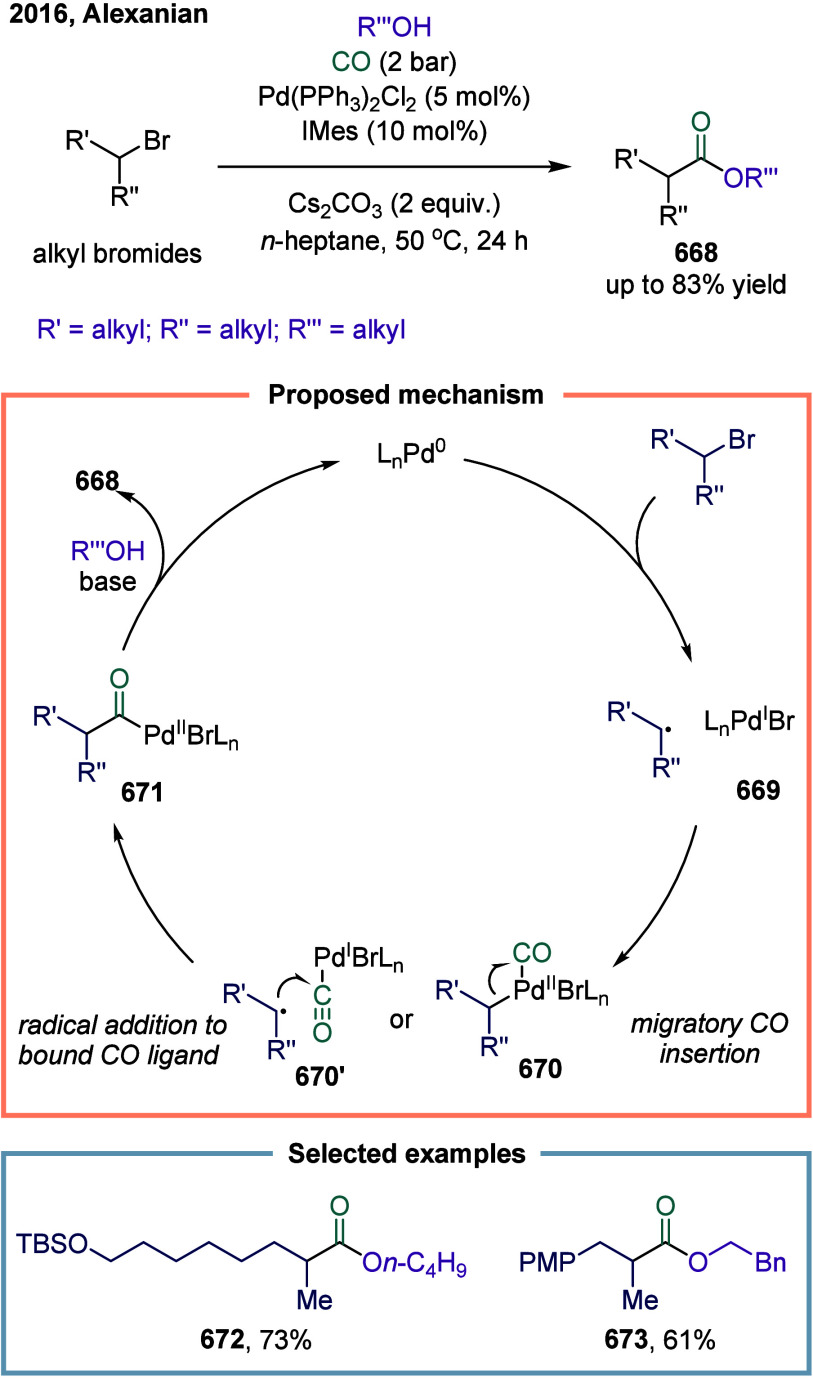
Palladium-Catalyzed Alkoxycarbonylation of Unactivated Secondary Alkyl Bromides at Low Pressure

Despite numerous significant advances in palladium-catalyzed carbonylation of organic halides, a fundamental challenge remains in balancing the oxidative addition and reductive elimination steps, as conditions or catalyst features that favor one step often inhibit the other. In 2020, Arndtsen and co-workers developed a dual light-driven palladium catalyst system that exploits visible-light excitation of both Pd^0^ and Pd^II^ intermediates to simultaneously and efficiently drive both oxidative addition and reductive elimination steps under mild, ambient conditions (Scheme 86a).^390^ This strategy overcame the conventional trade-off in catalyst design by eliminating the need to balance opposing steps through steric or electronic tuning. Light-driven excitation promoted radical pathways, wherein the excited state of Pd^0^ underwent SET to organic halides, generating a Pd^I^ species **675** and a carbon-centered radical, thereby facilitating low-barrier oxidative addition. Acyl-palladium complexes excited by light underwent reductive elimination via acyl radical formation, promoting acid chloride formation at room temperature. This approach exhibited a broad substrate scope, including functionalized aryl iodides/bromides and alkyl iodides/bromides. It was compatible with diverse nucleophiles, including anilines, alcohols, and thiols, affording the corresponding amides **678** and **679**, esters **680**, and thioesters **681**. The strategy achieved acid chloride formation (**676** and **677**) and enabled further transformations under mild conditions, often at ambient or subambient temperatures.

**Scheme 86:**
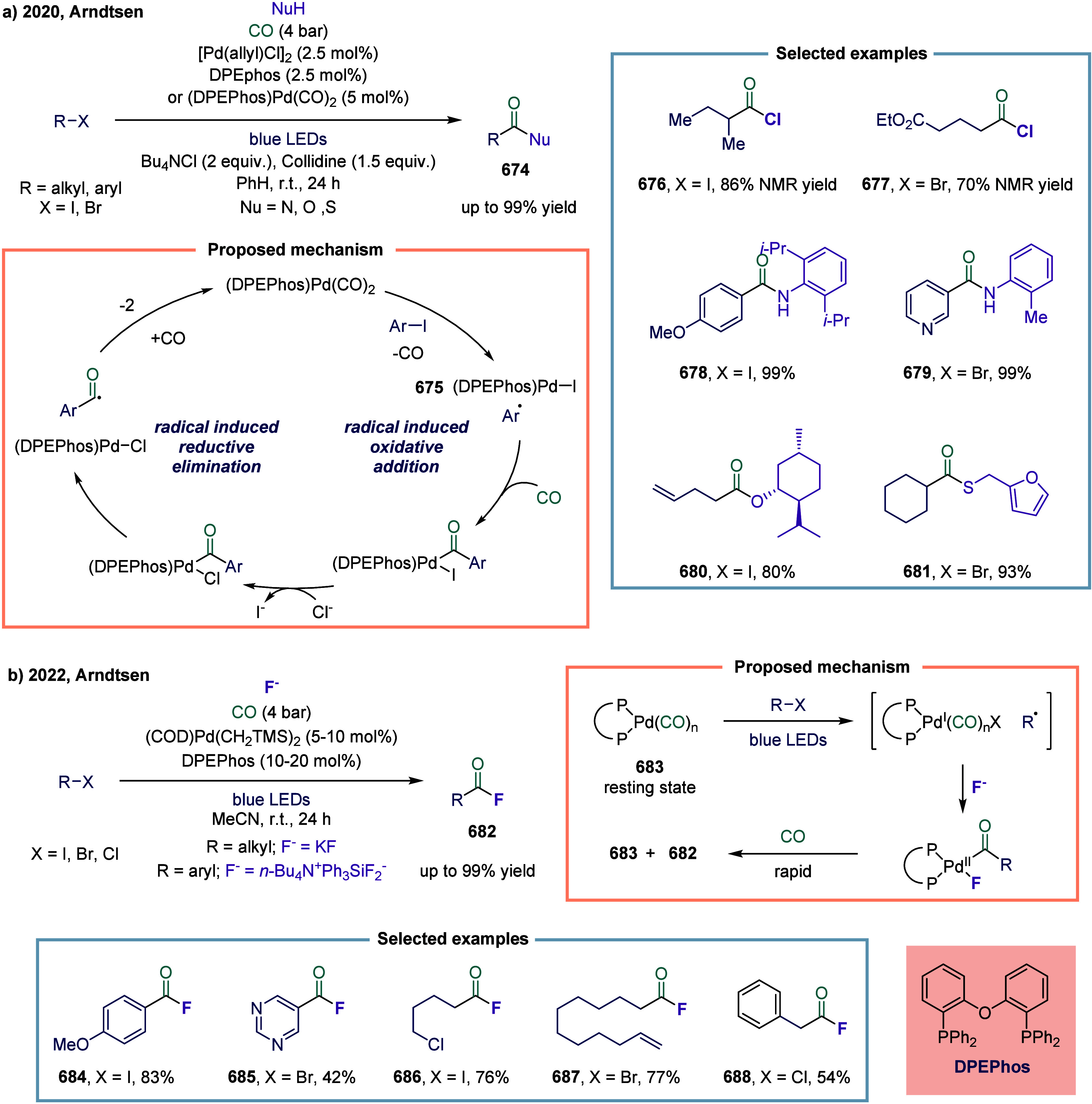
Light-Driven Palladium-Catalyzed Approach to Access Acyl Chloride and Acyl Fluorides

Compared with the well-studied formation of amides, esters, and ketones, the selective synthesis of acyl fluorides is more challenging and of greater synthetic value. In 2023, a versatile visible-light-promoted palladium-catalyzed general carbonylation platform to access acyl fluorides **682** was achieved by Arndtsen and co-workers (Scheme 86b).^391^ Acyl fluorides were selected as the focus of this study for two primary reasons: they are more stable to photoreduction than acid chlorides or alkyl halides, due to their higher reduction potentials;^392,393^ and they offer unique reactivity profiles, combining manageable stability, excellent electrophilicity, and broad nucleophile compatibility.^394,395^ The authors proposed that coordination of the sterically encumbered DPE-Phos ligand to palladium generated a catalyst **683** that integrated photoactivity, enabling visible-light-driven oxidative addition, along with a propensity for thermally promoted reductive elimination to form the acyl fluorides **682**. Aryl, heteroaryl, and alkyl halides, including iodides and bromides, were efficiently converted to the corresponding acyl fluorides (**684**-**687**). Notably, the scope of the reaction extended to activated alkyl chlorides, affording benzoyl fluoride **688** in 54% yield.

#### 3.3.3 Unsaturated Bonds

In 1995, Miyaura reported a palladium-catalyzed, three-component carbonylative cross-coupling reaction between iodoalkenes, carbon monoxide, and aryl- or alkyl-9-BBN reagents for the synthesis of unsymmetrical ketones **689** in up to 79% yield (Scheme 87a).^396^ A particularly notable feature of this transformation was that the oxidative addition of iodoalkenes to the palladium^0^ complex proceeded via a radical pathway, enabling intramolecular cyclization to form five-membered rings prior to coupling with carbon monoxide and the boron reagents. In 2011, a light-induced, palladium-catalyzed multicomponent carbonylative coupling reaction was developed, enabling the synthesis of functionalized esters **690** and lactones **691** in up to 84% and 77% yields (Scheme 87b).^397^ Under photoirradiation conditions with a xenon lamp and in the presence of Pd­(PPh_3_)_2_Cl_2_ as the catalyst, three- and four-component carbonylation reactions were successfully achieved using iodoalkanes, alkenes, carbon monoxide, and alcohols. In this reaction, various iodoalkanes bearing α-electron-withdrawing groups, such as ester, cyano, perfluoroalkyl, and sulfonyl substituents, smoothly delivered the target products in good to excellent yields.

**Scheme 87:**
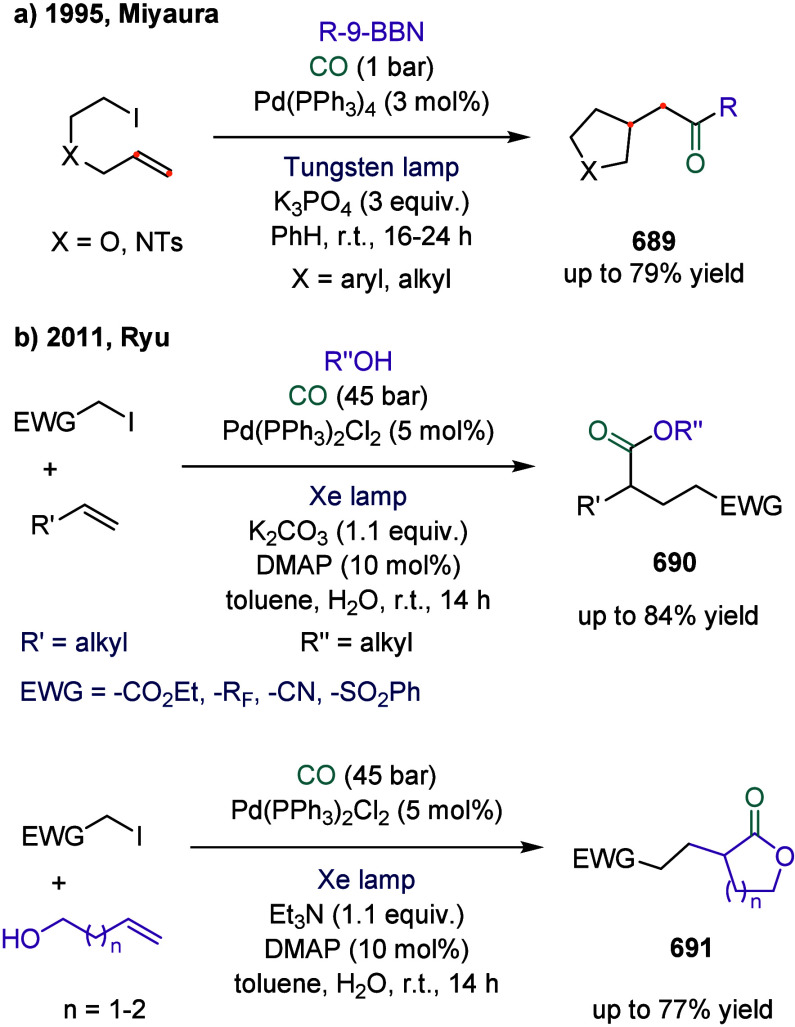
Palladium-Catalyzed Light-Induced Carbonylation of Alkenes Leading to Ketones, Esters, and Lactones

Palladium-catalyzed multicomponent carbonylative transformations of unactivated alkenes and carbon monoxide provide efficient access to a variety of functionalized esters and amides. In 2021, Wu and co-workers developed a palladium-catalyzed four-component carbonylation of unactivated alkenes and perfluoroalkyl halides, affording β-perfluoroalkyl esters **692** in high yields and with excellent chemoselectivity (up to 90% yield; Scheme 88a).^398^ Mechanistically, the catalytic cycle was initiated by the generation of the active Pd^0^Ln species from the Pd­(OAc)_2_ precatalyst under the reaction conditions. The Pd^0^Ln complex subsequently promoted SET reduction of the perfluoroalkyl halide, affording a perfluoroalkyl radical and a Pd^I^LnX species **693**. The perfluoroalkyl radical underwent addition to the alkene, generating a new secondary carbon radical. This radical then recombined with the Pd^I^LnX species **693** to form the key organopalladium intermediate **694**, which underwent migratory insertion of carbon monoxide to furnish intermediate **695**. Finally, intermediate **695** underwent tandem nucleophilic substitution and reductive elimination in the presence of base to deliver the desired product **692**. Additionally, the β-perfluoroalkyl iodide byproduct, formed via reductive elimination, was reactivated through further SET with the Pd^0^Ln species, ultimately leading to its conversion into the target compound **692**. In this reaction, a variety of fluoroalkyl halides were smoothly converted into the corresponding β-fluoroalkyl esters in good yields, exemplified by compounds **696–698**. Notably, even less reactive alkenes, including internal alkenes and ethylene, proved to be suitable substrates, affording the desired products **699** and **700**. Furthermore, a broad range of alkyl halides, including alkyl bromides, iodides, and chlorides, were successfully transformed into the corresponding β-perfluoroalkyl-substituted alkyl esters, such as compound **701**.

**Scheme 88:**
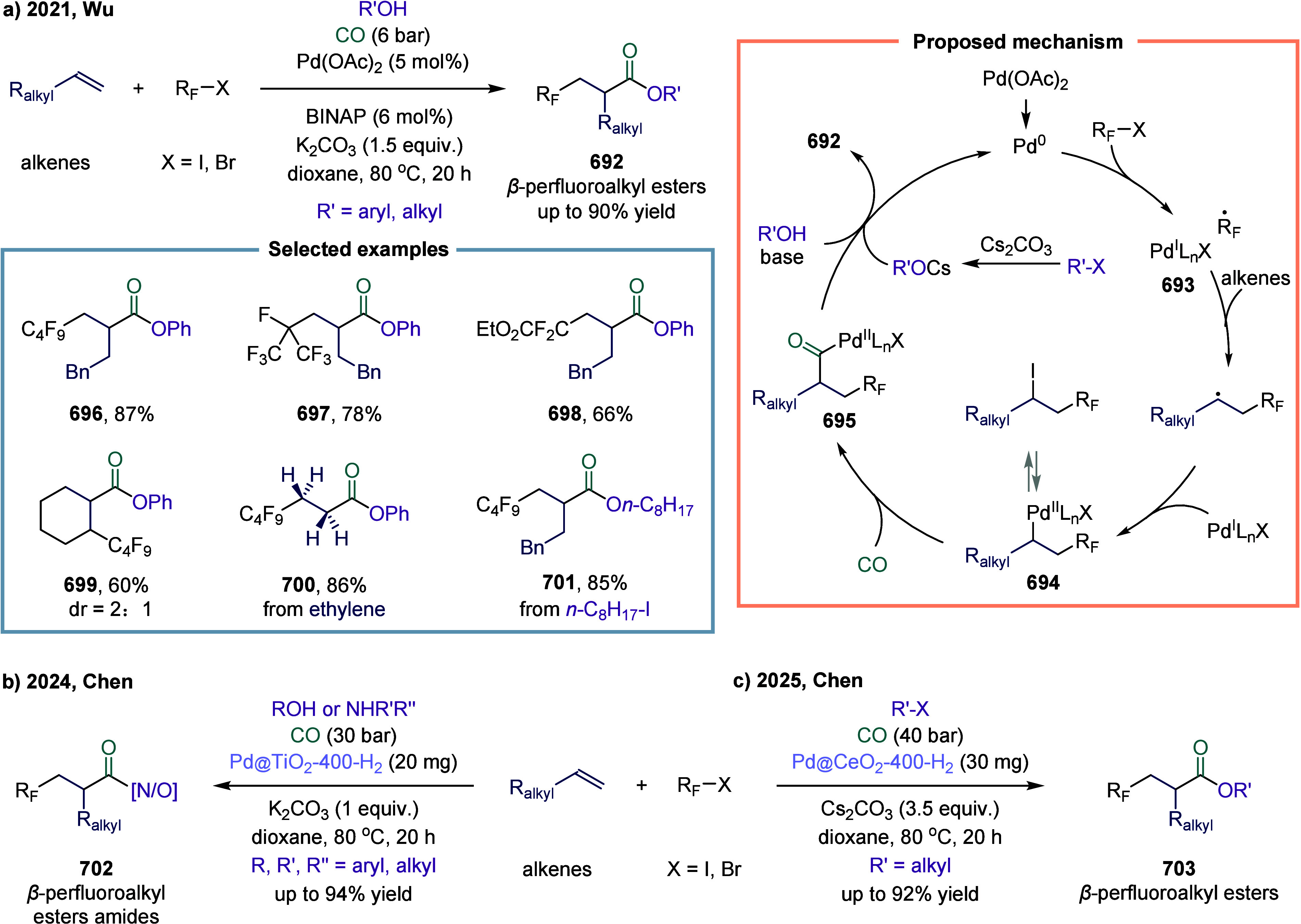
Palladium-Catalyzed Perfluoroalkylative Carbonylation of Unactivated Alkenes to Access *β*-Perfluoroalkyl Esters and Amides

In 2024, Chen and co-workers developed a ligand-free, heterogeneous Pd@TiO_2_-catalyzed perfluoroalkylative carbonylation of terminal alkenes (Scheme 88b).^399^ From a green chemistry perspective, issues such as metal contamination and challenging purification steps have hindered the practical application of carbonylation methodologies. This novel ligand-free, heterogeneous Pd@TiO_2_ catalyst enabled the selective carbonylative difunctionalization of terminal alkenes under mild and sustainable conditions. Notably, the catalyst exhibited outstanding recyclability: after five consecutive runs, no significant loss of activity or selectivity was observed. In 2025, the same group investigated a series of CeO_2_-supported palladium catalysts and applied them to the perfluoroalkylative carbonylation of alkenes with alkyl halides (Scheme 88c).^400^ Among these, the Pd@CeO_2_-400-H_2_ catalyst exhibited high activity and selectivity in the four-component synthesis of β-perfluoroalkyl esters **703**, demonstrating excellent recyclability and broad functional group tolerance.

In 2022, the Wu group reported a versatile palladium-catalyzed strategy for the perfluoroalkylative carbonylation of 2-allylaryl trifluoromethanesulfonates, enabling access to β-perfluoroalkyl amides (Scheme 89a).^401^ The reaction featured base-controlled selectivity, enabling the formation of three distinct classes of products: monoamide, bis-amide, and cyclized amide derivatives. Specifically, Cs_2_CO_3_ promoted cyclization to furnish benzazepine-1,3-dione derivatives **704**, NaOH afforded monocarbonylated amides **705**, and K_3_PO_4_ favored the formation of bis-amide products **706**. With respect to substrate scope, iodide-substituted alkenes provided the corresponding product **707** in 28% yield under Cs_2_CO_3_-mediated conditions. However, only anilines were found to be suitable nucleophiles, as aliphatic amines failed to deliver the desired products, thereby limiting the scope of applicable nucleophiles. Additionally, no target compounds were detected when 2-allyl trifluoromethanesulfonates (**710** and **711**) were employed as substrates. In 2024, Wu and co-workers reported a palladium-catalyzed difluoroalkylative carbonylation of unactivated alkenes for the synthesis of γ-lactams **712** (Scheme 89b).^402^ γ-Lactams represented important core structures found in numerous natural products and biologically active molecules. Under relatively mild conditions, the desired γ-lactams were obtained in moderate to good yields.

**Scheme 89:**
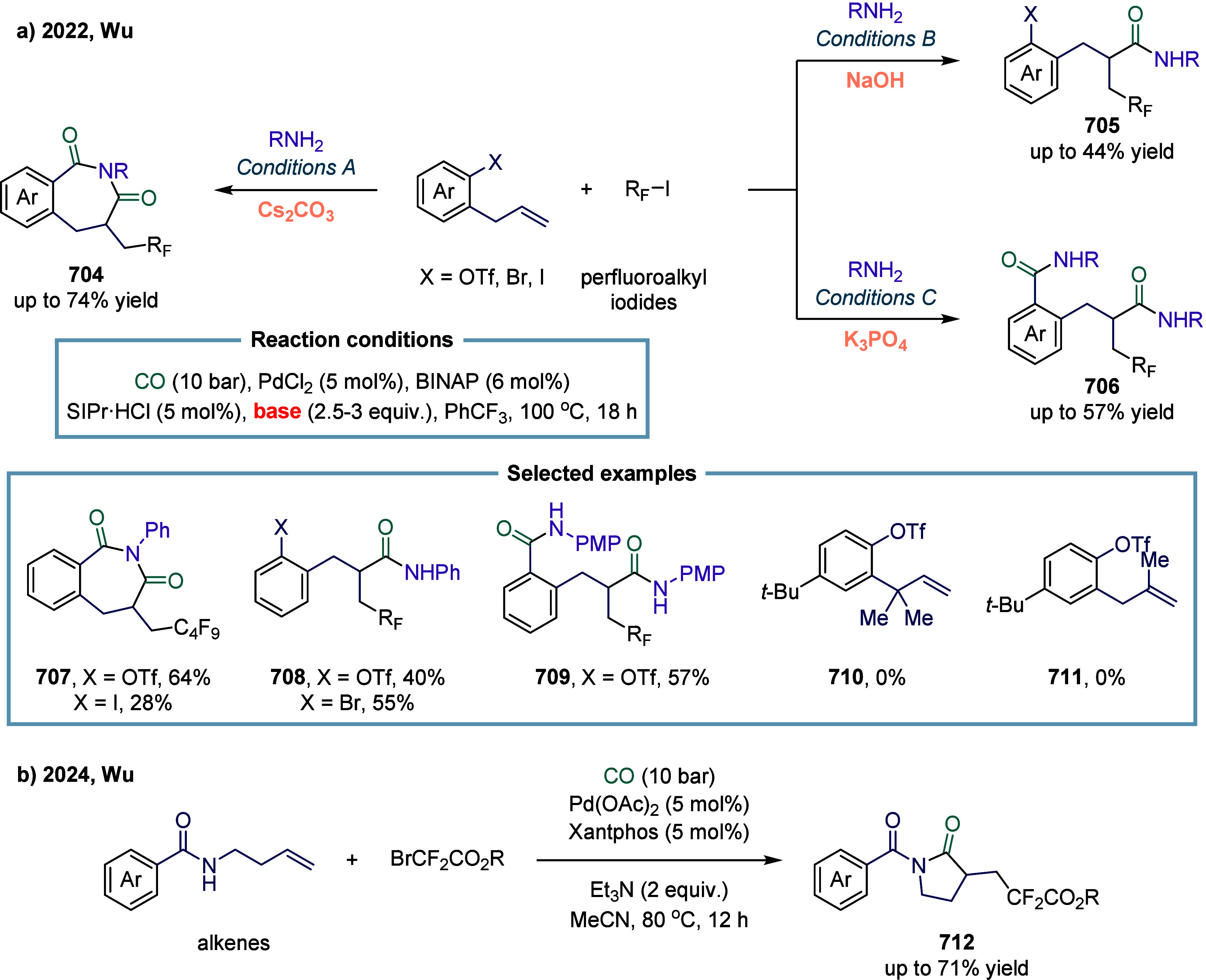
Palladium-Catalyzed Perfluoroalkylative Carbonylation of 2-Allylaryl Trifluoromethanesulfonates or Difluoroacetates

In addition to unactivated alkenes, palladium-catalyzed SET-mediated multicomponent carbonylation was also successfully applied to activated alkenes, particularly aromatic alkenes. In 2022, Wu and co-workers reported a simple and practical palladium-catalyzed strategy for the difluoroalkylative carbonylation of aromatic alkenes, enabling the efficient synthesis of difluoroglutaric acid ester derivatives **713** (Scheme 90a).^403^ In this transformation, ethyl bromodifluoroacetate served dually as the difluoroalkyl source and nucleophile in a single operational step. The method demonstrated excellent regioselectivity and broad functional group tolerance across a diverse range of aromatic olefins, including representative examples such as compounds **714**, **715**, and **716**. Furthermore, when an amine nucleophile was introduced into the reaction system, the protocol selectively furnished β-difluoromethylene-substituted amide derivatives **717** in up to 90% yield, highlighting its versatility for the construction of valuable fluorinated building blocks (Scheme 90b).^404^

**Scheme 90:**
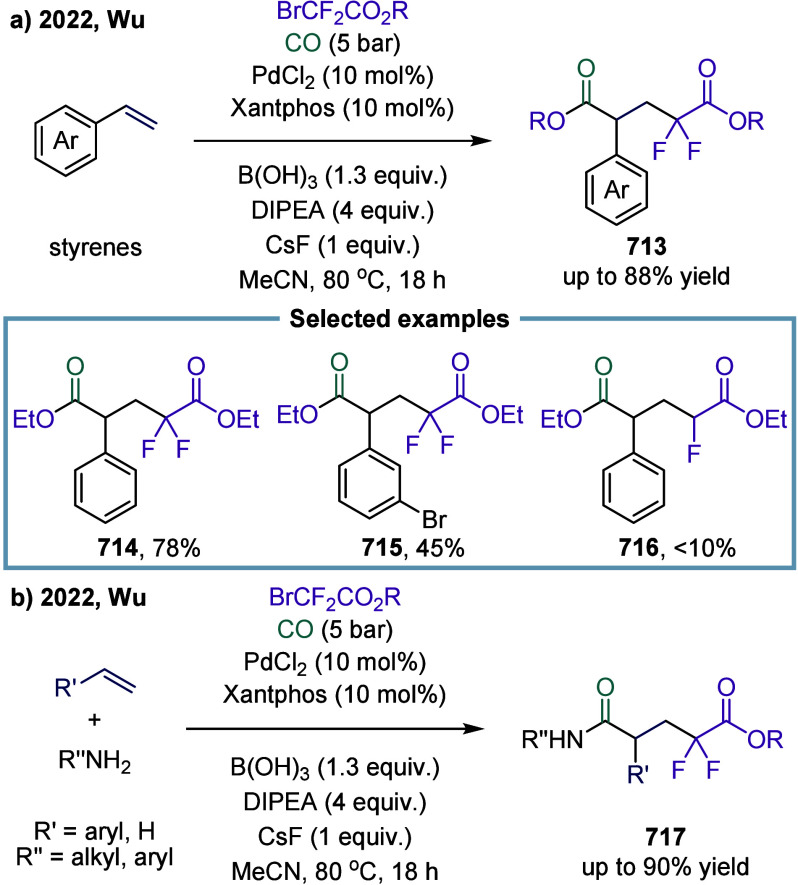
Palladium-Catalyzed Difluoroalkylative Carbonylation of Styrene or Ethylene

The difunctionalization of alkynes has greatly expanded the repertoire of synthetic methodologies in organic chemistry, owing to the inherent versatility of this transformation and the widespread availability of alkyne substrates.^405,406^ Among these strategies, transition-metal-catalyzed carbonylative functionalization has emerged as a highly effective approach to access structurally diverse and valuable molecules. In 2016, Liang and co-workers reported a palladium-catalyzed four-component strategy to accomplish difluoroalkylative carbonylation of alkynes with excellent yields (up to 88% yield; Scheme 91a).^407^ This transformation enabled the incorporation of the valuable CF_2_ moiety into organic scaffolds, concurrently forming two new C–C bonds, including a C–CF_2_ linkage, and either a C–O (ester) or C–N (amide) bond in a single step under mild conditions. Mechanistically, the difluoroalkyl radical added to an alkyne to generate vinyl radical intermediate. Subsequent recombination with Pd^I^ afforded the key Pd^II^ complex **719**. Carbon monoxide insertion into this Pd^II^ complex generated intermediate **720**, which underwent reductive elimination in the presence of a nucleophile and base to furnish the carbonylated alkenes **718** and regenerate the Pd^0^ catalyst. The synthetic utility of this reaction system was demonstrated by its broad applicability to a wide range of alkynes and nucleophiles, including compounds **721**, **722**, and **723**. Both aryl and alkyl amines were well tolerated in this transformation, affording products with excellent E/Z selectivity.

**Scheme 91:**
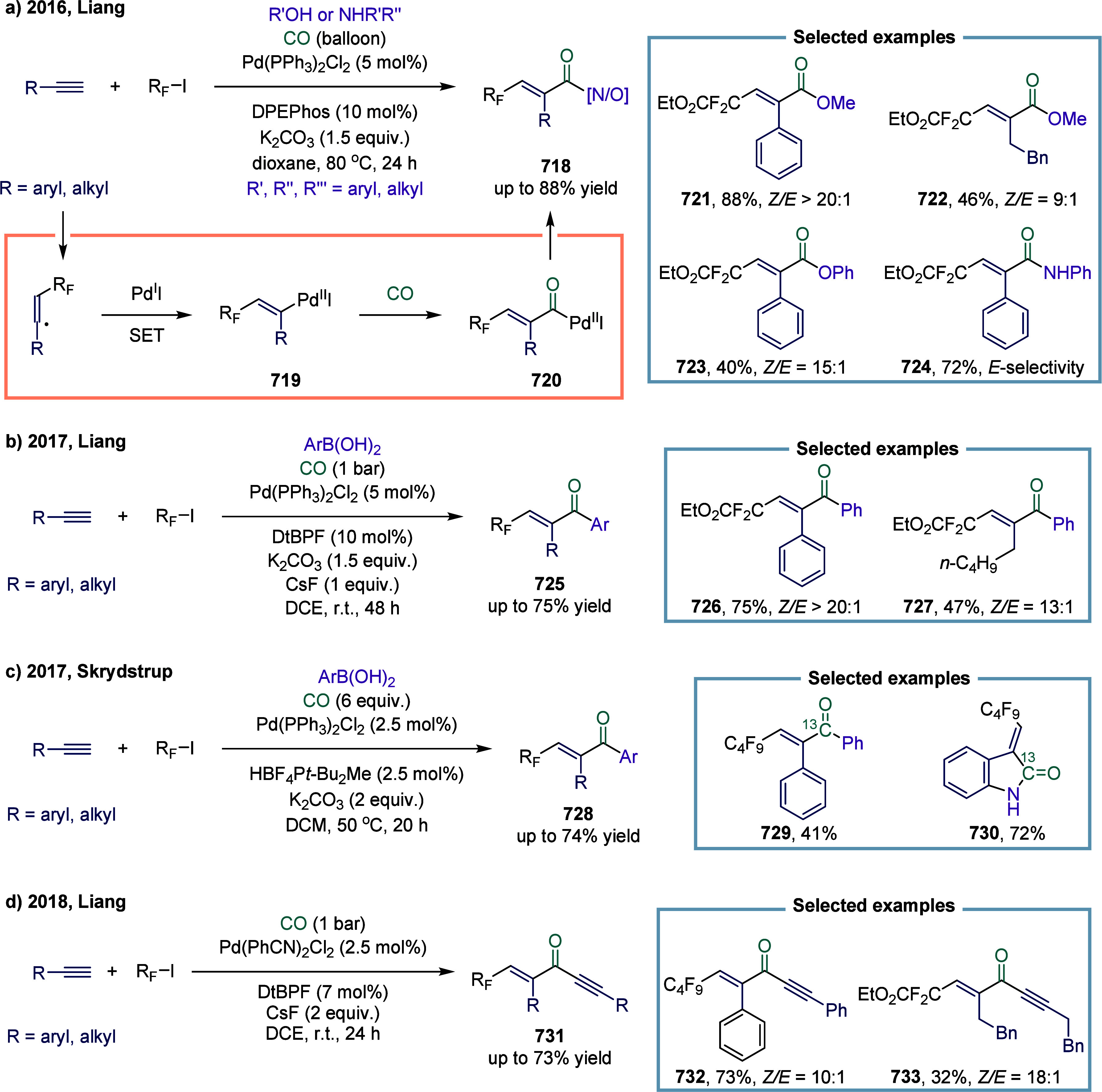
Palladium-Catalyzed Regioselective Fluoroalkylative Carbonylation of Alkynes

In 2017, the same group developed a regioselective four-component carbonylation strategy for the synthesis of difluoroalkyl- and perfluoroalkyl-substituted enones, enabled by a palladium catalyst (Scheme 91b).^408^ The reaction proceeds smoothly under mild conditions at room temperature and 1 atm of carbon monoxide, affording good yields and excellent *E*-selectivities (up to 75% yield, 20:1 *E/Z*). A variety of alkynes, including both aryl **726** and alkyl substrate **727**, were well tolerated in this reaction. Later in 2017, Skrydstrup and co-workers reported a palladium-catalyzed four-component carbonylative coupling reaction to access perfluoroalkyl-substituted enones (Scheme 91c).^409^ This method enabled the efficient synthesis of a broad range of highly functionalized enones **728** in a single step with good yields. When 2-aminophenylalkynes were employed as substrates, intramolecular aminocarbonylation predominated, furnishing the indolin-2-one scaffold **730**. Notably, the adaptation of a two-chamber technology permitted the incorporation of ^13^C-isotopic labeling, such as **729** and **730**, thereby expanding the synthetic utility of this approach. Subsequently, the Liang’s laboratory found that in the absence of external nucleophiles, such as alcohols, phenols, amines, and arylboronic acids, conjugated 1,4-enyn-3-ones **731** were obtained in up to 73% yield (Scheme 91d).^410^ Notably, alkyl-substituted alkynes also successfully underwent this reaction, affording the target product **733** in 32% yield with 18:1 *E/Z*.

In 2022, Wu and co-workers utilized benzene-1,3,5-triyl triformate (TFBen) as a CO surrogate to develop a palladium-catalyzed cascade carbonylation for the synthesis of perfluoroalkyl- and carbonyl-functionalized 3,4-dihydroquinolin-2­(*1H*)-one derivatives **734** (Scheme 92a).^411^ 3,4-Dihydroquinolin-2­(*1H*)-ones represented an important class of scaffolds widely found in natural products, pharmaceuticals, and biologically active molecules.^412,413^ This strategy enabled the simultaneous incorporation of perfluoroalkyl and carbonyl moieties into the 3,4-dihydroquinolin-2­(*1H*)-one framework, affording a diverse array of derivatives **734** in moderate to high yields and with excellent E/Z selectivity. The proposed mechanism involved a two-step sequential addition of radical species to unsaturated bonds, specifically, C=C and C≡C bonds, to generate alkyl radical intermediate and alkenyl radical intermediate, respectively. Additionally, when *N*-unsubstituted substrates **737**, *O*-linked 1,7-enynes **738**, nonsubstituted **739**, or phenyl-substituted **740** were subjected to the reaction conditions, no desired products were obtained. In 2024, Liang and co-workers reported a palladium-catalyzed four-component radical cascade carbonylation to access 2,3-disubstituted benzofuran derivatives (Scheme 92b).^414^ A variety of valuable 2,3-disubstituted benzofuran derivatives **741** were obtained in up to 92% yield with excellent functional group compatibility. However, ethyl bromodifluoroacetate and a series of iodofluoroalkyl reagents were not compatible with this transformation, such as compound **743**, and the corresponding indole products could not be successfully obtained by this approach.

**Scheme 92:**
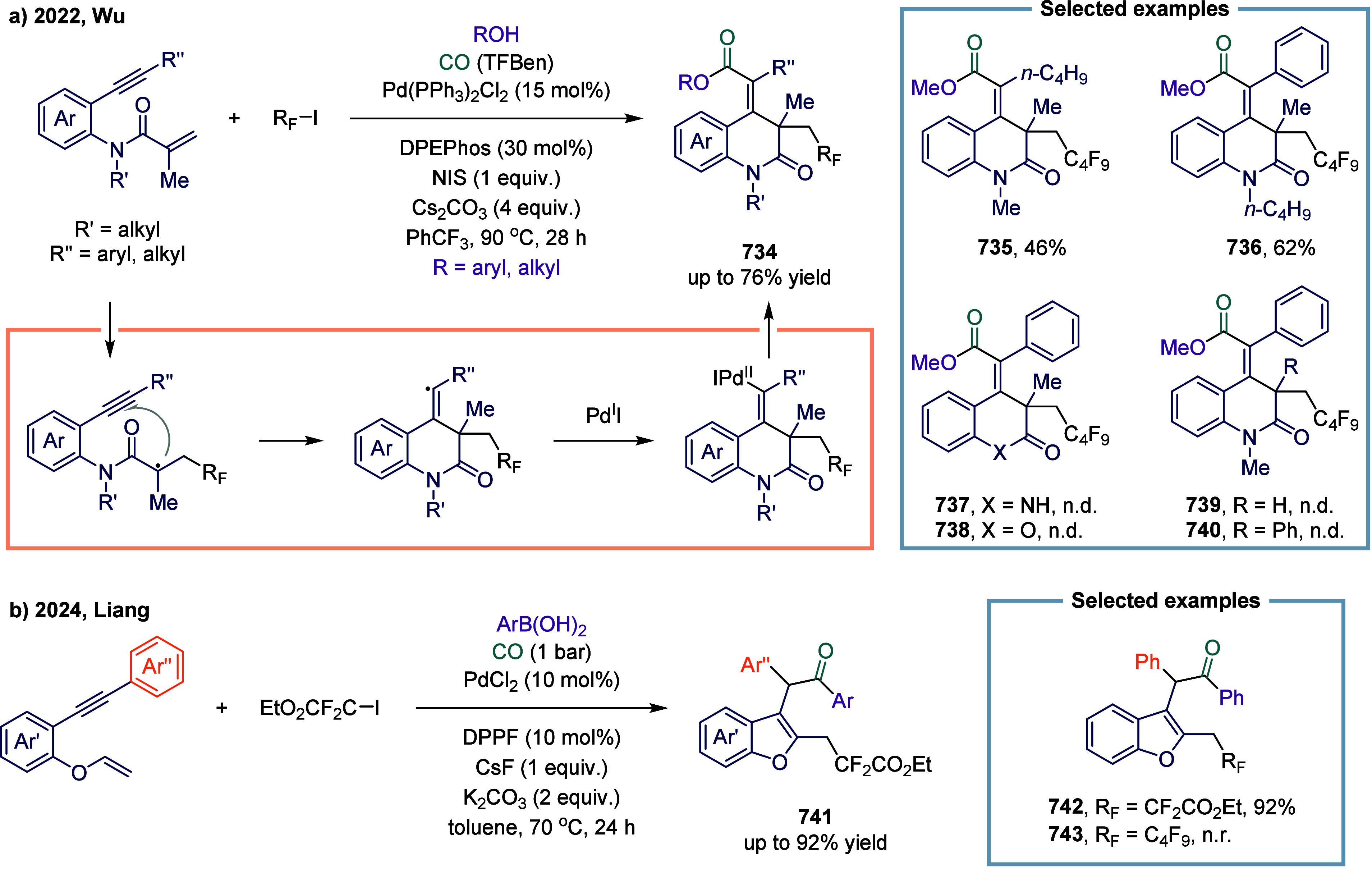
Palladium-Catalyzed Cascade Carbonylative Synthesis of Perfluoroalkyl and Carbonyl-Containing 3,4-Dihydroquinolin-2­(1*H*)-one Derivative and 2,3-Disubstituted Benzofuran Derivatives

In 2024, Beller and co-workers achieved a palladium-catalyzed four-component carbonylation of acetylene for the synthesis of β-perfluoroalkyl acrylamides **744** (Scheme 93).^415^ This method enabled the simultaneous installation of both an acrylamide moiety, a known covalent inhibitor motif, and a perfluoroalkyl group, a pharmacophore associated with favorable ADME properties. The protocol demonstrated broad functional group tolerance, including compatibility with complex bioactive molecules such as Norquetiapine and a Flunarizine fragment, delivering the corresponding products **745** and **746** in 61% and 55% yields, respectively. The combination of acetylene and carbon monoxide as gaseous C2 and C1 units, respectively, with perfluoroalkyl halides and amines in a one-pot palladium-catalyzed process represented a significant advance in synthetic methodology.

**Scheme 93:**
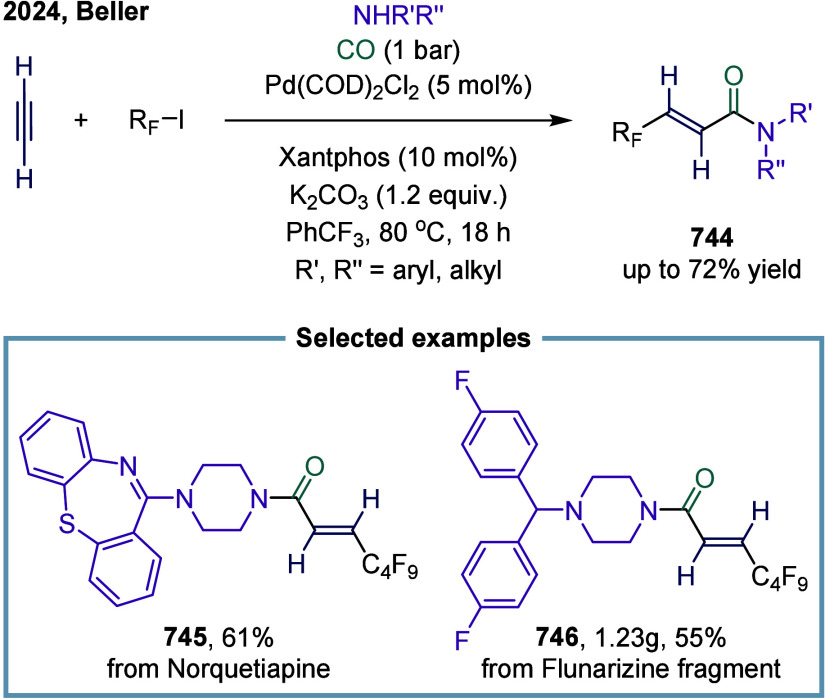
Palladium-Catalyzed Four-Component Carbonylation Reactions of Acetylene

### 3.4 Silver-Catalyzed System

Silver, with an electronic configuration of [Kr] 4d^10^5s^1^, forms a broad range of silver­(I) salts that act as effective σ- and π-type Lewis’s acids.^416^ Silver displays a distinctive alkynophilicity arising from its d^10^ electronic configuration, which enables the efficient activation and transformation of alkynes.^417,418^ The application of silver as a sole catalyst in carbonylative reactions remains scarce, as this metal generally exhibits limited catalytic activity in such transformations. In 2024, the Wu group studied carbamoylation and carbonylative cyclization of alkenes with oxamic acids catalyzed by a silver catalyst (Scheme 94).^419^ Among the catalysts evaluated, including copper, iron, and various silver salts, AgNO_3_ exhibited the highest activity. In the presence of silver salt and ammonium persulfate, oxamic acid underwent decarboxylation to generate a carbamoyl radical, which subsequently added to the unsaturated bond, forming a new carbon-centered radical. This intermediate then underwent carbonylation followed by intramolecular cyclization to afford the target product **747** in up to 61% yield. The reaction displayed broad applicability toward various 4-phenyl-1-butene derivatives. However, alkenes with either longer or shorter carbon chains, as well as α-oxa-phenylbutene **750**, failed to yield the desired products under the standard conditions. Additionally, phenyl-substituted oxamic acid **751** was found to be unreactive under these conditions.

**Scheme 94:**
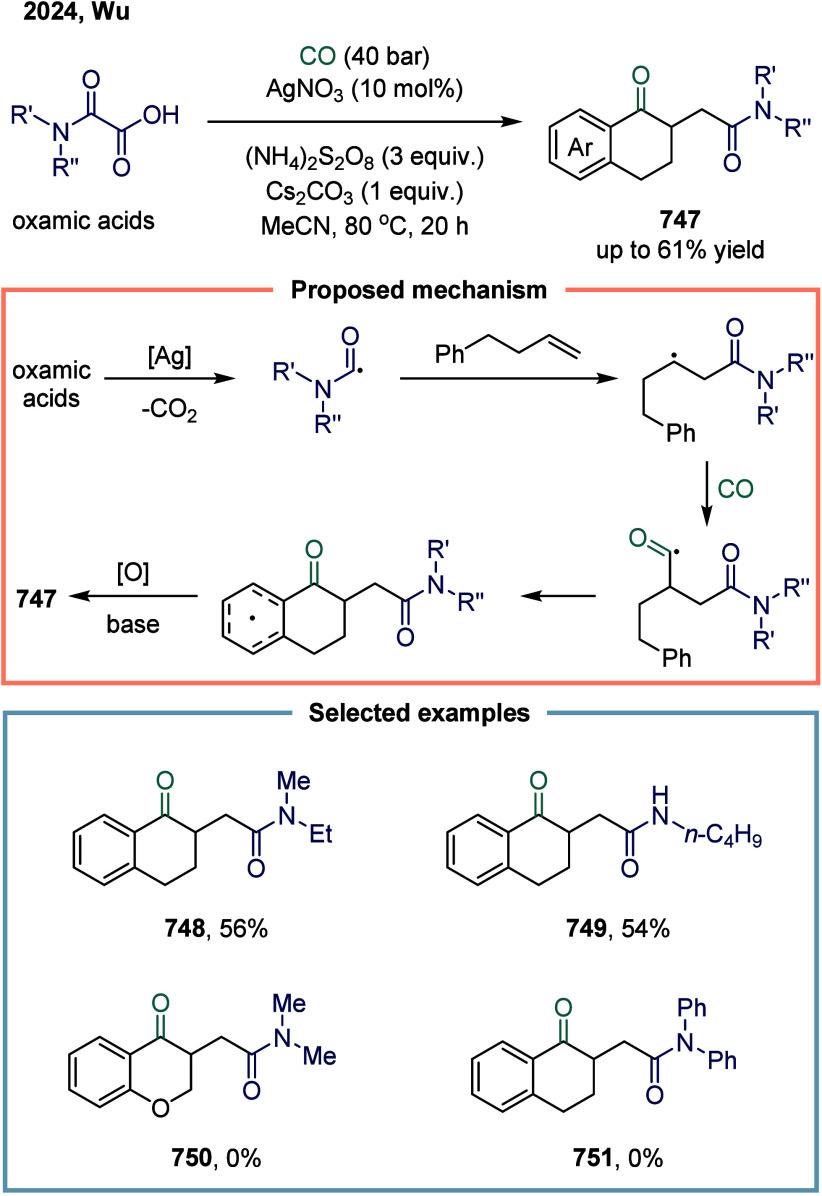
Silver-Catalyzed Carbamoylation and Carbonylative Cyclization of Alkenes with Oxamic Acids

## 4 Third-Row Transition Metals

Iridium (Ir) and tungsten (W), both belonging to the 5d transition metal series, differing from traditional transition metal-catalyzed carbonylation reactions, have recently emerged as powerful photocatalysts in carbonylation chemistry, primarily functioning as photocatalysts in recent years due to their distinct involvement in single-electron transfer (SET) processes.^420–422^ Leveraging robust photophysical properties, complexes based on these metals effectively facilitate carbonylation reactions under mild conditions. Iridium-based photocatalysts, in particular, demonstrate exceptional photoredox capabilities, characterized by strong visible-light absorption, uniquely long-lived excited states, and superior oxidation–reduction potentials, enabling efficient activation of otherwise inert substrates via radical-mediated pathways. Similarly, tungsten photocatalysts, especially polyoxotungstate complexes, stand out because of their unique electronic structures, exceptional stability, and robust photoinduced electron transfer capabilities, surpassing traditional transition metal catalysts such as ruthenium or copper complexes. Consequently, the utilization of W and Ir significantly broadens the scope, versatility, and efficiency of carbonylation methodologies, facilitating the synthesis of structurally diverse carbonyl-containing compounds.

### 4.1 Decatungstate Anion-Catalyzed System

The decatungstate anion, exemplified by TBADT (tetrabutylammonium decatungstate), is a powerful photocatalyst and belongs to the large family of polyoxometalates (POMs).^423–425^ The decatungstate anion exhibits a broad absorption band centered at 324 nm (ε_324_ = 14100 M^–1^·cm^–1^), corresponding to a HOMO–LUMO transition with pronounced LMCT character, involving electron transfer from oxygen to tungsten centers.^426^ It has emerged as a versatile and highly efficient HAT catalyst under UV or near-UV irradiation. Upon excitation with light (typically 365–390 nm), TBADT generates a highly reactive excited state capable of abstracting hydrogen atoms from strong aliphatic C–H bonds, thereby producing carbon-centered radicals.^427^ These radicals can participate in a broad range of synthetically valuable transformations, including C–C, C–O, and C–N bond formation. Due to its robust oxidative potential, high chemoselectivity, and operational simplicity, TBADT has been widely applied in site-selective C–H functionalization of unactivated hydrocarbons and late-stage modification of complex molecules. Furthermore, the decatungstate anion synergistically controls hydrogen abstraction from the S_H_2 (bimolecular homolytic substitution) transition state through combined polar and steric effects, offering a promising strategy for achieving site-selective carbonylation of C­(sp^3^)-H bonds under photocatalytic conditions. Ryu and co-workers have made significant contributions to the development of decatungstate anion-catalyzed C­(sp^3^)-H functionalization, particularly pioneering radical-based strategies for C­(sp^3^)-H carbonylative transformations.

In 2010, Ryu and co-workers developed a TBADT-catalyzed C­(sp^3^)-H carbonylation of alkanes and coupling with CO and electrophilic alkenes for the synthesis of unsymmetrical ketones **752** (Scheme 95a).^428^ Mechanistically, photoexcitation of the decatungstate catalyst, followed by intersystem crossing, generated the reactive triplet excited state, which abstracted a hydrogen atom from the alkane substrate to afford an alkyl radical intermediate along with the singly reduced decatungstate species. The alkyl radical subsequently underwent reversible addition to carbon monoxide, forming the acyl radical. This acyl radical then underwent radical addition to electrophilic alkenes, generating a new carbon-centered radical. Finally, back hydrogen atom transfer from the reduced decatungstate regenerated catalyst and yielded the ketone product **752**. The reaction was successfully carried out using a xenon lamp as the light source, with 4 mol % TBADT catalyst under 80 bar CO. Both 1,2-disubstituted and 1,1-disubstituted olefins were suitable substrates, affording the desired products **753** and **754** in 77% and 58% yields, respectively. Furthermore, the authors demonstrated that the hydrogen atom is exclusively transferred from the reduced photocatalyst, thereby regenerating it, rather than from the reaction medium.

**Scheme 95:**
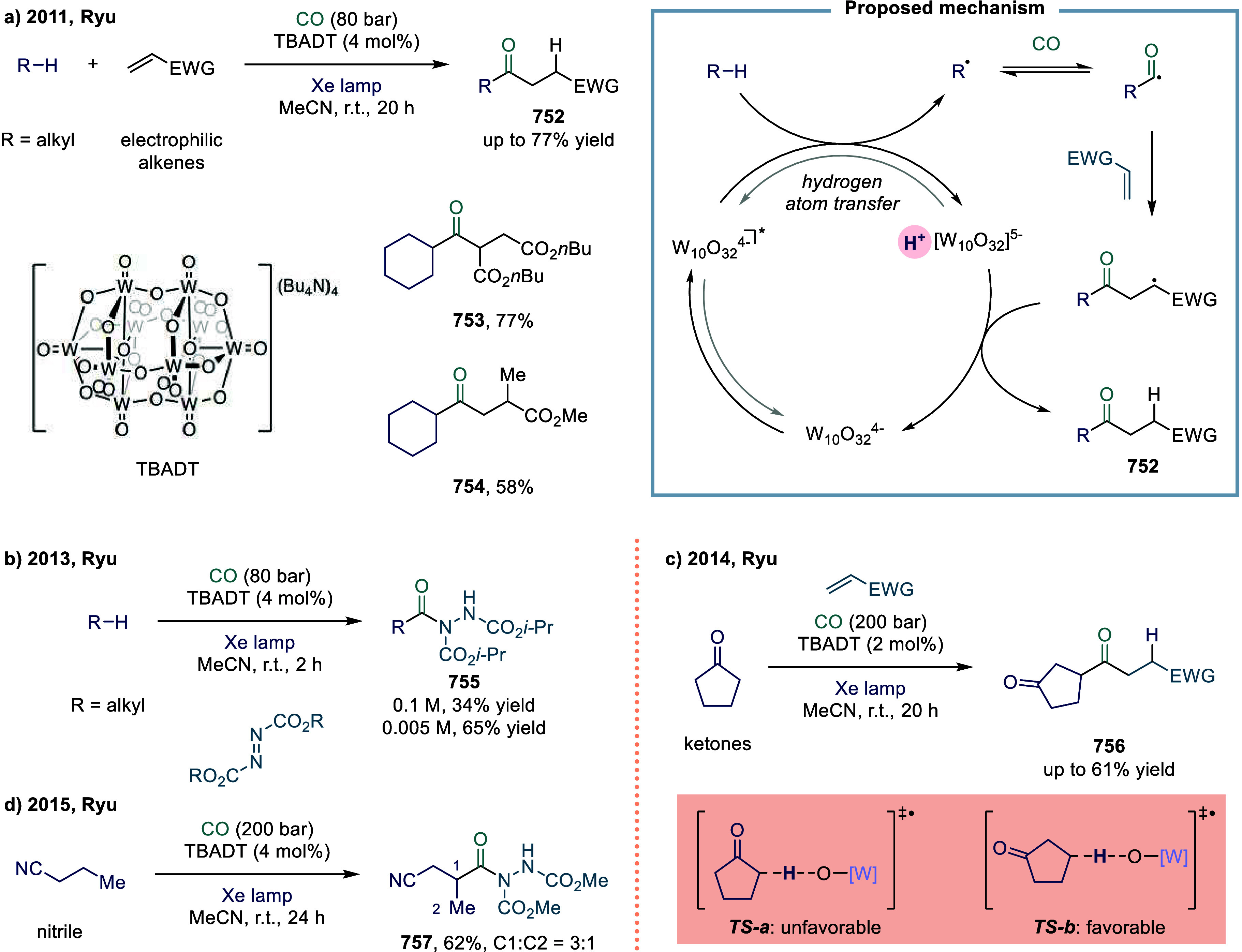
C­(sp^3^)-H Carbonylation Catalyzed by Decatungstate Anion Photocatalysis

In 2013, Ryu and co-workers developed a three-component carbonylation reaction involving cycloalkanes, CO, and diisopropyl azodicarboxylate (DIAD) under irradiation, which afforded acyl hydrazides **755** in up to 65% yield (Scheme 95b).^429^ At a DIAD concentration of 0.1 M under 80 atm of CO, product **755** was obtained in 34% yield, along with a comparable 28% yield of the alkylated byproduct, indicating that the addition of the cyclohexyl radical to DIAD proceeded rapidly. To favor radical carbonylation over direct addition of the cyclohexyl radical to DIAD, the effect of lowering the DIAD concentration was investigated. This strategy proved effective: dilution to 5 × 10^–3^ M afforded product **755** in 65% yield.

The site-selective transformation of C­(sp^3^)-H bonds into value-added chemicals continues to represent a significant challenge in synthetic organic chemistry.^430^ In recent years, considerable efforts have been devoted to achieving this objective through both transition metal-catalyzed strategies and radical-based methodologies.^431^ Ryu and co-workers disclosed a photoinduced direct regioselective β-carbonylation of cyclopentanones with electron-deficient alkenes in 2014 (Scheme 95c).^432^ when an acetonitrile solution of cyclopentanone, electrophilic alkenes, and TBADT (2 mol %) was irradiated for 20 h under 200 bar CO atmosphere, the corresponding β-carbonylated product **756** was obtained in up to 61% yield. In cyclopentanone, the α–C-H bond is intrinsically weaker than the β-C-H bond. Nevertheless, the authors proposed that β-selective C–H cleavage is favored by polar effects under conditions involving a highly polar S_H_2 transition state. The electronegativity of oxygen-centered radicals induces a polar transition state during hydrogen abstraction, stabilizing the partial positive charge developing on the carbon center. Consequently, transition state *TS-a*, corresponding to α–C-H cleavage forming the α-radical intermediate, generates an electron-deficient α-carbon exhibiting Umpolung character, which is inherently less stabilized. In contrast, β–C-H bond cleavage via transition state *TS-b* is more favorable, selectively leading to the formation of the β-radical intermediate. In 2015, the same group developed a photocatalyzed site-selective C­(sp^3^)-H carbonylation of aliphatic nitriles (Scheme 95d).^433^ The reaction gave a mixture of β-site product and γ-site product in a 3:1 ratio under high CO pressures. The observed site selectivity was attributed to the influence of radical polar effects within the hydrogen abstraction transition states.

Despite significant advances in C­(sp^3^)-H carbonylation in recent years, the development of methodologies for gaseous alkanes has progressed relatively slowly. This lag was primarily due to the presence of some of the strongest C­(sp^3^)-H bonds found in nature, which required harsh activation conditions. In 2023, Noël and colleagues reported an efficient method for the C­(sp^3^)-H carbonylation of alkanes, particularly gaseous alkanes, with CO under HAT photocatalysis in flow conditions (Scheme 96).^434^ The use of flow technology was critical for achieving high gas–liquid mass transfer rates and rapid reaction kinetics. Moreover, isotopic labeling was readily accomplished in this system, affording a ^13^C-labeled compound **759** in 79% yield. Importantly, gaseous alkanes such as butane, propane, and ethane proved to be suitable substrates, delivering the corresponding ketones **761**, **762**, and **763** in moderate yields.

**Scheme 96:**
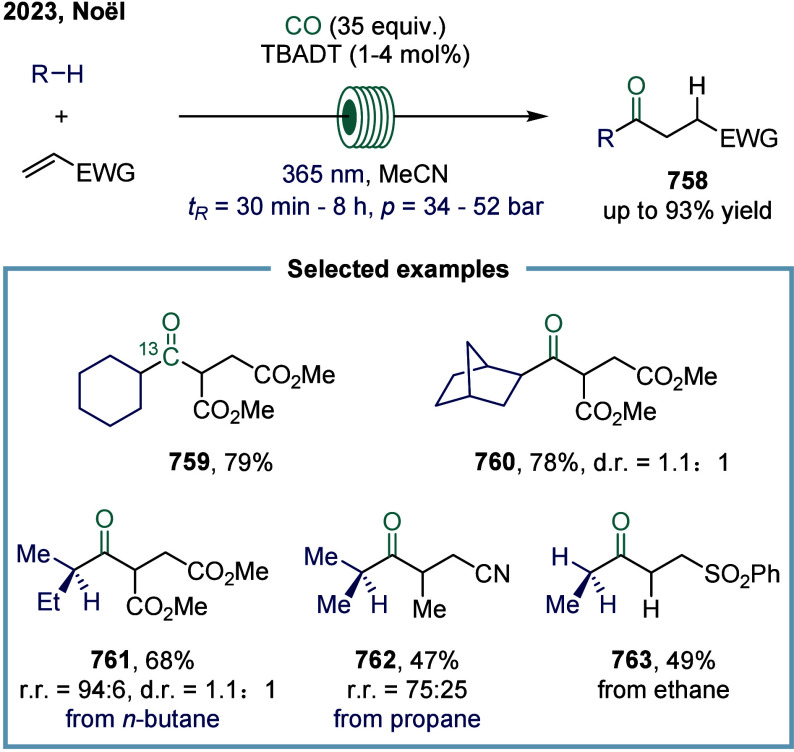
C­(sp^3^)-H Carbonylation of Light and Heavy Hydrocarbons Catalyzed by Decatungstate Anion Photocatalysis in Flow

In 2024, Gong and co-workers expanded this strategy to the asymmetric construction of β- and α-amino ketones (Scheme 97).^435^ The integration of tetra-*n*-butylammonium decatungstate with chiral sodium phosphate catalysts facilitated both alkane carbonylation/enantioselective Mannich-type transformations and alkane carbonylation/enantioselective radical addition cascades. This strategy enabled the asymmetric synthesis of β-amino ketones **764** (up to 99% yield, 98% ee) and α-amino ketones **765** (up to 99% yield, 95% ee) directly from simple alkanes, carbon monoxide, and anilines by shifting the equilibrium of the reversible photocatalytic C­(sp^3^)-H carbonylation step. Mechanistically, the acyl radical **766** underwent HAT process with [W_10_O_32_]^5–^H^+^ to generate the corresponding alkyl aldehyde, which could be trapped by amine to form imine intermediate **767**. In the presence of a ketone, an asymmetric Mannich reaction catalyzed by chiral sodium phosphate proceeds to furnish the β-amino ketone **764**. Conversely, in the absence of the ketone substrate, the imine can engage in an asymmetric radical addition with acyl radical **766**. Additionally, single-electron reduction of **767** by [W_10_O_32_]^6–^2H^+^ via a proton-coupled electron transfer (PCET) event followed by asymmetric radical coupling further contributes to product formation. β-Amino ketones, such as compounds **768**, **769**, and **770**, were successfully synthesized in this transformation under (*R*)-Na­[**5a**] conditions, while the corresponding α-amino ketones **771** and **772** were also obtained using the (*R*)-Na­[**5b**] catalyst.

**Scheme 97:**
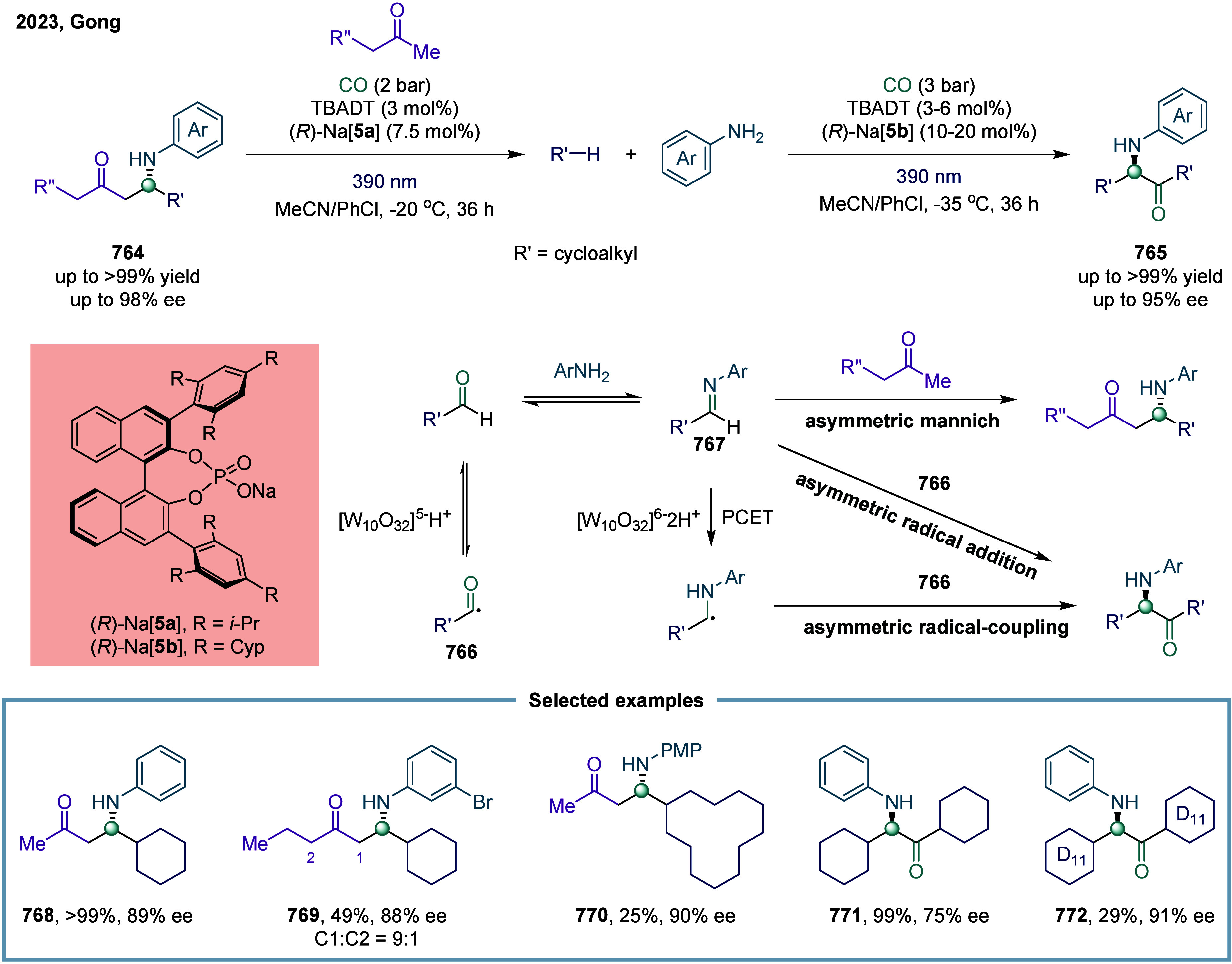
Enantioselective Synthesis of *β*- and *α*-Amino Ketones through Reversible Alkane Carbonylation

### 4.2 Iridium-Catalyzed System

In 1996, BP Chemicals developed the iridium-catalyzed *Cativa* process, which employs a cost-effective iridium catalyst that significantly reduces the water content required in the reaction mixture, thereby minimizing the formation of byproducts. Ir­(III) complexes, owing to their closed-shell electronic configuration and the pronounced spin–orbit coupling effects associated with their 5d electrons, have also been widely utilized as photocatalysts in SET-mediated carbonylation reactions. In such systems, Ir­(III) complexes function synergistically with visible-light irradiation to enable efficient and selective catalytic cycles.

#### 4.2.1 Carbon–Hydrogen Bonds

In 2024, Wu and co-workers reported a heteroarylative carbonylation of remote C­(sp^3^)-H bonds in tertiary alcohols, enabled by heteroaryl group migration (Scheme 98).^436^ This transformation involved the generation of alkoxy radical **759** via photocatalysis and employed carbon monoxide as a C1 building block to extend the carbon chain, thereby providing a favorable site for heteroaryl migration and facilitating a 1,4-HAT pathway. This strategy enabled the γ-C­(sp^3^) functionalization of alcohols, granting efficient access to 1,4-dicarbonyl compounds. Typically, 1,5-HAT processes were more prevalent; however, in the presence of CO, the formation of an acyl radical intermediate at the γ-position favored the 1,4-HAT pathway. This allowed the heteroaryl group, such as benzothiazole, to undergo 1,4-migration, ultimately delivering the major product. Mechanistically, radical intermediate **760** captured a molecule of CO to generate acyl radical intermediate **761**, which then underwent 1,4-migration of the benzothiazole moiety, forming intermediate **762**. Subsequent oxidation furnished the cationic intermediate **763**, which underwent deprotonation to yield the final product. It is notable that the migration of benzothiazole proceeded via a five-membered transition state, which inevitably led to the formation of byproduct. Various alkyl-substituted tertiary alcohols bearing *n*-propyl or *n*-butyl groups smoothly participated in this transformation, delivering the corresponding products **765** and **766** in 64% and 48% yields, respectively

**Scheme 98:**
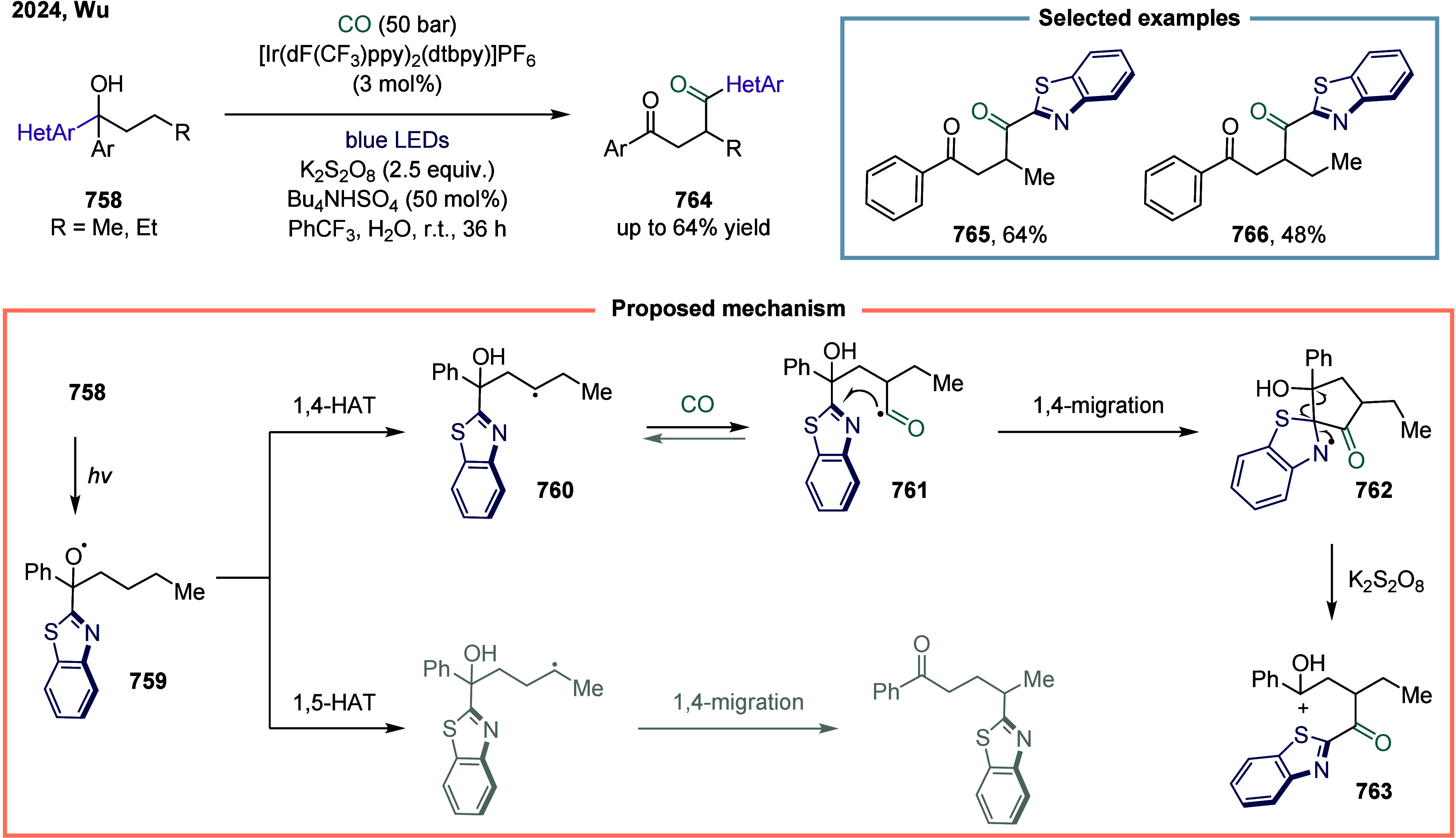
CO-Insertion-Enabled C­(sp^3^)-H Heteroarylative Carbonylation of Tertiary Alcohols via Heteroaryl Migration

#### 4.2.2 Carbon–Halogen Bonds

A mild and efficient method for the synthesis of alkyl amides from unactivated iodides, enabled by *fac*-Ir­(ppy)_3_ photocatalysis, was reported by Odell and co-workers in 2016 (Scheme 99).^437^ In this two-chamber setup, alkyl iodides, *fac*-Ir­(ppy)_3_, amines, a reductant, and carbon monoxide-released in situ from Mo­(CO)_6_, were combined to initiate a radical reductive dehalogenation, generating alkyl radicals. These radicals underwent carbonylation to form acyl radical intermediates, which were subsequently trapped by amine nucleophiles, affording a broad array of alkyl amides **767** in up to 90% yield.

**Scheme 99:**
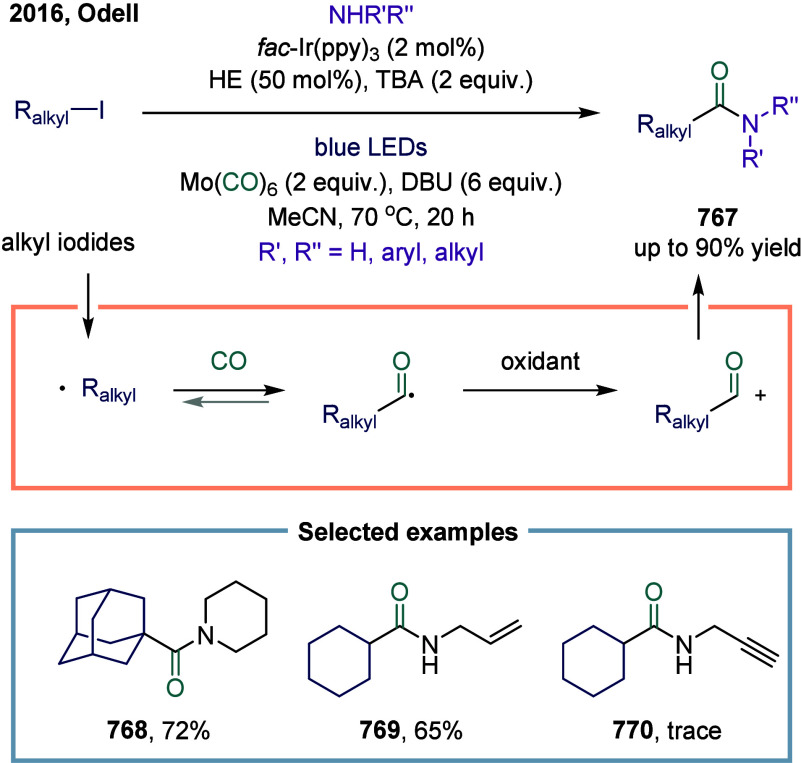
*fac*-Ir­(ppy)_3_-Mediated Aminocarbonylation of Unactivated Alkyl Iodides

Recent advances in carbonylation, driven by SET processes and photoredox catalysis, offered a promising alternative to traditional methods. In 2020, Polyzos and co-workers reported a visible-light-mediated aminocarbonylation of aryl, heteroaryl, and alkyl halides for the synthesis of diverse amides in up to 99% yield (Scheme 100a).^438^ This transformation employed a novel tandem photoredox catalytic system based on [Ir­(ppy)_2_(dtbpy)]^+^, which utilized a two-photon excitation cycle in the presence of DiPEA. This system generated a potent iridium photoreductant capable of activating a broad spectrum of aryl halides, including bromides, iodides, and chlorides, as well as alkyl halides. Mechanistically, sequential excitation and reductive quenching steps produced a highly reducing iridium species, [Ir-2]^0^*, from [Ir-1]^+^*. This intermediate facilitated electron transfer to otherwise inert halides, generating aryl and alkyl radicals that trapped CO to form acyl radical intermediates. Subsequent amine addition proceeded predominantly via a radical chain propagation mechanism involving α-hydroxy radicals **773**, as supported by DFT calculations and electrochemical studies. The reaction scope was further extended to include the ambient temperature amidation of methyl 4-chlorobenzoate, affording the corresponding amide **774** in 27% yield. Later in 2022, the same group developed a carbonylative hydroacylation of styrenes with alkyl halides by multiphoton tandem photoredox catalysis in flow (Scheme 100b).^439^ This protocol integrated a visible-light-driven multiphoton catalytic cycle of [Ir­(ppy)_2_(dtbpy)]^+^ with flow chemistry, enabling the transformation of energetically demanding alkyl bromides and iodides under CO atmosphere. Utilizing this mild and practical approach, 44 asymmetric dialkyl ketones, such as compounds **779**, **780**, and **781**, were synthesized from primary, secondary, and tertiary unactivated alkyl halides. The acyl radical underwent addition to the alkene, generating the benzylic radical intermediate. Subsequent radical polar crossover (RPC) furnished the corresponding carbanion **782**, which was ultimately protonated by water to yield the final product **778**.

**Scheme 100:**
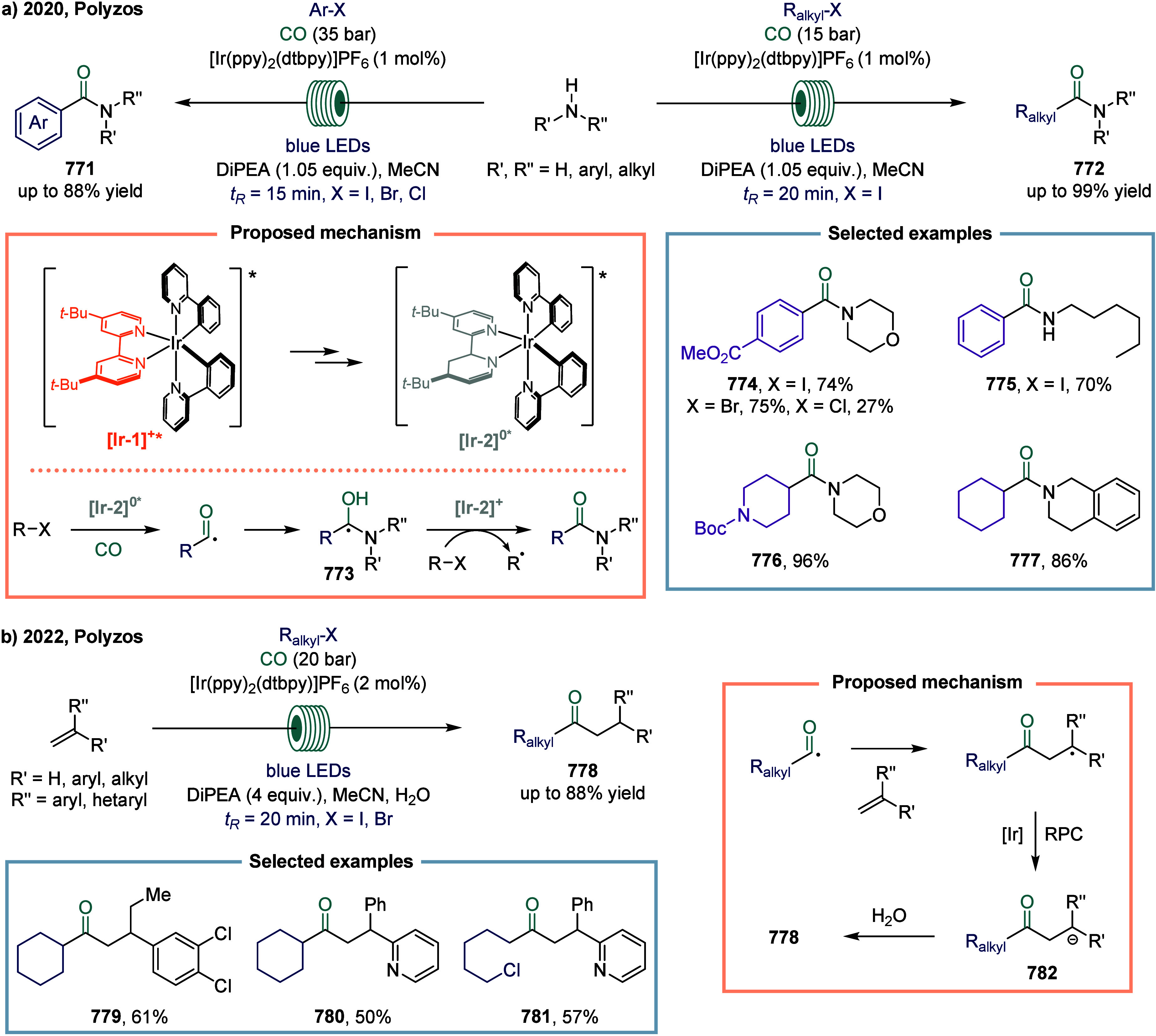
Visible-Light-Mediated Carbonylation of Aryl, Heteroaryl, and Alkyl Halides

#### 4.2.3 Unsaturated Bonds

In 2018, Polyzos and co-workers reported an annulative carbonylation of alkenyl-tethered arenediazonium salts using visible-light photocatalysis in continuous flow (Scheme 101).^440^ The key mechanism involved an aryl radical generated by SET process from the excited-state photocatalyst to the allyloxy-tethered arenediazonium salt, inducing homolytic cleavage of the C–N bond and addition to the alkene to deliver the primary radical species. The versatility of this transformation was further demonstrated by its compatibility with unsaturated *ortho*-tethered arenediazonium salts under the standard reaction conditions. For example, 3-acetate-functionalized 2,3-dihydrobenzofuran **785**, which featured an all-carbon quaternary center at the C3 position, was prepared in good yield from the corresponding substarte. Furthermore, the methodology proved amenable to structural diversification, as employing propargyloxy and 1-butenyloxy substituents provided straightforward access to the corresponding acetate-functionalized benzofuran **786** and chromane **787**, respectively.

**Scheme 101:**
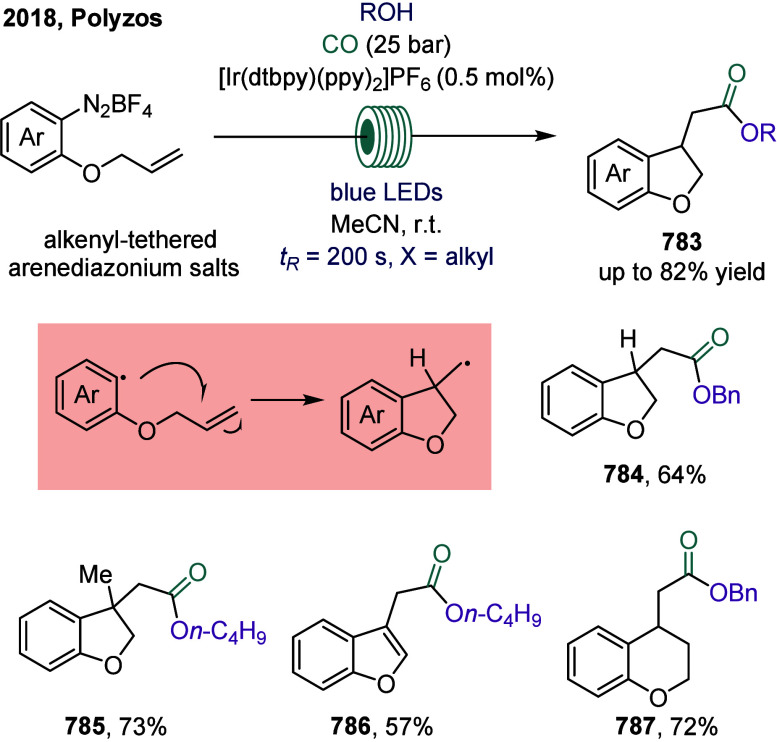
Radical Carbonylation Mediated by Continuous-Flow Visible-Light Photocatalysis

#### 4.2.4 Others

In 2024, Wu and co-workers reported a photoredox-catalyzed carbonylation of styrenes employing Hantzsch esters as radical precursors (Scheme 102a).^441^ The reaction proceeded under blue light irradiation to afford a series of alkyl ketones **788** in moderate to good yields under mild conditions. Subsequently, in 2025, the same group developed a visible-light-driven four-component carbonylation of styrenes with acyl azolium salts (Scheme 102b).^442^ In this transformation, acyl radicals generated from Hantzsch esters and carbon monoxide are added to the terminal position of styrenes. The resulting carbon-centered radicals then underwent further coupling with activated acyl azolium salts under light irradiation, furnishing the desired 1,4-diketone products **791**. Later in 2025, Wu and co-workers disclosed an *N*-heterocyclic carbene (NHC)- and photoredox-catalyzed carbonylation of styrenes (Scheme 102c).^443^ Under catalytic amounts of NHC, carbonylative diacylation of styrenes was achieved, delivering valuable 1,4-dicarbonyl compounds **792** in up to 89% yield.

**Scheme 102:**
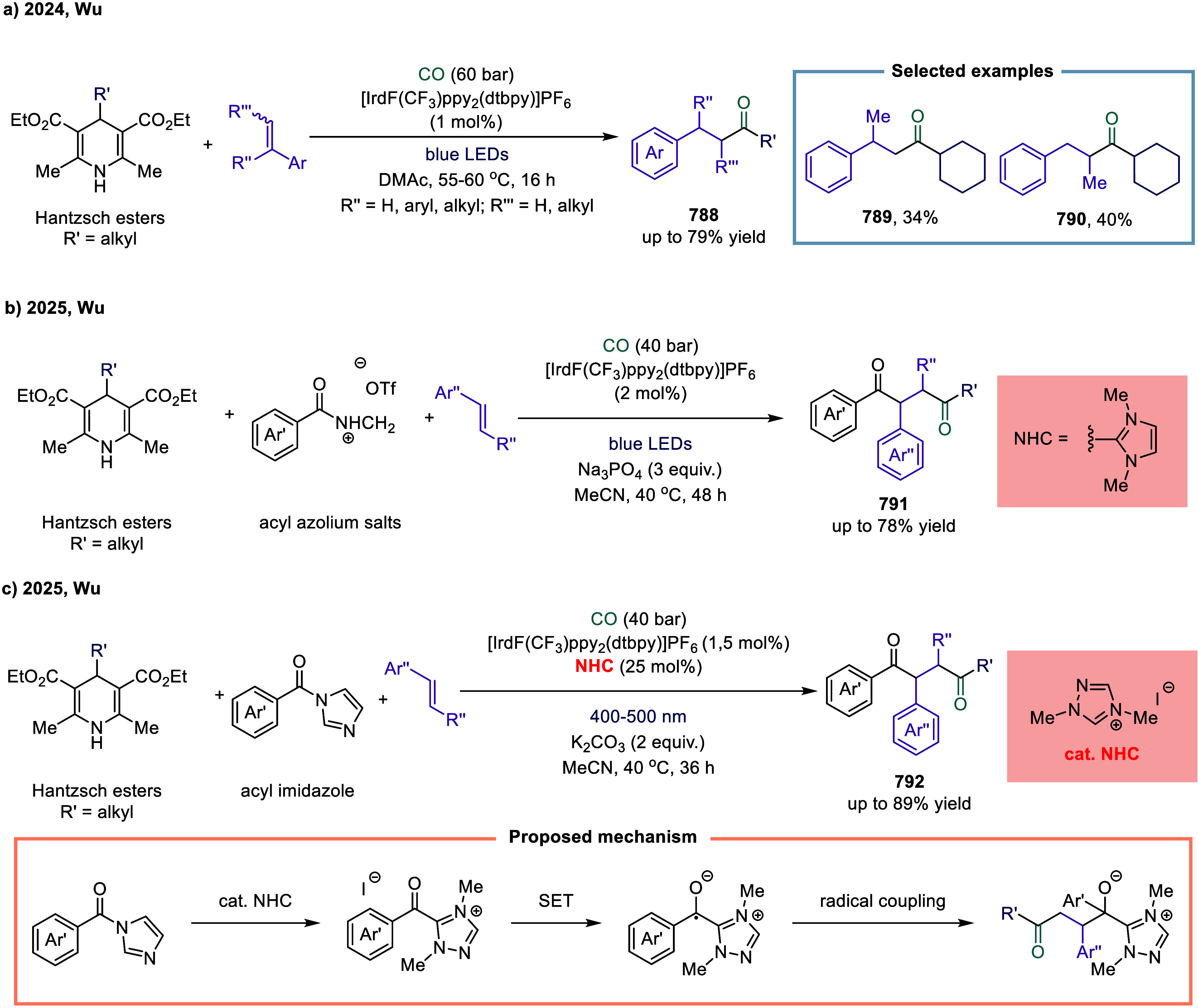
Photoredox-Catalyzed Carbonylation of Styrenes with Hantzsch Esters

Carboxylic acids are fundamental feedstocks that are produced on a large scale.^444,445^ In 2015, Xiao and co-workers reported a decarboxylative carbonylation of carboxylic acids enabled by visible-light photoredox catalysis, utilizing ethynylbenziodoxolones (EBX) as alkynylating reagents (Scheme 103).^446^ The transformation was initiated by single-electron oxidation of carboxylic acid by the excited state of an iridium photocatalyst, generating the corresponding alkyl radical. In the presence of CO, alkyl radical underwent carbonylation to afford acyl radical, which subsequently reacted with the EBX reagent to generate alkenyl radical intermediates along with a benziodoxolonyl (BI) radical. Triisopropylsilyl- and *tert*-butyl-substituted EBX reagents were well tolerated under the reaction conditions, affording the corresponding products **795** and **796** in yields of 56% and 27%, respectively.

**Scheme 103:**
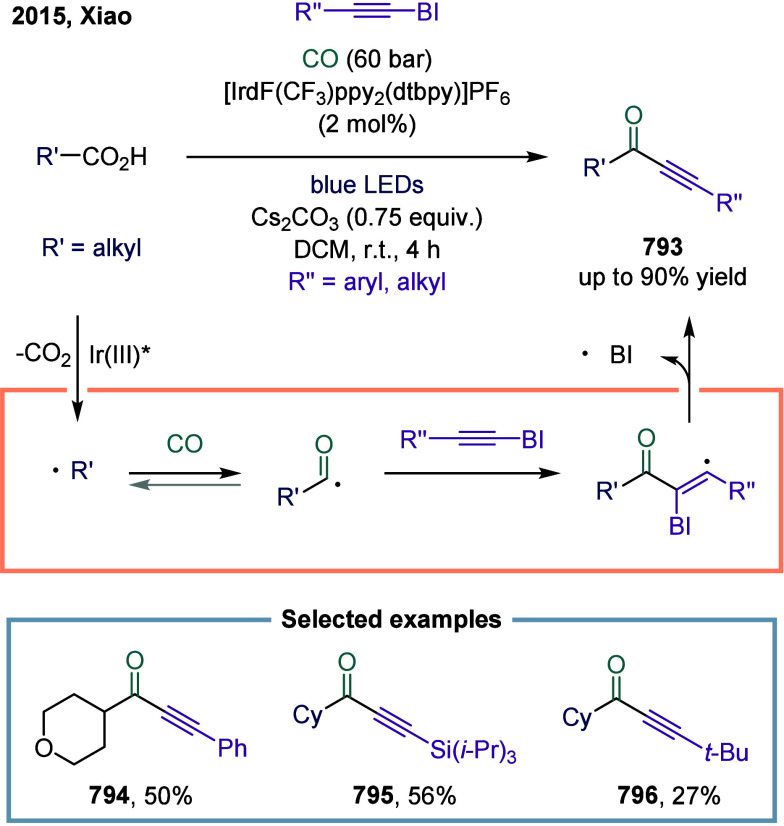
Decarboxylative Carbonylative Alkynylation of Carboxylic Acids Enabled by Visible-Light Photoredox Catalysis

Achieving controlled selectivity between single and double carbonylation from the same and simple starting materials is challenging. In 2022, Xiao and co-workers presented an innovative strategy for switchable radical carbonylation reactions driven by visible-light photoredox catalysis (Scheme 104).^447^ Mechanistically, the process was initiated by a SET reduction of redox-active compound by the excited-state photocatalyst *fac-Ir­(ppy)_3_^+^, generating an alkyl radical and the oxidized photocatalyst *fac*-Ir^IV^(ppy)_3_^+^, accompanied by the release of a carboxylate anion. Under a carbon monoxide atmosphere, the resulting alkyl radical readily underwent trapping by CO to form an acyl radical intermediate. In the first pathway, as demonstrated by the authors, the acyl radical engaged in a radical–radical coupling event with carbamoyl radical **799**, which was generated from amine via sequential SET oxidation and deprotonation, ultimately affording the α-ketoamide product **797**. Concurrently, the ground-state photocatalyst *fac*-Ir­(ppy)_3_ was regenerated, thereby completing the photocatalytic cycle. In contrast, the presence of DMAP promoted the addition of the acyl radical to DMAP, furnishing zwitterionic radical intermediate. Subsequent SET oxidation of the intermediate by *fac*-Ir^IV^(ppy)_3_^+^ generated the electrophilic acyl-DMAP complex. This activated species then underwent nucleophilic substitution with amine to deliver the monocarbonylation amide product **798**, again accompanied by regeneration of the ground-state photocatalyst. A variety of amines, including both aryl and alkyl amines, successfully underwent this transformation, affording the corresponding products **800**-**809** in good yields. In addition, this reaction demonstrated that a representative range of redox-active substrates could readily undergo the desired switchable radical carbonylation reactions under photoredox-catalyzed conditions.

**Scheme 104:**
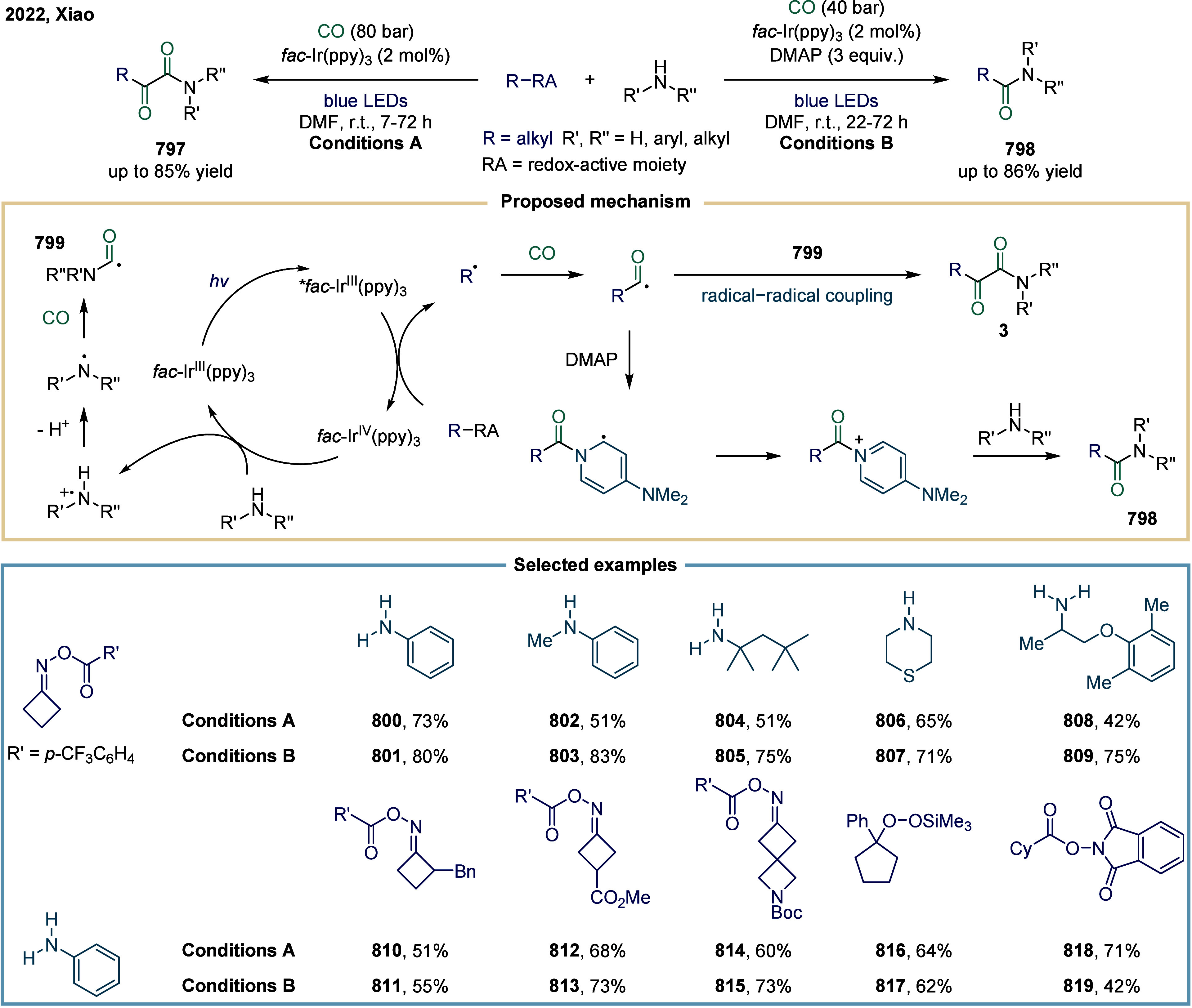
Photoredox-Catalyzed Single- and Double-Carbonylation of Redox-Active Moiety

β-Aminoketones, a vital class of organic compounds, constituted key structural motifs in numerous natural products, pharmaceuticals, and biologically active molecules. In 2025, Wu and co-workers reported a photopromoted carbonylative difunctionalization of alkenes for the synthesis of β-aminoketones **820** (Scheme 105).^448^ In this transformation, bench-stable alkyl oxime esters served as bifunctional reagents, generating both alkyl and imidyl radicals via energy transfer upon excitation by an iridium photocatalyst. This mild protocol exhibited high chemo- and regioselectivity, enabling the simultaneous installation of acyl and amine functionalities onto styrenes in a single step. A variety of β-aminoketones, including compounds **821** and **822**, were obtained in good yields. However, heterocyclic substrate **823** was unreactive under these conditions.

**Scheme 105:**
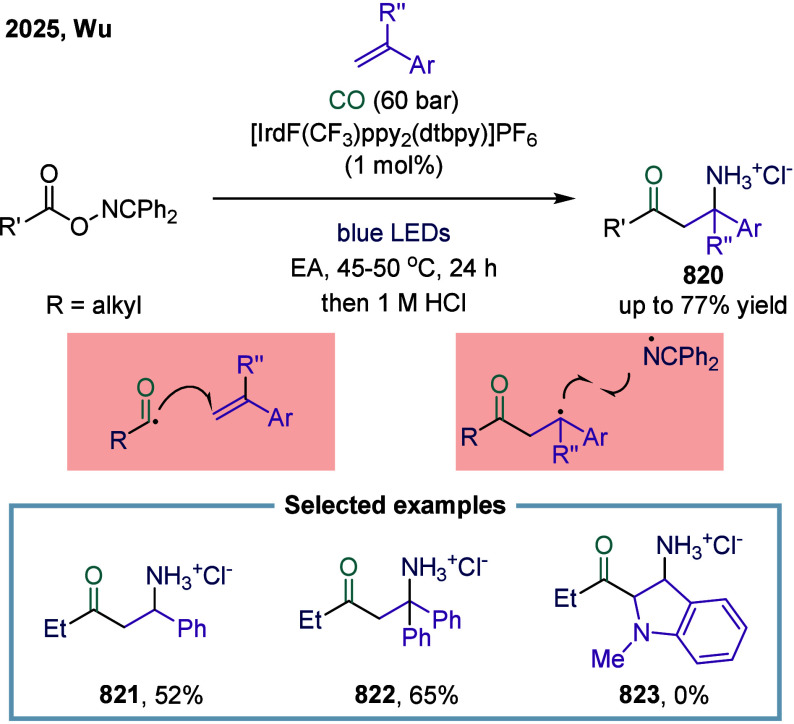
Photo-Promoted Difunctionalizative Carbonylation of Alkenes toward *β*-Aminoketones

In 2024, a visible-light-induced cooperative carbonylation and (hetero)­aryl migration for the synthesis of multicarbonyl compounds **824** (Scheme 106a).^449^ In this reaction, diazo compounds served as precursors to single-electron species. Under blue light irradiation, diazo compounds underwent a proton-coupled electron transfer (PCET) process with the excited-state iridium photocatalyst, resulting in extrusion of dinitrogen and generation of a nucleophilic carbon-centered radical. Subsequent trapping of CO followed by a favorable 1,4-heteroaryl migration delivered the final products. Both monoester- and diester-substituted diazo compounds participated smoothly in this transformation, affording the corresponding products **825** and **826** in yields of 59% and 72%, respectively. Subsequently, the same group reported a visible-light-promoted oxycarbonylation of unactivated alkenes (Scheme 106b).^450^ By leveraging a carbonylative heteroaryl migration strategy, the inherent challenge associated with the addition of oxygen-centered radicals to unactivated alkenes was successfully addressed. The alkoxycarbonyloxypyridinium salt was identified as an excellent precursor of oxygen-centered radicals in this transformation. Both aryl- and alkyl-substituted linear alkenes proved to be suitable substrates; however, alkyl-substituted alkenes afforded lower yields compared to their aryl-substituted counterparts. For example, compound **829** was obtained in only 34% yield.

**Scheme 106:**
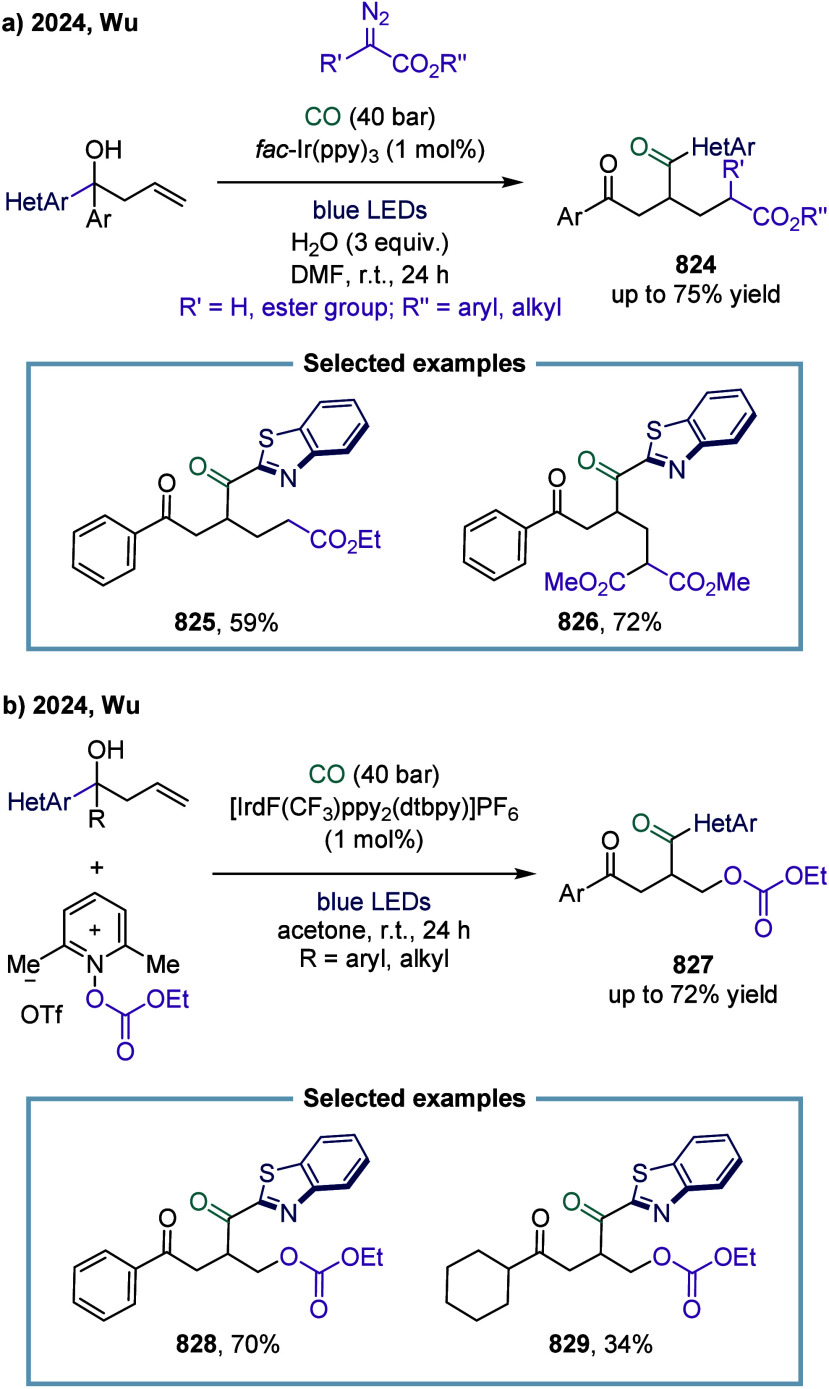
Visible-Light-Induced Carbonylation and (Hetero)­aryl Migration of Alkenes

In 2018, Xiao and co-workers reported a carbonylative Heck reaction for the synthesis of *α,β*-unsaturated ketones **830** (Scheme 107).^451^ Under blue LED irradiation, the [Ir^III^] photocatalyst was promoted to its excited state [Ir^III^]*, which was subsequently quenched via a SET process during the oxidation of Katritzky salt. In the context of alkyl-Heck-type reactivity, the resulting transient heteroaryl radical rapidly underwent C–N bond cleavage to furnish a stable pyridine species (Tppy) together with the generation of an active alkyl radical intermediate. This alkyl radical was initially trapped by carbon monoxide to form the corresponding acyl radical, which subsequently added to the olefin to generate a relatively stabilized radical adduct. Final oxidation and deprotonation of this intermediate delivered the desired enone product **830**. A series of representative Katritzky salts derived from primary alkyl amines, including both linear and cyclic amine substrates, were successfully employed in this transformation, affording the corresponding enone products in yields ranging from 60% to 74%.

**Scheme 107:**
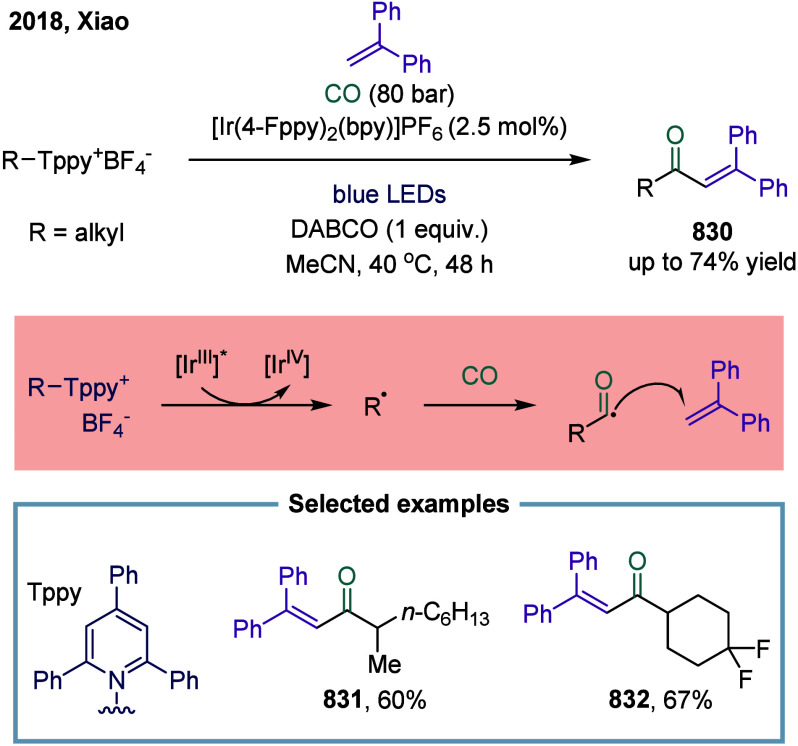
Deaminative Carbonylation Enabled by Photocatalytic C–N Bond Activation

## 5 Metal-Free System

With growing concerns over resource scarcity and environmental issues, the development of economically and environmentally sustainable synthetic methodologies has attracted increasing attention. Metal-free transformations, which avoid the use of expensive transition metal catalysts and eliminate the risk of metal contamination, represent an appealing synthetic approach. Accordingly, exploring transition metal-free carbonylation reactions holds promise for addressing many of the challenges associated with transition metal-catalyzed processes. However, due to the absence of metals capable of activating inert carbon monoxide molecules, the development of metal-free carbonylation remains a highly challenging area of research.

### 5.1 Thermally Mediated System

#### 5.1.1 Carbon–Hydrogen Bonds

Despite the outstanding achievements in C­(sp^3^)-H carbonylation reaction by utilizing transition-metal catalysts, such as palladium, cobalt, copper, and even decatungstate, analogous works were less applied in metal-free C­(sp^3^)-H carbonylation. In 2017, Lei and co-workers reported a metal-free radical oxidative carbonylation of alkanes to access the desired carbonyl products **833** in up to 86% yield (Scheme 108a).^452^ In the presence of 40 bar CO, the carbonylation of compounds **834** and **835** proceeded in 83% and 68% yields, respectively. Notably, the carbonylative coupling of adamantane afforded product **836** with a regioselectivity ratio of 2:1 and in up to 77% yield. Later in 2018, the same group reported a breakthrough method for the oxidative double carbonylation of alkanes using CO and amines to form α-ketoamides, facilitated by porous dual O- and N-doped carbon nanofibrous microspheres (CNMs) (Scheme 108b). The process was entirely transition-metal-free and relied on radical-mediated pathways, overcoming long-standing limitations in traditional carbonylation chemistry. CNMs synthesized from chitin, a natural and renewable biopolymer, possess a porous architecture, high surface area, and abundant O- and O-containing functional groups that collectively enable efficient adsorption of amines, suppress amine decomposition under oxidative conditions, and promote radical coupling steps.^453,454^ The corresponding α-ketoamides **837** were obtained with excellent functional tolerance and in up to 77% yield.

**Scheme 108:**
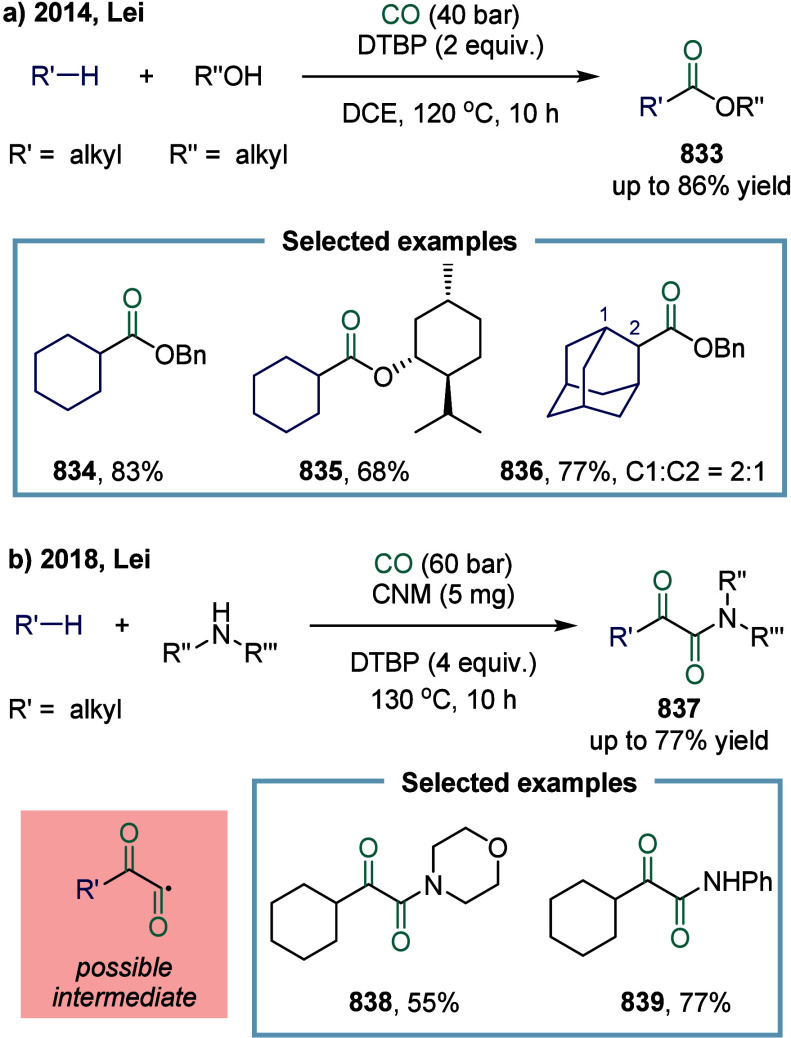
Metal-Free Radical Oxidative Single- and Double-Carbonylation of Alkanes

#### 5.1.2 Carbon–Halogen Bonds

In 2000, Ryu and colleagues reported the synthesis of diverse five- to seven-membered lactones by reacting hydroxyalkyl iodides with CO through an atom transfer carbonylation strategy that did not require transition metal catalysts (Scheme 109a).^455^ This transformation proceeded via a combined radical and ionic pathway, wherein the hydroxyacyl iodide intermediate, formed by atom transfer carbonylation, underwent intramolecular alcoholysis to furnish the corresponding lactones. Specifically, the key mechanistic steps involved the generation of single-electron species from hydroxyalkyl iodides by iodide atom transfer, followed by radical carbonylation to produce the acyl radical. This acyl radical then underwent intramolecular ionic quenching of the resulting acyl iodide to afford the lactone **840**, thereby shifting the equilibrium of the two reversible radical steps toward product formation. Moreover, a variety of hydroxyalkyl iodides served as suitable substrates, affording five-, six-, and seven-membered lactones (**841**-**844**) in good yields (58–84%). Another SET-mediated carbonylative cyclization initiated by AIBN via iodide atom transfer was also developed by the Ryu laboratory (Scheme 109b).^456^ Cyclohexanones were synthesized in good yields by allyltin-mediated three- and four-component cascade reactions involving (i) radical carbonylation, (ii) 6-endo cyclization, and (iii) alkene addition. In this transformation, 5-iodo-2-sulfonyl-1-pentene was subjected to typical tributyltin hydride-mediated radical carbonylation conditions, affording the corresponding 3-sulfonylcyclohexanone **845** in 62% yield. When 5-iodo-1-pentene was used as the substrate in the presence of allyltin and AIBN, 3-allylcyclohexanone **846** was obtained as the major product in up to 67% yield. Ryu previously demonstrated that allyltributyltin-mediated radical carbonylation offered an efficient approach for synthesizing a broad range of unsaturated ketones, wherein allyltin functioned as a unimolecular chain transfer (UMCT) reagent.^457,458^ The formation of the 6-endo product was likely attributed to the isomerization of the initially formed 5-exotype radical into a more thermodynamically stable 6-endo radical. This transformation was proposed to proceed through a sequential 5-exo cyclization, 3-exo cyclization, and β-scission pathway, a process facilitated by the relatively slow addition of the 5-exo radical to the allyltin reagent.

**Scheme 109:**
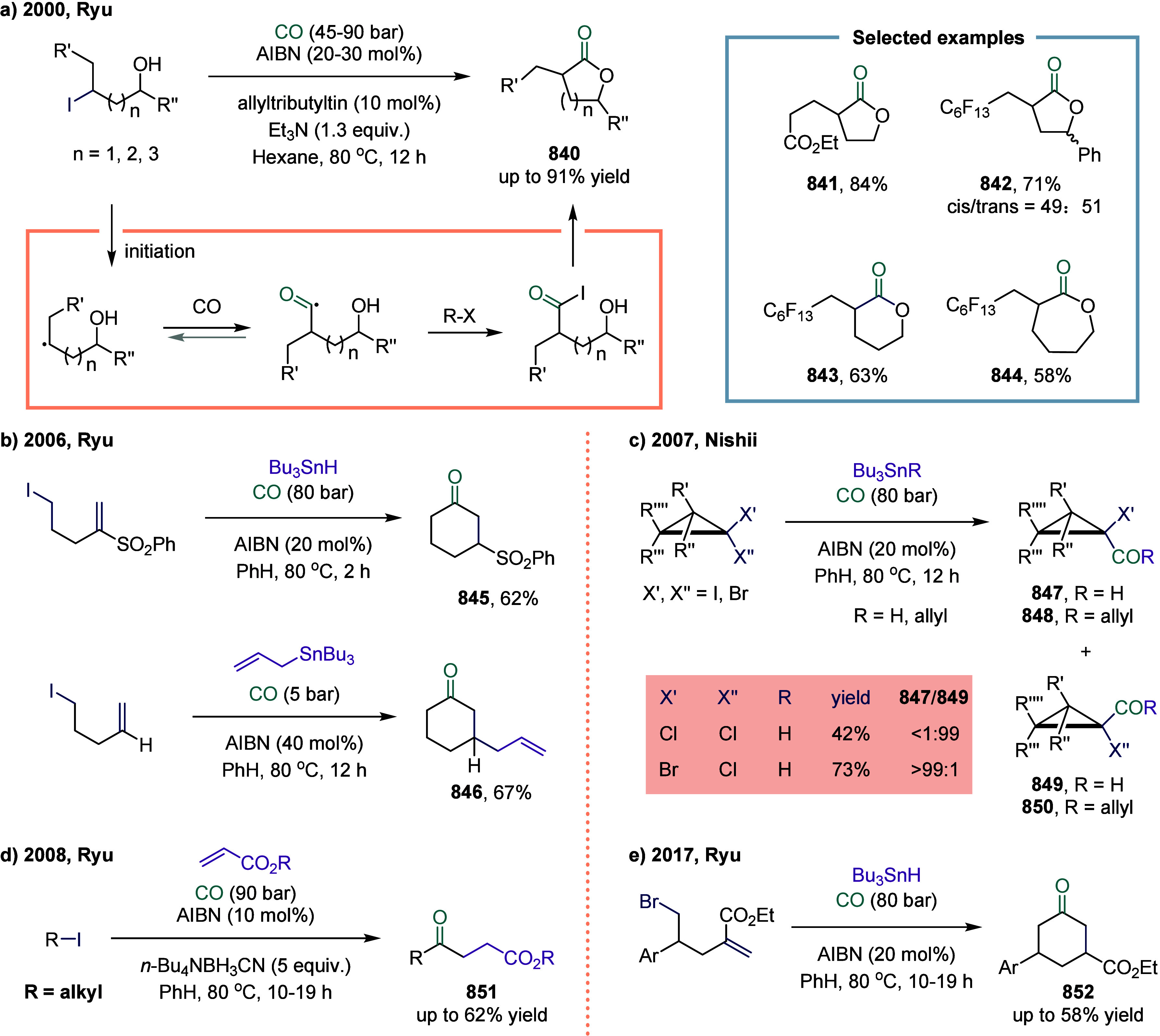
Metal-Free Carbonylative Coupling of Alkyl Halides Enabled by AIBN

In 2007, Tanabe and co-workers described the carbonylative coupling of *gem*-dihalocyclopropanes with CO in the presence of Bu_3_SnH or Bu_3_Sn­(CH_2_CH=CH_2_), which efficiently afforded trans- and cis-selective formylation and allylacylation adducts (**847**-**850**) with good to excellent stereocontrol (trans/cis ratios ranging from >99/1–75/25 and 17/83–1/99, respectively) (Scheme 109c).^459^ Notably, formylation of 2,3-cis-disubstituted 1,1-dihalocyclopropanes resulted in enhanced trans-selectivity (trans/cis >99/1–95/5), while both 2,3-cis-disubstituted and 2-monosubstituted substrates underwent allylacylation with almost complete trans-selectivity (trans/cis >99/1). Additionally, less reactive *gem*-dichloro- and bromochlorocyclopropanes proved to be suitable substrates, performing comparably to or even more favorably than *gem*-dibromocyclopropanes.

While tin-mediated generation of key radical intermediates has dominated metal-free carbonylation strategies, Ryu laboratory subsequently developed a tin-free Giese-type carbonylation protocol that proceeded efficiently using tetrabutylammonium cyanoborohydride as reductant (Scheme 109d).^460^ The reaction proceeded chemoselectively at the C–I bond, while leaving the C–Br and C–Cl bonds unaffected. A plausible mechanism involved iodine atom transfer, followed by hydride reduction of the resulting C–I intermediate. With this strategy, various unsymmetric ketones **851** were synthesized from simple alkyl iodides with moderate to good yields. Later in 2017, the same group subsequently reported a carbonylative radical cyclization employing AIBN, Bu_3_SnH, and carbon monoxide, which efficiently furnished the corresponding 3,5-disubstituted cyclohexanone derivatives **852** in moderate yields (Scheme 109e).^461^

In 2009, Ryu and co-workers extended this SET-mediated carbonylation strategy to a continuous microflow system, employing V-65 (2,2′-azobis­(2,4-dimethylvaleronitrile)) as a radical initiator and tributyltin hydride or TTMSS as radical mediators (Scheme 110).^462^ A variety of aldehydes **853** and unsymmetrical ketones **854** were obtained in good to excellent yields through radical formylation, carbonylative cyclization, and three-component coupling reactions.

**Scheme 110:**
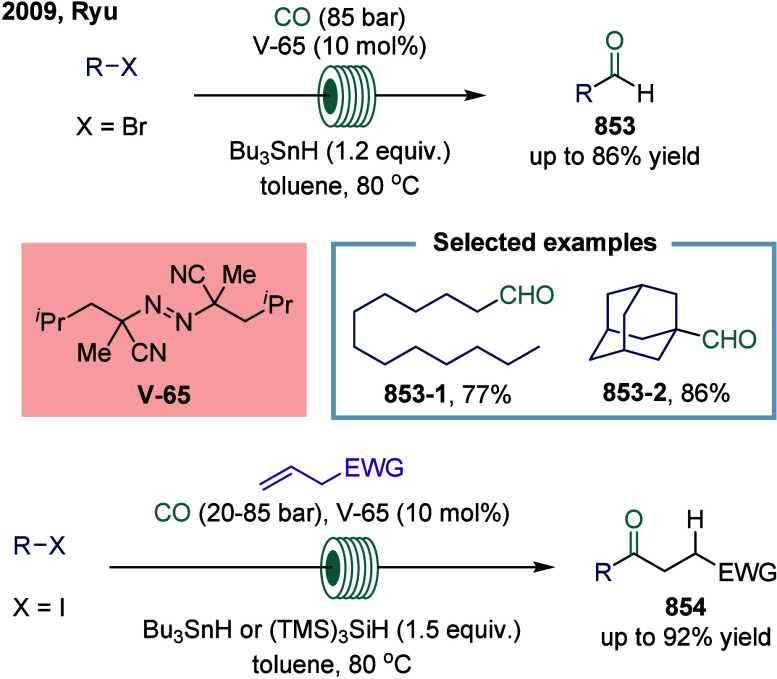
Metal-Free Radical Carbonylations Using a Continuous Microflow System: Synthesis of Aldehydes and Unsymmetrical Ketones

In 2012, Lei and co-workers disclosed a transition metal-free alkoxycarbonylation of aryl halides (Scheme 111a).^463^ Preliminary mechanistic studies revealed that an aryl halide undergoes single-electron reduction in the presence of 1,10-phenanthroline and KO*t*-Bu to generate an aryl halide radical anion, which subsequently eliminates X^–^ and traps CO to form an acyl radical. This intermediate further reacted with tert-butoxide to afford an ester radical anion. The resulting species transferred an electron to another molecule of aryl halide, thereby completing the catalytic cycle and ultimately furnishing the ester product. A variety of functional groups were tolerated in this reaction, affording the corresponding compounds **856**-**859**. This transformation constituted a convenient and efficient strategy for the synthesis of *tert*-butyl benzoate derivatives. Subsequently, the Fukuoka group developed a transition metal- and radical initiator-free carbonylative coupling protocol, which enabled the synthesis of aryl esters, carboxylic acids, and carboxylic acid anhydrides in up to 99% yields (Scheme 111b).^464^ These transformations proceeded simply by heating at 250–270 °C without the need for additional additives. The authors proposed that the reactions likely proceeded via a radical pathway involving SET processes. Aryl ester products **861**-**863** were efficiently synthesized from the corresponding sodium, potassium, and lithium salts, affording excellent yields. Additionally, the sodium and potassium carboxylate salts successfully underwent conversion to the corresponding anhydrides **865** and **866**, delivering 95% and 99% yields, respectively.

**Scheme 111:**
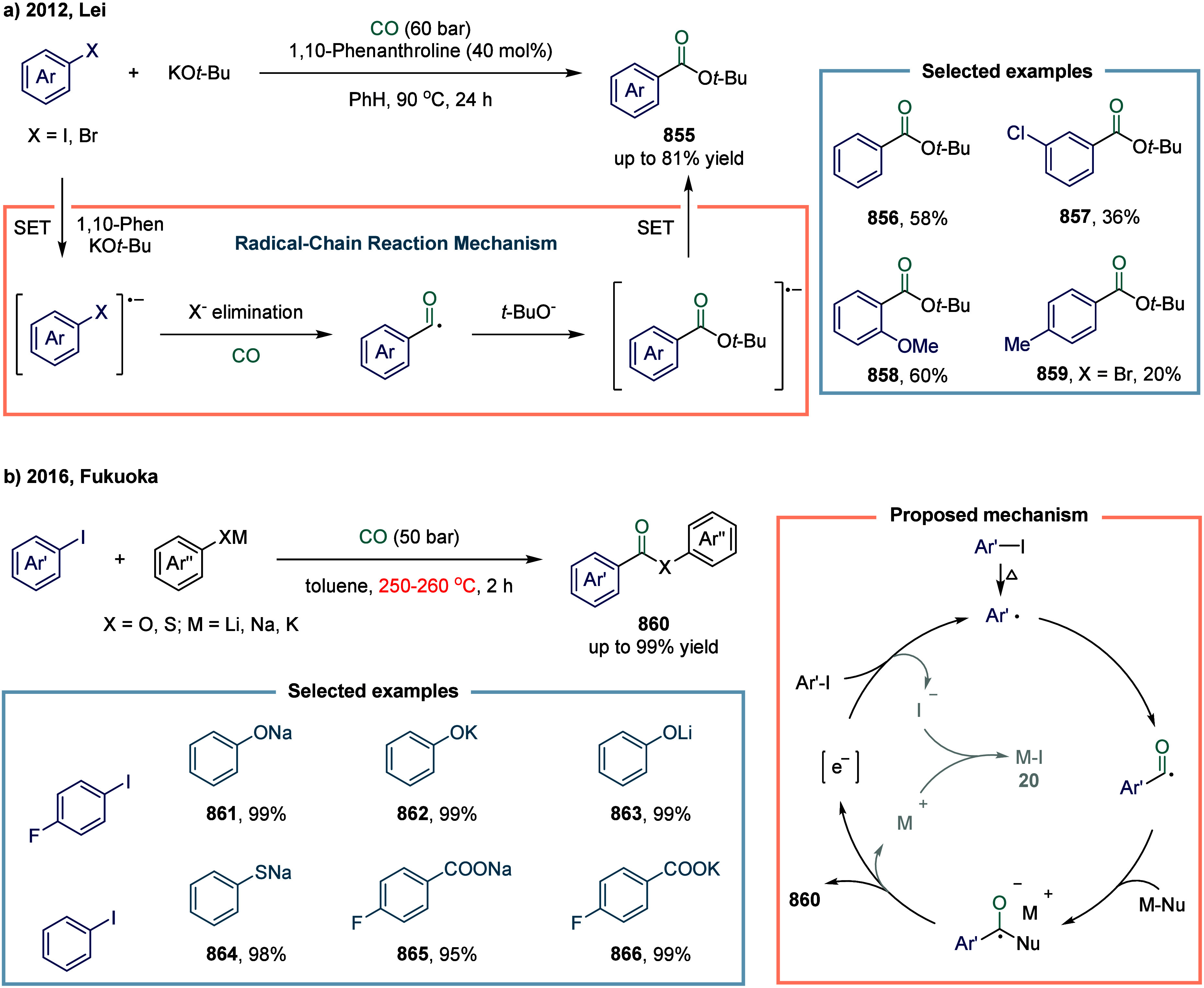
Metal-Free Carbonylations of Aryl Halides: Synthesis of Benzoic Acid Derivatives

In 2019, Han and co-workers disclosed transition-metal-free Suzuki carbonylative cross coupling of aryl halides with arylboronic acids by utilizing stoichiometric CHCl_3_ as the carbon monoxide precursor (Scheme 112a).^465^ Initially, aryl halides underwent base-assisted thermal dissociation to generate aryl radicals. These radicals subsequently trapped CO produced in situ from chloroform and CsOH·H_2_O, forming acyl radical intermediates. The acyl radicals then coupled with arylboronic acids in the presence of base to yield biaryl ketone radical anions. SET from these radical anions back to the aryl halides regenerated the aryl radicals and produced the desired biaryl ketones, thereby sustaining the radical chain process. Notably, various functionalities on aromatic ring (**868–872**), such as unprotected carboxyl and alkenyl group, were well tolerated under mild conditions. In 2019, the Han group reported a transition-metal-free carbonylative Suzuki-Miyaura reaction of aryl iodides with arylboronic acids, employing *N*-formylsaccharin as a CO surrogate (Scheme 112b).^466^ *N*-Formylsaccharin, initially employed by Manabe and co-workers in palladium-catalyzed reductive carbonylation of aryl halides, was applied as a carbon monoxide surrogate in this protocol.^467^ This protocol exhibited broad functional group tolerance on both coupling partners, affording a diverse array of biaryl ketones in good to excellent yields with high selectivity.

**Scheme 112:**
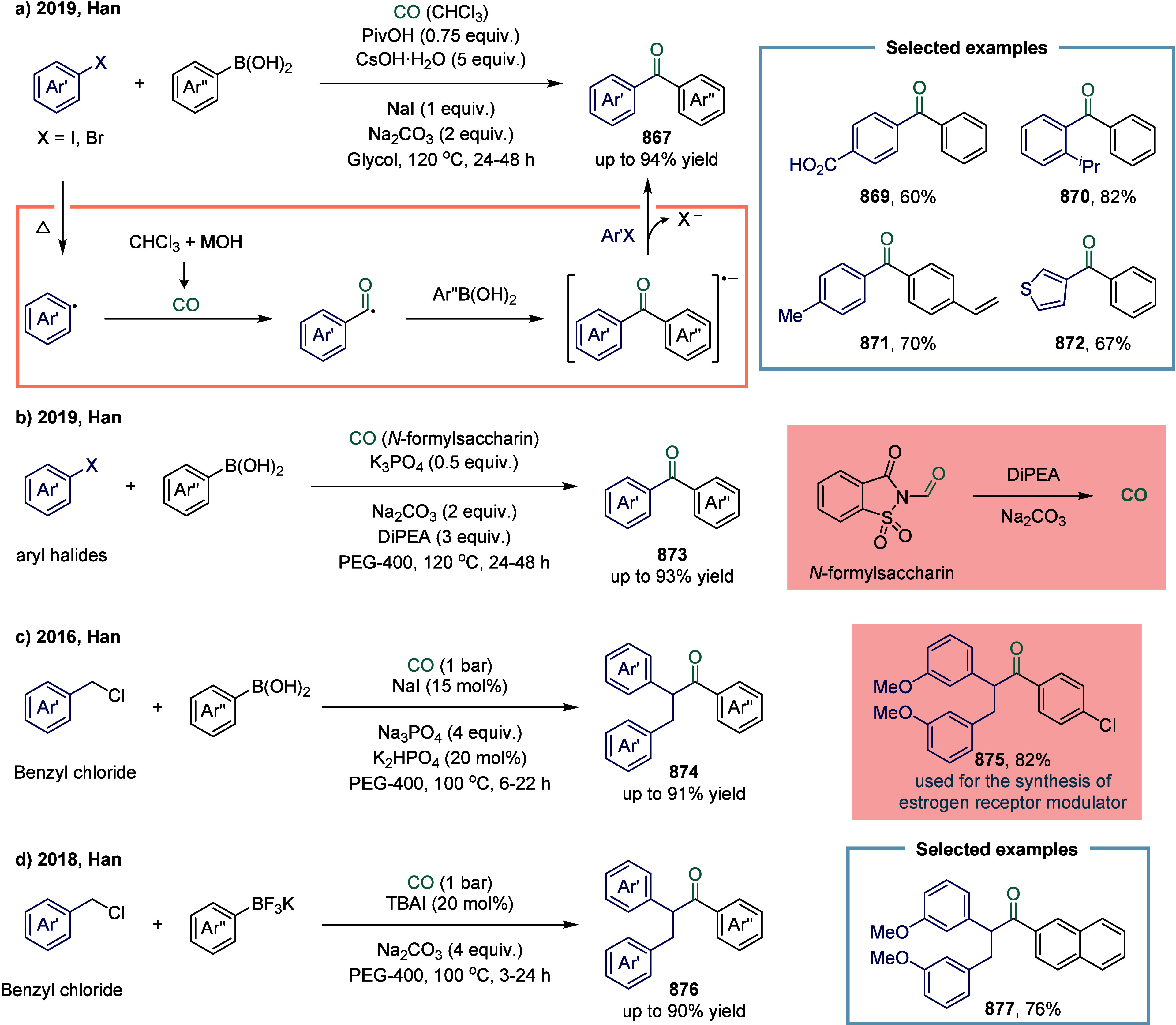
Metal-Free Carbonylation of Aryl Halides or Benzyl Chlorides: Synthesis of Ketones

In 2016, Han and co-workers a transition-metal-free, iodide-mediated domino carbonylation of benzyl chlorides with arylboronic acids (Scheme 112c).^468^ This innovative method represented a significant advancement over conventional palladium-catalyzed carbonylation protocols by employing NaI as a readily available, low-cost, and bench-stable catalyst. The transformation proceeded under considerably milder conditions and obviated the need for auxiliary ligands as well as the expensive purification procedures typically required to remove residual metal contaminants from the final products. Notably, a key intermediate **875**, essential for the synthesis of an important estrogen receptor modulator implicated in various cancer pathologies, was efficiently obtained in a single step, thereby circumventing the need for conventional multistep synthetic routes. In 2018, Han and co-workers extended the iodide-catalyzed carbonylation into benzyl chlorides with potassium aryltrifluoroborates, affording a variety of benzyl aryl ketones **876** in up to 90% yield (Scheme 112d).^469^

#### 5.1.3 Unsaturated Bonds

Metal-free radical cyclization offers a promising route to the construction of both carbocycles and heterocycles. For broad applicability, it is crucial to achieve high levels of regioselective control and to develop efficient protocols for the formation of small- and medium-sized ring systems. In 1998 and 2002, Ryu and co-workers demonstrated that the introduction of polar components enabled acyl radicals to undergo efficient and complete 5-exo cyclization onto the nitrogen atom of imines.^470,471^ Later in 2003, Ryu and co-workers utilized *α,β*-unsaturated acyl radicals as electrophilic species, with the imine functionality acting as the radical acceptor, thereby establishing a radical cyclization process notable for its broad generality (Scheme 113a).^472^ This cyclization proceeded with high regioselectivity, preferentially favoring nucleophilic attack at the nitrogen atom, and applied to the synthesis of 4- to 8-membered ring systems, as demonstrated by compounds **879**, **880**, and **881**. Moreover, the incorporation of CO effectively converted the polarity-mismatched vinyl radical intermediate into a polarity-matched radical species, which promoted efficient cyclization to afford the α-amino radical intermediate. Notably, this SET-mediated stannylcarbonylation of azaenyenes provided a general [n+1]-type condensation strategy for synthesizing α-stannylmethylene lactams. In 2004, Ryu and co-workers further extended this radical carbonylative cyclization process to 1,5-enynes by employing tris­(trimethylsilyl)­silane ((TMS)_3_SiH) as a chain carrier, affording the target product **882** in 27% yield (Scheme 113b).^473^ The reaction proceeded via a 5-exo cyclization of the acyl radical, followed by hydrogen atom abstraction from (TMS)_3_SiH. In 2007, Ryu reported the substitution of amine nitrogen by *α,β*-unsaturated acyl radicals, accompanied by the elimination of an α-phenethyl radical as a byproduct (Scheme 113c).^474^ In this transformation, acyl radicals bearing a benzylamine moiety underwent intramolecular cyclization to form a lactam through an S_H_i (intramolecular homolytic substitution) mechanism, which effectively released the benzyl radical and led to the formation of the lactam ring.

**Scheme 113:**
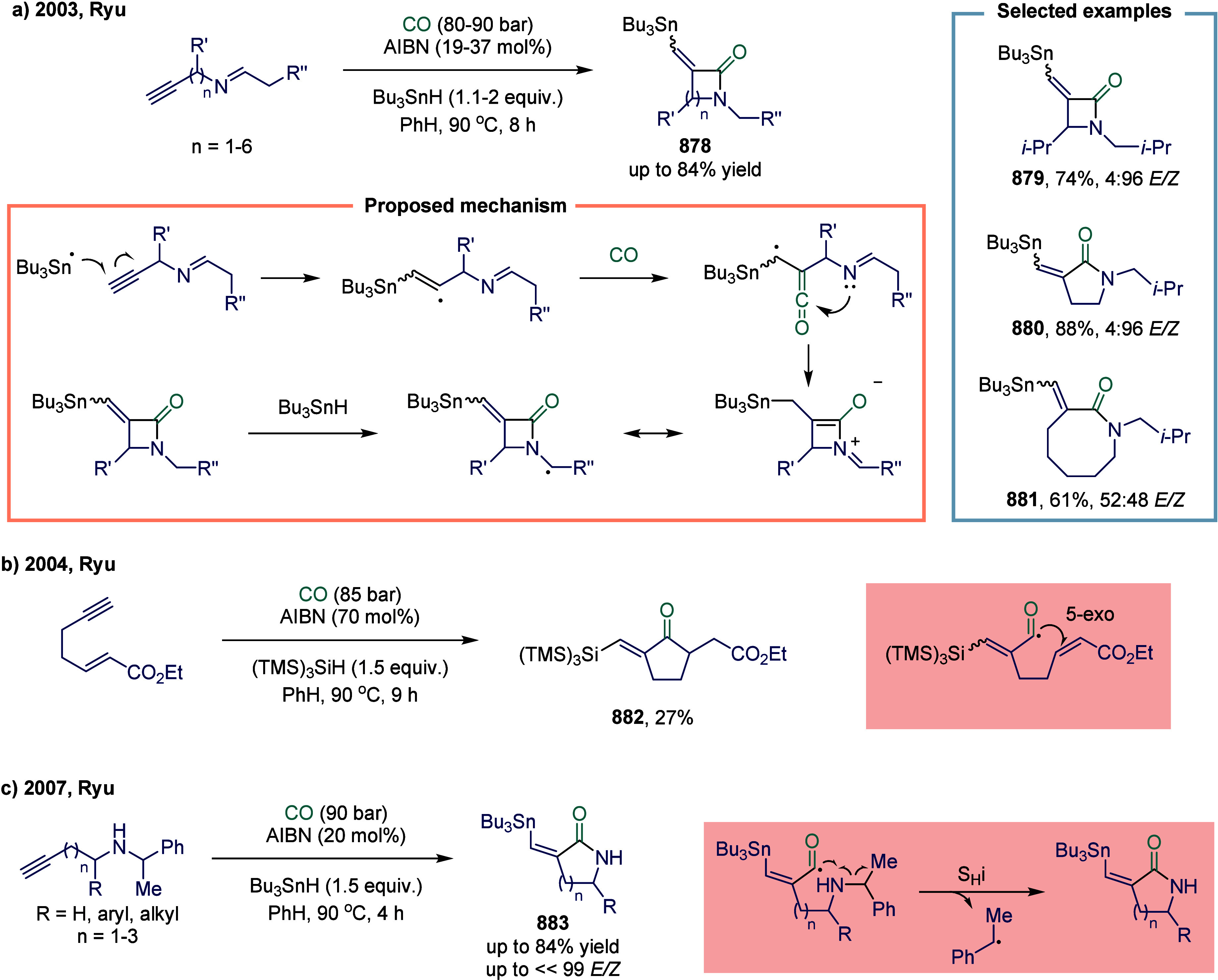
Metal-Free Intramolecular Carbonylation of Unsaturated Bonds Enabled by AIBN

Acrylamides and their derivatives serve as versatile building blocks in organic synthesis, participating in diverse transformations such as nucleophilic addition, cycloaddition, and radical-mediated processes.^475^ In 2005, Ryu and co-workers developed a metal-free carbonylation of alkynes and amines for the synthesis of α-methylene amides **884** (Scheme 114a).^476^ The authors proposed tin-radical-catalyzed hybrid radical/ionic mechanisms. Initially, tributyltin radical added to the terminal positions of the alkynes to generate vinyl radicals, which subsequently reacted with CO to form α-acylvinyl radicals. These intermediates were then trapped by amines to furnish 1-hydroxyallyl radicals. Subsequently, 1,4-hydrogen shifts generated α-keto radicals, which underwent β-fission to yield α-methylene amides while regenerating the tributyltin radicals. Various primary and secondary amines were compatible with the reaction, consistently delivering good yields. Terminal alkynes bearing diverse functional groups, including hydroxyl, chloro, phenyl thioether, and benzyloxy, underwent efficient carbonylation to afford the corresponding α-methylene amides **885**, **886**, **889**, and **890** in good to excellent yields. When substrates containing two alkynes were used, the reaction selectively targeted the terminal alkyne, producing compound **887**. Under the same conditions, phenylacetylene and pyrrolidine also reacted to give the corresponding amide **888**, albeit in moderate yield.

**Scheme 114:**
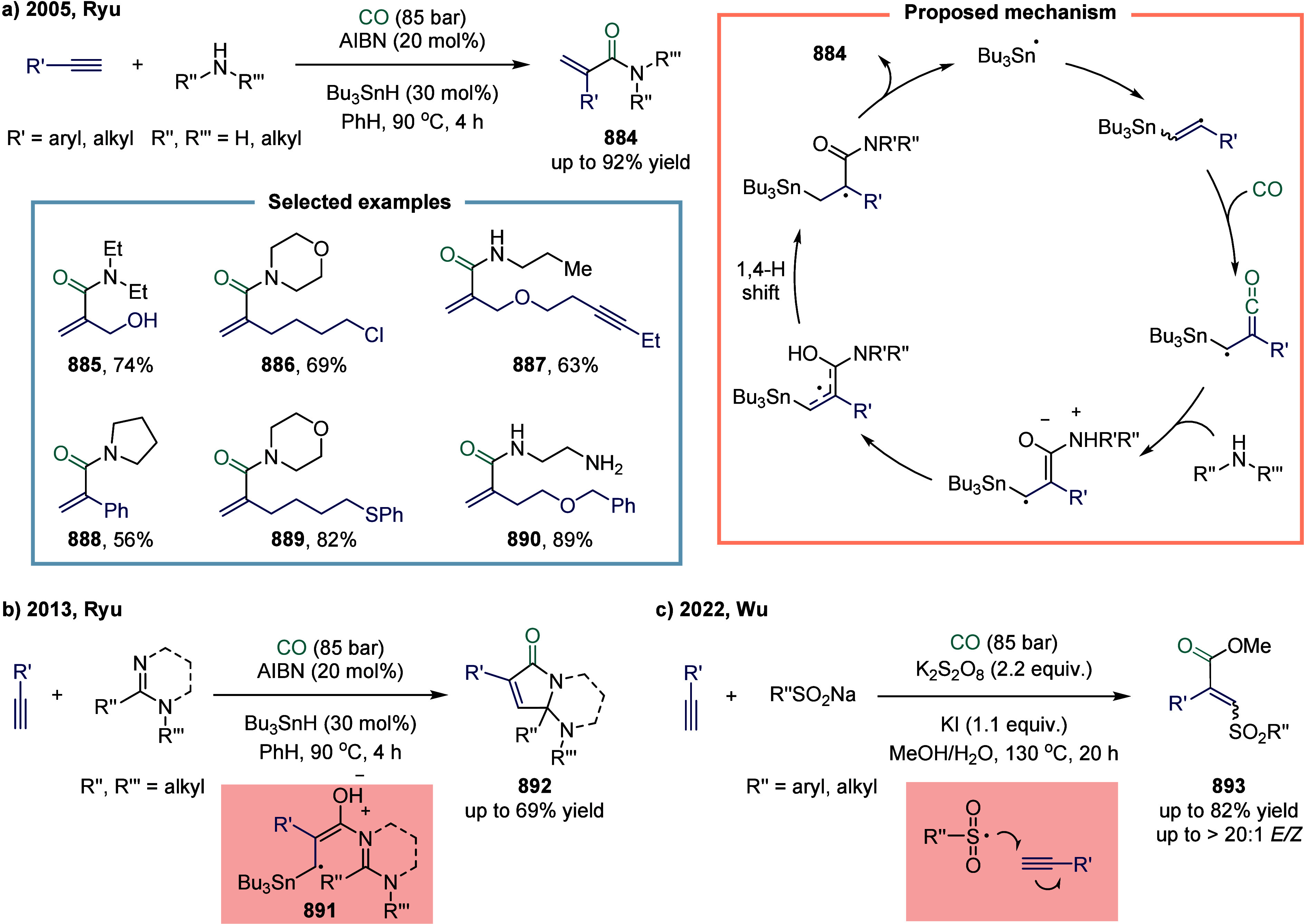
Metal-Free Carbonylation of Alkyne with CO

In 2013, Ryu and co-workers reported a SET-mediated aza-Pauson-Khand reaction involving an intermolecular [2 + 2 + 1] cycloaddition of acetylenes, amidines, and CO, which efficiently delivered various five-membered *α,β*-unsaturated lactams in up to 69% yield (Scheme 114b).^477^ Amidine facilitates the intermolecular trapping of the α-ketenyl radical, generating a highly conjugated and stabilized radical intermediate **891**. This species then underwent a five-membered ring closure, forming a distinctive heterocyclic framework. In 2022, Wu and co-workers reported a difunctional carbonylation of terminal alkynes with sodium sulfinates for the synthesis of olefin sulfonyl methyl esters **893** (Scheme 112c).^478^ A variety of olefin sulfonyl methyl esters were efficiently prepared through radical intermediates, achieving moderate to good yields. Importantly, this methodology proceeds without the need for costly metal catalysts or ligands. In 2005, Ryu and co-workers developed a streamlined one-pot protocol involving PRE-mediated radical 5-exo cyclization, followed by radical carbonylation, nitroxide trapping, and acid-promoted Friedel–Crafts-type acylation, enabling efficient access to 3,4-cyclopenta-1-tetralones.^479^

In 2024, Wu and co-workers successfully developed a trifluoromethylthiolation carbonylation of unactivated alkenes by employing AgSCF_3_ as a practical alternative to the Togni-II reagent under oxidative conditions (Scheme 115).^480^ This method utilized a distal migration strategy to achieve selective functionalization. A variety of corresponding products, such as compounds **895–897**, were successfully synthesized with moderate to good yields under the optimized conditions.

**Scheme 115:**
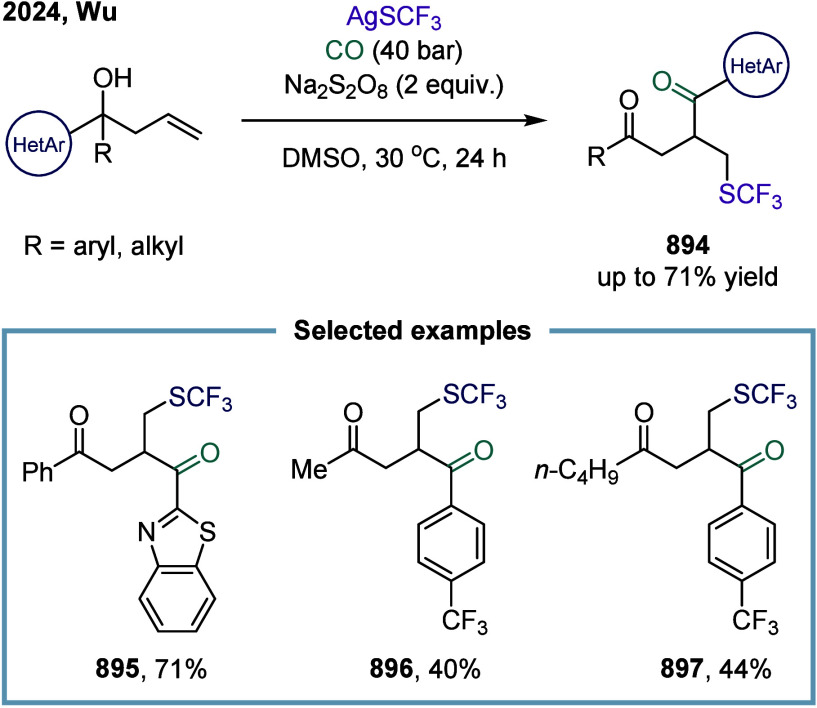
Trifluoromethylthiolation Carbonylation of Unactivated Alkenes via Distal Migration

#### 5.1.4 Others

Significant progress in organotin-mediated SET carbonylation reactions has been achieved, particularly under metal-free conditions. However, the high toxicity of organotin reagents has somewhat limited their broader application in organic synthesis. In 2005, Ryu and co-workers reported the use of alkyl allyl sulfone precursors as efficient and reliable alternatives for the generation of alkyl radicals under tin-free conditions, demonstrating excellent reactivity in SET-mediated carbonylation processes (Scheme 116).^481^ The transformation was initiated by the addition of a phenylsulfonyl radical to alkene, yielding an alkylsulfonyl radical intermediate. This intermediate underwent thermal desulfonylation, affording an alkyl radical along with the formation of phenyl allyl sulfone as a byproduct. The resulting alkyl radical was subsequently trapped by CO and/or phenyl benzenethiosulfonate, resulting in the formation of acyl radical intermediates or alkyl thioether products. Various thioesters, including compounds **899** and **900**, were synthesized in good yields. However, benzyl substrates **901** failed to undergo carbonylation, instead yielding thioether byproducts. In 2017, Wangelin and co-workers further optimized the metal-free carbonylative transformation of aryl diazonium salts (Scheme 117).^482^ This reaction proceeded via the generation of aryl radicals from arenediazonium salts under mild conditions, facilitated by the use of a weak base, sodium formate (HCO_2_Na).

**Scheme 116:**
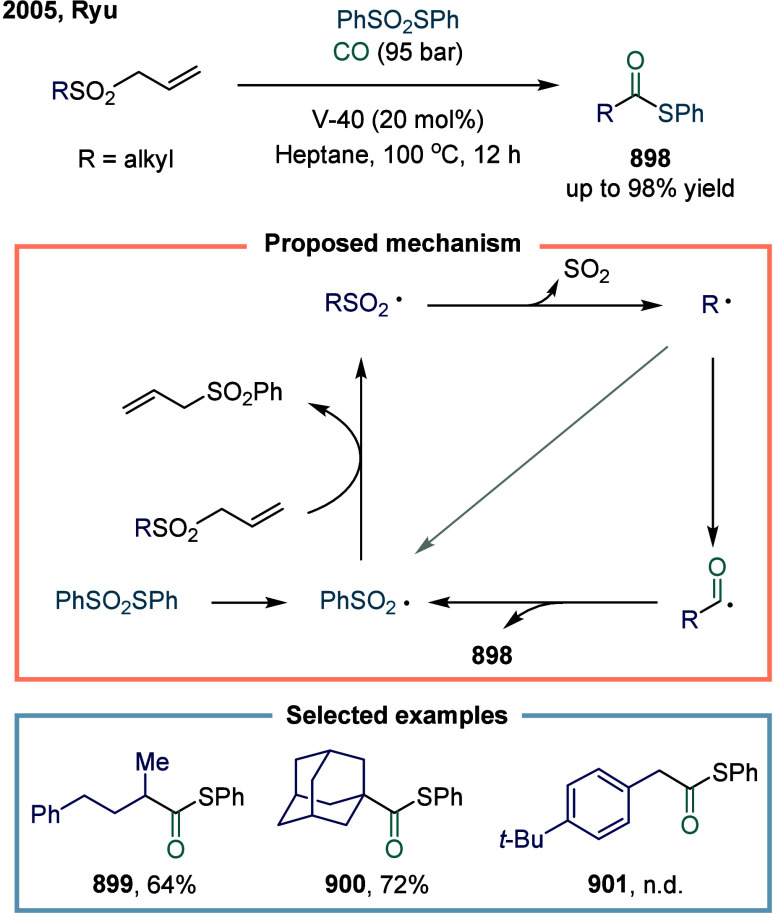
Tin-Free Radical Carbonylation: Thiol Ester Synthesis Using Alkyl Allyl Sulfone Precursors, Phenyl Benzenethiosulfonate, and CO

**Scheme 117:**
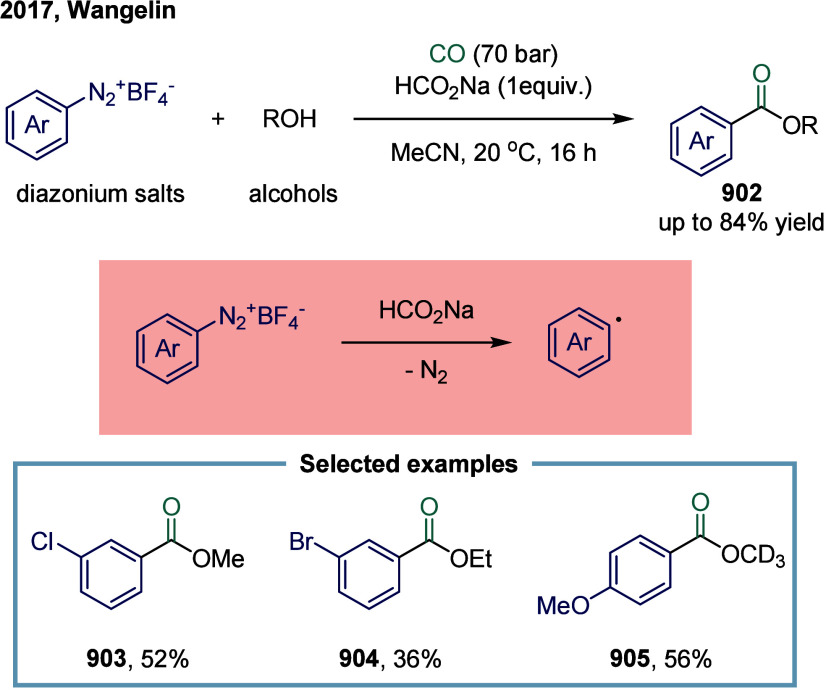
Metal-Free Radical Aromatic Carbonylations Mediated by Weak Bases

The synthetic utility of these salts in cross-coupling reactions was extensively demonstrated.^483–485^ In 2020, Wu and co-workers developed a transition-metal-free deaminative reaction that enabled efficient carbonylation of alkylamines with styrenes (Scheme 118a).^486^ Compared to the Katritzky salt decarbonylation reported by the Xiao group, which proceeded via visible-light-induced Ir-catalyzed C–N bond cleavage, this methodology achieved C–N bond carbonylation under metal- and photocatalyst-free conditions. A key intermediate in this transformation was the alkyl radical generated through base-promoted C–N bond cleavage of the Katritzky salt. Notably, di- and trisubstituted aryl alkenes exhibited good substrate compatibility in this reaction, as exemplified by compounds **907** and **908**. Furthermore, reactive functional group, such as hydroxyl group was well tolerated, affording the hydroxyl-containing product **909** in 61% yield with a diastereomeric ratio of 2.3:1. In the case of ester synthesis, various activated alkylamines were coupled with phenols and alcohols under mild CO pressures (1–6 bar), affording the corresponding products **911**-**913** in good yields and excellent selectivity (Scheme 118b).^487^ Subsequently, Wu and co-workers expanded the scope of coupling partners to strongly nucleophilic thiophenols. A range of thioesters, including compounds **915**-**917**, were obtained in moderate to excellent yields under mild reaction conditions (Scheme 118c).^488^

**Scheme 118:**
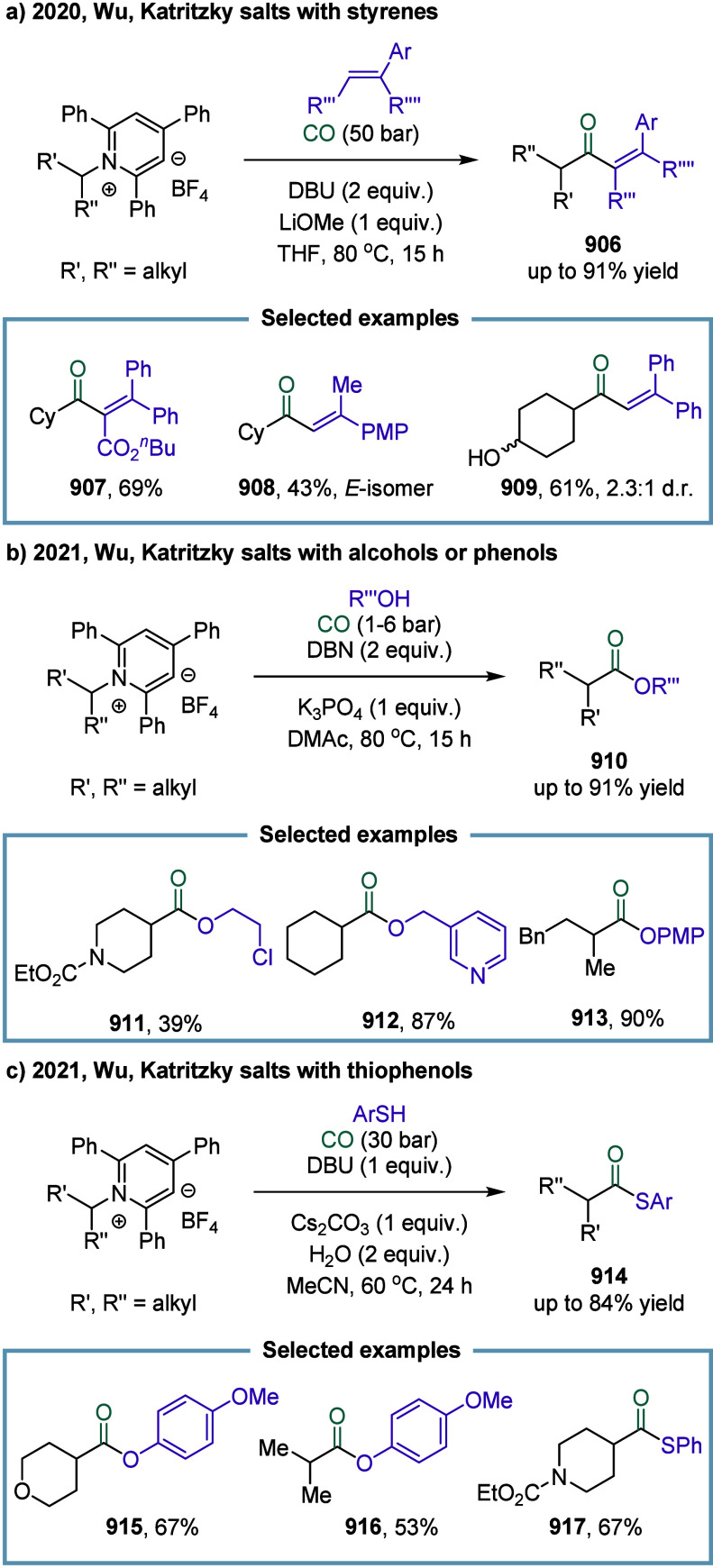
Metal-Free Carbonylation of Katritzky Salts

### 5.2 Visible-Light-Mediated System

#### 5.2.1 Carbon–Hydrogen Bonds

Li and co-workers employed *N*-alkoxyazinium salts as an alternative to DTBP for generating oxygen-centered radicals, successfully achieving the carbonylative coupling of simple alkanes with aryl alkenes under organic photoredox catalysis (Scheme 119).^489^ This metal-free strategy enabled the efficient synthesis of a broad range of *α,β*-unsaturated ketones **918**. Mechanistically, the excited state of 4CzIPN underwent oxidation by the *N*-alkoxyazinium salt, generating the oxidized photocatalyst [4CzIPN]^+·^ along with an isopropoxy radical. Subsequently, this radical abstracted a hydrogen atom from the alkane substrate via a HAT process, producing the corresponding alkyl radical and releasing isopropanol as a benign byproduct. The alkyl radical then underwent carbonylation and coupled with aryl alkenes to furnish the desired ketones, highlighting an efficient and environmentally benign approach to C–C­(O) bond formation.

**Scheme 119:**
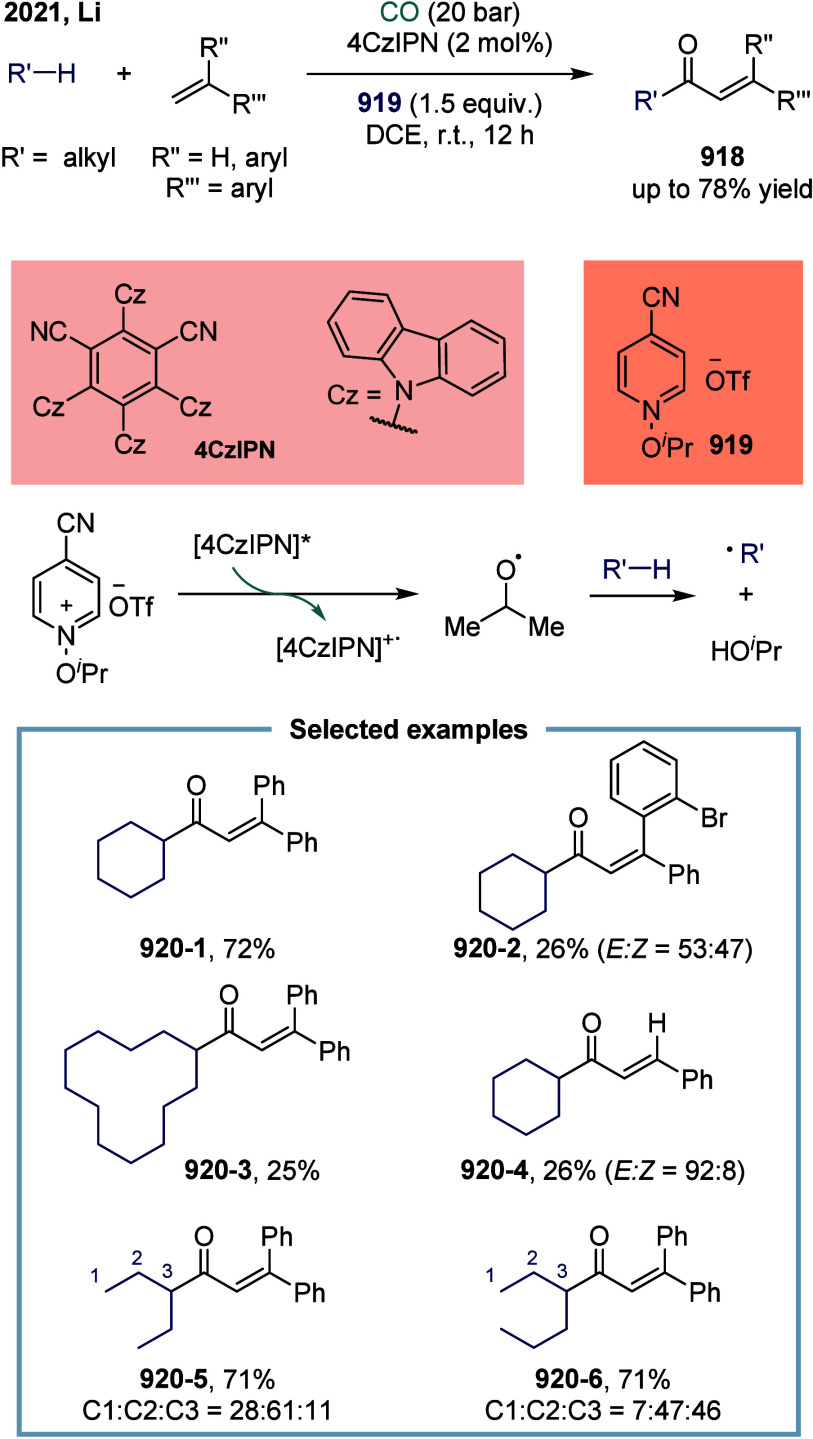
Carbonylative Coupling of Simple Alkanes and Alkenes Enabled by Organic Photoredox Catalysis

#### 5.2.2 Carbon–Halogen Bonds

Although AIBN-mediated generation of critical species has enabled a majority of C-X bonds carbonylation under metal-free conditions, Långström and co-workers disclosed a photomediated carbonylation of alkyl iodides with ^11^CO for the synthesis of [carboxyl-^11^C]­carboxylic acids **921** (Scheme 120a).^490^ To achieve high yields in reactions conducted in acetonitrile–water and THF–water mixtures, the addition of tetrabutylammonium hydroxide or potassium hydroxide was essential. Efficient carboxylation was observed for primary and secondary alkyl iodides. Among tertiary iodides, 1-iodoadamantane underwent successful carboxylation, whereas tert-butyl iodide **924** did not. This strategy provided an alternative synthetic route to [carboxyl-^11^C]-compounds. Then, Ryu and co-workers subsequently developed a tin-free, black-light-induced radical/ionic hydroxymethylation of alkyl iodides under atmospheric carbon monoxide, employing tetrabutylammonium borohydride as an efficient hydrogen donor (Scheme 120b).^491^ This protocol enabled smooth conversion of secondary and tertiary alkyl iodides via photoirradiation with black light, thereby avoiding the use of toxic tin reagents. A broad range of alkyl halides, including both bromides and iodides, was effectively transformed to afford the corresponding carbonylated alkyl alcohols **926** and **927**. However, when applied to functionalized iodolactones, the reaction efficiency significantly decreased, yielding low amounts of product **927** under both thermal and photochemical conditions, thus highlighting the substrate sensitivity of this transformation.

**Scheme 120:**
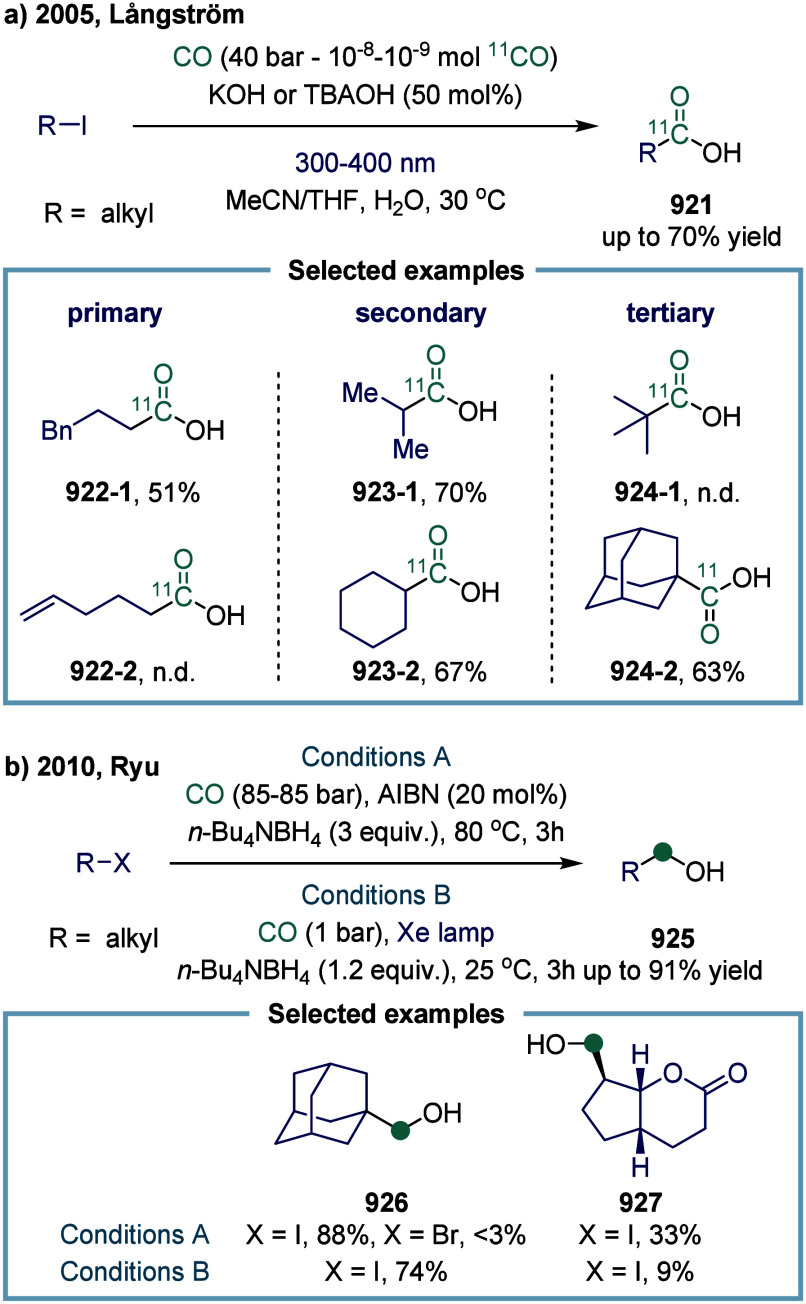
Metal-Free Carboxylation of Alkyl Iodides: Synthesis of Acid and Alcohols

In contrast to classical photoinduced metal-free carbonylation of carbon-halide bonds, which typically required high-energy Xe lamps for photoexcitation, advances in photochemistry had enabled SET-mediated carbonylation to proceed under low-energy visible light irradiation. In 2023, Wu and co-workers reported a photoinduced phosphine-catalyzed alkoxycarbonylation of alkyl iodides with phenols, affording a variety of alkylphenol esters **928** in up to 97% yield (Scheme 121a).^492^ The transformation began with the formation of an EDA complex between alkyl iodides and PCy_3_, which fragmented under blue light irradiation to generate a phosphinium radical ion pair and an alkyl radical. The resulting alkyl radical trapped carbon monoxide to furnish an acyl radical intermediate **8**. This acyl radical could undergo an atom transfer carbonylation (ATC) process to deliver the acyl iodide species or be oxidized by the intermediate to produce the corresponding acyl cation. Iodide- and hydroxyl-substituted phenols were successfully transformed into the corresponding esters **929** and **930** in 82% and 44% yields, respectively. A tertiary alkyl iodide, such as 1-iodoadamantane, was also smoothly converted to the desired product **932** in up to 94% yield. Moreover, various ^13^C-labeled alkylphenol esters, including **933** and **934**, were obtained in excellent yields under 1 bar of ^13^CO, providing opportunities for applications in isotopically labeled drug development.

**Scheme 121:**
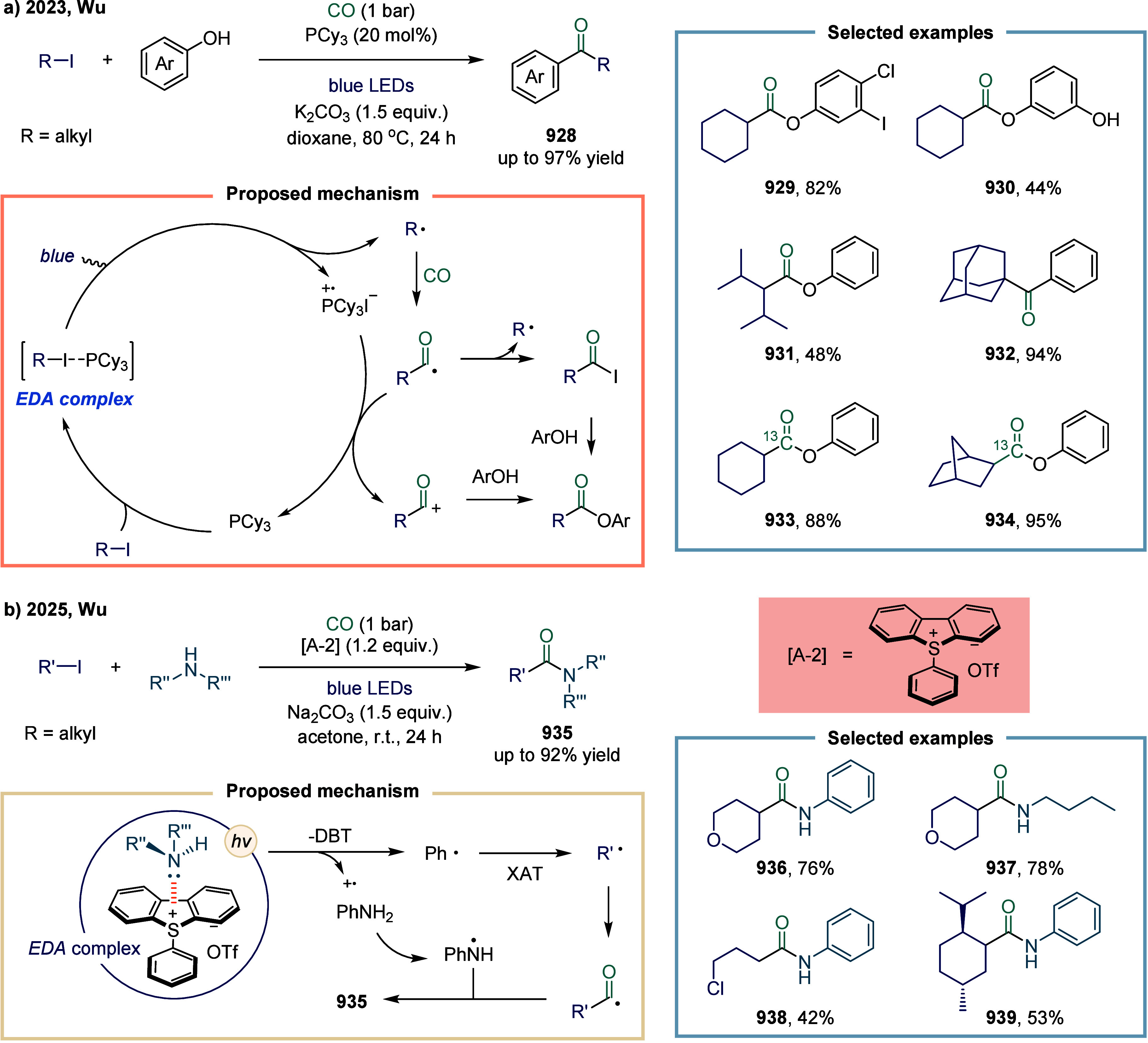
Metal-Free Photo-Induced Carbonylation of Alkyl Iodides to Esters and Amides via EDA Complex

Subsequently, in 2025, the same research group established a dual EDA/XAT-promoted sustainable carbonylative transformation of alkyl iodides and amines to access amides **935** in up to 92% yield (Scheme 121b).^493^ By leveraging the XAT strategy, this protocol circumvented the need to overcome the highly negative reduction potential of the C­(sp^3^)-halogen bond (E_red_ ≪ −2.0 V vs SCE), thereby significantly reducing reliance on transition metals. Mechanistically, the reaction proceeded via the formation of an EDA complex between phenyl sulfonium salts and aniline, which, upon visible-light irradiation, generated phenyl radicals and an aniline-derived cationic intermediate. The phenyl radical subsequently activated the alkyl iodide to form the corresponding alkyl radical, which, under a CO atmosphere, trapped carbon monoxide to furnish an acyl radical species. Concurrently, deprotonation of the aniline cation afforded a nitrogen-centered radical, which rapidly coupled with the acyl radical to deliver the desired amide product. This methodology exhibited a broad substrate scope and provided a diverse array of amides, including compounds **936–939**, in good to excellent yields. Notably, this approach overcame the limitations associated with the use of high-pressure carbon monoxide in metal-free carbonylation by enabling efficient radical coupling, thereby further expanding the synthetic utility of carbonylative processes.

In 2024, Miyake and co-workers introduced an innovative strategy for the carbonylation of alkyl halides with amines or alcohols to access carbonyl derivatives **940** in yields of up to 86% under visible-light irradiation (Scheme 122).^494^ Upon blue light exposure, 4-DPAIPN was excited to its singlet state 4-DPAIPN*, which engaged in a direct electron transfer with triethylamine, generating the radical anion intermediate. This intermediate subsequently reduced the alkyl halide to furnish the corresponding alkyl radical, which underwent single-electron oxidation to produce a carbocation species. The carbocation readily captured carbon monoxide to form an acylium ion intermediate. The final carbonylated products were obtained through nucleophilic addition of alcohols or amines to this reactive intermediate. Notably, primary iodides afforded the corresponding product **942** in up to 35%yield when benzyltriethylammonium was used as the electron donor and THF served as the reaction solvent.

**Scheme 122:**
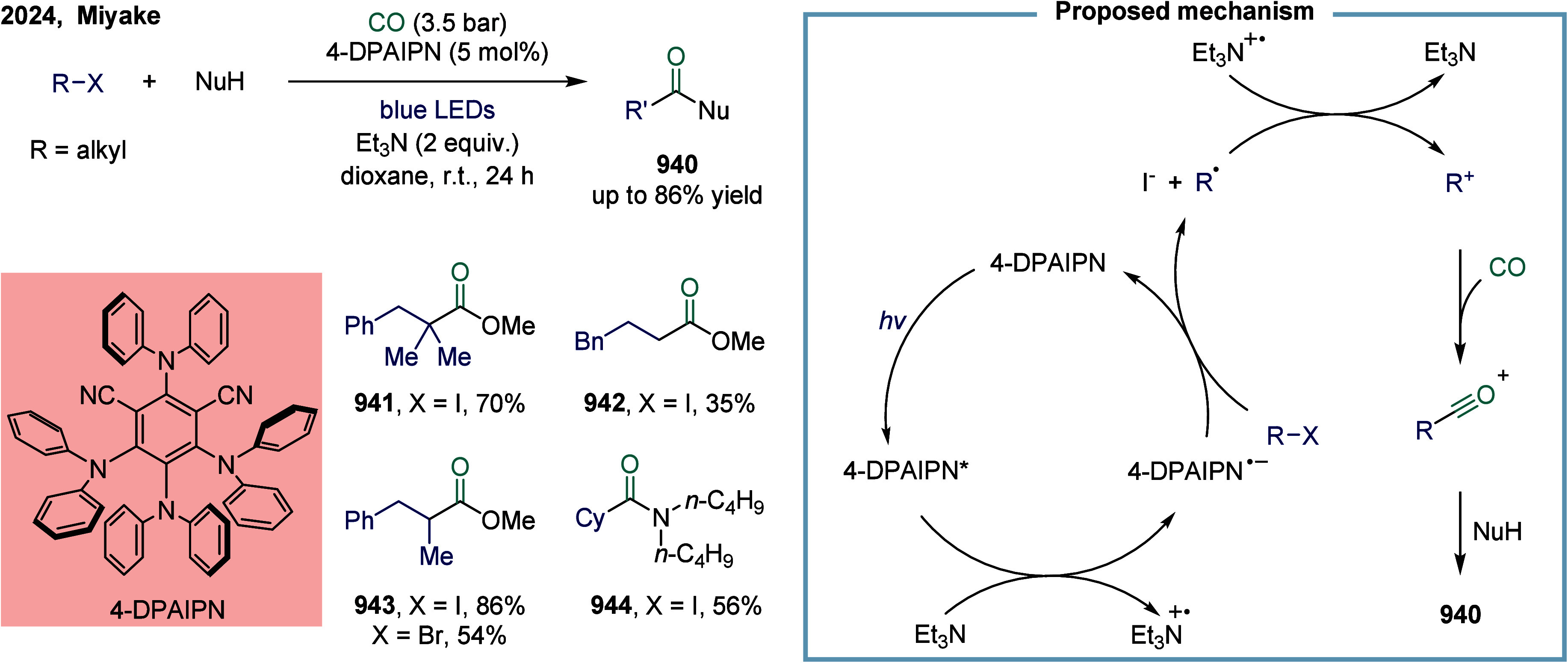
Organocatalyzed Carbonylation of Alkyl Halides Driven by Visible Light

The metal-free carbonylation coupling of aryl halides has been further studied with a variety of nucleophilic coupling partners, employing light instead of heat to promote the transformation. Ryu and co-workers reported a photoinduced aminocarbonylation of aryl iodides with CO and amines (Scheme 123a).^495^ In this transformation, high-temperature conditions to generate single-electron intermediates were unnecessary, as the reaction proceeded via photoinduced cleavage of the aryl iodide. The authors proposed a hybrid radical-ionic chain mechanism in which electron transfer occurred from zwitterionic radical intermediates, generated by the nucleophilic attack of amines, to the aroyl radicals. This methodology showed broad functional-group tolerance, including that of heteroaromatic amides. This methodology exhibited broad functional group tolerance, accommodating a wide range of substituents such as ester and acyl groups, and demonstrated excellent compatibility with heteroaromatic amide **948**. Later in 2018, the same group reported an electron-transfer-induced intramolecular Heck carbonylation of benzyl alcohols and benzyl amines with CO under heating at 250 °C or Xe lamp irradiation afforded the corresponding benzolactones and benzolactams **950** in up to 95% yield (Scheme 123b).^496^ Heating the reaction mixture to 250 °C for 16 h resulted in excellent reactivity. The authors also investigated photoirradiation conditions using a 500 W xenon lamp and a quartz tube, which afforded comparable results but required a longer reaction time. This transformation likewise proceeded via a hybrid radical-ionic chain mechanism.

**Scheme 123:**
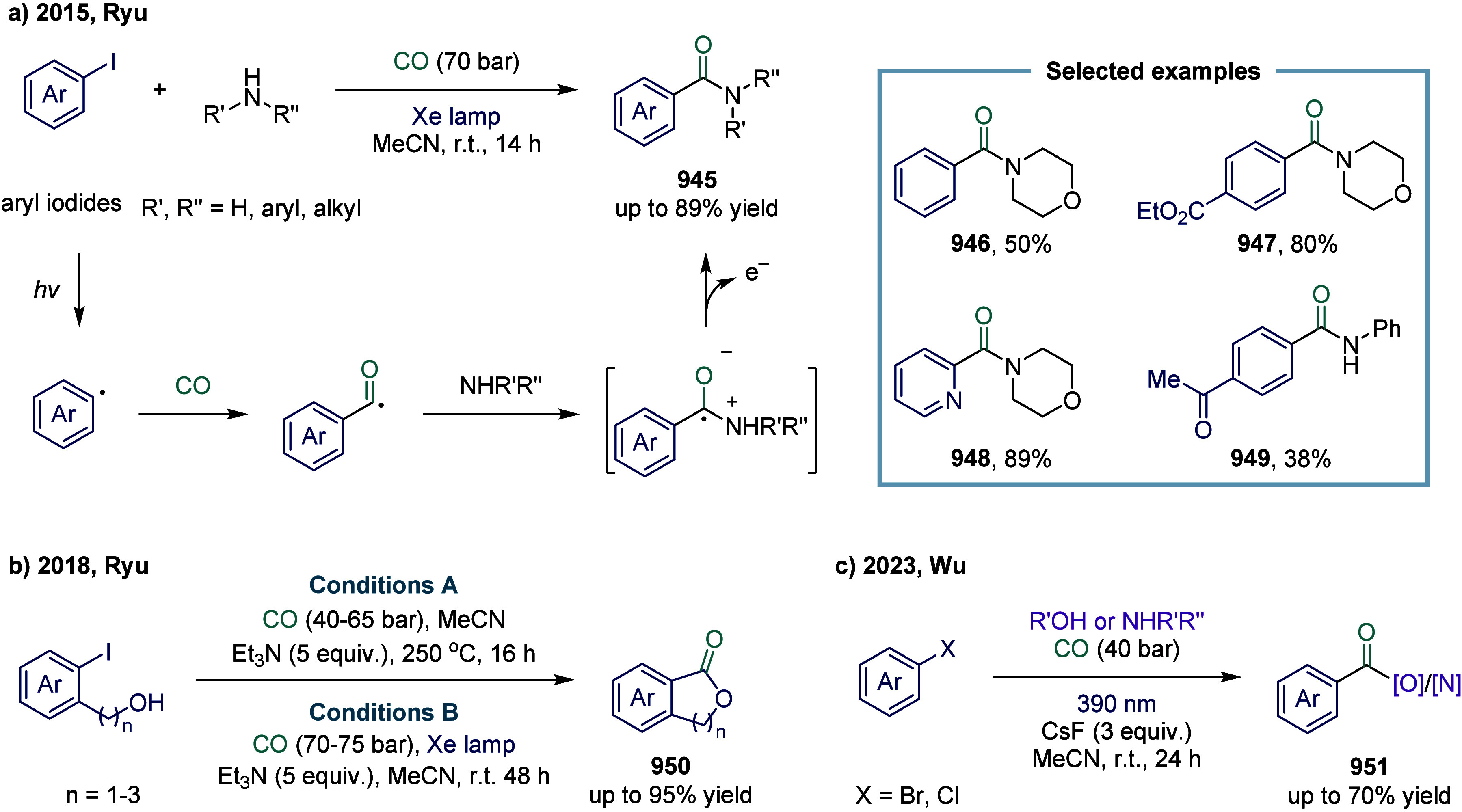
Photo-Induced Carbonylation of Aryl Halides for the Synthesis of Aryl Esters and Amides

While metal-free carbonylative methodologies had demonstrated efficacy with aryl iodides, their application to more inert aryl bromides or chlorides was often impeded by the significantly reduced reactivity of these substrates, thereby limiting the generality of these approaches. In 2023, Wu and co-workers had reported a photoinduced, metal-free carbonylation of aryl bromides for the synthesis of aryl esters and amides **951** in up to 70% yield (Scheme 123c).^497^ In this reaction, despite the higher bond dissociation energy of aryl chlorides, the desired product was obtained in 17% yield. However, the reaction necessitated the use of aryl halides bearing electron-withdrawing groups, such as acetyl and cyano substituents, to achieve acceptable product yields.

#### 5.2.3 Unsaturated Bonds

The Xiao group reported a visible-light-induced radical relay five-component double aminocarbonylation reaction of unactivated alkenes with CO under metal-free conditions (Scheme 124a).^498^ The identification of the dual role of amine coupling partners proved pivotal. Initially, they served as electron donors to generate photoactive EDA complexes with radical precursors. Subsequently, these amines acted as CO acceptors via nitrogen-centered radical cations, thereby furnishing carbamoyl radicals. The resulting carbamoyl radicals then underwent cross-coupling with acyl radicals, which were produced through an alkene relay process, ultimately leading to the formation of the double aminocarbonylation products. A variety of γ-trifluoromethyl α-ketoamides were successfully obtained with good yields and high chemoselectivity. Notably, internal alkenes including cyclohexene and acyclic trans-4-octene were compatible with the five-component radical relay aminocarbonylation, affording the corresponding α-ketoamides **955** and **956** in yields of 29% and 24%, respectively.

**Scheme 124:**
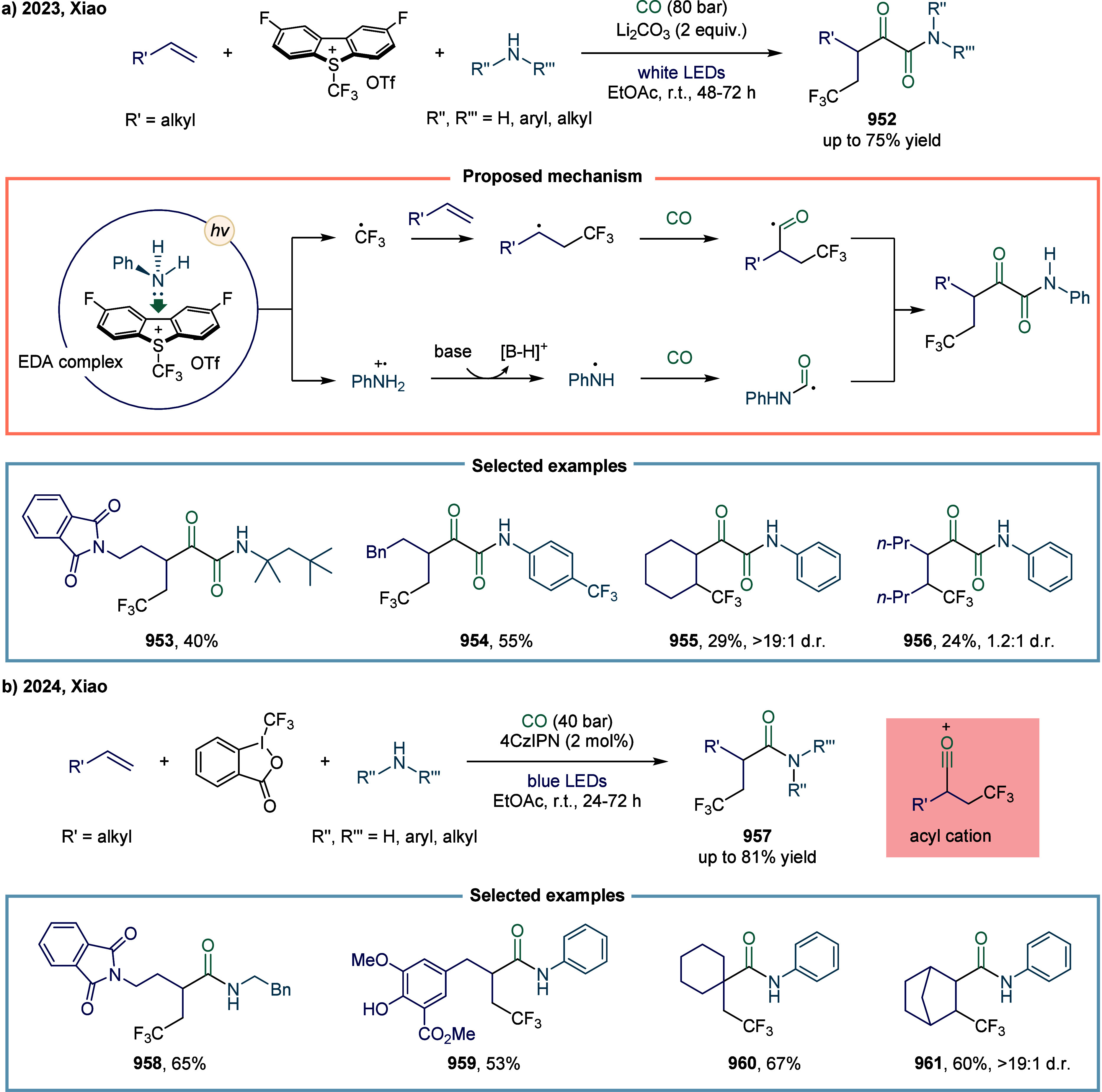
Photo-Induced Multi-Component Radical Relay Carbonylation of Alkenes toward Synthesis of α-Ketoamides and Amides

Despite these impressive advances, most four-component fluoroalkylative carbonylations still rely on transition metal catalysts such as palladium, copper, or cobalt. Given the synthetic importance of β-perfluoroalkyl carboxylic acid derivatives, developing metal-free and broadly applicable four-component SET-mediated carbonylation methods remains highly desirable. In 2024, the Xiao group reported a visible-light-driven radical relay strategy for 1,2-perfluoroalkylation aminocarbonylation of unactivated alkenes, using CO as the carbonyl source and 4CzIPN as an organic photocatalyst (Scheme 124b).^499^ This method tolerated a wide range of alkenes and amines, delivering β-perfluoroalkylated amides **958**-**961** in generally good yields with excellent chemoselectivity. Mechanistic studies suggested that the acyl radical underwent oxidation by the photoexcited 4CzIPN, forming an acyl cation intermediate.

Both experimental and theoretical studies have indicated that γ-position radical migration is generally disfavored due to the formation of energetically strained four-membered transition states.^500^ Wu and co-workers recently developed a visible-light-induced radical relay process in which CO played a pivotal role in promoting molecular rearrangement (Scheme 125a).^501^ The selective incorporation of CO into a carbon-centered radical facilitated subsequent (hetero)­aryl migration, and the rearrangement simultaneously enhanced the efficiency of radical trapping by CO, resulting in a synergistic interplay between the two processes. Under metal-free conditions, this methodology enabled the synthesis of diverse 1,4-dicarbonyl compounds **962** bearing fluoroalkyl and heteroaryl groups in up to 88% yield. Mechanistic studies suggested that visible-light irradiation generated trifluoromethyl radicals, which added to the alkene to give the secondary carbon radical. In the presence of CO, the resulting single-electron species underwent carbonylation to give an acyl radical, which then cyclized via a favorable five-membered transition state to yield the intermediate. Subsequent C–C bond homolysis, driven by aromatization, induced heteroaryl migration and furnished the α-hydroxy radical. Oxidation of radical species by the excited state photocatalyst, followed by deprotonation, delivered the final product **962**. Notably, this rearrangement strategy was not limited to heteroaryl substrates. A range of diaryl and monoaryl compounds were also compatible, affording the corresponding carbonylated products **964** and **966** in moderate yields of 48% and 57%, respectively. Then, the same group developed a visible light-promoted phosphorylation carbonylation reaction of unactivated alkenes via (hetero)­aryl migration (Scheme 125b).^502^

**Scheme 125:**
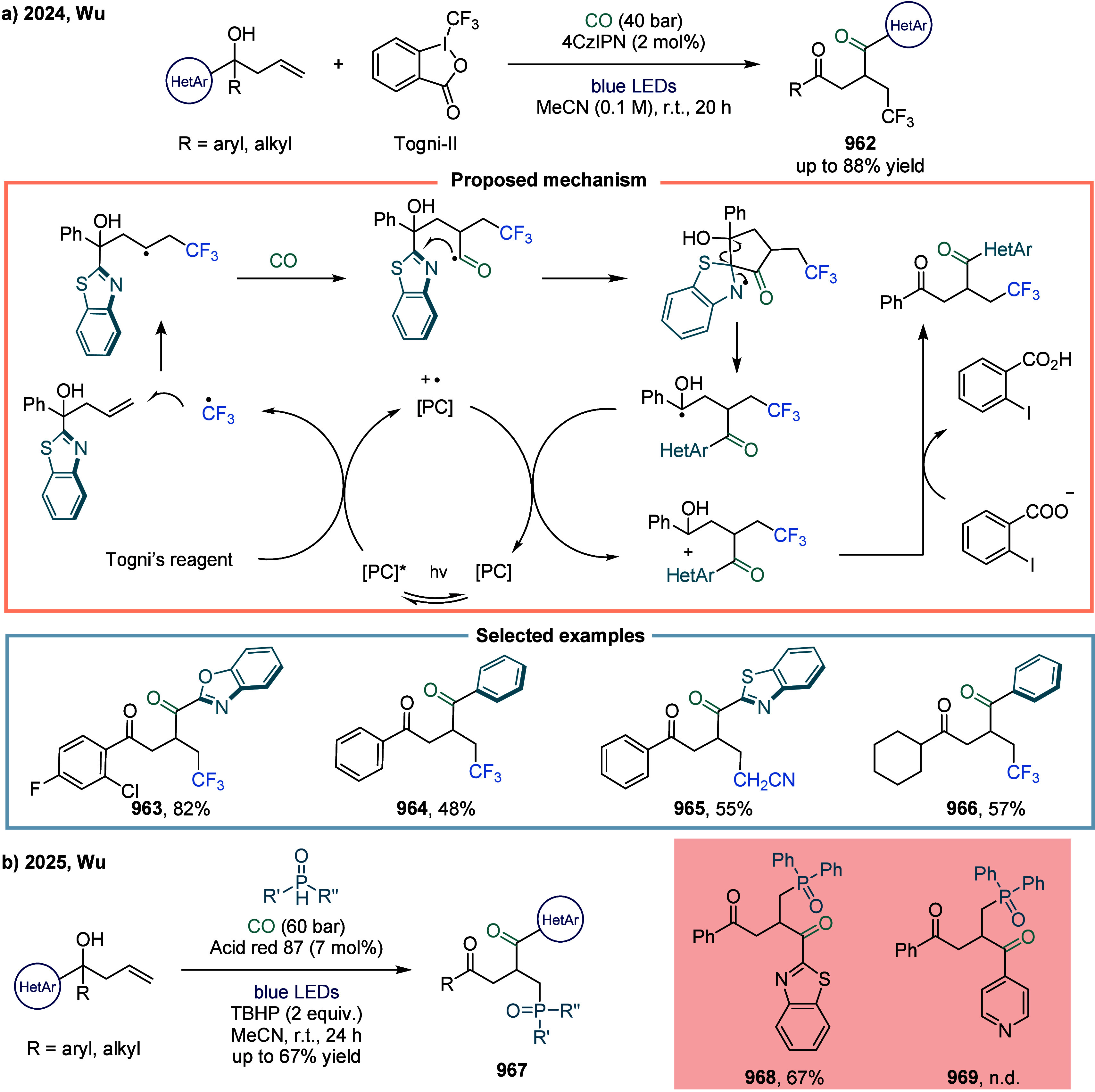
Carbon Monoxide Enabling Synergistic Carbonylation and (Hetero)­aryl Migration

In 2016, Gaunt and co-workers reported a general palladium-catalyzed strategy for the β–C-H carbonylation of aliphatic amines to access β-lactams.^503^ Inspired by the rising interest in photoinduced SET-mediated carbonylation under metal-free conditions, Wu and co-workers envisioned that β-amino acyl radicals could be generated via a tandem radical carbonylation sequence starting from readily available allylamines (Scheme 126).^504^ Upon photoexcitation, single-electron reduction of radical precursors generated electrophilic radicals, which added to the C=C bond of allylamine to form alkyl radicals. In the presence of CO, it captured CO to give β-amino acyl radicals. Oxidation of this species by the photocatalyst yielded acyl cation, which underwent intramolecular nucleophilic attack to furnish the β-lactam products **970**. Notably, the β-amino acyl radical intermediate exhibited high cyclization efficiency. A broad range of primary amines, including alkyl, aryl, benzyl, and amino acid derivatives, were found to be compatible and delivered β-lactam products **971**-**973** in good yields. In contrast, substrates bearing *gem*-dimethyl substitution, such as compound **974**, completely suppressed the reaction, likely due to steric hindrance. Moreover, the compatibility with electron-deficient radicals enabled the introduction of cyano and trichloromethyl substituents into the β-lactam scaffold, such as compounds **975** and **976**.

**Scheme 126:**
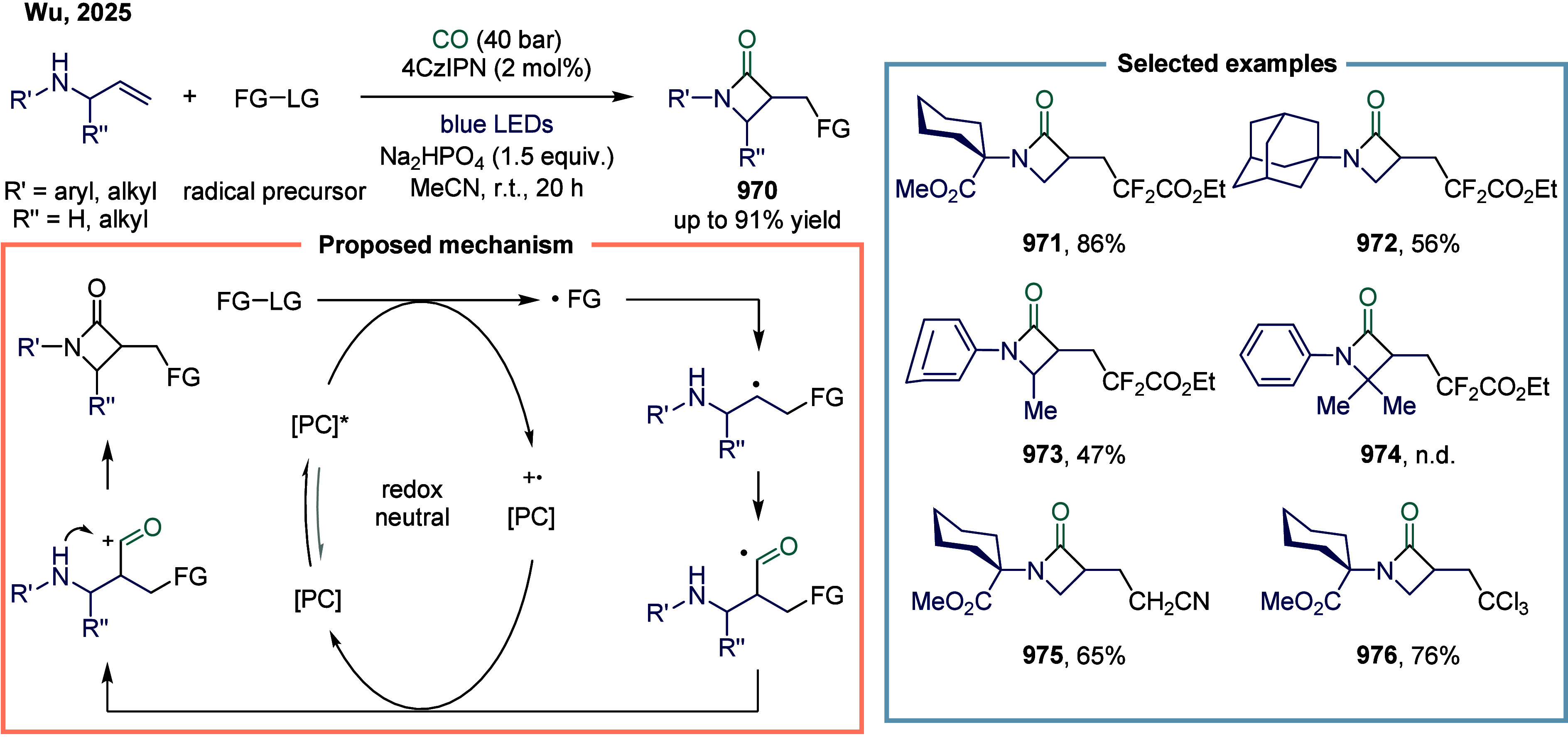
Photo-Induced Carbonylative Annulation Access to *β*-Lactams

In 2025, Wu and co-workers disclosed a divergent radical tandem carbonylation of multisubstituted homoallylic alcohols, which enabled the selective synthesis of γ-lactones **977** and 1,4-diones **978** (Scheme 127).^505^ The reaction pathway depended on the electron donor used, where quinuclidine directed the process toward lactonization, while DIPEA facilitated aryl migration to the carbonyl carbon. A series of substrates bearing various aromatic substitutions was investigated, delivering γ-lactones **978**-**983** and 1,4-diones **984**-**988** in good yields with excellent chemo-selectivity. Mechanistic studies revealed that an EDA complex formed between Togni’s-II reagent and the tertiary amine, generating both trifluoromethyl radicals and tertiary amine radical cations. The CF_3_ radical added to the olefin substrate to afford a secondary carbon radical. Under a CO atmosphere, this radical was trapped to form the acyl radical intermediate. Subsequent tertiary amine control afforded intermediates **989** and **990**, which underwent intramolecular lactonization and radical addition, respectively, furnishing products **977** and **978**.

**Scheme 127:**
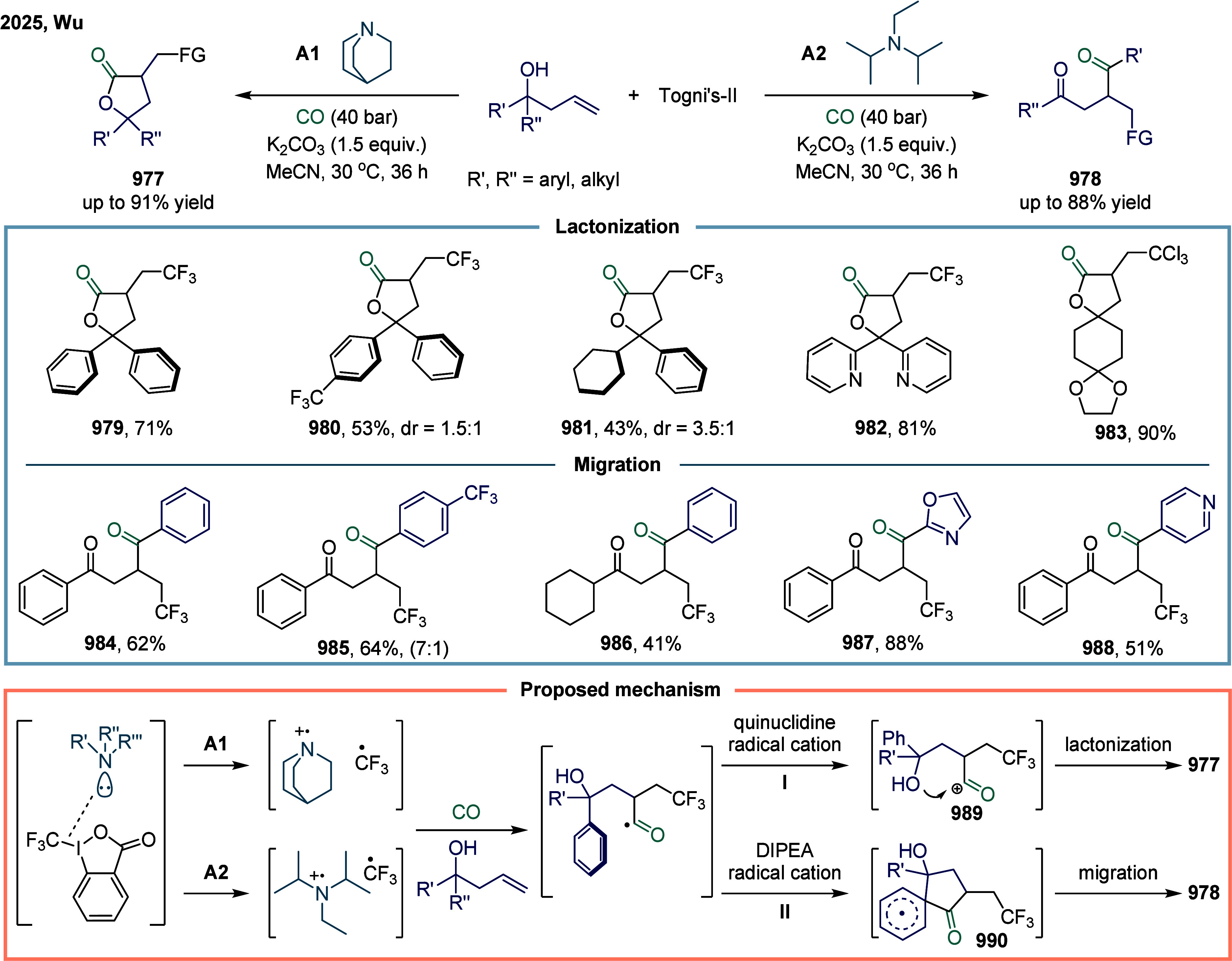
Amine-Tuned Controllable Carbonylation for the Synthesis of γ-Lactones and 1,4-Diones

#### 5.2.4 Others

In 2016, Li and co-workers developed a desulfonylation strategy that enabled carbonylative coupling between aryl sulfonyl chlorides and indoles (Scheme 128).^506^ This metal-free protocol exhibited broad functional group tolerance and eliminated the need for transition-metal catalysts, additives, or acidic/basic reaction conditions. Subsequently, in 2021, Peng and collaborators reported a visible-light-driven SET carbonylation of indoles with phenols (Scheme 129).^507^ This methodology afforded various aryl indole-3-carboxylates **996–998** in moderate to good yields.

**Scheme 128:**
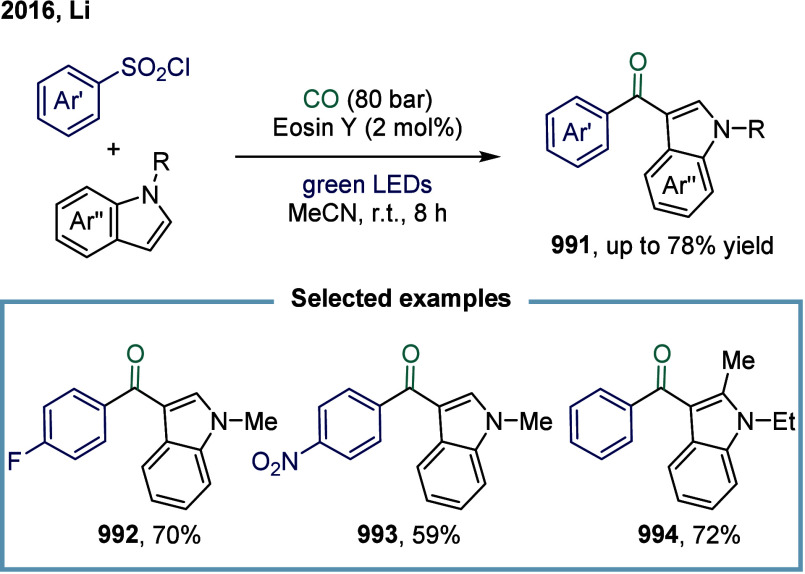
Visible-Light-Induced Carbonylation of Indoles with Arylsulfonyl Chlorides and CO

**Scheme 129:**
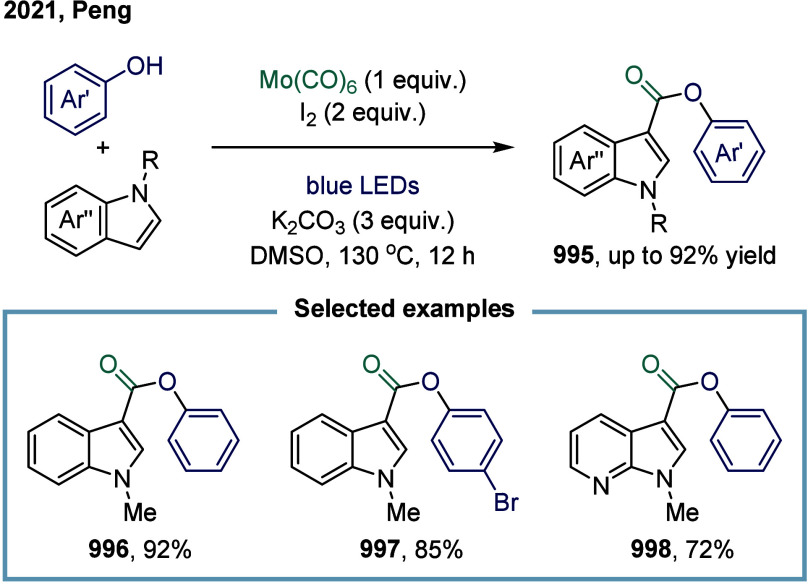
Visible-Light-Induced Carbonylation of Indoles with Phenols: Synthesis of Indole-3-carboxylates

In 2018, the Fensterbank and Ryu groups collaboratively investigated a photocatalytic carbonylation reaction that employed alkyl silicates as alkyl radical precursors (Scheme 130a).^508^ Using the organic dye 4CzIPN, a potent single-electron oxidant, as a photoredox catalyst, alkyl radicals were efficiently generated and subsequently trapped by CO to afford dialkyl ketones **999** via a radical pathway. In the presence of 4CzIPN, the reaction of alkyl silicates with CO and electron-deficient alkenes provided a range of unsymmetrical dialkyl ketones **1000–1002** in good yields. In 2020, Fensterbank and Ryu further extended this strategy to the synthesis of amides. In the presence of 4CzIPN and carbon tetrachloride (CCl_4_), various amides were synthesized in up to 89% yield (Scheme 130b).^509^ CCl_4_ acted as an acyl radical trapping agent, enabling the in situ generation of acyl chlorides, which served as key intermediates for amide bond formation.

**Scheme 130:**
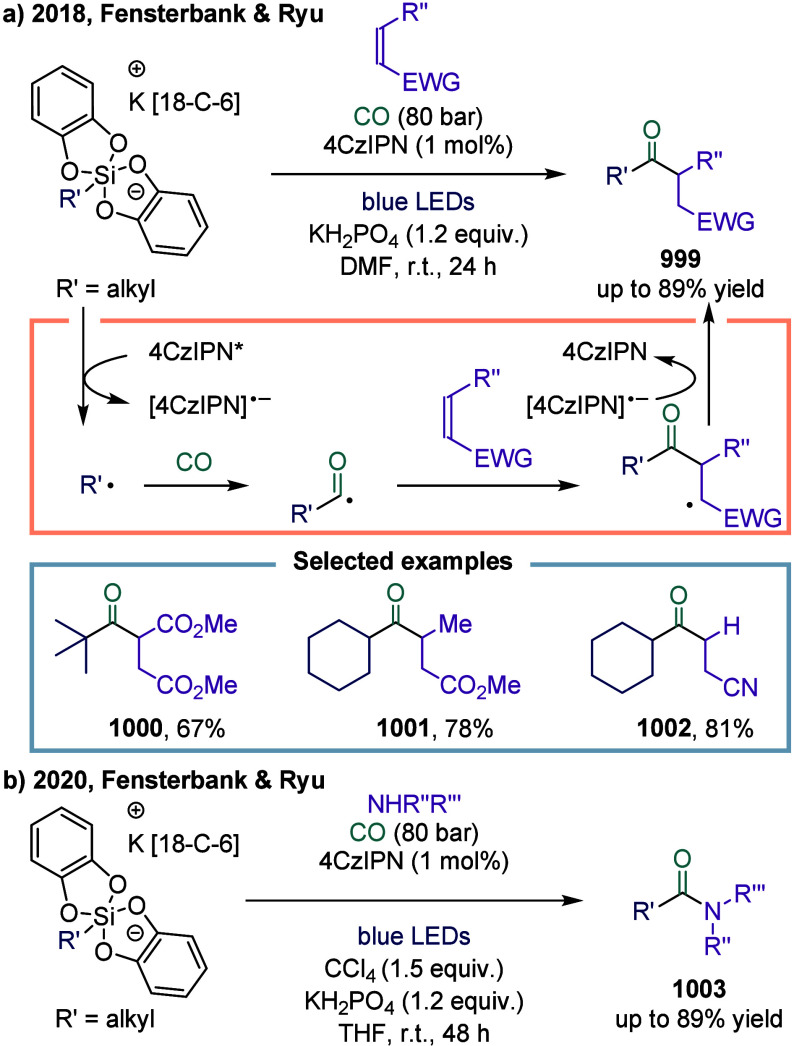
Carbonylation of Alkyl Radicals Derived from Organosilicates through Visible-Light Photoredox Catalysis

In 2025, a visible-light-driven metal-free carbonylation strategy was reported for the efficient synthesis of various acylsilanes **1004** under mild conditions (Scheme 131).^510^ The reactions proceeded via the in situ generation of silyl radicals, which underwent carbonylation with CO to afford silyl acyl radical intermediates. These intermediates were trapped by various reagents to generate new carbon-centered radical species, which were subsequently reduced by reductants to afford carbanions, followed by protonation to furnish the target acylsilanes. When HAT catalyst **A** was employed, the reaction likely proceeded via a radical chain mechanism, affording the acylsilane products **1004**. The transformation demonstrated broad compatibility with acyl radical trapping reagents, including monosubstituted, 1,1-disubstituted, and 1,2-disubstituted alkenes, providing the corresponding products **1006**-**1009** in moderate yields and excellent selectivity. However, the substrate scope with respect to silanes was limited, with satisfactory efficiency observed only for specific examples such as triethylsilane and *tert*-butyldimethylsilane.

**Scheme 131:**
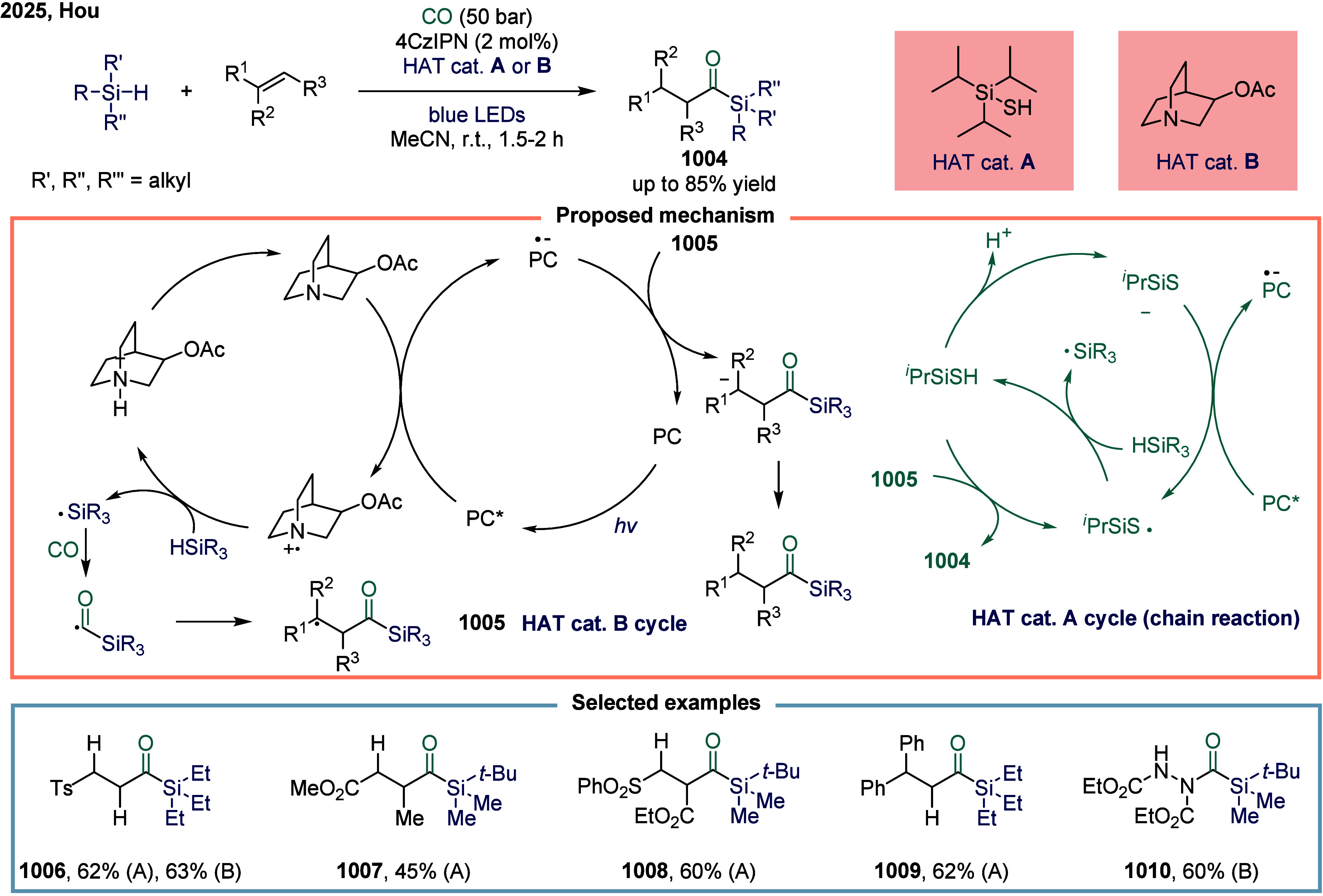
Visible-Light-Mediated Metal-Free Approach for Generating Silyl Acyl Radical Intermediates

In 2014, Wangelin and co-workers developed a metal-, ligand-, and base-free carbonylation protocol for synthesizing benzoic acid derivatives **1012** (Scheme 132a).^511^ Mechanistic studies revealed that electron-deficient aryl diazonium salts underwent SET processes from the photoexcited states of eosin Y (EY*), releasing N_2_ and generating aryl radical. These intermediates rapidly combined with CO to form the corresponding acyl radicals, which were subsequently oxidized to highly electrophilic acylium ions. The acylium ions then reacted readily with alcohols to afford the corresponding ester products. The rapid back electron transfer (BET) process occurred, and no adduct formation between the aryl radical and electron-rich donors was observed. DFT calculations supported a photoredox catalytic pathway proceeding without the need for sacrificial redox agents. In parallel, the Xiao group reported a visible-light-induced alkoxycarbonylation of aryl diazonium salts employing fluorescein as the photocatalyst (Scheme 132b).^512^ Upon irradiation with a 16 W blue LED under 80 atm CO pressure, the reaction of aryl diazonium salts with various alcohols in the presence of 3 mol % fluorescein afforded the corresponding benzoic acid derivatives in up to 85% yield. Notably, the method tolerated iodine substituents, which are typically incompatible with conventional metal-catalyzed carbonylation reactions, yielding product **1017** in 50% yield. A wide range of alcohols bearing diverse functional groups was applicable, including diols **1018** and alkynyl alcohols **1019**.

**Scheme 132:**
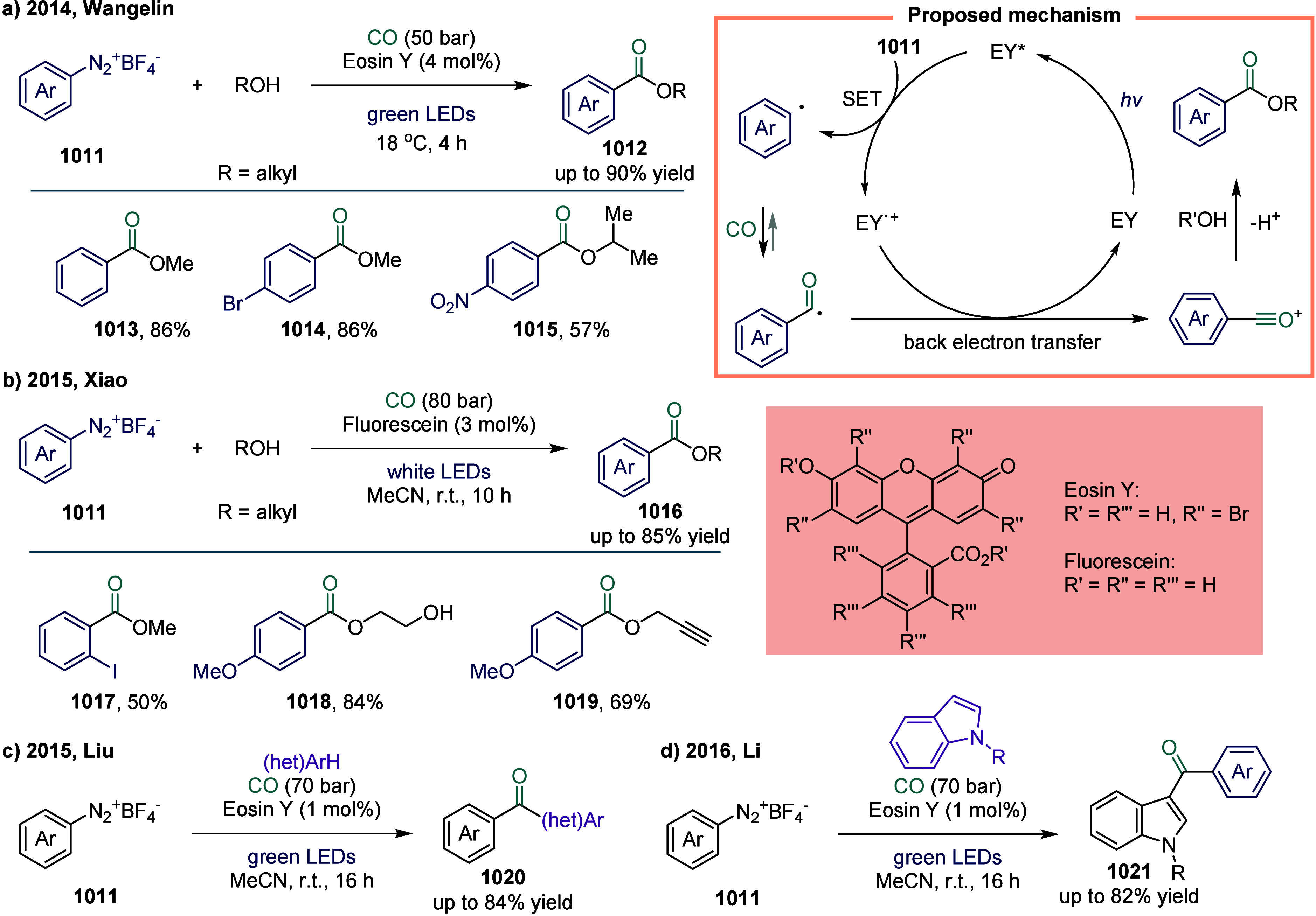
Visible-Light-Mediated Metal-Free Carbonylation of Aryldiazonium Salts

In 2015, Liu and co-workers extended the metal-free, visible-light-mediated transformation of aryl diazonium salts with (hetero)­arenes (Scheme 132c).^513^ Various (hetero)­aryl ketones **1020** were prepared via this transformation in up to 84% yield. In 2016, Li and co-workers reported a visible-light-catalyzed synthesis of indol-3-yl aryl ketones **1021** in up to 82% yield from aryldiazonium salts, carbon monoxide, and indoles at room temperature (Scheme 132d).^514^

In 2024, Wu and co-workers reported a photochemical carbonylation method of aryl sulfonium salts via photoexcitation of EDA complexes (Scheme 133).^515^ By selecting different amines, reaction intermediates could be sequentially trapped and quenched. Specifically, using DBU as the electron donor directed the intermediates toward acylium ion formation, whereas employing DMAP resulted in the generation of reactive aryl acyl-DMAP salts **1023**. This approach, combined with aryl C–H sulfonium formation, enabled selective C–H carbonylation of arenes to synthesize esters and amides under metal-free conditions. The versatility of this protocol was demonstrated by its compatibility with various functionalized reagents, including phenols, alkyl alcohols, anilines, and alkyl amines, making it a powerful strategy for the synthesis of aryl carboxylic acid derivatives relevant to medicinal chemistry.

**Scheme 133:**
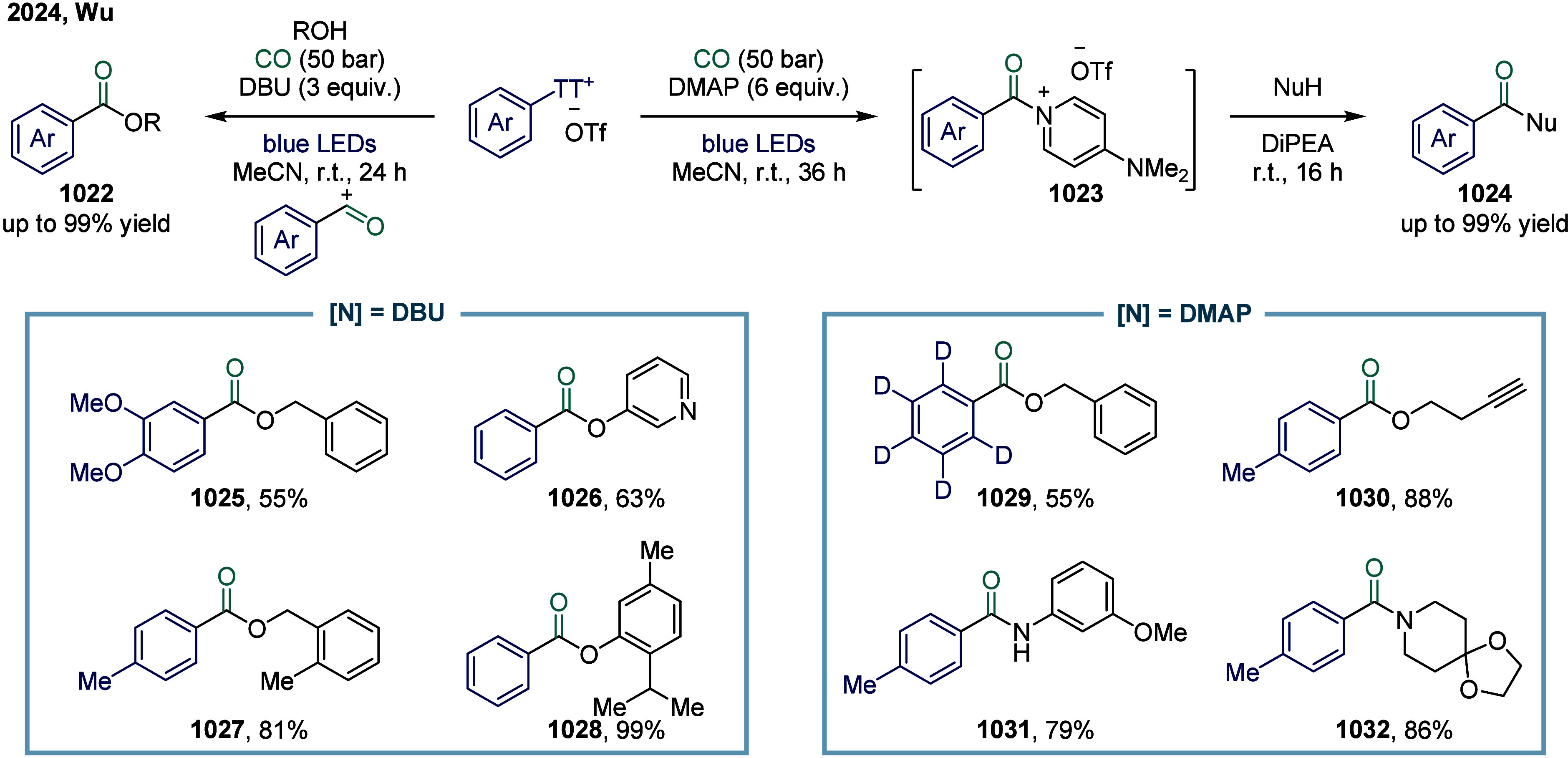
Tertiary Amine-Promoted Photoactivation Metal-Free Carbonylation of Aryl Sulfonium Salts to Aryl Carboxylic Acid Derivatives

## 6 Conclusions and Outlook

Carbonylation reaction is a fast-growing field that has had and will continue to impact the many research areas of synthetic chemistry. Although numerous mini reviews have addressed specific reaction classes or mechanistic facets of SET carbonylation, a unified and comprehensive treatment encompassing the full breadth of this rapidly evolving field has been lacking. This review fills this gap by systematically surveying SET carbonylation chemistry from 2000 to July 2025, highlighting key mechanistic insights, catalytic innovations, and synthetic applications. SET strategies uniquely exploit the high reactivity of alkyl and aryl radicals, which can be generated from a broad range of abundant chemical bonds, including otherwise inert C–H, C–X (halogen), and unsaturated moieties. In contrast to traditional two-electron transfer mechanisms that often rely heavily on precious metal catalysts and face limitations with challenging substrates, SET-mediated carbonylation offers a versatile and sustainable alternative. Its distinctive capability to activate unactivated C–H and C–X bonds and various other linkages under mild conditions, coupled with efficient in situ incorporation of carbon monoxide, significantly expands the synthetic toolbox for the construction of carbonyl-containing compounds. Special emphasis is placed on diverse carbonyl incorporation strategies under both metal-catalyzed and metal-free conditions, engaging a wide variety of alkyl, aryl, heteroatom, and π-bond-containing substrates. The strategic control of radical generation, relay, and termination emerges as a central theme enabling access to previously inaccessible bond constructions and substantially expanding the synthetic utility of carbonylation.

Despite remarkable progress and a growing body of literature, several critical challenges and opportunities remain unaddressed, providing fertile ground for future exploration. One promising direction lies in the design and development of novel hydrogen atom transfer (HAT) reagents derived from earth-abundant, inexpensive, and environmentally benign sources. Such reagents hold the potential to achieve unprecedented levels of site- and chemoselectivity in radical generation, thereby enabling highly controlled carbonylation processes. Moreover, broadening the substrate scope to include less reactive chemical bonds, such as C–F, C–O, and C–C bonds, would not only enhance the versatility of SET carbonylation but also push the boundaries of catalyst design and mechanistic understanding. Equally important is the establishment of universal catalytic platforms capable of efficiently capturing CO under low pressure or directly using CO_2_ as the CO source. Such platforms would not only improve the operational simplicity and sustainability of these transformations but also facilitate the incorporation of isotopically labeled CO, a critical feature for applications in pharmaceutical synthesis and molecular imaging. Mechanistic studies, including computational modeling and in situ spectroscopic techniques, are essential for elucidating reaction pathways and optimizing catalyst performance.

In conclusion, the advent of SET-mediated carbonylation transformations has brought with it innumerable opportunities in the streamlining of syntheses to uncover novel fundamental reactivity. SET-mediated carbonylation stands at the forefront of carbonylation chemistry, offering innovative solutions to longstanding synthetic challenges. Continued efforts toward expanding reagent diversity, substrate scope, and catalytic efficiency promise to unlock new horizons in the synthesis of complex carbonyl architectures, thereby enriching the landscape of modern organic synthesis.
